# Distinct molecular phenotypes involving several human diseases are induced by IFN-λ3 and IFN-λ4 in monocyte-derived macrophages

**DOI:** 10.1038/s41435-022-00164-w

**Published:** 2022-02-03

**Authors:** Manjarika De, Anand Bhushan, William S. Grubbe, Subhajit Roy, Juan L. Mendoza, Sreedhar Chinnaswamy

**Affiliations:** 1 National Institute of Biomedical Genomics, Kalyani, West Bengal 741251 India; 2 Pritzker School of Molecular Engineering and Department of Biochemistry and Molecular Biology, University of Chicago, Chicago, IL USA; 3 Present Address: Cleveland Clinic Cole Eye Institute & Lerner Research Institute, Cleveland, OH 44195 USA

**Keywords:** Interferons, Gene expression profiling

## Abstract

Human Interferon (IFN) lambda 3 (IFN-λ3) and IFN-λ4 are closely linked at the *IFNL* locus and show association with several diseases in genetic studies. Since they are only ~30% identical to each other, to better understand their roles in disease phenotypes, comparative studies are needed. Monocytes are precursors to macrophages (monocyte-derived macrophages; MDMs) that get differentiated under the influence of various immune factors, including IFNs. In a recent study, we characterized lipopolysaccharide-activated M1 and M2-MDMs that were differentiated in presence of IFN-λ3 or IFN-λ4. In this study, we performed transcriptomics on these M1 and M2-MDMs to further understand their molecular phenotypes. We identified over 760 genes that were reciprocally regulated by IFN-λ3 and IFN-λ4, additionally we identified over 240 genes that are significantly affected by IFN-λ4 but not IFN-λ3. We observed that IFN-λ3 was more active in M2-MDMs while IFN-λ4 showed superior response in M1-MDMs. Providing a structural explanation for these functional differences, molecular modeling showed differences in expected interactions of IFN-λ3 and IFN-λ4 with the extracellular domain of IFN-λR1. Further, pathway analysis showed several human infectious diseases and even cancer-related pathways being significantly affected by IFN-λ3 and/or IFN-λ4 in both M1 and M2-MDMs.

## Introduction

The type III interferon (IFN) locus on chromosome 19 in humans has four genes: IFN-λ1-4 [1–3], that are known to be under strong evolutionary pressure [4, 5]. Even though they have potent antiviral properties, and are classified as IFNs, they structurally resemble members of the IL-10 superfamily [6]. IFN-λ1-3 were discovered in the year 2003 [1, 2]; however, IFN-λ4 was discovered more recently (2013) during follow-up studies to the genome-wide association studies of 2009 that were conducted to identify the genes behind clearance of hepatitis C virus in humans [3]. There are two sets of genetic variants identified at the *IFNL* locus that control: 1) expression of IFN-λ3 [7]; and 2) expression and activity of IFN-λ4 [8, 9]. These genetic variants are being increasingly reported to be associated with various infectious and inflammatory diseases [10]. IFN-λ4 is only ~30% identical to IFN-λ3 [3] but is reported to be equally potent in its in vitro signaling and antiviral activities [11, 12]. Interestingly, genetic studies have shown strong evidence for the involvement of IFN-λ4, but not IFN-λ3, in many infectious disease conditions [13–16]. Besides, we see that IFN-λ4 is present in a significant proportion of the world population [17]. This is despite IFN-λ4 being poorly secreted by cells [12, 18, 19], not being induced in many cell types [19] and poorly transcribed due to weak promoter activity [20]. Other genetic studies suggest that IFN-λ3 and not IFN-λ4 may be responsible for certain inflammatory conditions in the liver and lungs [21]. Strong linkage disequilibrium, between the variants regulating expression of IFN-λ3/4, in a majority of the modern human populations poses a problem in correctly identifying whether IFN-λ3 or IFN-λ4 is behind the disease phenotypes [9]. Therefore, comparative studies on IFN-λ3 and IFN-λ4, to better understand their functions, are needed.

## Materials and methods

### Generation of M1 and M2 macrophages from CD14^+^ monocytes, RNA sequencing (RNA-seq) and bioinformatics

The protocol for generation of M1 and M2-MDMs (monocyte-derived macrophages) in presence of recombinant IFN-λ3/4 has been described in our earlier study [22]. Briefly, 10 million CD14^+^ monocytes (PromoCell, Heidelberg, Germany) each, obtained from four unrelated Caucasian donors were grown in RPMI-1640 medium supplemented with antibiotics and 10% FBS (all from Gibco, Waltham, MA, USA). Human recombinant IFN-λ4 (catalog #9165-IF; carrier-free form; *E. coli*­-derived) and IFN-λ3 (catalog #5259-IL/CF; Chinese Hamster Ovary cell line, CHO-derived), containing <0.1 EU/µg of endotoxin purchased from R&D Systems (Minneapolis, MN, USA) were included in the differentiation medium at 0.05 µg/ml and 6 µg/ml for IFN-λ3 and IFN-λ4 respectively. These concentrations gave comparable interferon stimulated gene (ISG) induction by the two IFNs tested on different cell types as was determined in our earlier study [22]. After six days of differentiation, lipopolysaccharides (LPS) at 100 ng/ml (Sigma-Aldrich, St. Louis, MO, USA) was used to stimulate the MDMs before processing them for RNA isolation. Total RNA was isolated using RNeasy kit (Qiagen, Hilden, Germany) and sent to Quickbiology, Pasadena, CA, USA, where RNA-seq and all the raw data analysis were carried out. The detailed protocol of RNA-seq is described in our recent paper [22]. Fastqc software was used to check the data quality of original sequencing data. TMM (trimmed mean of *M*-values) method in edgeR package was used to normalize the gene expression. The reads were first mapped to the latest UCSC transcript set using Bowtie2 version 2.1.0 [23] and the gene expression level was estimated using RSEM v1.2.15. [24]. Differentially expressed genes (DEG) were identified using the edgeR program [25]. Genes showing altered expression with *p* < 0.05 and more than 1.5-fold changes (FC) were considered as DEGs. All the sequence files (raw and processed) have been deposited in gene expression omnibus (GEO; series accession number GSE182823). Goseq [26] was used to perform the GO (gene ontology) enrichment analysis and Kobas [27] was used to perform the pathway analysis using KEGG (Kyoto Encyclopedia of Genes and Genomes) and Reactome databases. The principal components analysis (PCA) function in the FactoMineR package was used to calculate the coordinates of the principal component (PC1 and PC2) based on the gene expression of each sample. Ggplot2 was used for the plotting of each sample.

### Molecular modeling

RaptorX [28] was used to create a molecular model of IFN-λ4, and the structure of wild type IFN-λ3 was obtained from the Protein Data Bank in Europe (PDB ID 3HHC). These structures were then aligned to the known structure of IFN-λ in complex with its receptors (PDB ID 5T5W) [29] using PyMOL (The PyMOL Molecular Graphics System, Version 2.4 Schrödinger, LLC). Predicted polar contacts formed between the models and their receptors were also determined using PyMOL.

## Results

### Many genes are perturbed in LPS-activated MDMs that are differentiated in presence of IFN-λ3 or IFN-λ4

In our recent study [22], we had showed that recombinant IFN-λ4 when incorporated in the medium during differentiation of THP-1 cells, had altered the phenotypes of the resulting macrophage-like cells [22]. Among other changes, IFN-λ4 caused differences in surface marker expression in LPS-activated THP-1-derived macrophage-like cells [22]. To compare the effects of IFN-λ3 and IFN-λ4, we differentiated THP-1 cells into macrophage-like cells using established protocols [22] in presence of IFN-λ3 or IFN-λ4 and carried out flow cytometry analysis of several surface markers (Suppl. Fig. 1). Firstly, of all the markers tested, CD83, a dendritic cell activation marker, was expressed at high levels when macrophage-like cells were incubated with either IFN-λ3 or IFN-λ4, compared to control untreated cells, confirming that IFN-λ treatment during macrophage differentiation can influence their phenotypes [22]. Secondly, IFN-λ3 and IFN-λ4 showed differences between each other in influencing the expression of some surface markers like CD16, CD80 and CD83, however they were not highly significant (Suppl. Fig. 1).

**Fig. 1:**
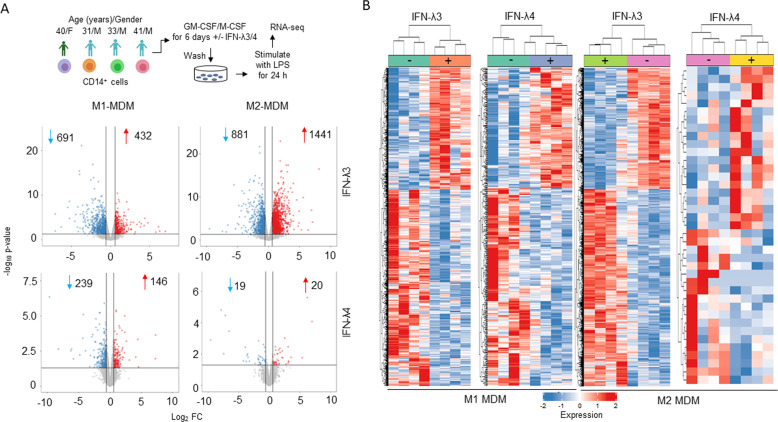
Results from RNA-seq of activated M1 and M2-MDMs differentiated in presence of IFN-λ3 and IFN-λ4. **A** Schematic shows the study design. Four CD14+ cell samples derived from four unrelated Caucasian individuals were obtained and differentiated into M1 or M2-MDMs for six days in presence or absence of respective IFN-λs. After thoroughly washing to remove all traces of growth factors and IFNs, the differentiated MDMs were activated in presence of LPS for an additional 24 h and cells were washed, and RNA was isolated before proceeding for RNA-seq. Lower panel shows the volcano plots generated from genes altered in expression when doing the comparison in the respective sequencing data sets (IFN-λ3 or IFN-λ4 vs. NT). DEGs are those with FC more than or equal to 1.5 and *p* < 0.05 and are shown as red (upregulation), or blue dots (downregulation); gray dots are genes that are insignificantly affected for expression. **B** Heat maps showing DEGs: 1123 for IFN-λ3 vs. NT, 385 for IFN-λ4 vs. NT for M1-MDMs; 2322 for IFN-λ3 vs. NT, 39 for IFN-λ4 vs. NT for M2-MDMs. NT-no treatment. Each row is a gene, and each column is a sample from one individual.

In the previous study, we reported that LPS-activated M1 or M2-MDMs differentiated in presence of recombinant human IFN-λ3 or IFN-λ4 were affected for cytokine expression when compared to IFN-λ-untreated cells [22]. Importantly, we had shown that IFN-λ4 conferred an anti-inflammatory phenotype to M1-MDMs as we saw that M1-MDMs differentiated in presence of IFN-λ4 induced more IL-10 secretion while those differentiated in presence of IFN-λ3 induced lesser IL-10 secretion when compared to untreated cells [22] hinting that IFN-λ3 and IFN-λ4 could be inducing opposite phenotypes in these cells. In this study, we used the same M1 and M2-MDMs used in the earlier study [22], to perform RNA-seq and to further characterize the molecular phenotypes induced by the two IFNs. The schematic in Fig. 1A shows the study design. After six days of differentiation in presence or absence of either IFN-λ3 or IFN-λ4 in M1 (GM-CSF) or M2 (M-CSF) conditions [22], the macrophages were activated with LPS for 24 h in the absence of any IFN-λs, after which the total cellular RNA was isolated and RNA-seq was performed. As per the study design, the affected genes will be a direct outcome of the effect of LPS on the no treatment (NT) or IFN-λ3/4-treated cells; however, we compared the mean FC values of the affected genes in the four independent samples between IFN-λ3/4 vs. NT or IFN-λ3 vs. IFN-λ4 treatment so that the specific effect of LPS would cancel out and the differences in gene expression would reflect only those arising due to IFN-λ3/4 treatment.

The results showed that 1123 genes were significantly affected in M1-MDMs treated with IFN-λ3 while only 385 genes were significantly affected by IFN-λ4 in relation to NT (Fig. 1A, B). Results in M2-MDMs showed a different pattern. Firstly, more than 2300 genes were significantly affected by IFN-λ3 treatment while only 39 genes were significantly perturbed by IFN-λ4 treatment, in relation to NT. Interestingly, while a greater number of genes were upregulated in M2-MDMs treated with IFN-λ3 compared to downregulated genes, there were more downregulated genes than upregulated genes in M1-MDMs treated with either IFN-λ3 or IFN-λ4. Surprisingly, instead of showing more affected genes in M2-MDMs than M1-MDMs (like IFN-λ3-treated cells), IFN-λ4 treatment showed a greatly diminished number of perturbations in gene expression in M2-MDMs.

**Fig. 2:**
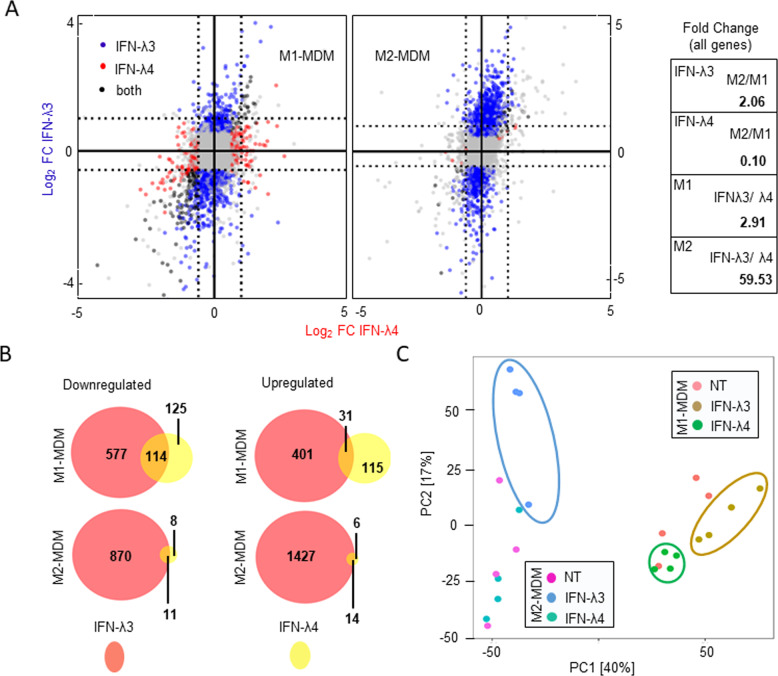
IFN-λ3 and IFN-λ4 have distinct effects on gene expression in differentiating M1 and M2-MDMs. **A** Scatter plots showing distinct effects of IFN-λ3 and IFN-λ4 on M1 and M2-MDMs. Each dot is a gene: gray dot denotes a gene that was insignificantly affected (*p* > 0.05 and FC < 1.5); blue dot is a gene that was specifically and significantly affected by IFN-λ3 while red dot shows the gene that was specifically and significantly affected by IFN-λ4; black dot denotes a gene affected significantly by both IFN-λ3 and IFN-λ4. The *X*-axis shows FC for IFN-λ4-affected genes, *Y*-axis is for IFN-λ3-affected genes. The dotted lines show FC = 2.0 for upregulated genes and 1.5 for downregulated genes. The table to the right shows some proportions of changes in total (both upregulated and downregulated) genes (only significantly affected ones) that were altered in the different conditions shown. **B** Venn diagram showing the unique and common set of significantly affected genes in the conditions shown. **C** PCA plot showing the distribution of the four samples after multidimensional scaling according to gene expression. Each dot represents an individual sample. The ellipses are drawn to separate out the different clusters for better visualization. NT no treatment.

Next, we examined if similar or different set of genes were perturbed by the two IFN-λs in the stimulated MDMs that we had obtained (Fig. 2A, B). Surprisingly, unlike the results from previous studies [30, 31], we saw that most of the genes affected by either IFN-λ3 or IFN-λ4 were unique in M1-MDMs, the cell type, where IFN-λ4 had a larger effect. When we carried out principal components analysis (PCA) to see if the genes affected by IFN-λ4 indeed segregate as a cluster, we saw that the most differentiated cluster (among the IFN-treated cells) was formed by IFN-λ4-treated M1-MDMs, which was well-separated from IFN-λ3-treated M1-MDMs. The next distinct cluster was formed by IFN-λ3-treated cells in the M2-MDM type, while IFN-λ4-treated cells clustered along with the NT control cells (Fig. 2C). These results also indicate that our experiments in differentiating monocytes to the respective macrophage types have worked well since the M1 and M2-MDMs have clear separation from each other in the PCA plot. They also show that IFN-λ4 had a unique and strong effect on differentiating macrophages under M1 but not M2 conditions. Further, while IFN-λ3 had an ~2-fold increase in the number of genes affected in M2 vs. M1-MDMs, IFN-λ4 had a disproportionate (~10-fold) decrease in genes that it targeted in M2 vs. M1-MDMs (Fig. 2A, table). Similarly, in M1-MDMs, there was ~3-fold greater number of genes perturbed by IFN-λ3 than IFN-λ4 while this difference increased to ~60-fold in M2-MDMs (Fig. 2A, table). These results suggest that IFN-λ3 and IFN-λ4 can have preferential effects on M1 and M2-MDMs, even though they use the same receptors for signaling [3, 6, 18].

**Fig. 3:**
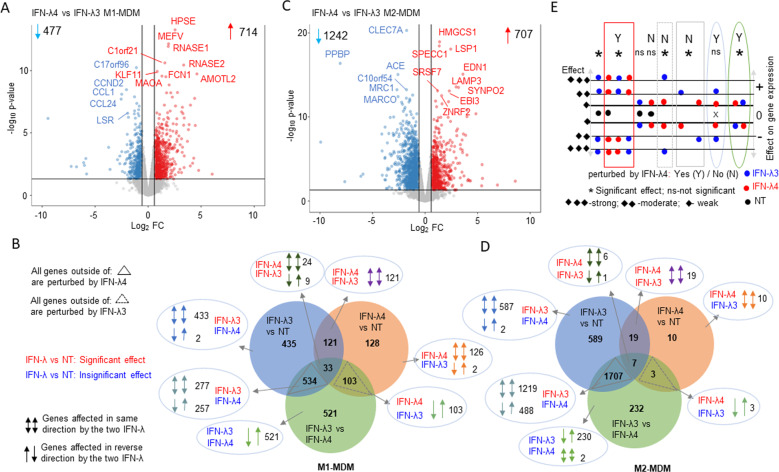
IFN-λ4 has both significant and insignificant effects on many genes in differentiating M1 and M2-MDMs. Volcano plots showing DEGs in M1-MDMs (**A**) or M2-MDMs (**C**) treated with IFN-λ3 or IFN-λ4 during differentiation; some of the strongly and significantly affected genes are named. **B** (for M1-MDMs) and **D** (for M2-MDMs) shows Venn diagram analysis for depicting the unique and common DEGs affected between the three comparison sets shown. The genes within solid and dashed triangles are considered ‘unperturbed’ by IFN-λ3 and IFN-λ4 respectively. NT-no treatment. All the genes shown within the Venn diagrams in **B** and **D** are all significantly affected genes (FC > 1.5 and *p* < 0.05). The direction of effect on a gene was determined by the symbols (+/−) of the mean FC for the two IFNs and are shown as similarly or oppositely directed arrows next to the number of genes so affected by the two IFNs. If the IFN shows a significant effect on a gene vs. NT, then it is shown in red, if not then is shown in blue. The significant or insignificant effect shown as red or blue for an IFN applies to all genes within the oval irrespective of effect direction. **E** Schematic that explains our strategy used to define perturbed and unperturbed genes by IFN-λ4 shown in **B** and **D**. IFN-λ4 (or IFN-λ3) could affect a gene strongly, moderately, weakly, or not at all as shown in the left side *y*-axis, defined by a hypothetical expression range as shown in the right-side *y*-axis. When the gene expression level falls within the “0” range, then that gene is indeed “unperturbed” by the IFN. When the expression level due to either IFN-λ3 or IFN-λ4 falls in the “weak” range, then although the effect is insignificant for the given IFN (vs. NT), it can still qualify as “perturbed” if the same gene is moderately (same direction) or weakly (opposite direction) but not strongly affected by the other IFN. The dashed rectangle shows the case where IFN-λ4 is weakly affecting a gene that is also strongly affected by IFN-λ3 (could be either direction), hence qualify as “unperturbed” by IFN-λ4. Whereas, a weakly affected gene qualifies as “perturbed” by IFN-λ4, if the same gene is moderately affected by IFN-λ3 in the same direction (blue oval) or weakly affected by IFN-λ3 in the reverse direction (green oval). The solid gray rectangle shows the case where a gene is moderately affected by one IFN, but weakly affected by the other in reverse direction which counts as unperturbed by the latter. Any significant (*) or insignificant (ns) comparison is between IFN-λ3/4 vs. NT or IFN-λ3 vs. IFN-λ4 vertically. The red rectangle shows all the genes that are truly perturbed by IFN-λ4. *X* indicates no comparison across. Related results to **B** and **D** are shown in Suppl. Fig. 2.

IFN-λ4 and IFN-λ3 are both expressed in individuals who carry the variant alleles (ΔG at rs368234815, TT/ΔG) that allow the translation of *IFNL4* in to a functional IFN-λ4 protein [3]. We did a comparison between the genes altered by IFN-λ4 and IFN-λ3 in M1-MDMs (Fig. 3A). A cursory look showed that an additional 68 genes were significantly altered for expression in this analysis in comparison to the number of genes altered by IFN-λ3 vs. NT in M1-MDMs (1123 genes shown in Fig. 1A vs. 1191 genes in Fig. 3A) while a decrease of 373 significantly affected genes was seen in M2-MDMs (2322 genes shown in Fig. 1A vs. 1949 genes in Fig. 3C). However, Venn diagram analysis (Fig. 3B, D) revealed that there could be more genes perturbed by IFN-λ4 and that several of them could be affected in reverse directions by IFN-λ3 and IFN-λ4 (Suppl. Fig. 2). We found that there were 624 additional genes in M1-MDMs and 235 additional genes in M2-MDMs that were significantly affected when IFN-λ3 affected genes were compared against IFN-λ4 affected genes instead of NT (unique genes within green circles in IFN-λ3 vs IFN-λ4 when compared against unique genes within blue circles in IFN-λ3 vs. NT, Fig. 3B, D). Out of the 624 genes in M1-MDMs, 521 genes, and out of the 235 genes in M2-MDMs, 232 genes are novel and were not identified as significant in either IFN-λ3 vs. NT or IFN-λ4 vs. NT. This would suggest that IFN-λ3 and IFN-λ4 were perturbing some genes, likely at insignificant levels (FC < 1.5 and *p* > 0.05), such that these were not revealed in the comparison set of IFN-λ3/4 vs. NT but were revealed as significant genes only when the comparison was between IFN-λ3 vs. IFN-λ4. We tried to focus on such genes affected by IFN-λ4 in the context that it is affecting lesser genes significantly than IFN-λ3 (Fig. 1); the schematic in Fig. 3E helps to understand this better. For example, if a gene is affected by IFN-λ4 sufficiently (i.e., weakly but more than NT, Fig. 3E) and IFN-λ3 affects the same gene strongly in the same direction, then the gene can be considered as unperturbed by IFN-λ4; however, if IFN-λ3 affects the gene either moderately in the same or weakly in the reverse direction to IFN-λ4, then the gene can be considered as sufficiently perturbed by IFN-λ4 (Fig. 3E). This would imply that all those genes absent from the solid triangles shown in Fig. 3B, D are perturbed by IFN-λ4; similarly, all genes unperturbed by IFN-λ3 are within the dashed triangles in Fig. 3B, D. We examined the gene expression data of all the subsets and compared the mean FC expression changes induced by IFN-λ3 and IFN-λ4 to group them as those affected in similar or opposite directions and are shown in Fig. 3B, D.

A large subset of 521 genes (subset within green circle, Fig. 3B) were reciprocally regulated by IFN-λ3 and IFN-λ4 (same as green oval in the schematic Fig. 3E). In this subset, 290 genes were downregulated by IFN-λ3 but upregulated by IFN-λ4 in relation to NT; another 231 were upregulated by IFN-λ3 while they were all downregulated by IFN-λ4 in relation to NT (data not shown). Interestingly, 103 genes (overlapping subset of genes between green and orange circles, Fig. 3B) are strongly affected by IFN-λ4 but are unperturbed by IFN-λ3; more interestingly all the 103 genes were affected by IFN-λ3 in opposite direction to that of IFN-λ4. An additional nine genes were also reciprocally regulated between IFN-λ3 vs. NT and IFN-λ4 vs. NT, but the effect was significant in both sets (Fig. 3B and Suppl. Fig. 2). Therefore, our analysis suggested that there could be at least 530 genes (9 + 521) that may be reciprocally regulated by IFN-λ3 and IFN-λ4 in M1-MDMs. A large subset of 433 genes (subset within blue circle, Fig. 3B) are also weakly but sufficiently perturbed by IFN-λ4 in the same direction to that of IFN-λ3 which has a moderate but significant effect on these genes (same as blue oval in the schematic in Fig. 3E). Similarly, a subset of 126 genes (subset within orange circle, Fig. 3B) are sufficiently perturbed by IFN-λ3 in the same direction to that of IFN-λ4, but the effect of IFN-λ4 is significant even though moderate in comparison to a weak and insignificant effect of IFN-λ3 (refer Fig. 3E for an explanation of what constitutes strong, moderate and a weak effect on gene expression by IFN-λ3/4).

In M2-MDMs, many genes (1707, solid triangle Fig. 3D) remained unperturbed by IFN-λ4 consistent with our results from Fig. 2. However, there were also many genes (230 genes, subset within the green circle in Fig. 3D; all of them are novel significantly affected genes but insignificant in either IFN-λ3 vs. NT or IFN-λ4 vs. NT) that were driven in opposite directions by IFN-λ3 and IFN-λ4 (same as green oval in the schematic Fig. 3E). Moreover, an even larger number of genes (587 genes, subset within blue circle in Fig. 3D) were sufficiently perturbed by IFN-λ4 in the same direction as that of IFN-λ3 (same as blue oval in Fig. 3E). It is clear from this data that IFN-λ4 seems to have a higher threshold for signaling in M2-MDMs than M1-MDMs, given that so many genes are perturbed insignificantly and only 39 genes (all genes within orange set, Fig. 3D) are affected significantly.

In summary, we found a total of 761 genes in both M1 and M2-MDMs that were reciprocally regulated by IFN-λ3 and IFN-λ4 (521 + 9 in Fig. 3B, 230 + 1 in 3D). In addition, many genes appear to be affected in similar directions by the two IFNs; most interestingly a subset of 136 genes (126 in M1 and ten in M2-MDMs; Fig. 3B, D) are affected in similar directions by both IFN-λ3 and IFN-λ4, but the effect is significant only for IFN-λ4. These 136 genes along with 106 other genes (103 in M1 and three in M2-MDMs; Fig. 3B, D), i.e., a total of 242 genes represent the group of genes that are uniquely affected by IFN-λ4 but not by IFN-λ3. In conclusion, our results point towards a large set of genes that could be important in understanding the differential effect of IFN-λ3 and IFN-λ4 in macrophage functions. While the perturbation of genes by both IFN-λ3 and IFN-λ4 shown in our experiments are a direct result of LPS stimulation, and that these same set of genes may not be affected solely by IFN-λs without LPS or with other PAMP (pathogen associated molecular pattern) activation, our results are important to show that the effects of IFN-λ3 and IFN-λ4 could be both same and different in differentiating MDMs.

### Modeling the interactions of IFN-λ3 and IFN-λ4 with the IFN-λ receptor

**Fig. 4:**
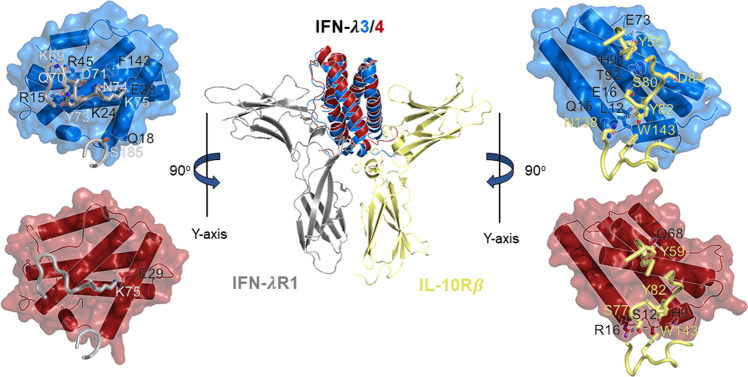
Predicted structural differences of IFN-λ3/4 receptor interactions. Structure of IFN-λ3 (blue, PDB ID 3HHC) structurally aligned to the model of IFN-λ4 (red) are shown as cartoons in context of the receptor complex (middle). IFN-λ4 (red) is predicted to form fewer polar contacts with loops of IFN-λR1 (left, gray) and IL-10Rβ (right, gold) as compared to IFN-λ3 (blue). Residues on the IFNs are labeled in black.

Differences in receptor engagements between IFN-λ3 and IFN-λ4 may also contribute to the differences in gene expression induced by the two IFN-λs observed in this study. Using molecular modeling techniques, we compared the predicted polar contacts between the IFNs and both of their receptor subunits: IFN-λR1 and IL-10Rβ (Fig. 4). IFN-λ3 is predicted to form, in total, fourteen polar contacts in the complex, while IFN-λ4 is predicted to form six polar contacts. There are eight polar contacts predicted for IFN-λ3: IFN-λR1, seven of which occur between the L5 loop of IFN-λR1 [32] and multiple helices of IFN-λ3. Of these seven contacts, five are forming hydrogen bonds, and two form salt bridges. In contrast, there is only one predicted polar contact, a salt bridge, between IFN-λ4 and IFN-λR1 (Glu29 of IFN-λ4 and Lys75 of IFN-λR1). This contact is seen with IFN-λ3: IFN-λR1 (Glu28 of IFN-λ3 and Lys75 of IFN-λR1) and has a similar position on both ligands, indicating that this could be a shared interaction between the different IFNs. IL-10Rβ has six hydrogen bonds with IFN-λ3 and five hydrogen bonds with IFN-λ4 (Tyr82 of IL-10Rβ forms two contacts with IFN-λ4) (Fig. 4); the residues on IL-10Rβ which are identified as important for both proteins are located on loops 2, 3, and 5 of the protein and have been previously identified as important for IFN-λ recognition and binding (Tyr59, Tyr82, and Trp143) [29]. Additional hydrogen bond-forming residues are located on these loops but differ slightly in their placement and involvement.

With the predicted polar interactions being similar between IFN-λ3 and IFN-λ4 with IL-10Rβ, these results suggest that the differences seen in gene expression and macrophage profile could be explained by differences in interaction with IFN-λR1. Although binding to both receptors is necessary for signal transduction, fewer contacts with the high-affinity receptor IFN-λR1 that may impact cooperative binding of IL-10Rβ, combined with the differences in receptor expression in the two cell-types could contribute to the differential signaling profile observed.

### Gene expression changes induced by IFN-λ3 or IFN-λ4 in differentiating MDMs give rise to distinct molecular phenotypes that are relevant in several human diseases

The top 20 genes affected by IFN-λ3 or IFN-λ4 in LPS-stimulated M1 and M2-MDMs are shown in Table 1 (for M1 MDM) and Table 2 (for M2 MDM). The top 100 genes (50 upregulated and 50 downregulated) for each of the comparison sets in M1 and M2-MDMs are listed in Suppl. Tables 1–6 along with information from published literature about their relevance in different human diseases.

**Table 1:** Top up and downregulated genes in M1 MDMs.

Top 20 genes affected by IFN-λ3 (IFN-λ3 vs. NT; M1-MDM)					
Upregulated					
S.No	Gene symbol	Name	Fold change	*P* Value	FDR
1.	CD207	CD207 molecule; langerin	27.55313	0.00016	0.006733
2.	ATP1B2	ATPase; Na+/K+ transporting; beta 2 polypeptide	21.18599	0.0000153	0.001273
3.	ENHO	Energy homeostasis associated	15.62919	0.000236	0.008859
4.	CLEC4F	C-type lectin domain family 4; member F	14.36026	0.001233	0.028224
5.	CD1E	CD1e molecule	13.0977	0.000108	0.005142
6.	SLC51B	Solute carrier family 51; beta subunit	10.48144	0.00039	0.012569
7.	GTF2H2C	GTF2H2 family member C	9.145261	0.017436	0.143113
8.	ITGA11	Integrin; alpha 11	7.645454	0.000123	0.005671
9.	TINAGL1	Tubulointerstitial nephritis antigen-like 1	6.378747	0.004273	0.060536
10.	CYP4F22	cytochrome P450; family 4; subfamily F; polypeptide 22	6.204716	0.004925	0.066438
Downregulated:					
1.	CEACAM8	Carcinoembryonic antigen-related cell adhesion molecule 8	−138.856	1.74E−06	0.000274
2.	SERPINB2	Serpin peptidase inhibitor; clade B (ovalbumin); member 2	−45.5896	5.48E−08	0.0000181
3.	ACKR3	Atypical chemokine receptor 3	−38.6404	2.46E−09	1.62E−06
4.	DNER	Delta/notch-like EGF repeat containing	−24.3093	0.0000745	0.003928
5.	SERPINB7	Serpin peptidase inhibitor; clade B (ovalbumin); member 7	−22.0323	2.14E−06	0.000309
6.	LRRC38	Leucine rich repeat containing 38	−19.4235	0.001343	0.029566
7.	OSMR	Oncostatin M receptor	−18.429	4.3E−08	0.0000151
8.	PLOD2	Procollagen-lysine; 2-oxoglutarate 5-dioxygenase 2	−16.2227	3.71E−16	1.56E−12
9.	RNASE2	Ribonuclease; RNase A family; 2 (liver; eosinophil-derived neurotoxin)	−15.8116	2.34E−15	7.36E−12
10.	SLC8A3	Solute carrier family 8 (sodium/calcium exchanger); member 3	−14.6559	1.97E−06	0.000295

Top 20 genes affected by IFN-λ4 (IFN-λ4 vs. NT; M1-MDM)					
Upregulated					
S.No	Gene symbol	Name	Fold change	*P* value	FDR
1.	JMJD7-PLA2G4B	JMJD7-PLA2G4B readthrough	153.8192	0.000231	0.10347
2.	EPHA7	EPH receptor A7	6.091567	0.000625	0.1784
3.	AMOTL2	Angiomotin like 2	5.162788	0.000143	0.078194
4.	STEAP4	STEAP family member 4	5.041265	9.85E-06	0.015477
5.	HRASLS2	HRAS-like suppressor 2	4.929572	0.00425	0.382293
6.	CHRNA1	Cholinergic receptor; nicotinic; alpha 1 (muscle)	4.002784	0.001796	0.290233
7.	NGFR	Nerve growth factor receptor	3.996891	0.008515	0.461936
8.	CCL19	Chemokine (C–C motif) ligand 19	3.919622	0.002292	0.304988
9.	AUTS2	Autism susceptibility candidate 2	3.737385	0.000106	0.069972
10.	CXCL11	chemokine (C–X–C motif) ligand 11	3.559506	0.003024	0.335556
Downregulated:					
1.	EEF1E1-BLOC1S5	EEF1E1-BLOC1S5 readthrough (NMD candidate)	−819.53	4.42E−07	0.00555
2.	RPS10-NUDT3	RPS10-NUDT3 readthrough	−01.102	0.00231	0.304988
3.	FABP4	Fatty acid binding protein 4; adipocyte	−20.4982	0.000384	0.155867
4.	SERPINB2	Serpin peptidase inhibitor; clade B (ovalbumin); member 2	−19.2608	7.82E−06	0.014033
5.	CEACAM8	Carcinoembryonic antigen-related cell adhesion molecule 8	−8.93941	0.006643	0.434797
6.	BEX2	Brain expressed X-linked 2	−7.61027	0.000304	0.127407
7.	AKR1C1	Aldo-keto reductase family 1; member C1	−6.73425	0.00021	0.097575
8.	ANKRD1	Ankyrin repeat domain 1 (cardiac muscle)	−5.96424	0.002779	0.330014
9.	CXCL6	chemokine (C–X–C motif) ligand 6	−5.87435	0.008196	0.459985
10.	TUBB4A	tubulin; beta 4A class IVa	−5.26986	0.029523	0.629087

Top 20 genes affected in IFN-λ4 vs. IFN-λ3 (M1-MDM)					
Upregulated					
S. no	Gene symbol	Name	Fold change	*P* value	FDR
1.	AMOTL2	Angiomotin like 2	23.94947	1.86E−10	2.92E−07
2.	CTGF	Connective tissue growth factor	21.60929	2.51E−06	0.000524
3.	CXCL13	Chemokine (C–X–C motif) ligand 13	20.62044	1.71E−08	0.0000113
4.	LIFR	Leukemia inhibitory factor receptor alpha	15.74502	5.88E−08	0.0000321
5.	SLC8A3 (NCX-3)	Solute carrier family 8 (sodium/calcium exchanger); member 3	14.49298	2.14E−06	0.000463
6.	ACKR3 (CXCR7)	Atypical chemokine receptor 3	13.79251	5.46E−06	0.000945
7.	LBP	Lipopolysaccharide binding protein	12.88769	0.0000475	0.00439
8.	FOLR2	Folate receptor 2 (fetal)	10.52442	6.28E−07	0.000193
9.	CXCL12	Chemokine (C–X–C motif) ligand 12	10.47408	0.000694	0.022375
10.	DLL4	Delta-like 4 (Drosophila)	10.41865	0.005953	0.076214
Downregulated:					
1.	EEF1E1-BLOC1S5	EEF1E1-BLOC1S5 readthrough (NMD candidate)	−702.578	6.85E−07	0.000196
2.	RPS10-NUDT3	RPS10-NUDT3 readthrough	−155.093	0.004791	0.067953
3.	TBC1D3H	TBC1 domain family; member 3H	−109.147	0.005198	0.071109
4.	ATP1B2	ATPase; Na+/K+ transporting; beta 2 polypeptide	−13.4416	0.000143	0.008274
5.	GTF2H2C	GTF2H2 family member C	−12.227	0.008495	0.09482
6.	CPNE6	Copine VI (neuronal)	−7.51288	0.001389	0.032584
7.	CD1E	CD1e molecule	−6.44149	0.002925	0.050776
8.	CCL24	Chemokine (C–C motif) ligand 24	−5.93285	1.93E−08	0.0000121
9.	CCL1	Chemokine (C–C motif) ligand 1	−5.68683	7.85E−09	6.17E−06
10.	GPR35	G protein-coupled receptor 35	−5.64183	0.000315	0.01384

**Table 2:** Top up and downregulated genes in M2 MDMs.

Top 20 genes affected by IFN-λ3 (IFN-λ3 vs. NT; M2-MDM)					
Upregulated:					
S.No	Gene symbol	Name	Fold change	*P* Value	FDR
1.	PPBP	Pro-platelet basic protein (chemokine (C–X–C motif) ligand 7)	280.9127	3.3E−17	5.92E−14
2.	GTF2H2C	GTF2H2 family member C	133.659	7.25E−05	0.000982
3.	RGS18	Regulator of G-protein signaling 18	47.45056	1.27E−10	2.34E−08
4.	SPATA12	Spermatogenesis associated 12	30.027	1.98E−07	0.0000091
5.	ALK	Anaplastic lymphoma receptor tyrosine kinase	26.89163	0.000575	0.004879
6.	TMEM37	Transmembrane protein 37	22.69274	4.84E−10	6.99E−08
7.	F13A1	Coagulation factor XIII; A1 polypeptide	15.9446	1.95E−06	0.000057
8.	ASIC1	Acid sensing (proton gated) ion channel 1	15.75164	4.28E−05	0.000641
9.	CXCL5	Chemokine (C–X–C motif) ligand 5	15.66281	7.38E−14	3.76E−11
10.	FAXDC2	Fatty acid hydroxylase domain containing 2	13.9745	7.75E−12	2.32E−09
Downregulated:					
1.	H3F3A	H3 histone; family 3A	−747.187	0.002392	0.01467
2.	CCL19	Chemokine (C–C motif) ligand 19	−48.3947	1.58E−13	7.35E−11
3.	KIAA1644	KIAA1644	−32.2811	0.000138	0.001611
4.	FCAMR	Fc receptor; IgA; IgM; high affinity	−28.1209	9.66E−09	0.000000724
5.	CAMK2A	calcium/calmodulin-dependent protein kinase II alpha	−26.0324	4.4E−06	0.000109
6.	EBF4	Early B-cell factor 4	−21.6916	1.58E−07	0.00000749
7.	SLC8A3	Solute carrier family 8 (sodium/calcium exchanger); member 3	−19.9602	1.34E−08	0.000000921
8.	MMP10	Matrix metallopeptidase 10	−19.4389	3.83E−12	1.3E−09
9.	ADAM19	ADAM metallopeptidase domain 19	−16.5674	3.63E−09	0.000000334
10.	GJA4	Gap junction protein; alpha 4; 37 kDa	−16.0479	0.000454	0.004066

Top 20 genes affected by IFN-λ4 (IFN-λ4 vs. NT; M2-MDM)					
Upregulated:					
S.No	Gene symbol	Name	Fold change	*P* value	FDR
1.	CLEC5A	C-type lectin domain family 5; member A	3.410393	0.007795	1
2.	ARAP3	ArfGAP with RhoGAP domain; ankyrin repeat and PH domain 3	2.451104	0.009496	1
3.	PCDHGA11	protocadherin gamma subfamily A; 11	2.434315	0.047776	1
4.	IL1R2	Interleukin 1 receptor; type II	2.218644	0.033769	1
5.	SDC2	Syndecan 2	2.061122	0.005596	1
7.	PDGFRA	Platelet-derived growth factor receptor; alpha polypeptide	1.993987	0.003407	1
8.	CXCL8	Chemokine (C–X–C motif) ligand 8	1.951685	0.033416	1
9.	TRIB2	Tribbles pseudokinase 2	1.910104	0.030882	1
10.	NOTCH3	Notch 3	1.860591	0.010832	1
Downregulated:					
1.	RGPD8	RANBP2-like and GRIP domain containing 8	−757.257	0.001271	1
2.	EEF1E1-BLOC1S5	EEF1E1-BLOC1S5 readthrough (NMD candidate)	−231.847	1.54E−05	0.096851
3.	LY75-CD302	LY75-CD302 readthrough	−145.615	3.46E−05	0.144833
4.	SNORD3A	Small nucleolar RNA; C/D box 3A	−3.35462	0.001409	1
5.	LBP	Lipopolysaccharide binding protein	−3.08656	0.032509	1
6.	FCAMR	Fc receptor; IgA; IgM; high affinity	−2.87411	0.034976	1
7.	GPX8	Glutathione peroxidase 8 (putative)	−2.57192	0.015076	1
8.	ALDH1A2	Aldehyde dehydrogenase 1 family; member A2	−2.12572	0.017627	1
9.	ANKRD22	Ankyrin repeat domain 22	−1.91806	0.002439	1
10.	FAM134B	Family with sequence similarity 134; member B	−1.86998	0.022027	1

Top 20 genes affected by IFN-λ4 vs IFN-λ3; M2-MDM)					
Upregulated:					
S.No	Gene symbol	Name	Fold change	*P* value	FDR
1.	KIAA1644	KIAA1644	42.57416	4.67E−05	0.001079
2.	CCL19	Chemokine (C–C motif) ligand 19	28.38406	4.08E−11	1.56E−08
3.	MMP10	Matrix metallopeptidase 10	15.67925	8.99E−11	3.14E−08
4.	IRF4	Interferon regulatory factor 4	15.59398	1.45E−11	6.3E−09
5.	ADAM19	ADAM metallopeptidase domain 19	14.48353	1.44E−08	0.00000181
6.	GJA4	Gap junction protein; alpha 4; 37 kDa	12.92545	0.001052	0.010261
7.	NR4A3	Nuclear receptor subfamily 4; group A; member 3	12.44311	1.04E−08	0.0000014
8.	EDN1	Endothelin 1	12.05188	7.98E−16	1.55E−12
9.	SLC8A3 (NCX-3)	Solute carrier family 8 (sodium/calcium exchanger); member 3	11.72561	1.71E−06	0.0000804
10.	NTN1	Netrin 1	11.67039	8.54E−07	0.0000462
Downregulated:					
1.	LY75-CD302	LY75-CD302 readthrough	−989.265	9.65E−08	0.00000842
2.	PPBP (CTAPIII)	Pro-platelet basic protein (chemokine (C–X–C motif) ligand 7)	−272.27	4.1E−17	1.03E−13
3.	CA12	Carbonic anhydrase XII	−17.4539	1.03E−08	0.00000139
4.	F13A1	Coagulation factor XIII; A1 polypeptide	−17.0878	1.12E−06	0.0000576
5.	PVALB	Parvalbumin	−16.8867	0.000833	0.00866
6.	SPATA12	Spermatogenesis associated 12	−14.047	6.36E−06	0.000238
7.	CCL13	Chemokine (C–C motif) ligand 13	−14.0324	1.57E−09	0.000000329
8.	ALK	Anaplastic lymphoma receptor tyrosine kinase	−13.6253	0.00311	0.022872
9.	EDNRB	Endothelin receptor type B	−12.8236	0.000898	0.0091
10.	TMEM37	Transmembrane protein 37	−12.0911	1.19E−07	0.0000097

Some of the important perturbations in M1-MDMs were a 27-fold upregulation of the *CD207* and a 138-fold downregulation of *CEACAM8* by IFN-λ3; a 153-fold upregulation of *JMJD7-PLA2G4B* and an 819-fold (700-fold in IFN-λ4 vs. IFN-λ3) and 300-fold (155-fold in IFN-λ4 vs. IFN-λ3) downregulation of *EEF1E1-BLOC1S5*, *RPS10-NUDT3* respectively by IFN-λ4; and a 24-fold upregulation of *AMOTL2* and a 109-fold downregulation of *TBC1D3H* by IFN-λ4 vs. IFN-λ3. In M2-MDMs, 281-fold and a 133-fold upregulation of *PPBP (CXCL7)* and *GTF2H2C* respectively, a 747-fold downregulation of *H3F3A* was seen with IFN-λ3; a 28 and 15-fold upregulation of *CCL19* and *MMP10* respectively, a 989-fold and 272-fold downregulation of *LY75-CD302* and *PPBP (CTAPIII)* respectively was seen with IFN-λ4 vs. IFN-λ3. Clearly, some of the genes are very strongly affected by the two IFN-λs and since they are all associated with human diseases (Suppl. Tables 1–6), further research is warranted in these areas.

**Fig. 5:**
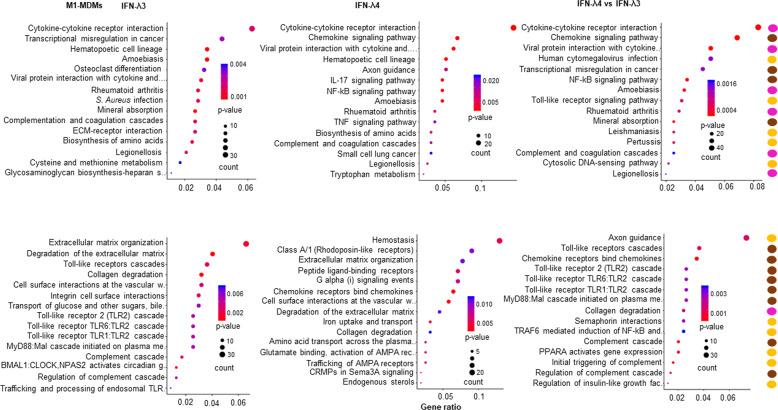
M1-MDMs differentiated in presence of IFN-λ3 and IFN-λ4 show distinct molecular phenotypes. The top panel shows analysis from KEGG pathways, and the bottom panel shows results from Reactome analysis. The top 15 pathways are only shown, all the affected pathways are shown in Suppl. Data 1. The buttons in different colors to the right indicate whether the pathway was affected either in IFN-λ3 vs. NT or IFN-λ4 vs. NT (brown) or both (pink) or is unique (yellow) to IFN-λ3 vs. IFN-λ4.

**Fig. 6:**
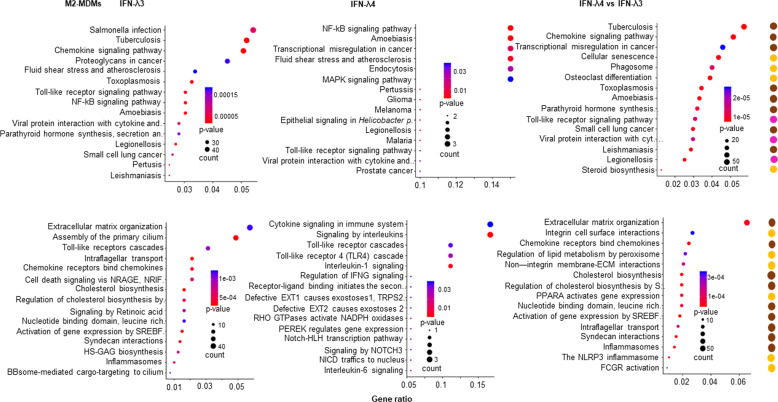
M2-MDMs differentiated in presence of IFN-λ3 and IFN-λ4 show distinct molecular phenotypes. The top panel shows analysis from KEGG pathways, and the bottom panel shows results from Reactome analysis. The top 15 pathways are only shown, all the affected pathways are shown in Suppl. Data 2. The buttons in different colors to the right indicate whether the pathway was affected either in IFN-λ3 vs. NT or IFN-λ4 vs. NT (brown) or both (pink) or is unique (yellow) to IFN-λ3 vs. IFN-λ4.

We performed GO enrichment and pathway analysis (using KEGG and Reactome databases) using the gene expression data. All three comparison sets (IFN-λ3 vs. NT, IFN-λ4 vs. NT and IFN-λ4 vs. IFN-λ3) from M1-MDMs and M2-MDMs were included in the GO enrichment and pathway analysis. The results of GO enrichment analysis for M1-MDMs are shown as Suppl. Fig. 3A–F. The results of top fifteen significantly affected KEGG and Reactome pathways in each comparison set for M1-MDMs and M2-MDMs are presented in Fig. 5 and Fig. 6, respectively; the details of all the affected pathways are shown as Suppl. Data 1 (M1-MDM) and 2 (M2-MDM). Several important disease-related pathways were affected in all three comparison sets when interrogated against the KEGG pathway database (Fig. 5, upper panel). Amoebiasis, rheumatoid arthritis, *S. aureus* infection and legionellosis were some of the top pathways affected in M1-MDMs differentiated in presence of IFN-λ3; amoebiasis, rheumatoid arthritis, small cell lung cancer, and legionellosis were affected by IFN-λ4 in M1-MDMs, while amoebiasis, rheumatoid arthritis, and legionellosis were also affected pathways when IFN-λ4 vs. IFN-λ3 gene sets were analyzed. Interestingly, five new pathways that did not show up either in IFN-λ3 vs. NT or IFN-λ4 vs. NT pathway analysis were significantly affected when the comparison was made between IFN-λ4 vs. IFN-λ3; these novel pathways were involved in some important human diseases like human cytomegalovirus (CMV) infection, pertussis and leishmaniasis (Fig. 5). It is known that IFN-λ gene polymorphisms are associated with CMV infections in humans [33] and recent reports suggest type III IFNs are important for inflammatory responses in *Bordetella pertussis* infection in mice [34]. Other than the disease-related pathways, several other critical pathways involved in physiology and pathology like cytokine-cytokine receptor interaction, transcriptional misregulation in cancer, viral protein interaction with cytokine and cytokine receptor, NF-kB signaling pathway, Toll-like receptor (TLR) signaling pathway and others were significantly affected in one or two of the three comparison sets (Fig. 5, upper panel). Reactome pathway analysis in M1-MDMs revealed involvement of extracellular matrix (ECM) organization, TLR pathways among others (Fig. 5, lower panel). Collagen degradation was the only pathway that was affected both independently in IFN-λ3 and IFN-λ4 treated M1-MDMs and in IFN-λ4 vs. IFN-λ3 comparison set.

Pathway analysis in M2-MDMs showed that IFN-λ3 treatment on differentiating macrophages had led to differential expression of genes that were important in several infectious diseases including tuberculosis, salmonellosis, toxoplasmosis, amoebiasis, legionellosis, pertussis, and leishmaniasis (Fig. 6, upper panel). IFN-λ4, even though had affected only a small number of genes significantly (Figs. 1 and 2), showed that it had affected important disease pathways in M2-MDMs. Apart from infectious diseases like amoebiasis, pertussis, legionellosis and malaria, IFN-λ4 had affected some cancer pathways including glioma, melanoma, and prostate cancer. IFN-λ4 is known to be associated with prostate cancer [35, 36]. When genes affected in IFN-λ4 vs. IFN-λ3 were probed, tuberculosis was the most significantly affected pathway in M2-MDMs, among other infectious diseases. Pathways like cellular senescence, phagosome, osteoclast differentiation and steroid biosynthesis showed up in genes affected only when the comparison was between IFN-λ4 vs. IFN-λ3. Reactome pathways analysis showed six pathways becoming significant (that did not show up either with IFN-λ4 or IFN-λ3, in relation to NT) when the comparison was between IFN-λ4 vs. IFN-λ3 in M1 and M2-MDMs. ECM organization was the top-most pathway affected in the Reactome analysis in the IFN-λ4 vs. IFN-λ3 gene set from M2-MDMs. In summary, several important pathways involving both human pathology and physiology are significantly affected in IFN-λ3/4 treated LPS-stimulated MDMs leading to some unique molecular phenotypes.

## Discussion

Previous transcriptomics studies [30, 31], where RNA-seq was performed soon after IFN-λ treatment in primary human hepatocytes or human airway epithelial cells [30] or Huh7 [31] cells have shown that IFN-λ3 and IFN-λ4 affect almost similar set of genes. A very recent study in HepG2 cells that expressed intracellular IFN-λ3/4 does show hugely different DEGs affected by IFN-λ3 and IFN-λ4 [37]. However, all the above studies have reported mostly ISGs as the principally affected genes by either IFN-λ3/4 recombinant protein treatment [30, 31] or due to their intracellular overexpression [37] because RNA-seq was performed immediately following IFN-λ3/4 treatment for various time intervals in these studies [30, 31, 37]. Our experimental design is different from the above studies in that, we are looking for an effect of IFN-λ3/4 on the monocyte-to-macrophage differentiation pathway since the IFN-λs were added into the differentiation medium, allowing them to influence the developing macrophage phenotypes over a period of 6 days. Moreover, no IFN-λs were present for 24 h in the medium when the MDMs were being stimulated, after which the cells were subject to RNA-seq (Fig. 1A, schematic). Therefore, the DEGs within IFN-λ-treated MDMs in our experiments, even though directly arise from LPS signaling during stimulation, are expected to reflect a more complex phenomenon resulting from sequential interactions between ISG signatures induced by IFN-λ3/4 and macrophage development pathways. Moreover, this protocol tries to mimic the in vivo situation wherein immune cells are developing in a milieu of various chemokines, growth factors and cytokines including IFNs along with different microbial stimuli like LPS. Further, as LPS treatment was given to all cells (Fig. 1, schematic), the DEGs analyzed will reflect only changes arising from the respective IFN treatment since the effect of LPS would cancel out in the comparison sets.

One of the important findings we make in this study is that IFN-λ4 can influence gene expression that is both similar and unique to that of IFN-λ3 (Figs. 1, 2, and 3 and Suppl. Fig. 2). A cursory analysis of the unique and common genes induced in IFN-λ3 vs. NT and IFN-λ4 vs. NT showed 254 (125 + 115 in M1-MDMs; 8 + 6 in M2-MDMs; Fig. 2B) significantly affected genes that are unique to IFN-λ4 (Fig. 2B). However, after more detailed analysis, especially after utilizing the gene sets from IFN-λ3 vs. IFN-λ4 comparison, we obtain a clearer picture of the genes significantly and insignificantly affected by IFN-λ4 in similar or opposite directions to that affected by IFN-λ3. However, genes affected significantly by IFN-λ4 are far lesser when compared to those affected significantly by IFN-λ3 (Fig. 3). We have also used a very high concentration of IFN-λ4 (6 µg/ml) compared to IFN-λ3 (0.05 µg/ml) [22] as we found that these concentrations of the proteins were found to give comparable level of ISG stimulation in different cell types as detailed in our earlier study [22]. This is due to the low specific activity of the recombinant IFN-λ4 protein used [22], which is a limitation of our study; further, the different heterologous systems used to obtain IFN-λ3 and IFN-λ4 as recombinant proteins that were used in this study may also have contributed to some differences in the induction of genes. Hence, more studies are needed with a more active protein to identify and confirm the full effect of IFN-λ4 on differentiating MDMs.

Another observation we have made in this study, even though based on the number of genes affected in RNA-seq and not through extensive experimentation, is that IFN-λ4 has preference for M1-MDMs over M2-MDMs while IFN-λ3 has a greater effect on M2-MDMs than M1-MDMs (Figs. 1, 2). If we include all those genes unperturbed by IFN-λ4 (solid triangles in Fig. 3B, D) and compare the same with IFN-λ3 (dashed triangles in Fig. 3B, D), we see that in M1-MDMs there are ~5-fold greater number of genes unperturbed by IFN-λ4 than IFN-λ3, while the number increases to ~570 in M2-MDMs, clearly suggesting that IFN-λ3 and IFN-λ4 have different preferences for M1 and M2-MDMs. This needs further investigation, considering the noise in the experiments due to interindividual variation; for example, a recent study showed that IFN-λ3 induced more genes in M1 than M2-MDMs using primary cells obtained from human volunteers [38]. Therefore, the different preferences of IFN-λ3 and IFN-λ4 for different MDM types may show wide variation depending on the individual background. While we can observe these differences in our study, we are unable to offer a complete explanation for the same. However, we do speculate that the differences could be due, in part, to cells having different thresholds for the two IFN-λs and thus, optimal activity will be cell-type dependent. For. Ex. IFN-λ4 has a higher threshold in M2-MDMs than M1-MDMs, implying that there may be differences in receptor subunit abundance and/or intrinsic differences in the pathways in the two cell types that lead to these threshold changes [39]. To this effect, the structural model is an important starting point in understanding the differences of IFN-λ3 and IFN-λ4 signaling influenced by their interactions with the IFN-λR1 receptor subunit (Fig. 4). More detailed biophysical and structural studies to answer these questions are currently not possible due to the inability to produce milligram quantities of IFN-λ4 protein needed for such studies.

Some of the genes like *EEF1E1-BLOC1S5, RPS10-NUDT3, LY75-CD302 and PPBP (CTAPIII)* have been affected several hundred-fold in their expression by either IFN-λ3 or IFN-λ4 (or both) in M1 or M2-MDMs (Tables 1 and 2 and Suppl. Tables 1–6). These and other genes that are affected significantly and at very high levels, certainly need further studies for confirmation and to gain further insights into the diseases that they are associated with (Suppl. Tables 1–6). GO and KEGG, Reactome pathway analysis, similarly, have thrown up several diseases that may be relevant in the context of macrophage and type III IFN biology that needs further attention (Figs. 5 and 6 and Suppl. Data 1 and 2). The top four pathways, identified in our previous study [22] using KEGG pathway analysis from RNA-seq data obtained in IFN-λ4-treated M1-MDMs (IFN-λ4 vs. NT) derived from an unrelated individual (Suppl. Fig. 4) were all represented within the top six KEGG pathways affected in M1-MDMs in this study (Fig. 5, upper panel), demonstrating the reproducibility of the results from independent experiments carried out on cells derived from unrelated individuals. However, these results should be interpreted in the context of the specific effect of LPS on differentiated MDMs, as IFN-λ3/4 treatment would not give the same set of DEGs shown in this study that were eventually used to perform pathway enrichment analysis. As a recent study has shown, this effect could be different or same for different PAMPs [40].

In summary, we have shown, by using transcriptomics, that human IFN-λ3 and IFN-λ4 that are only ~30% identical to each other, can have significant differences in their functions based on the distinct molecular phenotypes we have identified in LPS-activated M1 and M2 MDMs. It is known that IFN-λ3 and IFN-λ4 have different kinetics of ISG induction [11, 41], and it is possible that even though they may induce similar set of ISGs [30, 31], the differences in the kinetics of ISG induction between IFN-λ3 and IFN-λ4 could have contributed to the different molecular phenotypes of MDMs observed in this study.

## Supplementary information

Supplemental Figures

Supplemental Tables

Supplemental Data 1

Supplemental Data 2

## References

[1] Sheppard P, Kindsvogel W, Xu W, Henderson K, Schlutsmeyer S, Whitmore TE (2003). IL-28, IL-29 and their class II cytokine receptor IL-28R. Nat Immunol.

[2] Kotenko SV, Gallagher G, Baurin VV, Lewis-Antes A, Shen M, Shah NK (2003). IFN-λs mediate antiviral protection through a distinct class II cytokine receptor complex. Nat Immunol.

[3] Prokunina-Olsson L, Muchmore B, Tang W, Pfeiffer RM, Park H, Dickensheets H (2013). A variant upstream of IFNL3 (IL28B) creating a new interferon gene IFNL4 is associated with impaired clearance of hepatitis C virus. Nat Genet.

[4] Manry J, Laval G, Patin E, Fornarino S, Itan Y, Fumagalli M (2011). Evolutionary genetic dissection of human interferons. J Exp Med.

[5] Key FM, Peter B, Dennis MY, Huerta-Sánchez E, Tang W, Prokunina-Olsson L (2014). Selection on a variant associated with improved viral clearance drives local, adaptive pseudogenization of interferon lambda 4 (IFNL4). PLoS Genet.

[6] Gad HH, Dellgren C, Hamming OJ, Vends S, Paludan SR, Hartmann R (2009). Interferon-lambda is functionally an interferon but structurally related to the interleukin-10 family. J Biol Chem.

[7] Roy S, Guha Roy D, Bhushan A, Bharatiya S, Chinnaswamy S (2021). Functional genetic variants of the IFN-λ3 (IL28B) gene and transcription factor interactions on its promoter. Cytokine.

[8] Bhushan A, Ghosh S, Bhattacharjee S, Chinnaswamy S (2017). Confounding by single nucleotide polymorphism rs117648444 (P70S) affects the association of interferon lambda locus variants with response to Interferon-α-ribavirin therapy in patients with chronic genotype 3 hepatitis C virus infection. J Interferon Cytokine Res.

[9] Chinnaswamy S, Kowalski ML (2019). The genetic association of IFN-λs with human inflammatory disorders remains a conundrum. J Interferon Cytokine Res.

[10] Prokunina-Olsson L (2019). Genetics of the human interferon lambda region. J Interferon Cytokine Res.

[11] Obajemu AA, Rao N, Dilley KA, Vargas JM, Sheikh F, Donnelly RP (2017). IFN-λ4 attenuates antiviral responses by enhancing negative regulation of IFN signaling. J Immunol.

[12] Chen Q, Coto-Llerena M, Suslov A, Teixeira RD, Fofana I, Nuciforo S (2021). Interferon lambda 4 impairs hepatitis C viral antigen presentation and attenuates T cell responses. Nat Commun.

[13] Prokunina-Olsson L, Morrison RD, Obajemu A, Mahamar A, Kim S, Attaher O (2021). IFN-λ4 is associated with increased risk and earlier occurrence of several common infections in African children. Genes Immun.

[14] O’Brien TR, Pfeiffer RM, Paquin A, Lang kuhs KA, Chen S, Bonkovsky HL (2015). Comparison of functional variants in IFNL4 and IFNL3 for association with HCV clearance. J Hepatol.

[15] Ansari MA, Aranday-Cortes E, Ip CL, da Silva Filipe A, Lau SH, Bamford C (2019). Interferon lambda 4 impacts the genetic diversity of hepatitis C virus. Elife.

[16] Samayoa-Reyes G, Jackson C, Ogolla S, Sabourin K, Obajemu A, Dent AE (2021). IFN-λ4 genetic variants influence clinical malaria episodes in a cohort of Kenyan children. Malar J.

[17] Onabajo OO, Muchmore B, Prokunina-Olsson L (2019). The IFN-λ4 conundrum: when a good interferon goes bad. J Interferon Cytokine Res.

[18] Hamming OJ, Terczyńska-Dyla E, Vieyres G, Dijkman R, Jørgensen SE, Akhtar H (2013). Interferon lambda 4 signals via the IFNλ receptor to regulate antiviral activity against HCV and coronaviruses. EMBO J.

[19] Hong M, Schwerk J, Lim C, Kell A, Jarret A, Pangallo J (2016). Interferon lambda 4 expression is suppressed by the host during viral infection. J Exp Med.

[20] Zhou H, Møhlenberg M, Terczyńska-Dyla E, Winther KG, Hansen NH, Vad-Nielsen J (2020). The IFNL4 gene is a noncanonical interferon gene with a unique but evolutionarily conserved regulation. J Virol.

[21] Eslam M, Ahlenstiel G, George J (2019). Interferon lambda and liver fibrosis. J Interferon Cytokine Res.

[22] De M, Bhushan A, Chinnaswamy S (2021). Monocytes differentiated into macrophages and dendritic cells in the presence of human IFN-λ3 or IFN-λ4 show distinct phenotypes. J Leukoc Biol.

[23] Langmead B, Salzberg SL (2012). Fast gapped-read alignment with Bowtie 2. Nat Methods.

[24] Li B, Dewey CN (2011). RSEM: accurate transcript quantification from RNA-Seq data with or without a reference genome. BMC Bioinform.

[25] Robinson MD, McCarthy DJ, Smyth GK (2010). edgeR: a Bioconductor package for differential expression analysis of digital gene expression data. Bioinformatics.

[26] Young MD, Wakefield MJ, Smyth GK, Oshlack A (2010). Gene ontology analysis for RNA-seq: accounting for selection bias. Genome Biol.

[27] Xie C, Mao X, Huang J, Ding Y, Wu J, Dong S (2011). KOBAS 2.0: a web server for annotation and identification of enriched pathways and diseases. Nucleic Acids Res.

[28] Källberg M, Wang H, Wang S, Peng J, Wang Z, Lu H (2012). Template-based protein structure modeling using the RaptorX web server. Nat Protoc.

[29] Mendoza JL, Schneider WM, Hoffmann HH, Vercauteren K, Jude KM, Xiong A (2017). The IFN-λ-IFN-λR1-IL-10Rβ complex reveals structural features underlying type III IFN functional plasticity. Immunity.

[30] Lauber C, Vieyres G, Terczyńska-Dyla E, Anggakusuma, Dijkman R, Gad HH (2015). Transcriptome analysis reveals a classical interferon signature induced by IFNλ4 in human primary cells. Genes Immun.

[31] Lunova M, Kubovciak J, Smolková B, Uzhytchak M, Michalova K, Dejneka A (2021). Expression of interferons lambda 3 and 4 induces identical response in human liver cell lines depending exclusively on canonical signaling. Int J Mol Sci.

[32] Miknis ZJ, Magracheva E, Li W, Zdanov A, Kotenko SV, Wlodawer A (2010). Crystal structure of human interferon-λ1 in complex with its high-affinity receptor interferon-λR1. J Mol Biol.

[33] Egli A, Levin A, Santer DM, Joyce M, O’Shea D, Thomas BD (2014). Immunomodulatory function of Interleukin 28B during primary infection with cytomegalovirus. J Infect Dis.

[34] Ardanuy J, Scanlon K, Skerry C, Fuchs SY, Carbonetti NH (2020). Age-dependent effects of type I and type III IFNs in the pathogenesis of bordetella pertussis infection and disease. J Immunol.

[35] Minas TZ, Tang W, Smith CJ, Onabajo OO, Obajemu A, Dorsey TH (2018). IFNL4-ΔG is associated with prostate cancer among men at increased risk of sexually transmitted infections. Commun Biol.

[36] Tang W, Wallace TA, Yi M, Magi-Galluzzi C, Dorsey TH, Onabajo OO (2018). IFNL4-ΔG allele is associated with an interferon signature in tumors and survival of African-American men with prostate cancer. Clin Cancer Res.

[37] Onabajo OO, Wang F, Lee MH, Florez-Vargas O, Obajemu A, Tanikawa C (2021). Intracellular accumulation of IFN-λ4 induces ER stress and results in anti-cirrhotic but pro-HCV effects. Front Immunol.

[38] Read SA, Wijaya R, Ramezani-Moghadam M, Tay E, Schibeci S, Liddle C (2019). Macrophage coordination of the interferon lambda immune response. Front Immunol.

[39] Pervolaraki K, Rastgou Talemi S, Albrecht D, Bormann F, Bamford C, Mendoza JL (2018). Differential induction of interferon stimulated genes between type I and type III interferons is independent of interferon receptor abundance. PLoS Pathog.

[40] Read SA, Gloss BS, Liddle C, George J, Ahlenstiel G (2021). Interferon-λ3 exacerbates the inflammatory response to microbial ligands: implications for SARS-CoV-2 pathogenesis. J Inflamm Res.

[41] Guo C, Reuss D, Coey JD, Sukumar S, Lang B, McLauchlan J (2021). Conserved induction of distinct antiviral signalling kinetics by primate interferon lambda 4 proteins. Front Immunol.

