# Supplementary material for: Distinct molecular phenotypes involving several human diseases are induced by IFN-λ3 and IFN-λ4 in monocyte-derived macrophages
Source: Genes Immun. 2022 Feb 3;23(2):73–84. doi: 10.1038/s41435-022-00164-w (PMC9042695; doi:10.1038/s41435-022-00164-w)

Suppl. Fig. 1

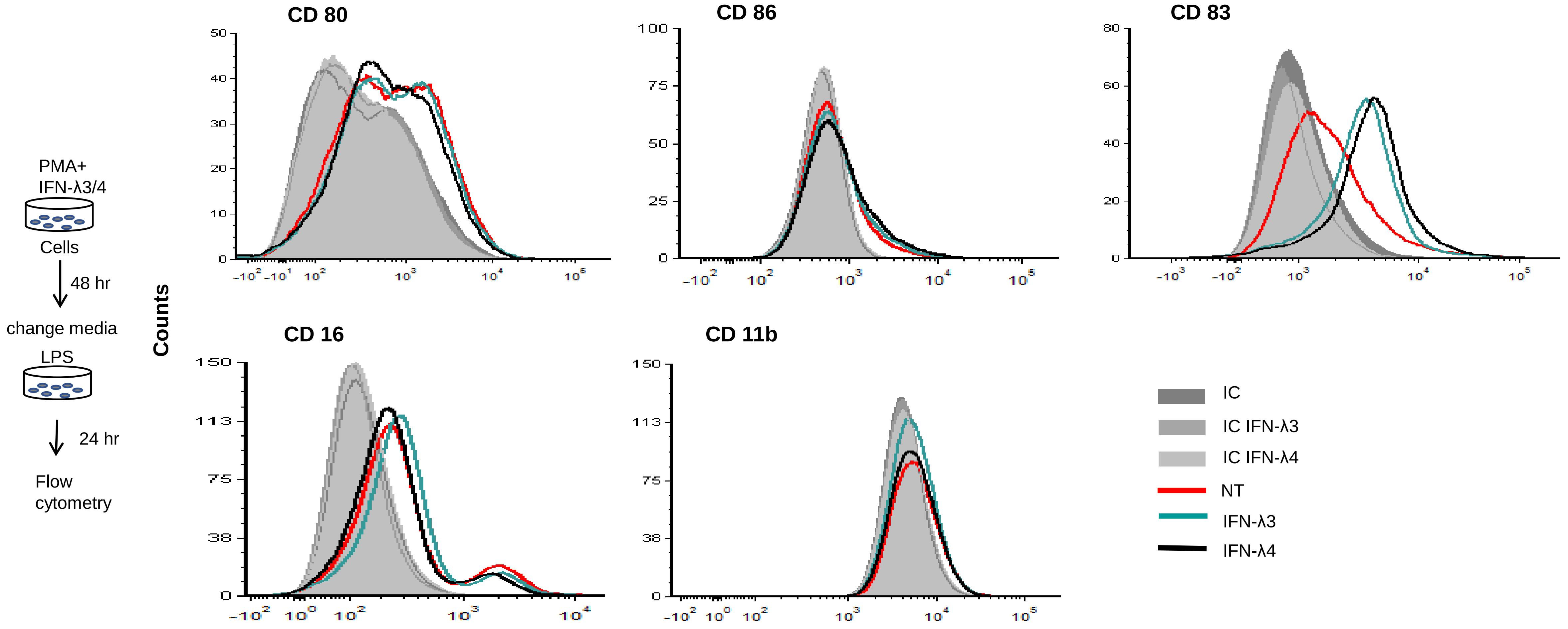

Suppl. Fig. 1: Expression of surface markers CD80, CD83, CD16, CD11b and CD86 was determined by flowcytometry in THP-1-derived macrophage-like cells differentiated in absence (NT) or presence of 50 ng/ml of IFN- λ3 or 6 µg/ml of IFN- λ4 proteins for 48 h (pretreatment strategy, Ref. 22) and then matured with 1 µg/ml LPS for 24 h. IC-isotype control; NT- no treatment. Schematic of the protocol is shown at the left. The surface protein marker expression was assayed in M1 THP-1-derived macrophage-like cells differentiated using the pre-treatment strategy (22). THP-1 cells were differentiated with PMA as before along with IFN-λ4 (6 µg/ml) or IFN-λ3 (50 ng/ml) or no IFNs for 48 h. After maturation with LPS (1 µg/ml) for an additional 24 h, cells were harvested and incubated with human Fc block (BD Biosciences ,San Jose, CA, USA) before staining. The cells were stained with PE Cy7-conjugated anti-human CD80 mAb, APC-conjugated anti-human CD86 mAb, APCH7-conjugated anti-human HLADR mAb, PE-CF594- conjugated CD83 mAb, Alexa-Fluor700-conjugated anti-human CD16 mAb and V-500-conjugated anti-human CD 11b mAb or isotype-matched control mAbs (all from BD Biosciences). The cells were analyzed using BD Aria Fusion cytometer and the data was analyzed with FCS Express 6 (DeNovoSoftware, Pasadena, CA, USA).

Suppl. Fig. 2

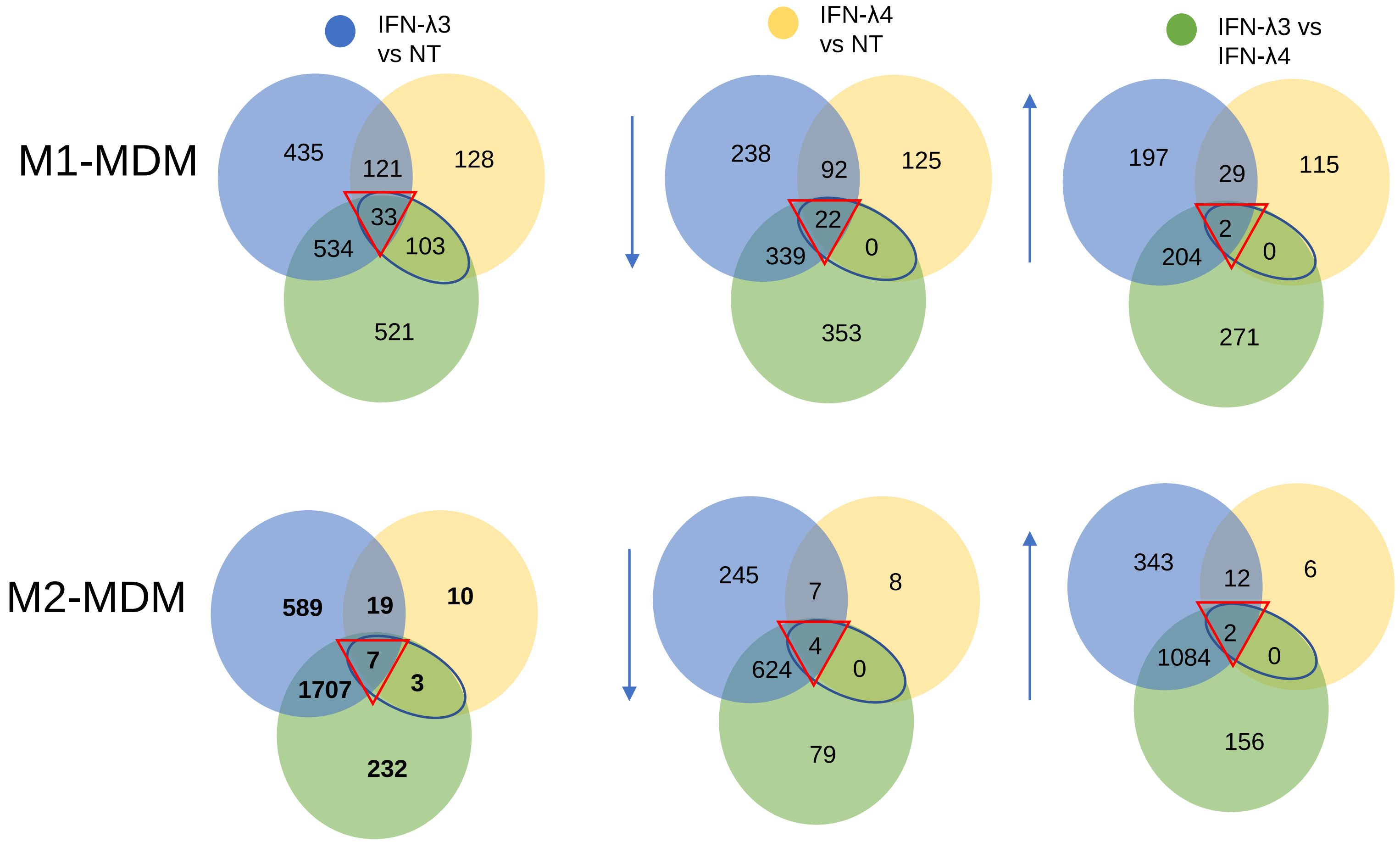

Suppl. Fig. 2: Venn diagram analysis shows that IFN-λ4 may be affecting far a greater number of genes than the significantly affected ones shown in Fig. 1. The numbers show the genes that are significantly affected in the main sets and subsets. Arrows show the subset of genes that are upregulated or downregulated. Blue ellipses show the common genes in the main sets when comparison is made to deduce the reciprocally regulated genes between the sets. Ex. Blue ellipse in the main sets in M1-MDMs shows a subset of 103 genes common to IFN-λ4 vs NT and IFN-λ3 vs IFN-λ4, but they were all reciprocally regulated; hence zero genes are seen in the subsets. Nine genes within the subset of 33 genes shown in the comparison between all three sets (IFN-λ3 vs NT, IFN-λ4 vs NT and IFN-λ3 vs IFN-λ4 shown inside red triangles) were reciprocally regulated, hence are missing in the subsets (only 22 and 2 are seen).

Suppl. Fig. 3A

GO enrichment analysis for IFN-λ3 vs NT M1-MDM.

GO Enrichment Analysis: the main use of the GO is to perform enrichment analysis on gene sets. For example, given a set of genes that are upregulated and /or downregulated under certain conditions, an enrichment analysis will find which GO terms are over-represented (or under-represented) using annotations for that gene set.

$$p = 1 - \sum_{i=0}^{m-1} \frac{\binom{M}{i}\binom{N-M}{n-i}}{\binom{N}{n}}$$

Here N is the number of all genes with GO annotation, n is the number of target gene candidates in N, M is the number of all genes annotated to a certain pathway, and m is the number of target gene candidates in M.

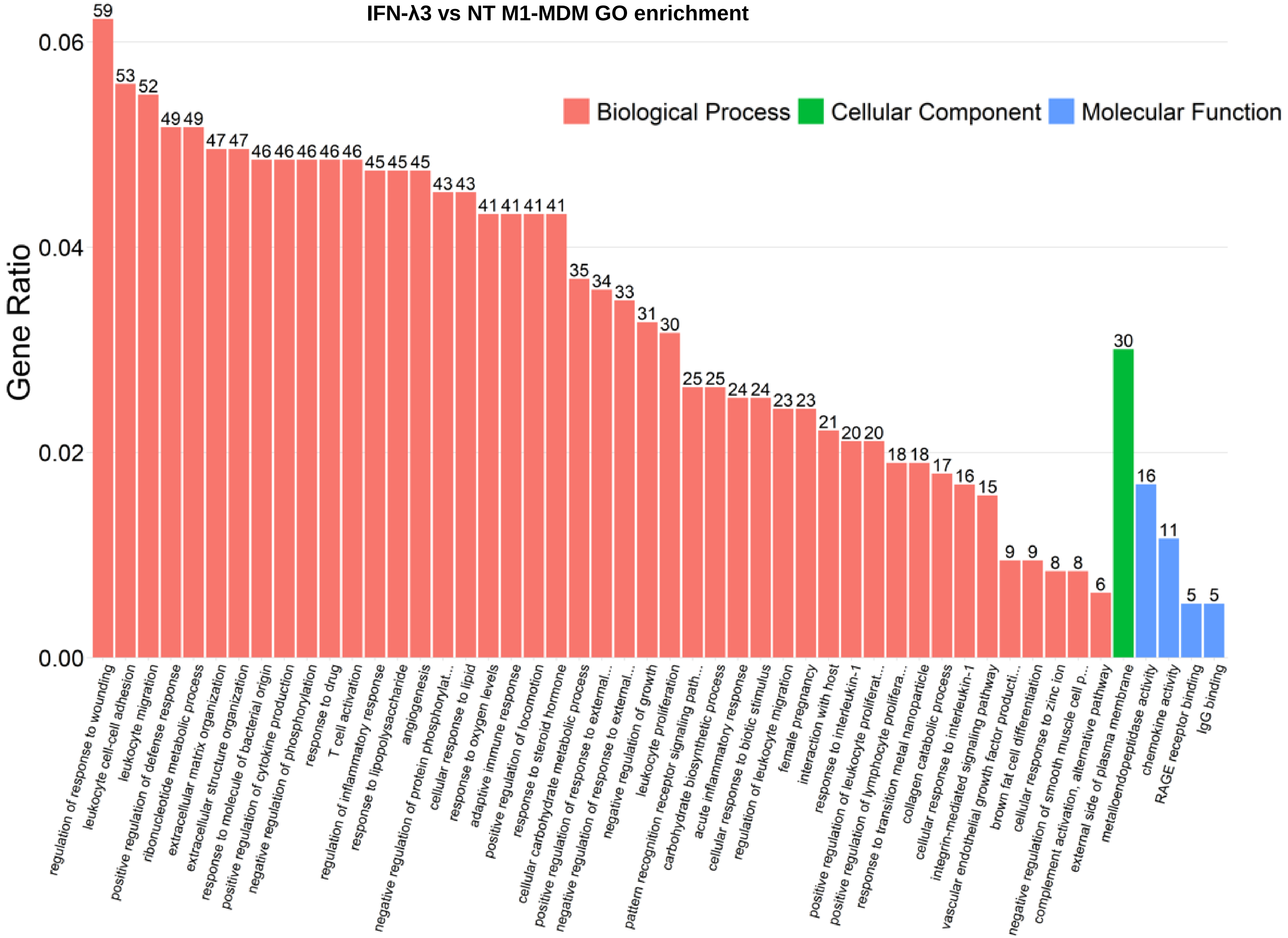

Suppl. Fig. 3B

GO enrichment analysis for IFN-λ4 vs NT M1-MDM.

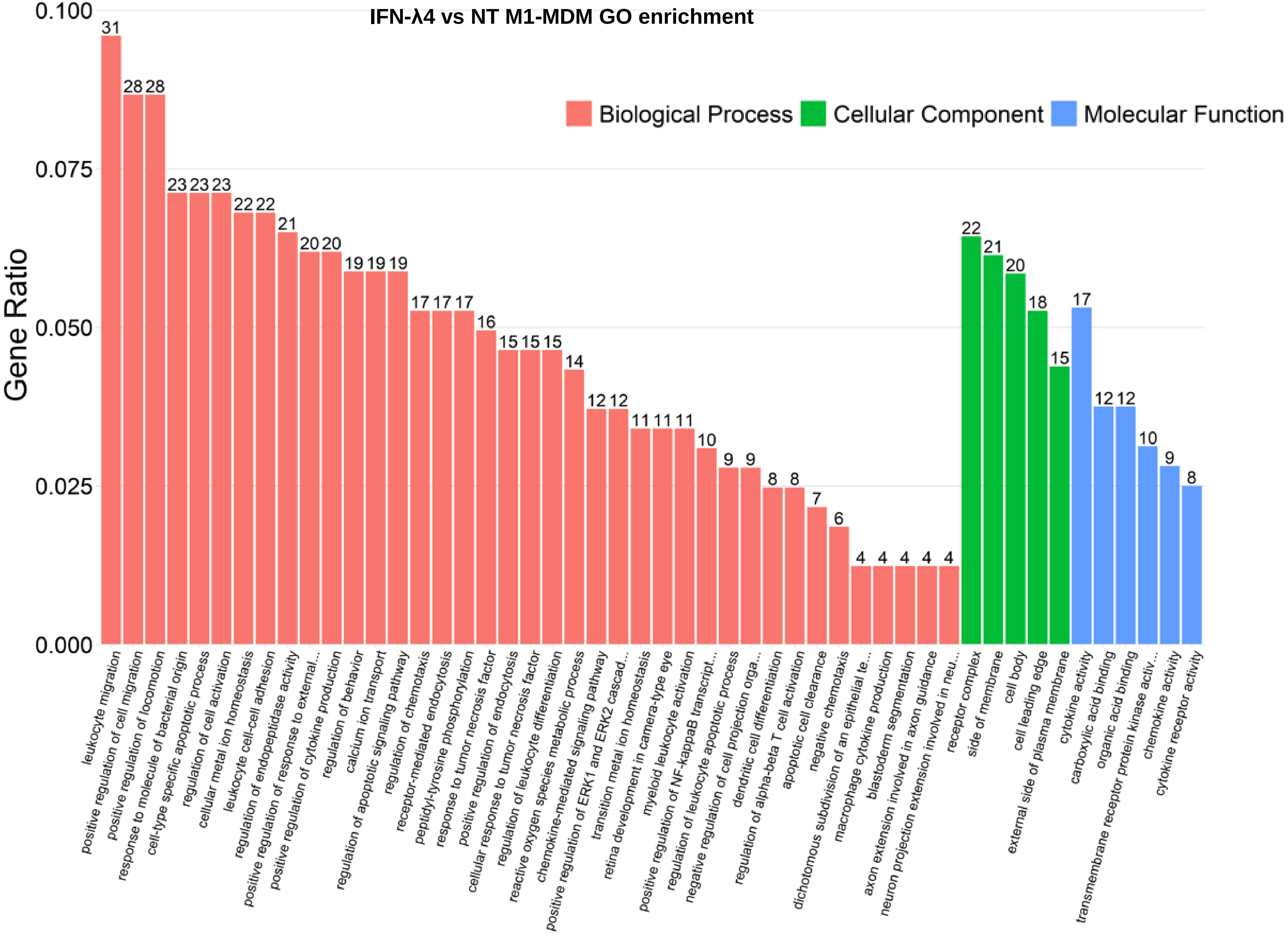

Suppl. Fig. 3C

GO enrichment analysis for IFN-λ4 vs IFN-λ3 M1-MDM.

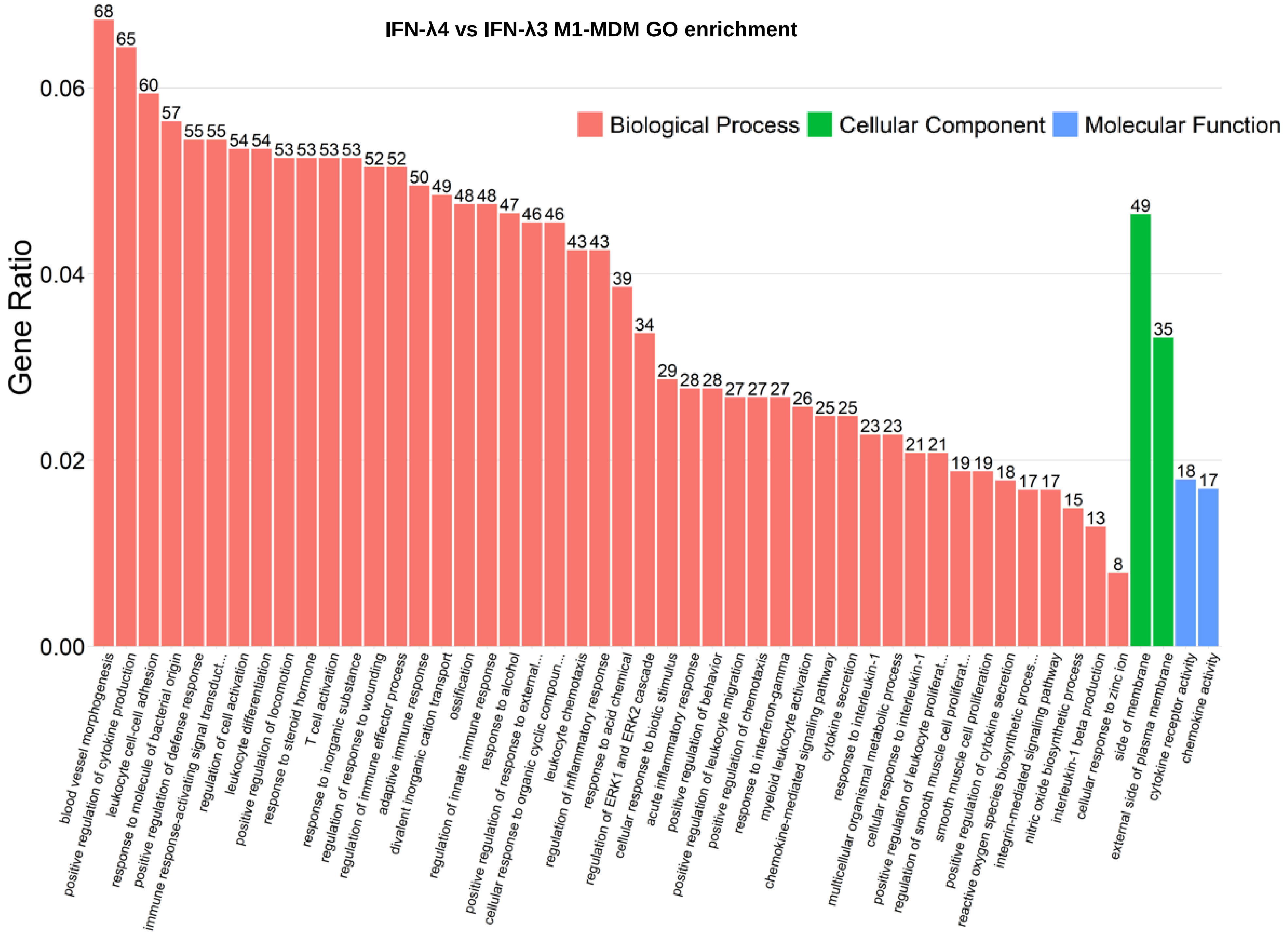

Suppl. Fig. 3D

GO enrichment analysis for IFN-λ3 vs NT M2-MDM.

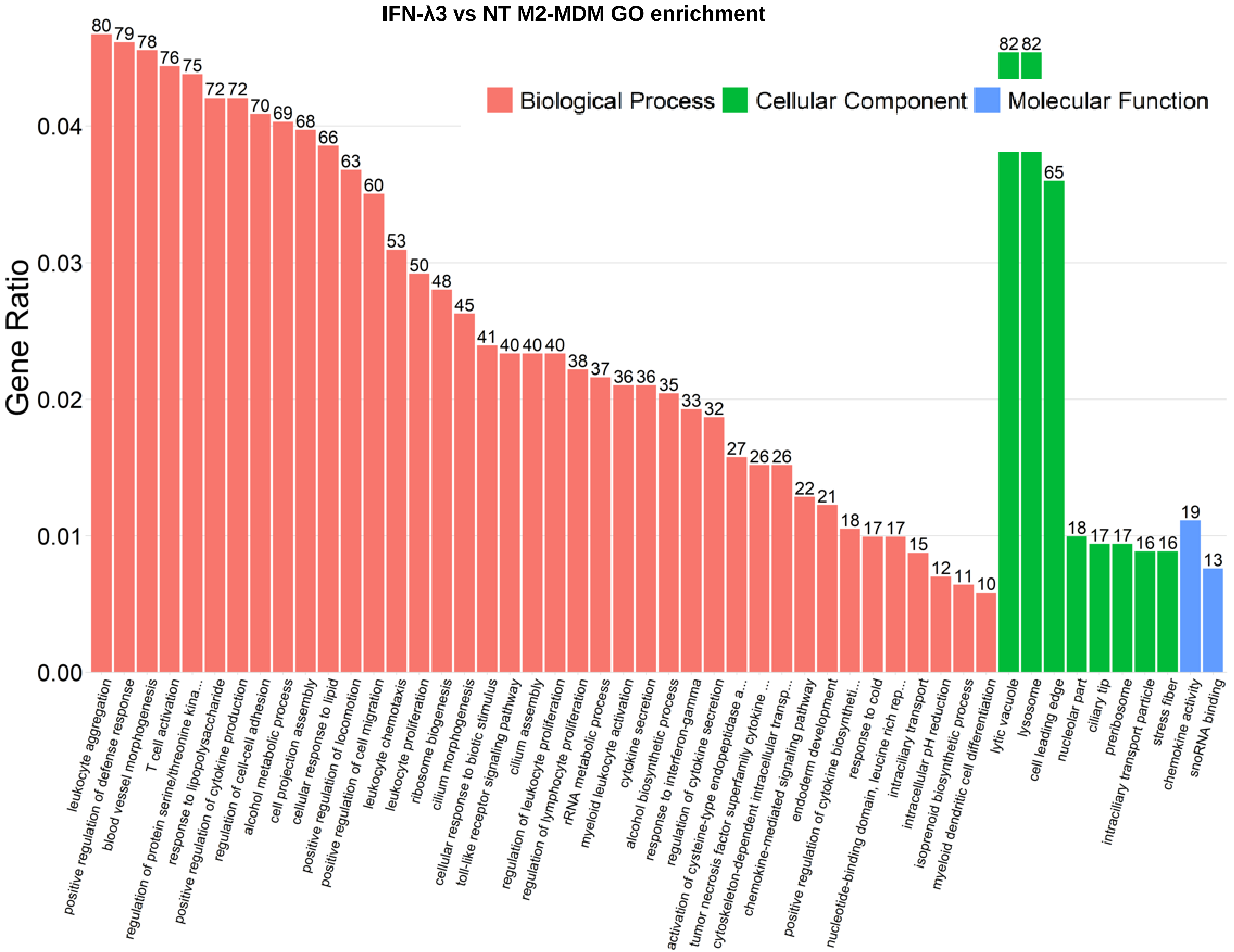

Suppl. Fig. 3E

GO enrichment analysis for IFN-λ4 vs NT M2-MDM.

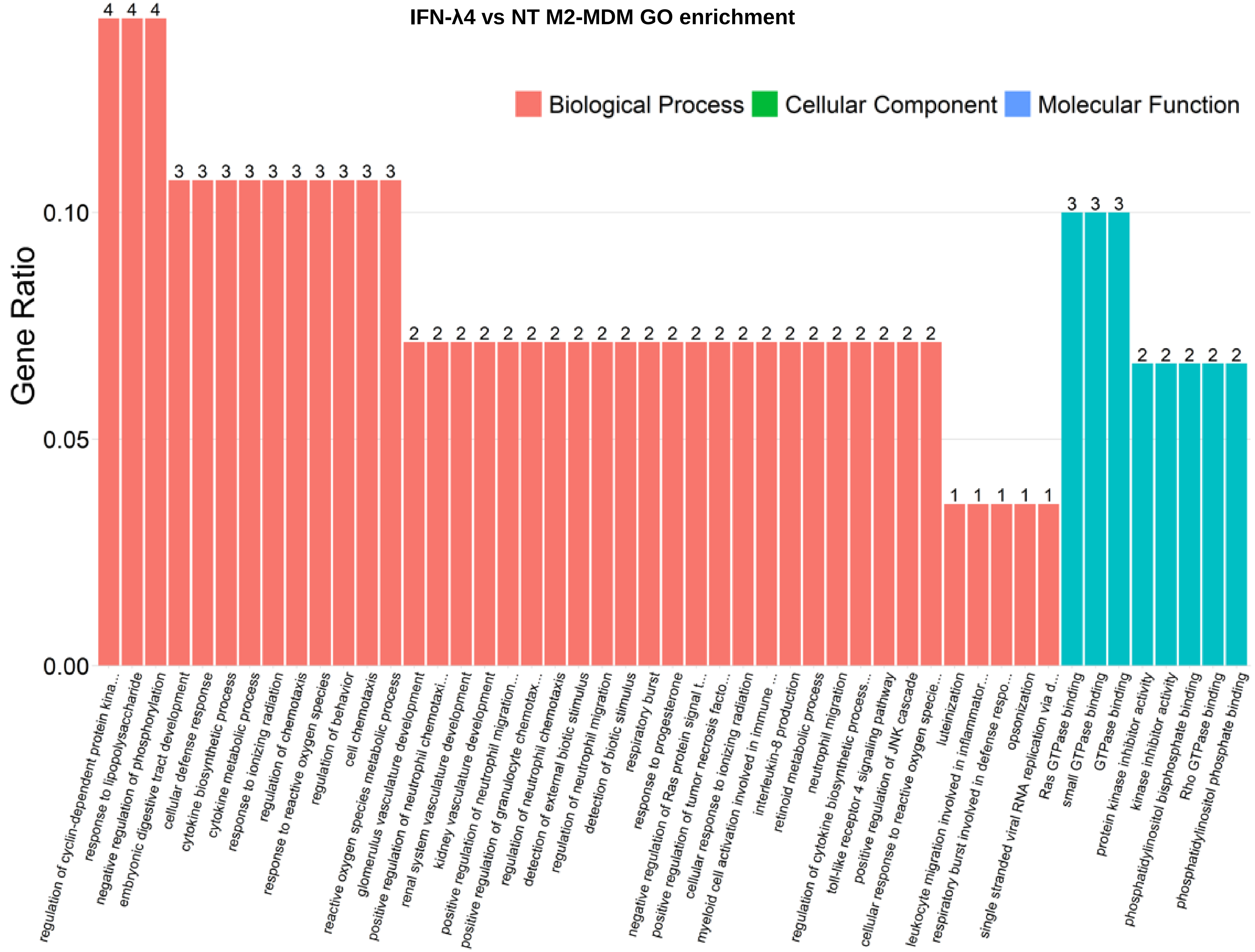

Suppl. Fig. 3F

GO enrichment analysis for IFN-λ4 vs IFN-λ3 M2-MDM.

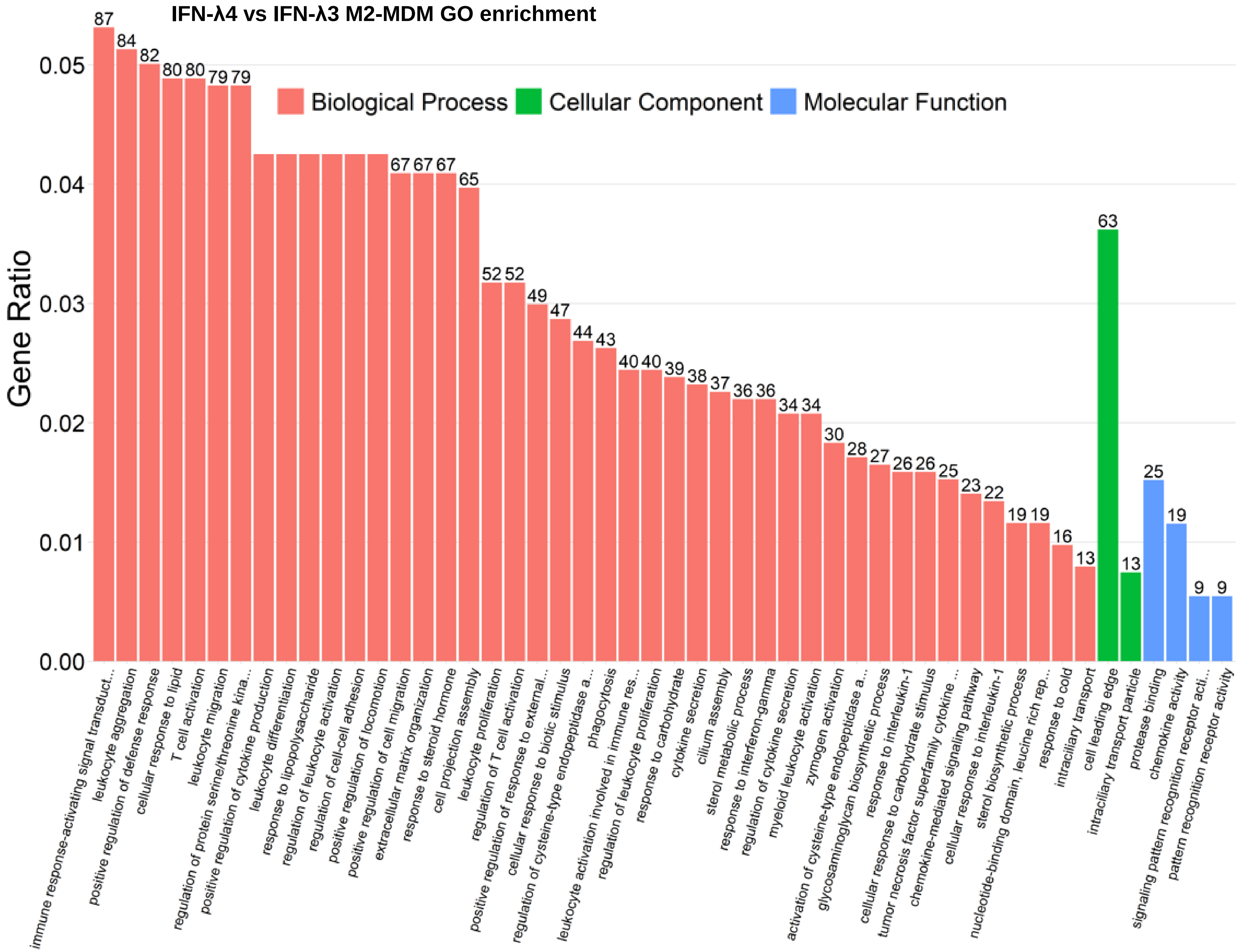

Suppl. Fig. 4

KEGG pathway analysis for the RNA-seq data (M1-MDM) described in (Ref#22), "De, Bhushan and Chinnaswamy. J. Leukocyte Biology. 2021. 110(2): 357-374.

KEGG pathway analysis of RNA-seq data described in Ref# 22; IFN-λ4 vs NT (in duplicates) treatment in M1-MDMs from monocytes derived from a single individual were subject to RNA-seq and KEGG pathway analysis.

KEGG Pathway Enrichment Analysis: pathway enrichment analysis identifies significantly enriched metabolic pathways or signal transduction pathways associated with differentially expressed genes compared with the whole genome background.

$$p = 1 - \sum_{i=0}^{m-1} \frac{\binom{M}{i}\binom{N-M}{n-i}}{\binom{N}{n}}$$

Here N is the number of all genes with KEGG annotation, n is the number of target gene candidates in N, M is the number of all genes annotated to a certain pathway, and m is the number of target gene candidates in M.

Reactome Pathway Enrichment Analysis: Reactome data model generalizes the concept of a reaction to include transformations of entities such as transport from one compartment to another and interaction to form a complex, as well as the chemical transformations of classical biochemistry.

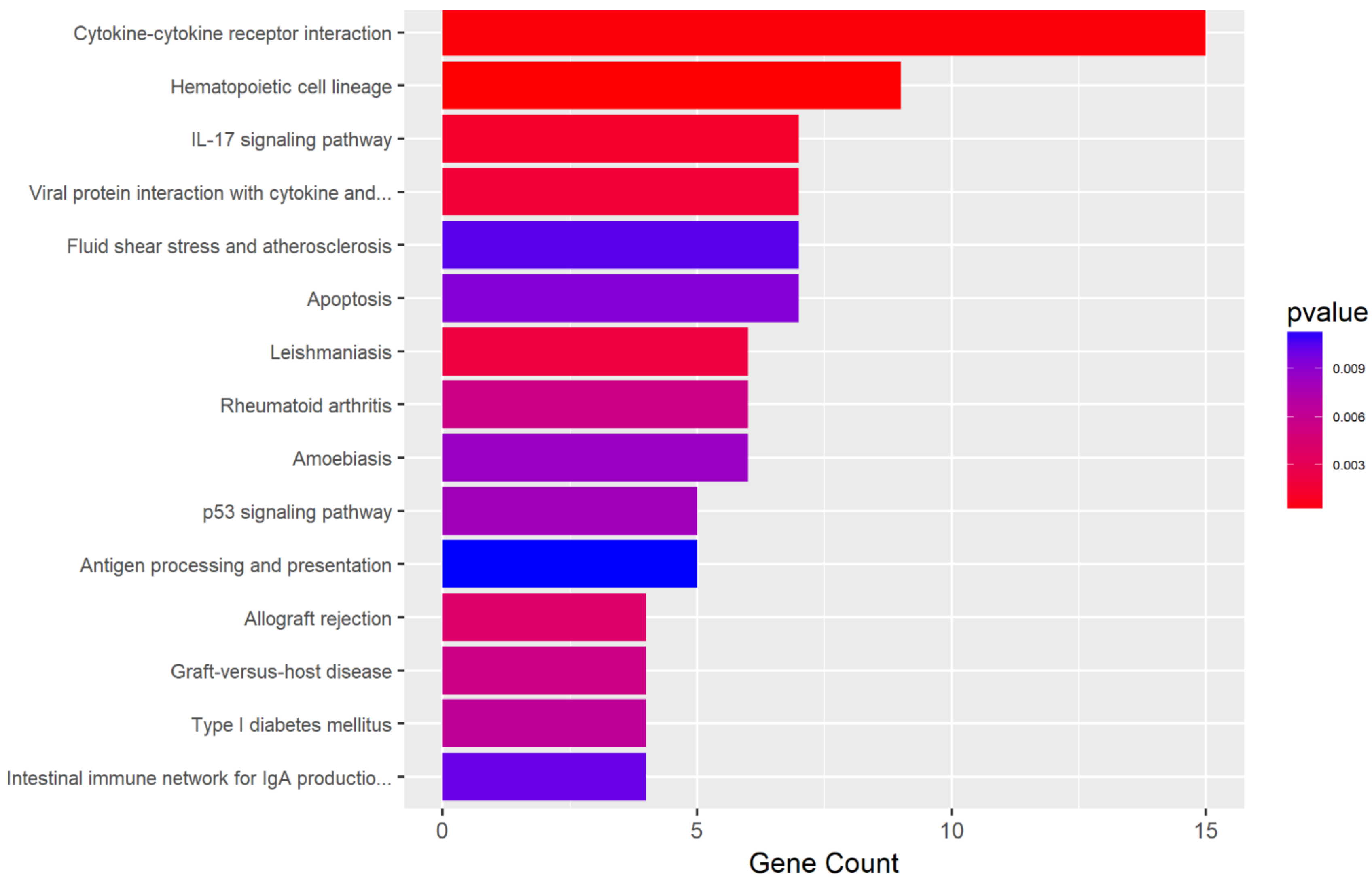

Supplement: Supplementary file 1 — Supplemental Figures [file 41435_2022_164_MOESM1_ESM.pdf]
