# Supplementary material for: Distinct molecular phenotypes involving several human diseases are induced by IFN-λ3 and IFN-λ4 in monocyte-derived macrophages
Source: Genes Immun. 2022 Feb 3;23(2):73–84. doi: 10.1038/s41435-022-00164-w (PMC9042695; doi:10.1038/s41435-022-00164-w)
Supplement: Supplementary file 2 — Supplemental Tables [file 41435_2022_164_MOESM2_ESM.pdf]

Supplementary Tables 1-6 (De et al., Human IFN-λ3 and IFN-λ4 show distinct molecular phenotypes in macrophages)

Table 1: Top 100 genes affected by IFN-λ3 (IFN-λ3 vs NT; M1-MDM)

S.No	Gene symbol	Name	Foldchange	PValue	FDR	Associated diseases	References
1.	CD207	CD207 molecule; langerin	27.55313	0.00016	0.006733	• bubonic plague<br>• Langerhans cells histiocytosis<br>• HIV Infection	• Yang K, Park CG, Cheong C, Bulgheresi S, Zhang S, Zhang P, He Y, Jiang L, Huang H, Ding H, Wu Y. Host Langerin (CD207) is a receptor for Yersinia pestis phagocytosis and promotes dissemination. Immunology and cell biology. 2015 Oct;93(9):815-24.<br>• de Menthon M, Meignin V, Mahr A, Tazi A. Histiocytose à cellules de Langerhans de l'adulte. La Presse Médicale. 2017 Jan 1;46(1):55-69.<br>• Kaldensjö T, Petersson P, Tolf A, Morgan G, Broliden K, Hirbod T. Detection of intraepithelial and stromal Langerin and CCR5 positive cells in the human endometrium: potential targets for HIV infection. PloS one. 2011 Jun 29;6(6):e21344.
2.	ATP1B2	ATPase; Na+/K+ transporting; beta 2 polypeptide	21.18599	1.53E-05	0.001273	• Esophageal squamous cell carcinoma	• Wu C, Wang Z, Song X, Feng XS, Abnet CC, He J, Hu N, Zuo XB, Tan W, Zhan Q, Hu Z. Joint analysis of three genome-wide association studies of esophageal squamous cell carcinoma in Chinese populations. Nature genetics. 2014 Sep;46(9):1001-6.
3.	ENHO	energy homeostasis associated	15.62919	0.000236	0.008859	• Dyslipidemia	• Grzegorzewska AE, Niepolski L, Świderska MK, Mostowska A, Stolarek I, Warchoł W, Figlerowicz M, Jagodziński PP. ENHO, RXRA, and LXRA polymorphisms and dyslipidaemia, related comorbidities and survival in haemodialysis patients. BMC medical genetics. 2018 Dec;19(1):1-8.
4.	CLEC4F	C-type lectin domain family 4; member F	14.36026	0.001233	0.028224	• Hepatocyte necrosis	• Lindquist ME, Zeng X, Altamura LA, Daye SP, Delp KL, Blancett C, Coffin KM, Koehler JW, Coyne S, Shoemaker CJ, Garrison AR. Exploring Crimean-Congo hemorrhagic fever virus-induced hepatic injury using antibody-mediated type I interferon blockade in mice. Journal of virology. 2018 Oct 12;92(21):e01083-18.
5.	CD1E	CD1e molecule	13.0977	0.000108	0.005142	• Celiac disease	• Aureli A, Aboulaghras S, Oumhani K, Del Beato T, Sebastiani P, Colanardi A, El Aouad R, El Barhdadi IB, Piancatelli D. CD1 gene polymorphism and susceptibility to celiac disease: Association of CD1E* 02/02 in Moroccans. Human Immunology. 2020 Jul 1;81(7):361-5.
6.	SLC51B	solute carrier family 51; beta subunit	10.48144	0.00039	0.012569	• Cholestasis	• Sultan M, Rao A, Elpeleg O, Vaz FM, Abu-Libdeh B, Karpen SJ, Dawson PA. Organic solute transporter-β (SLC51B) deficiency in two brothers with congenital diarrhea and features of cholestasis. Hepatology. 2018 Aug;68(2):590-8.
7.	GTF2H2C	GTF2H2 family member C	9.145261	0.017436	0.143113	• Coronary heart disease	• Fragou D, Pakkidi E, Aschner M, Samanidou V, Kovatsi L. Smoking and DNA methylation: Correlation of methylation with smoking behavior and association with diseases and fetus development following prenatal exposure. Food and Chemical Toxicology. 2019 Jul 1;129:312-27.
8.	ITGA11	integrin; alpha 11	7.645454	0.000123	0.005671	• Non-Small Cell Lung Carcinoma	• Ando T, Kage H, Matsumoto Y, Zokumasu K, Yotsumoto T, Maemura K, Amano Y, Watanabe K, Nakajima J, Nagase T, Takai D. Integrin α11 in

							non–small cell lung cancer is associated with tumor progression and postoperative recurrence. Cancer science. 2020 Jan;111(1):200-8.
9.	TINAGL1	tubulointerstitial nephritis antigen-like 1	6.378747	0.004273	0.060536	• Liver carcinoma	• Sun L, Dong Z, Gu H, Guo Z, Yu Z. TINAGL1 promotes hepatocellular carcinogenesis through the activation of TGF-β signaling-medicated VEGF expression. Cancer management and research. 2019;11:767.
10.	CYP4F22	cytochrome P450; family 4; subfamily F; polypeptide 22	6.204716	0.004925	0.066438	• Congenital Nonbullous Ichthyosiform Erythroderma	• Gruber R, Rainer G, Weiss A, Udvardi A, Thiele H, Eckl KM, Schupart R, Nürnberg P, Zschocke J, Schmuth M, Volc-Platzer B. Morphological alterations in two siblings with autosomal recessive congenital ichthyosis associated with CYP 4F22 mutations. British Journal of Dermatology. 2017 Apr;176(4):1068-73.
11.	DSG2	desmoglein 2	6.194617	0.00442	0.061721	• Arrhythmogenic right ventricular dysplasia/cardiomyopathy	• Awad MM, Dalal D, Cho E, Amat-Alarcon N, James C, Tichnell C, Tucker A, Russell SD, Bluemke DA, Dietz HC, Calkins H. DSG2 mutations contribute to arrhythmogenic right ventricular dysplasia/cardiomyopathy. The American Journal of Human Genetics. 2006 Jul 1;79(1):136-42.<br>• Lin Y, Huang J, Zhao T, He S, Huang Z, Chen X, Fei H, Luo H, Liu H, Wu S, Lin X. Compound and heterozygous mutations of DSG2 identified by Whole Exome Sequencing in arrhythmogenic right ventricular cardiomyopathy/dysplasia with ventricular tachycardia. Journal of electrocardiology. 2018 Sep 1;51(5):837-43.
12.	PLAT	plasminogen activator; tissue type	6.184967	0.000111	0.00524	• Cerebral hemorrhage<br>• Reperfusion injury	• Naganuma M, Koga M, Shiokawa Y, Nakagawara J, Furui E, Kimura K, Yamagami H, Okada Y, Hasegawa Y, Kario K, Okuda S. Reduced estimated glomerular filtration rate is associated with stroke outcome after intravenous rt-PA: the Stroke Acute Management with Urgent Risk-Factor Assessment and Improvement (SAMURAI) rt-PA registry. Cerebrovascular Diseases. 2011;31(2):123-9.<br>• Grotta JC, Alexandrov AV. tPA-associated reperfusion after acute stroke demonstrated by SPECT. Stroke. 1998 Feb;29(2):429-32.
13.	MMP12	matrix metallopeptidase 12	5.902733	0.000412	0.013113	• Pulmonary emphysema	• Doyle AD, Mukherjee M, LeSuer WE, Bittner TB, Pasha SM, Frere JJ, Neely JL, Kloeber JA, Shim KP, Ochkur SI, Ho T. Eosinophil-derived IL-13 promotes emphysema. European Respiratory Journal. 2019 May 1;53(5).
14.	CD1A	CD1a molecule	5.03933	0.005951	0.07449	• Langerhans cells histiocytosis<br>• Liver gastrointestinal cancer	• El Sissy FN, Lorillon G, Mandonnet E, Polivka M, Addle-Biassette H, Jean-François E. BRAF-mutated histiocytosis of the skull lacking the expression of Langerhans cell markers. Clinical neuropathology. 2020 Mar 1;39(2):64.<br>• Gulubova M, Manolova I, Ananiev J, Kjurkchiev D, Julianov A, Altunkova I. Relationship of TGF-β1 and Smad7 expression with decreased dendritic cell infiltration in liver gastrointestinal cancer metastasis. Apmis. 2013 Oct;121(10):967-75.
15.	PMF1-BGLAP	PMF1-BGLAP readthrough	4.94261	0.002557	0.043545	• Stroke	• Wang L, Xu F, Brickell A, Sun N, Mao X, Zhang Q, Wang G, Zhou Q, Yang B, Li F, Yue L. Additional common loci associated with stroke and obesity identified using pleiotropic analytical approach. Molecular Genetics and Genomics. 2020 Mar;295(2):439-51.
16.	CD1B	CD1b molecule	4.893187	1.52E-07	4.07E-05	• Tuberculosis	• Chancellor A, Tocheva AS, Cave-Ayland C, Tezera L, White A, Juma'a R, Bridgeman JS, Tews I, Wilson S, Lissin NM, Tebruegge M. CD1b-restricted GEM T cell responses are modulated by Mycobacterium tuberculosis mycolic acid meromycolate chains. Proceedings of the National Academy of Sciences. 2017 Dec 19;114(51):E10956-64.

17.	COL23A1	collagen; type XXIII; alpha 1	4.467163	0.000562	0.016118	• Clear cell renal cell carcinoma	• Xu F, Chang K, Ma J, Qu Y, Xie H, Dai B, Gan H, Zhang H, Shi G, Zhu Y, Zhu Y. The oncogenic role of COL23A1 in clear cell renal cell carcinoma. Scientific reports. 2017 Aug 29;7(1):1-9.
18.	CPNE6	copine VI (neuronal)	4.40343	0.013637	0.121631	• Epilepsy	• Zhu B, Zha J, Long Y, Hu X, Chen G, Wang X. Increased expression of copine VI in patients with refractory epilepsy and a rat model. Journal of the neurological sciences. 2016 Jan 15;360:30-6.
19.	ADGRG2	adhesion G protein-coupled receptor G2	4.247021	3.32E-05	0.00223	• Congenital bilateral aplasia of vas deferens<br>• Male infertility	• Yang B, Wang J, Zhang W, Pan H, Li T, Liu B, Li H, Wang B. Pathogenic role of ADGRG 2 in CBAVD patients replicated in Chinese population. Andrology. 2017 Sep;5(5):954-7.<br>• Zhang DL, Sun YJ, Ma ML, Wang YJ, Lin H, Li RR, Liang ZL, Gao Y, Yang Z, He DF, Lin A. Gq activity-and β-arrestin-1 scaffolding-mediated ADGRG2/CFTR coupling are required for male fertility. Elife. 2018 Feb 2;7:e33432.
20.	OLFM2	olfactomedin 2	4.246847	0.000479	0.014501	• Liver carcinoma<br>• Glaucoma, Open-Angle	• Zhang R, Ye J, Huang H, Du X. Mining featured biomarkers associated with vascular invasion in HCC by bioinformatics analysis with TCGA RNA sequencing data. Biomedicine & Pharmacotherapy. 2019 Oct 1;118:109274.<br>• Funayama T, Mashima Y, Ohtake Y, Ishikawa K, Fuse N, Yasuda N, Fukuchi T, Murakami A, Hotta Y, Shimada N, Glaucoma Gene Research Group. SNPs and interaction analyses of noelin 2, myocilin, and optineurin genes in Japanese patients with open-angle glaucoma. Investigative ophthalmology & visual science. 2006 Dec 1;47(12):5368-75.
21.	FST	follistatin	4.245115	0.016617	0.138849	• Breast carcinoma<br>• Liver carcinoma<br>• Miscarriage	• Zabkiewicz C, Resaul J, Hargest R, Jiang WG, Ye L. Increased expression of follistatin in breast cancer reduces invasiveness and clinically correlates with better survival. Cancer genomics & proteomics. 2017 Jul 1;14(4):241-51.<br>• Shi L, Resaul J, Owen S, Ye L, Jiang WG. Clinical and therapeutic implications of follistatin in solid tumours. Cancer genomics & proteomics. 2016 Nov 1;13(6):425-35.<br>• Fullerton PT, Monsivais D, Kommagani R, Matzuk MM. Follistatin is critical for mouse uterine receptivity and decidualization. Proceedings of the National Academy of Sciences. 2017 Jun 13;114(24):E4772-81.
22.	CCND2	cyclin D2	4.214359	2.96E-09	1.74E-06	• Rhabdomyosarcoma	• Li L, Sarver AL, Alamgir S, Subramanian S. Downregulation of microRNAs miR-1,-206 and-29 stabilizes PAX3 and CCND2 expression in rhabdomyosarcoma. Laboratory investigation. 2012 Apr;92(4):571-83.
23.	NDRG2	NDRG family member 2	4.056135	0.000159	0.006698	• Malignant tumour of colon	• Lorentzen A, Vogel LK, Lewinsky RH, Sæbø M, Skjelbred CF, Godiksen S, Hoff G, Tveit KM, Lothe IM, Ikdahl T, Kure EH. Expression of NDRG2 is down-regulated in high-risk adenomas and colorectal carcinoma. BMC cancer. 2007 Dec;7(1):1-8.
24.	IL1R2	interleukin 1 receptor; type II	3.968416	0.000496	0.014911	• Ulcerative colitis	• Yoshida K, Murayama MA, Shimizu K, Tang C, Katagiri N, Matsuo K, Fukai F, Iwakura Y. IL-1R2 deficiency suppresses dextran sodium sulfate-induced colitis in mice via regulation of microbiota. Biochemical and biophysical research communications. 2018 Feb 12;496(3):934-40.
25.	CCL1	chemokine (C-C motif) ligand 1	3.856524	4.72E-06	0.000549	• Liver carcinoma	• Wiedemann GM, Röhrle N, Makeschin MC, Fesseler J, Endres S, Mayr D, Anz D. Peritumoural CCL1 and CCL22 expressing cells in hepatocellular carcinomas shape the tumour immune infiltrate. Pathology. 2019 Oct 1;51(6):586-92.
26.	TSPAN2	tetraspanin 2	3.761509	0.001312	0.029245	• Lung cancer	• Otsubo C, Otomo R, Miyazaki M, Matsushima-Hibiya Y, Kohno T, Iwakawa R, Takeshita F, Okayama H, Ichikawa H, Saya H, Kiyono T. TSPAN2 is

							involved in cell invasion and motility during lung cancer progression. Cell reports. 2014 Apr 24;7(2):527-38.
27.	VASN	vasorin	3.483602	0.004496	0.062515	• Hepatocellular carcinoma	• Da Li TZ, Yang X, Geng J, Li S, Ding H, Li H, Huang A, Wang C, Sun L, Bai C, Zhang H. Identification of functional mimotopes of human vasorin ectodomain by biopanning. International journal of biological sciences. 2018;14(4):461.
28.	GP1BA	glycoprotein Ib (platelet); alpha polypeptide	3.396357	0.035879	0.213186	• Bernard-Soulier syndrome	• Özdemir ZC, Kar YD, Ceylaner S, Bör Ö. A novel mutation in the GP1BA gene in Bernard–Soulier syndrome. Blood Coagulation & Fibrinolysis. 2020 Jan 1;31(1):83-6.
29.	CLEC10A	C-type lectin domain family 10; member A	3.378708	0.003436	0.053047	• Lung adenocarcinoma	• He M, Han Y, Cai C, Liu P, Chen Y, Shen H, Xu X, Zeng S. CLEC10A is a prognostic biomarker and correlated with clinical pathologic features and immune infiltrates in lung adenocarcinoma. Journal of Cellular and Molecular Medicine. 2021 Apr;25(7):3391-9.
30.	TACSTD2	tumor-associated calcium signal transducer 2	3.368786	0.004985	0.06693	• Familial Gelatinous Drop-Like Corneal Dystrophy	• Cabral-Macias J, Zenteno JC, Ramirez-Miranda A, Navas A, Bermudez-Magner JA, Boullosa-Graña VM, Graue-Hernandez EO, Buentello-Volante B. Familial Gelatinous Drop-Like Corneal Dystrophy Caused by a Novel Nonsense TACSTD2 Mutation. Cornea. 2016 May 9;35(7):987-90.
31.	TRIB2	tribbles pseudokinase 2	3.366329	6.85E-07	0.000125	• Myeloid leukemia<br>• Colorectal cancer<br>• Liver cancer	• Salomé M, Magee A, Yalla K, Chaudhury S, Sarrou E, Carmody RJ, Keeshan K. A Trib2-p38 axis controls myeloid leukaemia cell cycle and stress response signalling. Cell death & disease. 2018 Apr 18;9(5):1-7.<br>• Hou Z, Guo K, Sun X, Hu F, Chen Q, Luo X, Wang G, Hu J, Sun L. TRIB2 functions as novel oncogene in colorectal cancer by blocking cellular senescence through AP4/p21 signaling. Molecular cancer. 2018 Dec;17(1):1-5.<br>• Guo S, Chen Y, Yang Y, Zhang X, Ma L, Xue X, Qiao Y, Wang J. TRIB2 modulates proteasome function to reduce ubiquitin stability and protect liver cancer cells against oxidative stress. Cell death & disease. 2021 Jan 7;12(1):1-8.
32.	TNFRSF4	tumor necrosis factor receptor superfamily; member 4	3.350243	0.000948	0.023421	• Immunodeficiency 16<br>• Immune thrombocytopenia	• Byun M, Ma CS, Akçay A, Pedergnana V, Palendira U, Myoung J, Avery DT, Liu Y, Abhyankar A, Lorenzo L, Schmidt M. Inherited human OX40 deficiency underlying classic Kaposi sarcoma of childhoodHuman OX40 deficiency. The Journal of experimental medicine. 2013 Aug 26;210(9):1743-59.<br>• Cui D, Lv Y, Yuan X, Ruan G, Zhang Y, Yan C, Xu D, Lv M, Mao Y, Cao J, Jin J. Increased expressions of OX40 and OX40 ligand in patients with primary immune thrombocytopenia. Journal of immunology research. 2019 Mar 3;2019.
33.	CACNA1G	calcium channel; voltage-dependent; T type; alpha 1G subunit	3.338829	0.015478	0.131781	• Spinocerebellar ataxia	• Barresi S, Dentici ML, Manzoni F, Bellacchio E, Agolini E, Pizzi S, Ciolfi A, Tarnopolsky M, Brady L, Garone G, Novelli A. Infantile-onset syndromic cerebellar ataxia and CACNA1G mutations. Pediatric neurology. 2020 Mar 1;104:40-5.
34.	NPR1	natriuretic peptide receptor 1	3.307361	3.70E-06	0.000466	• Hypertension	• Kumar P, Gogulamudi VR, Periasamy R, Raghavaraju G, Subramanian U, Pandey KN. Inhibition of HDAC enhances STAT acetylation, blocks NF-κB, and suppresses the renal inflammation and fibrosis in Npr1 haplotype male mice. American Journal of Physiology-Renal Physiology. 2017 Sep 1;313(3):F781-95.
35.	HOPX	HOP homeobox	3.29259	0.007394	0.08472	• Papillary thyroid cancer	• Ooizumi Y, Katoh H, Yokota M, Watanabe M, Yamashita K. Epigenetic silencing of HOPX is critically involved in aggressive phenotypes and

							patient prognosis in papillary thyroid cancer. Oncotarget. 2019 Oct 15;10(57):5906.
36.	ADGRA2	adhesion G protein-coupled receptor A2	3.27652	0.000977	0.023706	• Cancer	• Purcell RH, Hall RA. Adhesion G protein–coupled receptors as drug targets. Annual review of pharmacology and toxicology. 2018 Jan 6;58:429-49.
37.	IGSF3	immunoglobulin superfamily; member 3	3.269329	0.000567	0.016163	• Congenital dacryocystocele	• Foster J, Kapoor S, Diaz-Horta O, Singh A, Abad C, Rastogi A, Moharana R, Tekeli O, Walz K, Tekin M. Identification of an IGSF3 mutation in a family with congenital nasolacrimal duct obstruction. Clinical genetics. 2014 Dec;86(6):589-91.
38.	PLA1A	phospholipase A1 member A	3.232797	4.38E-06	0.000519	• HCV	• Yang Q, Guo M, Zhou Y, Hu X, Wang Y, Wu C, Yang M, Pei R, Chen X, Chen J. Phosphatidylserine-specific phospholipase A1 is the critical bridge for hepatitis C virus assembly. Virologica Sinica. 2019 Oct;34(5):521-37.
39.	SH3BP4	SH3-domain binding protein 4	3.220215	0.000165	0.006877	• Tumorigenesis	• Antas P, Novellasdemunt L, Kucharska A, Massie I, Carvalho J, Oukrif D, Nye E, Novelli M, Li VS. SH3BP4 regulates intestinal stem cells and tumorigenesis by modulating β-Catenin nuclear localization. Cell reports. 2019 Feb 26;26(9):2266-73.
40.	DMPK	dystrophia myotonica-protein kinase	3.186579	0.002517	0.043036	• Myotonic dystrophy 1	• Yamamoto T, Miura A, Itoh K, Takeshima Y, Nishio H. RNA sequencing reveals abnormal LDB3 splicing in sudden cardiac death. Forensic science international. 2019 Sep 1;302:109906.
41.	SYT12	synaptotagmin XII	3.181378	0.027698	0.185444	• Oral cancer	• Eizuka K, Nakashima D, Oka N, Wagai S, Takahara T, Saito T, Koike K, Kasamatsu A, Shiiba M, Tanzawa H, Uzawa K. SYT12 plays a critical role in oral cancer and may be a novel therapeutic target. Journal of Cancer. 2019;10(20):4913.
42.	CD1C	CD1c molecule	3.155951	0.000334	0.011183	• Rheumatoid arthritis	• Ashton MP, Eugster A, Dietz S, Loebel D, Lindner A, Kuehn D, Taranko AE, Heschel B, Gavrisan A, Ziegler AG, Aringer M. Association of Dendritic Cell Signatures With Autoimmune Inflammation Revealed by Single-Cell Profiling. Arthritis & Rheumatology. 2019 May;71(5):817-28.
43.	LINC00520	long intergenic non-protein coding RNA 520	3.141258	1.91E-05	0.00148	• Lung cancer<br>• Acute kidney injury	• Xia G, Li X, Chen F, Shao Z. LncRNA LINC00520 Predicts Poor Prognosis and Promotes Progression of Lung Cancer by Inhibiting MiR-3175 Expression. Cancer Management and Research. 2020;12:5741.<br>• Tian X, Ji Y, Liang Y, Zhang J, Guan L, Wang C. LINC00520 targeting miR-27b-3p regulates OSMR expression level to promote acute kidney injury development through the PI3K/AKT signaling pathway. Journal of cellular physiology. 2019 Aug;234(8):14221-33.
44.	CLDN1	claudin 1	2.998908	0.017253	0.142178	• Ichthyosis, Sclerosing Cholangitis and Acquired Alopecia<br>• Lung cancer	• Alsafri MS, Charbit-Henrion F, Lacaille F, Bourrat E, Steffann J, Hadj-Rabia S. Novel CLDN1 Deletion Associated With Ichthyosis, Sclerosing Cholangitis and Acquired Alopecia. Acta dermato-venereologica. 2020 Jun 11;100(13):adv00173.<br>• Wu JE, Wu YY, Tung CH, Tsai YT, Chen HY, Chen YL, Hong TM. DNA methylation maintains the CLDN1-EPHB6-SLUG axis to enhance chemotherapeutic efficacy and inhibit lung cancer progression. Theranostics. 2020;10(19):8903.
45.	RASGRF1	Ras protein-specific guanine nucleotide-releasing factor 1	2.996825	0.000728	0.019507	• Myopia	• Kunceviciene E, Sriubiene M, Liutkeviciene R, Miceikiene IT, Smalinskiene A. Heritability of myopia and its relation with GDJ2 and RASGRF1 genes in Lithuania. BMC ophthalmology. 2018 Dec;18(1):1-6.
46.	HOMER2	homer scaffolding protein 2	2.987759	0.004032	0.058039	• endometrial adenocarcinoma<br>• schizophrenia	• Mhawech-Fauceglia P, Walia S, Yessaian A, Machida H, Matsuo K, Lawrenson K. Overexpression of HOMER2 predicts better outcome in low-grade endometrioid endometrial adenocarcinoma. Pathology. 2018 Aug 1;50(5):499-503.<br>• Gilks WP, Allott EH, Donohoe G, Cummings E, Gill M, Corvin AP, Morris DW, International Schizophrenia Consortium. Replicated genetic

							evidence supports a role for HOMER2 in schizophrenia. Neuroscience letters. 2010 Jan 14;468(3):229-33.
47.	ITGB7	integrin; beta 7	2.96223	6.54E-05	0.003589	• Pancreatic cancer<br>• Fulminant type-1 diabetes	• Sun Q, Ye Z, Qin Y, Fan G, Ji S, Zhuo Q, Xu W, Liu W, Hu Q, Liu M, Zhang Z. Oncogenic function of TRIM2 in pancreatic cancer by activating ROS-related NRF2/ITGB7/FAK axis. Oncogene. 2020 Oct;39(42):6572-88.<br>• Kawabata Y, Nishida N, Awata T, Kawasaki E, Imagawa A, Shimada A, Osawa H, Tanaka S, Takahashi K, Nagata M, Yasuda H. Genome-wide association study confirming a strong effect of HLA and identifying variants in CSAD/lnc-ITGB7-1 on chromosome 12q13. 13 associated with susceptibility to fulminant type 1 diabetes. Diabetes. 2019 Mar 1;68(3):665-75.
48.	C10orf128	chromosome 10 open reading frame 128	2.924428	0.000725	0.019507	• oral squamous cell carcinoma	• Kim KY, Zhang X, Cha IH. Combined genomic expressions as a diagnostic factor for oral squamous cell carcinoma. Genomics. 2014 May 1;103(5-6):317-22.
49.	OTOF	otoferlin	2.866537	0.000245	0.009047	• Deafness, Autosomal Recessive 9	• Akil O, Dyka F, Calvet C, Emptoz A, Lahlou G, Nouaille S, de Monvel JB, Hardelin JP, Hauswirth WW, Avan P, Petit C. Dual AAV-mediated gene therapy restores hearing in a DFNB9 mouse model. Proceedings of the National Academy of Sciences. 2019 Mar 5;116(10):4496-501.
50.	CCL22	chemokine (C-C motif) ligand 22	2.861227	0.008151	0.089777	• Atopic dermatitis	• Miake S, Tsuji G, Takemura M, Hashimoto-Hachiya A, Vu YH, Furue M, Nakahara T. IL-4 augments IL-31/IL-31 receptor alpha interaction leading to enhanced Ccl 17 and Ccl 22 production in dendritic cells: implications for atopic dermatitis. International journal of molecular sciences. 2019 Jan;20(16):4053.

51.	CEACAM8	carcinoembryonic antigen-related cell adhesion molecule 8	-138.856	1.74E-06	0.000274	• Colorectal cancer	• Hu X, Li YQ, Ma XJ, Zhang L, Cai SJ, Peng JJ. A risk signature with inflammatory and T immune cells infiltration in colorectal cancer predicting distant metastases and efficiency of chemotherapy. Frontiers in oncology. 2019 Aug 13;9:704.
52.	SERPINB2	serpin peptidase inhibitor; clade B (ovalbumin); member 2	-45.5896	5.48E-08	1.81E-05	• Kidney injury<br>• Aging	• Sen P, Helmke A, Liao CM, Sörensen-Zender I, Rong S, Bräsen JH, Melk A, Haller H, von Vietinghoff S, Schmitt R. SerpinB2 regulates immune response in kidney injury and aging. Journal of the American Society of Nephrology. 2020 May 1;31(5):983-95.
53.	ACKR3	atypical chemokine receptor 3	-38.6404	2.46E-09	1.62E-06	• Autoimmune diseases	• García-Cuesta EM, Santiago CA, Vallejo-Díaz J, Juarranz Y, Rodríguez-Frade JM, Mellado M. The role of the CXCL12/CXCR4/ACKR3 axis in autoimmune diseases. Frontiers in endocrinology. 2019 Aug 27;10:585.
54.	DNER	delta/notch-like EGF repeat containing	-24.3093	7.45E-05	0.003928	• Breast cancer<br>• Chronic obstructive pulmonary disease	• Wang Z, Li Z, Wu Q, Li C, Li J, Zhang Y, Wang C, Sun S, Sun S. DNER promotes epithelial–mesenchymal transition and prevents chemosensitivity through the Wnt/β-catenin pathway in breast cancer. Cell death & disease. 2020 Aug 18;11(8):1-6.<br>• Ballester-López C, Conlon TM, Ertüz Z, Greiffo FR, Irmler M, Verleden SE, Beckers J, Fernandez IE, Eickelberg O, Yildirim AÖ. The Notch ligand DNER regulates macrophage IFNγ release in chronic obstructive pulmonary disease. EBioMedicine. 2019 May 1;43:562-75.
55.	SERPINB7	serpin peptidase inhibitor; clade B (ovalbumin); member 7	-22.0323	2.14E-06	0.000309	• Nagashima-type palmoplantar keratoderma	• Hashimoto T, Teye K, Numata S, Suga Y, Hamada T, Ishii N. Detection of SERPINB 7 mutation can distinguish Nagashima-type palmoplantar keratoderma from other keratodermas with palmoplantar lesions. Clinical and experimental dermatology. 2017 Apr;42(3):342-5.

56.	LRRC38	leucine rich repeat containing 38	-19.4235	0.001343	0.029566	NA	NA
57.	OSMR	oncostatin M receptor	-18.429	4.30E-08	1.51E-05	• Inflammatory bowl disease	• Du Q, Qian Y, Xue W. Molecular Simulation of Oncostatin M and Receptor (OSM–OSMR) Interaction as a Potential Therapeutic Target for Inflammatory Bowel Disease. Frontiers in molecular biosciences. 2020 Mar 4;7:29.
58.	PLOD2	procollagen-lysine; 2-oxoglutarate 5-dioxygenase 2	-16.2227	3.71E-16	1.56E-12	• Bruck syndrome 2	• Puig-Hervás MT, Temtamy S, Aglan M, Valencia M, Martínez-Glez V, Ballesta-Martínez MJ, López-González V, Ashour AM, Amr K, Pulido V, Guillén-Navarro E. Mutations in PLOD2 cause autosomal-recessive connective tissue disorders within the Bruck syndrome—osteogenesis imperfecta phenotypic spectrum. Human mutation. 2012 Oct;33(10):1444-9.
59.	RNASE2	ribonuclease; RNase A family; 2 (liver; eosinophil-derived neurotoxin)	-15.8116	2.34E-15	7.36E-12	• Rheumatoid arthritis	• Fodil M, Teixeira VH, Chaudru V, Hilliquin P, Bombardieri S, Balsa A, Westhovens R, Barrera P, Alves H, Migliorin P, Bardin T. Relationship between SNPs and expression level for candidate genes in rheumatoid arthritis. Scandinavian journal of rheumatology. 2015 Jan 2;44(1):2-7.
60.	SLC8A3	solute carrier family 8 (sodium/calcium exchanger); member 3	-14.6559	1.97E-06	0.000295	• Rheumatoid arthritis	• Julià A, González I, Fernández-Nebro A, Blanco F, Rodriguez L, González A, Cañete JD, Maymó J, Alperi-López M, Olivé A, Corominas H. A genome-wide association study identifies SLC8A3 as a susceptibility locus for ACPA-positive rheumatoid arthritis. Rheumatology. 2016 Jun 1;55(6):1106-11.
61.	VCAN	versican	-13.2621	7.42E-22	9.32E-18	• Gastric cancer<br>• Glioma	• Li W, Han F, Fu M, Wang Z. High expression of VCAN is an independent predictor of poor prognosis in gastric cancer. Journal of International Medical Research. 2020 Jan;48(1):0300060519891271.<br>• Zhu C, Mao X, Zhao H. The circ_VCAN with radioresistance contributes to the carcinogenesis of glioma by regulating microRNA-1183. Medicine. 2020 Feb;99(8).
62.	PPFIA4	protein tyrosine phosphatase; receptor type; f polypeptide (PTPRF); interacting protein (liprin); alpha 4	-12.1869	1.59E-06	0.000257	• Colon cancer	• Huang J, Yang M, Liu Z, Li X, Wang J, Fu N, Cao T, Yang X. PPFIA4 Promotes Colon Cancer Cell Proliferation and Migration by Enhancing Tumor Glycolysis. Frontiers in oncology. 2021 May 20;11:1776.
63.	FFAR3	free fatty acid receptor 3	-12.069	1.80E-08	7.82E-06	• Breast cancer	• Thirunavukkarasan M, Wang C, Rao A, Hind T, Teo YR, Siddiquee AA, Goghari MA, Kumar AP, Herr DR. Short-chain fatty acid receptors inhibit invasive phenotypes in breast cancer cells. PloS one. 2017 Oct 19;12(10):e0186334.
64.	FABP4	fatty acid binding protein 4; adipocyte	-11.6009	0.002091	0.03864	• Malignant neoplasm of breast	• Cui Y, Song M, Kim SY. Prognostic significance of fatty acid binding protein-4 in the invasive ductal carcinoma of the breast. Pathology international. 2019 Feb;69(2):68-75.
65.	MMP8	matrix metallopeptidase 8	-11.4812	4.86E-07	9.85E-05	• Melanoma	• Palavalli LH, Prickett TD, Wunderlich JR, Wei X, Burrell AS, Porter-Gill P, Davis S, Wang C, Cronin JC, Agrawal NS, Lin JC. Analysis of the matrix metalloproteinase family reveals that MMP8 is often mutated in melanoma. Nature genetics. 2009 May;41(5):518-20.
66.	LUM	lumican	-11.3033	0.0001	0.004922	• Acute pancreatitis	• Naito Z, Ishiwata T, Lu YP, Teduka K, Fujii T, Kawahara K, Sugisaki Y. Transient and ectopic expression of lumican by acinar cells in L-arginine-induced acute pancreatitis. Experimental and molecular pathology. 2003 Feb 1;74(1):33-9.

67.	ABLIM3	actin binding LIM protein family; member 3	-11.0739	1.53E-05	0.001273	• Breast cancer	• Wu JR, Zhao Y, Zhou XP, Qin X. Estrogen receptor 1 and progesterone receptor are distinct biomarkers and prognostic factors in estrogen receptor-positive breast cancer: Evidence from a bioinformatic analysis. Biomedicine & Pharmacotherapy. 2020 Jan 1;121:109647.
68.	MUCL1	mucin-like 1	-10.9229	8.38E-06	0.000833	• Multiple sclerosis	• Navarro-Barriuso J, Mansilla MJ, Quirant-Sánchez B, Ardiaca-Martínez A, Teniente-Serra A, Presas-Rodríguez S, Ten Brinke A, Ramo-Tello C, Martínez-Cáceres EM. MAP7 and MUCL1 are biomarkers of Vitamin D3-induced tolerogenic dendritic cells in multiple sclerosis patients. Frontiers in immunology. 2019 Jun 19;10:1251.
69.	CXCL13	chemokine (C-X-C motif) ligand 13	-10.7257	3.85E-06	0.000475	• Rheumatoid arthritis<br>• Breast carcinoma	• Bao YQ, Wang JP, Dai ZW, Mao YM, Wu J, Guo HS, Xia YR, Ye DQ. Increased circulating CXCL13 levels in systemic lupus erythematosus and rheumatoid arthritis: a meta-analysis. Clinical rheumatology. 2020 Jan;39(1):281-90.<br>• Razis E, Kalogeras KT, Kotsantis I, Koliou GA, Manousou K, Wirtz R, Veltrup E, Patsea H, Poulakaki N, Dionysopoulos D, Pervana S. The role of CXCL13 and CXCL9 in early breast cancer. Clinical breast cancer. 2020 Feb 1;20(1):e36-53.
70.	ANGPTL4	angiopoietin-like 4	-9.53658	2.46E-05	0.001821	• Coronary artery disease and type 2 diabetes	• Lotta LA, Stewart ID, Sharp SJ, Day FR, Burgess S, Luan JA, Bowker N, Cai L, Li C, Wittemans LB, Kerrison ND. Association of genetically enhanced lipoprotein lipase–mediated lipolysis and low-density lipoprotein cholesterol–lowering alleles with risk of coronary disease and type 2 diabetes. JAMA cardiology. 2018 Oct 1;3(10):957-66.
71.	CTGF	connective tissue growth factor	-9.05881	0.000531	0.015545	• Chronic kidney disease	• Kok HM, Falke LL, Goldschmeding R, Nguyen TQ. Targeting CTGF, EGF and PDGF pathways to prevent progression of kidney disease. Nature Reviews Nephrology. 2014 Dec;10(12):700-11.
72.	MT1A	metallothionein 1A	-8.50583	4.83E-07	9.85E-05	• Thymic epithelial tumor	• Muguruma K, Kondo K, Kishibuchi R, Tsuboi M, Soejima S, Tegshee B, Kajiura K, Kawakami Y, Kawakita N, Yoshida M, Takizawa H. MA20. 03 DNA Methylation of MT1A and NPTX2 Genes Predict Malignant Behavior of Thymic Epithelial Tumors. Journal of Thoracic Oncology. 2019 Oct 1;14(10):S331.
73.	AOX1	aldehyde oxidase 1	-8.48817	1.42E-08	6.87E-06	• Prostate cancer	• Li W, Middha M, Bicak M, Sjoberg DD, Vertosick E, Dahlin A, Häggström C, Hallmans G, Rönn AC, Stattin P, Melander O. Genome-wide scan identifies role for AOX1 in prostate cancer survival. European urology. 2018 Dec 1;74(6):710-9.
74.	HEY1	hes-related family bHLH transcription factor with YRPW motif 1	-7.9985	8.30E-06	0.000833	• Colorectal cancer<br>• salivary adenoid cystic carcinoma<br>• Glioblastoma	• Du J, Zhang L, Ma H, Wang Y, Wang P. Lidocaine suppresses cell proliferation and aerobic glycolysis by regulating circHOMER1/miR-138-5p/HEY1 Axis in colorectal cancer. Cancer Management and Research. 2020;12:5009.<br>• Xie J, Lin LS, Huang XY, Gan RH, Ding LC, Su BH, Zhao Y, Lu YG, Zheng DL. The NOTCH1-HEY1 pathway regulates self-renewal and epithelial-mesenchymal transition of salivary adenoid cystic carcinoma cells. International journal of biological sciences. 2020;16(4):598.<br>• Brun M, Jain S, Monckton EA, Godbout R. Nuclear factor I represses the notch effector HEY1 in glioblastoma. Neoplasia. 2018 Oct 1;20(10):1023-37.

75.	RCAN2	regulator of calcineurin 2	-7.82051	1.70E-07	4.35E-05	• Gastric carcinoma	• Hattori Y, Sentani K, Shinmei S, Oo HZ, Hattori T, Imai T, Sekino Y, Sakamoto N, Oue N, Niitsu H, Hinoi T. Clinicopathological significance of RCAN2 production in gastric carcinoma. Histopathology. 2019 Feb;74(3):430-42.
76.	MIR210HG	MIR210 host gene	-7.79725	1.98E-08	8.31E-06	• Cervical cancer<br>• Breast cancer	• Wang AH, Jin CH, Cui GY, Li HY, Wang Y, Yu JJ, Wang RF, Tian XY. MIR210HG promotes cell proliferation and invasion by regulating miR-503-5p/TRAF4 axis in cervical cancer. Aging (Albany NY). 2020 Feb 29;12(4):3205.<br>• Du Y, Wei N, Ma R, Jiang SH, Song D. Long Noncoding RNA MIR210HG promotes the Warburg effect and tumor growth by enhancing HIF-1α translation in triple-negative breast cancer. Frontiers in oncology. 2020;10.
77.	LIFR	leukemia inhibitory factor receptor alpha	-7.60096	5.30E-05	0.003154	• Cakut syndrome	• Kosfeld A, Brand F, Weiss AC, Kreuzer M, Goerk M, Martens H, Schubert S, Schäfer AK, Riehmer V, Hennies I, Bräsen JH. Mutations in the leukemia inhibitory factor receptor (LIFR) gene and Lifr deficiency cause urinary tract malformations. Human molecular genetics. 2017 May 1;26(9):1716-31.
78.	VLDLR	very low density lipoprotein receptor	-7.39914	3.55E-05	0.002311	• Dysequilibrium syndrome	• Kizhakkedath P, John A, Al-Gazali L, Ali BR. Degradation routes of trafficking-defective VLDLR mutants associated with Dysequilibrium syndrome. Scientific reports. 2018 Jan 25;8(1):1-2.<br>• Micalizzi A, Moroni I, Ginevrino M, Biagini T, Mazza T, Romani M, Valente EM. Very mild features of dysequilibrium syndrome associated with a novel VLDLR missense mutation. neurogenetics. 2016 Jul;17(3):191-5.
79.	TMEM26	transmembrane protein 26	-6.98172	1.17E-07	3.26E-05	• Breast cancer	• Nass N, Dittmer A, Hellwig V, Lange T, Beyer JM, Leyh B, Ignatov A, Weiβenborn C, Kirkegaard T, Lykkesfeldt AE, Kalinski T. Expression of transmembrane protein 26 (TMEM26) in breast cancer and its association with drug response. Oncotarget. 2016 Jun 21;7(25):38408.
80.	FCAMR	Fc receptor; IgA; IgM; high affinity	-6.96838	0.000219	0.008341	• Coronary Arteriosclerosis	• Ward-Caviness CK, Neas LM, Blach C, Haynes CS, LaRocque-Abramson K, Grass E, Dowdy ZE, Devlin RB, Diaz-Sanchez D, Cascio WE, Miranda ML. A genome-wide trans-ethnic interaction study links the PIGR-FCAMR locus to coronary atherosclerosis via interactions between genetic variants and residential exposure to traffic. PloS one. 2017 Mar 29;12(3):e0173880.
81.	BEX2	brain expressed X-linked 2	-6.64812	0.000565	0.016124	• Breast carcinoma	• Naderi A. Molecular functions of brain expressed X-linked 2 (BEX2) in malignancies. Experimental cell research. 2019 Mar 15;376(2):221-6.
82.	NPAS2	neuronal PAS domain protein 2	-6.51667	0.000455	0.01407	• Hepatocellular carcinoma<br>• Liver fibrosis	• Yuan P, Yang T, Mu J, Zhao J, Yang Y, Yan Z, Hou Y, Chen C, Xing J, Zhang H, Li J. Circadian clock gene NPAS2 promotes reprogramming of glucose metabolism in hepatocellular carcinoma cells. Cancer letters. 2020 Jan 28;469:498-509.<br>• Yang T, Yuan P, Yang Y, Liang N, Wang Q, Li J, Lu R, Zhang H, Mu J, Yan Z, Chang H. NPAS2 contributes to liver fibrosis by direct transcriptional activation of Hes1 in hepatic stellate cells. Molecular Therapy-Nucleic Acids. 2019 Dec 6;18:1009-22.

83.	MMP1	matrix metallopeptidase 1	-6.33929	0.00047	0.014385	• oral squamous cell carcinoma<br>• acute myeloid leukaemia	• Wang C, Mao C, Lai Y, Cai Z, Chen W. MMP1 3' UTR facilitates the proliferation and migration of human oral squamous cell carcinoma by sponging miR-188-5p to up-regulate SOX4 and CDK4. Molecular and Cellular Biochemistry. 2021 Feb;476(2):785-96.<br>• Pietrzak J, Mirowski M, Jeleń A, Świechowski R, Wodziński D, Niebudek K, Balcerczak E. Decreased MMP1 gene expression in acute myeloid leukaemia. Molecular biology reports. 2019 Apr;46(2):2293-8.
84.	ROR2	receptor tyrosine kinase-like orphan receptor 2	-5.95491	2.88E-05	0.002071	• Robinow syndrome	• Yang K, Zhu J, Tan Y, Sun X, Zhao H, Tang G, Zhang D, Qi H. Whole-exome sequencing identified compound heterozygous variants in ROR2 gene in a fetus with Robinow syndrome. Journal of clinical laboratory analysis. 2020 Feb;34(2):e23074.
85.	HPSE	heparanase	-5.83096	3.94E-14	8.26E-11	• Neoplasm Metastasis	• Masola V, Zaza G, Gambaro G, Franchi M, Onisto M. Role of heparanase in tumor progression: Molecular aspects and therapeutic options. InSeminars in cancer biology 2020 May 1 (Vol. 62, pp. 86-98). Academic Press.
86.	HES2	hes family bHLH transcription factor 2	-5.79063	0.011203	0.109057	• Neuroblastoma<br>• Lung cancer	• Zage PE, Nolo R, Fang W, Stewart J, Garcia-Manero G, Zweidler-McKay PA. Notch pathway activation induces neuroblastoma tumor cell growth arrest. Pediatric blood & cancer. 2012 May;58(5):682-9.<br>• Xu QP, Xiao RD, Xiong WM, He F, Cai L. Association between polymorphism in notch signaling pathway and lung cancer risk. Zhonghua yu Fang yi xue za zhi [Chinese Journal of Preventive Medicine]. 2018 Mar 1;52(3):243-52.
87.	SOGA3	SOGA family member 3	-5.68055	0.000122	0.005671	• NA	• NA
88.	SPAG4	sperm associated antigen 4	-5.41617	6.02E-07	0.000115	• Lung carcinoma<br>• glioblastoma	• Ji Y, Jiang J, Huang L, Feng W, Zhang Z, Jin L, Xing X. Sperm-associated antigen 4 (SPAG4) as a new cancer marker interacts with Nesprin3 to regulate cell migration in lung carcinoma. Oncology reports. 2018 Aug 1;40(2):783-92.<br>• Zhao J, Liu B, Yang JA, Tang D, Wang X, Chen Q. Human sperm-associated antigen 4 as a potential biomarker of glioblastoma progression and prognosis. Neuroreport. 2019 Apr 10;30(6):446-51.
89.	SERPINE2	serpin peptidase inhibitor; clade E (nexin; plasminogen activator inhibitor type 1); member 2	-5.32929	0.000472	0.01439	• Tumours<br>• Esophageal squamous cell carcinoma<br>• Gastric carcinoma	• Yang Y, Xin X, Fu X, Xu D. Expression pattern of human SERPINE2 in a variety of human tumors. Oncology letters. 2018 Apr 1;15(4):4523-30.<br>• Zhang J, Huang F, Gong T, Liu Z. SERPINE2 promotes esophageal squamous cell carcinoma metastasis by activating BMP4. Cancer letters. 2020 Jan 28;469:390-8.<br>• Liu J, Song S, Lin S, Zhang M, Du Y, Zhang D, Xu W, Wang H. Circ-SERPINE2 promotes the development of gastric carcinoma by sponging miR-375 and modulating YWHAZ. Cell proliferation. 2019 Jul;52(4):e12648.
90.	TUBB4A	tubulin; beta 4A class IVa	-5.29679	0.028709	0.188697	• Leukodystrophy	• Tonduti D, Aiello C, Renaldo F, Dorboz I, Saaman S, Rodriguez D, Fettah H, Elmaleh M, Biancheri R, Barresi S, Boccone L. TUBB4A-related hypomyelinating leukodystrophy: New insights from a series of 12 patients. European Journal of Paediatric Neurology. 2016 Mar 1;20(2):323-30.
91.	HS3ST1	heparan sulfate (glucosamine) 3-O-sulfotransferase 1	-5.28886	0.002077	0.038494	• Atherosclerosis	• Smits NC, Kobayashi T, Srivastava PK, Skopelja S, Ivy JA, Elwood DJ, Stan RV, Tsongalis GJ, Sellke FW, Gross PL, Cole MD. HS3ST1 genotype regulates antithrombin's inflammomodulatory tone and associates with atherosclerosis. Matrix Biology. 2017 Nov 1;63:69-90.

						• Colorectal cancer	• Wang S, Qu Y, Xia P, Chen Y, Zhu X, Zhang J, Wang G, Tian Y, Ying J, Fan Z. Transdifferentiation of tumor infiltrating innate lymphoid cells during progression of colorectal cancer. Cell research. 2020 Jul;30(7):610-22.
92.	APBA2	amyloid beta (A4) precursor protein-binding; family A; member 2	-5.28032	5.41E-05	0.003209	• Schizophrenia<br>• Dermatologic disorders	• Kirov G, Gumus D, Chen W, Norton N, Georgieva L, Sari M, O'Donovan MC, Erdogan F, Owen MJ, Ropers HH, Ullmann R. Comparative genome hybridization suggests a role for NRXN1 and APBA2 in schizophrenia. Human molecular genetics. 2008 Feb 1;17(3):458-65.<br>• Kibriya MG, Jasmine F, Parvez F, Argos M, Roy S, Paul-Brutus R, Islam T, Ahmed A, Rakibuz-Zaman M, Shinkle J, Slavkovich V. Association between genome-wide copy number variation and arsenic-induced skin lesions: a prospective study. Environmental Health. 2017 Dec;16(1):1-3.
93.	IGFBP3	insulin-like growth factor binding protein 3	-5.26849	8.62E-06	0.000839	• Colorectal carcinoma	• Hou YL, Luo P, Ji GY, Chen H. Clinical significance of serum IGFBP-3 in colorectal cancer. Journal of clinical laboratory analysis. 2019 Jul;33(6):e22912.
94.	TNFRSF21	tumor necrosis factor receptor superfamily; member 21	-5.1943	6.98E-10	6.75E-07	• Covid-19<br>• High myopia<br>• Alzheimer's disease	• Nain Z, Rana HK, Liò P, Islam SM, Summers MA, Moni MA. Pathogenetic profiling of COVID-19 and SARS-like viruses. Briefings in bioinformatics. 2021 Mar;22(2):1175-96.<br>• Pan H, Wu S, Wang J, Zhu T, Li T, Wan B, Liu B, Luo Y, Ma X, Sui R, Wang B. TNFRSF21 mutations cause high myopia. Journal of medical genetics. 2019 Oct 1;56(10):671-7.<br>• Zhang T, Yu J, Wang G, Zhang R. Amyloid precursor protein binds with TNFRSF21 to induce neural inflammation in Alzheimer's Disease. European Journal of Pharmaceutical Sciences. 2021 Feb 1;157:105598.
95.	ACHE	acetylcholinesterase (Yt blood group)	-5.17054	6.18E-05	0.003516	• Alzheimer's disease<br>• Amyloidoisis<br>• Persenile dementia	• El-Sayed NF, El-Hussieny M, Ewies EF, Fouad MA, Boulos LS. New phosphazine and phosphazide derivatives as multifunctional ligands targeting acetylcholinesterase and β-Amyloid aggregation for treatment of Alzheimer's disease. Bioorganic chemistry. 2020 Jan 1;95:103499.<br>• Fawzi SF, Menze ET, Tadros MG. Deferiprone ameliorates memory impairment in Scopolamine-treated rats: The impact of its iron-chelating effect on β-amyloid disposition. Behavioural brain research. 2020 Jan 27;378:112314.<br>• Tan EC, Johnell K, Bell JS, Garcia-Ptacek S, Fastbom J, Nordström P, Eriksdotter M. Do acetylcholinesterase inhibitors prevent or delay psychotropic prescribing in people with dementia? Analyses of the Swedish Dementia Registry. The American journal of geriatric psychiatry. 2020 Jan 1;28(1):108-17.
96.	MT1L	metallothionein 1L (gene/pseudogene)	-5.158	0.022419	0.165142	• Bladder cancer	• Ding Y, Liu N, Chen M, Xu Y, Fang S, Xiang W, Hua X, Chen G, Zhong Y, Yu H. Overexpressed pseudogene MT1L associated with tumor immune infiltrates and indicates a worse prognosis in BLCA. World Journal of Surgical Oncology. 2021 Dec;19(1):1-2.
97.	CD300E	CD300e molecule	-5.13318	8.02E-07	0.00014	• Fulminant type 1 diabetes	• Haseda F, Imagawa A, Nishikawa H, Mitsui S, Tsutsumi C, Fujisawa R, Sano H, Murase-Mishiba Y, Terasaki J, Sakaguchi S, Hanafusa T. Antibody to CMRF35-like molecule 2, CD300e a novel biomarker detected in patients with fulminant type 1 diabetes. PLoS one. 2016 Aug 11;11(8):e0160576.
98.	SLC2A5	solute carrier family 2 (facilitated glucose/fructose transporter); member 5	-5.05379	0.003581	0.054549	• Acute myeloid leukemia	• Chen WL, Wang YY, Zhao A, Xia L, Xie G, Su M, Zhao L, Liu J, Qu C, Wei R, Rajani C. Enhanced fructose utilization mediated by SLC2A5 is a unique metabolic feature of acute myeloid leukemia with therapeutic potential. Cancer cell. 2016 Nov 14;30(5):779-91.

						• Lung adenocarcinoma<br>• Alzheimer's disease	• Jiang C, Zhao H, Yang B, Sun Z, Li X, Hu X. lnc-REG3G-3-1/miR-215-3p Promotes Brain Metastasis of Lung Adenocarcinoma by Regulating Leptin and SLC2A5. Frontiers in oncology. 2020 Aug 12;10:1344.<br>• Sierksma A, Lu A, Mancuso R, Fattorelli N, Thrupp N, Salta E, Zoco J, Blum D, Buée L, De Strooper B, Fiers M. Novel Alzheimer risk genes determine the microglia response to amyloid-β but not to TAU pathology. EMBO Mol Med.
99.	DNAH17	dynein; axonemal; heavy chain 17	-5.03015	0.000403	0.01288	• Male infertility	• Zhang B, Ma H, Khan T, Ma A, Li T, Zhang H, Gao J, Zhou J, Li Y, Yu C, Bao J. A DNAH17 missense variant causes flagella destabilization and asthenozoospermia. Journal of Experimental Medicine. 2020 Feb 3;217(2).
100.	NNMT	nicotinamide N-methyltransferase	-5.02533	0.004689	0.064472	• Liver carcinoma	• Li J, You S, Zhang S, Hu Q, Wang F, Chi X, Zhao W, Xie C, Zhang C, Yu Y, Liu J. Elevated N-methyltransferase expression induced by hepatic stellate cells contributes to the metastasis of hepatocellular carcinoma via regulation of the CD44v3 isoform. Molecular oncology. 2019 Sep;13(9):1993-2009.

Table 2: Top 100 genes affected by IFN-λ4 (IFN-λ4 vs NT; M1-MDM)

S.No	Gene_symbol	Name	Foldchange	PValue	FDR	Associated Diseases	References
1.	JMJD7-PLA2G4B	JMJD7-PLA2G4B readthrough	153.8192	0.000231	0.10347	• head and neck squamous cell carcinoma	• Cheng Y, Wang Y, Li J, Chang I, Wang CY. A novel read-through transcript JMJD7-PLA2G4B regulates head and neck squamous cell carcinoma cell proliferation and survival. Oncotarget. 2017 Jan 10;8(2):1972.
2.	EPHA7	EPH receptor A7	6.091567	0.000625	0.1784	• Colorectal carcinoma<br>• small cell carcinoma of lungs	• Üçüncü M, Serilmez M, Sarı M, Bademler S, Karabulut S. The diagnostic significance of PDGF, EphA7, CCR5, and CCL5 levels in colorectal cancer. Biomolecules. 2019<br>• Peifer M, Fernández-Cuesta L, Sos ML, George J, Seidel D, Kasper LH, Plenker D, Leenders F, Sun R, Zander T, Menon R. Integrative genome analyses identify key somatic driver mutations of small-cell lung cancer. Nature genetics. 2012 Oct;44(10):1104-10.Sep;9(9):464.
3.	AMOTL2	angiomotin like 2	5.162788	0.000143	0.078194	• Malignant neoplasm of liver<br>• Glioblastoma<br>• Colorectal carcinoma	• Han H, Yang B, Wang W. Angiomotin-like 2 interacts with and negatively regulates AKT. Oncogene. 2017 Aug;36(32):4662-9.<br>• Artinian N, Cloninger C, Holmes B, Benavides-Serrato A, Bashir T, Gera J. Phosphorylation of the Hippo Pathway Component AMOTL2 by the mTORC2 Kinase Promotes YAP Signaling, Resulting in Enhanced Glioblastoma Growth and Invasiveness* . Journal of Biological Chemistry. 2015 Aug 7;290(32):19387-401.<br>• Rotoli D, Morales M, Ávila J, Maeso MD, García MD, Mobasheri A, Martín-Vasallo P. Commitment of scaffold proteins in the onco-biology of human colorectal cancer and liver metastases after oxaliplatin-based chemotherapy. International journal of molecular sciences. 2017 Apr;18(4):891.
4.	STEAP4	STEAP family member 4	5.041265	9.85E-06	0.015477	• colon tumorigenesis	• Liao Y, Zhao J, Bulek K, Tang F, Chen X, Cai G, Jia S, Fox PL, Huang E, Pizarro TT, Kalady MF. Inflammation mobilizes copper metabolism to promote colon tumorigenesis via an IL-17-STEAP4-XIAP axis. Nature communications. 2020 Feb 14;11(1):1-5.

						• Rheumatoid arthritis<br>• Obesity	• Ebe H, Matsumoto I, Kawaguchi H, Kurata I, Tanaka Y, Inoue A, Kondo Y, Tsuboi H, Sumida T. Clinical and functional significance of STEAP4-splice variant in CD14+ monocytes in patients with rheumatoid arthritis. Clinical & Experimental Immunology. 2018 Mar;191(3):338-48.<br>• Ozmen F, Ozmen MM, Gelecek S, Bilgic İ, Moran M, Sahin TT. STEAP4 and HIF-1α gene expressions in visceral and subcutaneous adipose tissue of the morbidly obese patients. Molecular immunology. 2016 May 1;73:53-9.
5.	HRASLS2	HRAS-like suppressor 2	4.929572	0.00425	0.382293	• Poland syndrome<br>• Gastric cancer	• Tang J, Qin C. Rare concurrent ocular myasthenia gravis and Graves' ophthalmopathy in a man with Poland syndrome: a case report. BMC neurology. 2020 Dec;20(1):1-5.<br>• Liang Y, Zhang C, Dai DQ. Identification of DNA methylation-regulated differentially-expressed genes and related pathways using Illumina 450K BeadChip and bioinformatic analysis in gastric cancer. Pathology-Research and Practice. 2019 Oct 1;215(10):152570.
6.	CHRNA1	cholinergic receptor; nicotinic; alpha 1 (muscle)	4.002784	0.001796	0.290233	• Primary focal hyperhidrosis<br>• Lung adenocarcinoma<br>• Congenital myasthenic syndrome	• Lin JB, Kang MQ, Huang LP, Zhuo Y, Li X, Lai FC. CHRNA1 promotes the pathogenesis of primary focal hyperhidrosis. Molecular and Cellular Neuroscience. 2021 Mar 1;111:103598.<br>• Chang PM, Yeh YC, Chen TC, Wu YC, Lu PJ, Cheng HC, Lu HJ, Chen MH, Chou TY, Huang CY. High expression of CHRNA1 is associated with reduced survival in early stage lung adenocarcinoma after complete resection. Annals of surgical oncology. 2013 Oct;20(11):3648-54.<br>• Ishii HT, Mitsuhashi H, Ishiura S. Antisense oligonucleotide-mediated exon skipping of CHRNA1 pre-mRNA as potential therapy for Congenital Myasthenic Syndromes. Biochemical and biophysical research communications. 2015 Jun 5;461(3):481-6.
7.	NGFR	nerve growth factor receptor	3.996891	0.008515	0.461936	• Prostate cancer<br>• schizophrenia	• Singh R, Karri D, Shen H, Shao J, Dasgupta S, Huang S, Edwards DP, Ittmann MM, O'Malley BW, Yi P. TRAF4-mediated ubiquitination of NGF receptor TrkA regulates prostate cancer metastasis. The Journal of clinical investigation. 2018 Jul 2;128(7):3129-43.<br>• Colantuoni C, Hyde TM, Mitkus S, Joseph A, Sartorius L, Aguirre C, Creswell J, Johnson E, Deep-Soboslay A, Herman MM, Lipska BK. Age-related changes in the expression of schizophrenia susceptibility genes in the human prefrontal cortex. Brain Structure and Function. 2008 Sep;213(1):255-71.
8.	CCL19	chemokine (C-C motif) ligand 19	3.919622	0.002292	0.304988	• B-cell malignancies	• Kozlova V, Ledererova A, Ladungova A, Peschelova H, Janovska P, Slusarczyk A, Domagala J, Kopcil P, Vakulova V, Oppelt J, Bryja V. CD20 is dispensable for B-cell receptor signaling but is required for proper actin polymerization, adhesion and migration of malignant B cells. PloS one. 2020 Mar 25;15(3):e0229170.
9.	AUTS2	autism susceptibility candidate 2	3.737385	0.000106	0.069972	• schizophrenia	• Zhang B, Xu YH, Wei SG, Zhang HB, Fu DK, Feng ZF, Guan FL, Zhu YS, Li SB. Association study identifying a new susceptibility gene (AUTS2) for schizophrenia. International journal of molecular sciences. 2014 Nov;15(11):19406-16.
10.	CXCL11	chemokine (C-X-C motif) ligand 11	3.559506	0.003024	0.335556	• Celiac disease	• Haghbin M, Rostami-Nejad M, Forouzesh F, Sadeghi A, Rostami K, Aghamohammadi E, Asadzadeh-Aghdaei H, Masotti A, Zali MR. The role of CXCR3 and its ligands CXCL10 and CXCL11 in the pathogenesis of celiac disease. Medicine. 2019 Jun;98(25).
11.	CGNL1	cingulin-like 1	3.255181	0.027146	0.60877	• Schizophrenia	• Rees E, Walters JT, Chambert KD, O'Dushlaine C, Szatkiewicz J, Richards AL, Georgieva L, Mahoney-Davies G, Legge SE, Moran JL, Genovese G. CNV analysis in a large schizophrenia sample implicates deletions at

							16p12. 1 and SLC1A1 and duplications at 1p36. 33 and CGNL1. Human molecular genetics. 2014 Mar 15;23(6):1669-76.
12.	CCL17	chemokine (C-C motif) ligand 17	3.226381	0.009208	0.464732	• Covid-19<br>• Peritoneal fibrosis<br>• osteoarthritis	• Sugiyama M, Kinoshita N, Ide S, Nomoto H, Nakamoto T, Saito S, Ishikane M, Kutsuna S, Hayakawa K, Hashimoto M, Suzuki M. Serum CCL17 level becomes a predictive marker to distinguish between mild/moderate and severe/critical disease in patients with COVID-19. Gene. 2021 Jan 15;766:145145.<br>• Chen YT, Hsu H, Lin CC, Pan SY, Liu SY, Wu CF, Tsai PZ, Liao CT, Cheng HT, Chiang WC, Chen YM. Inflammatory macrophages switch to CCL17-expressing phenotype and promote peritoneal fibrosis. The Journal of pathology. 2020 Jan;250(1):55-66.<br>• Lee MC, Saleh R, Achuthan A, Fleetwood AJ, Förster I, Hamilton JA, Cook AD. CCL17 blockade as a therapy for osteoarthritis pain and disease. Arthritis research & therapy. 2018 Dec;20(1):1-0.
13.	FLT3	fms-related tyrosine kinase 3	2.987848	0.039472	0.684373	• Acute myeloid leukemia	• Scholl S, Fleischmann M, Schnetzke U, Heidel FH. Molecular mechanisms of resistance to FLT3 inhibitors in acute myeloid leukemia: ongoing challenges and future treatments. Cells. 2020 Nov;9(11):2493.
14.	KDM4D	lysine (K)-specific demethylase 4D	2.907669	7.09E-05	0.059358	• Gastrointestinal stromal tumor<br>• hepatic fibrogenesis	• Hu F, Li H, Liu L, Xu F, Lai S, Luo X, Hu J, Yang X. Histone demethylase KDM4D promotes gastrointestinal stromal tumor progression through HIF1β/VEGFA signalling. Molecular cancer. 2018 Dec;17(1):1-2.<br>• Dong F, Jiang S, Li J, Wang Y, Zhu L, Huang Y, Jiang X, Hu X, Zhou Q, Zhang Z, Bao Z. The histone demethylase KDM4D promotes hepatic fibrogenesis by modulating Toll-like receptor 4 signaling pathway. EBioMedicine. 2019 Jan 1;39:472-83.
15.	TNFSF18	tumor necrosis factor (ligand) superfamily; member 18	2.883893	0.035638	0.664195	• African swine fever	• Zhu JJ, Ramanathan P, Bishop EA, O'Donnell V, Gladue DP, Borca MV. Mechanisms of African swine fever virus pathogenesis and immune evasion inferred from gene expression changes in infected swine macrophages. PLoS One. 2019 Nov 14;14(11):e0223955.
16.	CCR4	chemokine (C-C motif) receptor 4	2.8822	0.006177	0.430671	• Adult T-cell leukemia	• Ishitsuka K, Yurimoto S, Tsuji Y, Iwabuchi M, Takahashi T, Tobinai K. Safety and effectiveness of mogamulizumab in relapsed or refractory adult T-cell leukemia-lymphoma. European journal of haematology. 2019 May;102(5):407-15.
17.	HSPA4L	heat shock 70kDa protein 4-like	2.867243	0.003895	0.376357	• Male infertility	• Liu X, Wang X, Liu F. Decreased expression of heat shock protein A4L in spermatozoa is positively related to poor human sperm quality. Molecular reproduction and development. 2019 Apr;86(4):379-86.
18.	CCL15-CCL14	CCL15-CCL14 readthrough (NMD candidate)	2.744757	0.006517	0.434797	• NA	• NA
19.	NET1	neuroepithelial cell transforming 1	2.732961	5.03E-05	0.048669	• Malignant neoplasm of breast	• Dutertre M, Gratadou L, Dardenne E, Germann S, Samaan S, Lidereau R, Driouch K, de la Grange P, Auboeuf D. Estrogen regulation and physiopathologic significance of alternative promoters in breast cancer. Cancer research. 2010 May 1;70(9):3760-70.
20.	PDZD2	PDZ domain containing 2	2.621536	0.049534	0.710255	• Osteosarcoma	• He F, Fang L, Yin Q. miR-363 acts as a tumor suppressor in osteosarcoma cells by inhibiting PDZD2. Oncology reports. 2019 May 1;41(5):2729-38.
21.	FOLR2	folate receptor 2 (fetal)	2.619773	0.027033	0.60877	• Non-alcoholic Fatty Liver Disease	• Lake AD, Hardwick RN, Leamon CP, Low PS, Cherrington NJ. Folate receptor-beta expression as a diagnostic target in human & rodent nonalcoholic steatohepatitis. Toxicology and applied pharmacology. 2019 Apr 1;368:49-54.
22.	TMEM169	transmembrane protein 169	2.558828	0.013528	0.519888	• NA	• NA
23.	CD1B	CD1b molecule	2.55799	0.001506	0.266564	• Mycobacterium tuberculosis	• Chancellor A, Tocheva AS, Cave-Ayland C, Tezera L, White A, Juma'a R, Bridgeman JS, Tews I, Wilson S, Lissin NM, Tebruegge M. CD1b-restricted

							GEM T cell responses are modulated by Mycobacterium tuberculosis mycolic acid meromycolate chains. Proceedings of the National Academy of Sciences. 2017 Dec 19;114(51):E10956-64.
24.	COL23A1	collagen; type XXIII; alpha 1	2.528681	0.032801	0.650324	• Clear cell renal cell carcinoma	• Xu F, Chang K, Ma J, Qu Y, Xie H, Dai B, Gan H, Zhang H, Shi G, Zhu Y, Zhu Y. The oncogenic role of COL23A1 in clear cell renal cell carcinoma. Scientific reports. 2017 Aug 29;7(1):1-9.
25.	LOC100506178	NA	2.518839	0.019109	0.573884	• Renal cancer	• Hu G, Ma J, Zhang J, Chen Y, Liu H, Huang Y, Zheng J, Xu Y, Xue W, Zhai W. Hypoxia-induced lncHILAR promotes renal cancer cell invasion and metastasis via ceRNA for the miR-613/206/1-1-3p/Jagged-1/Notch/CXCR4 signaling pathway. Molecular Therapy. 2021 May 29.
26.	EDARADD	EDAR-associated death domain	2.50893	0.003157	0.342033	• Hypohidrotic ectodermal dysplasia	• Chassaing N, Cluzeau C, Bal E, Guigue P, Vincent MC, Viot G, Ginisty D, Munnich A, Smahi A, Calvas P. Mutations in EDARADD account for a small proportion of hypohidrotic ectodermal dysplasia cases. British Journal of Dermatology. 2010 May;162(5):1044-8.
27.	FBN2	fibrillin 2	2.491034	0.014877	0.526648	• Colorectal cancer<br>• congenital contractural arachnodactyly	• Hibi K, Mizukami H, Saito M, Kigawa G, Nemoto H, Sanada Y. FBN2 methylation is detected in the serum of colorectal cancer patients with hepatic metastasis. Anticancer research. 2012 Oct 1;32(10):4371-4.<br>• Wang J, Xia Y, Wang Y, Yang F, Kong X. Pathological variant of FBN2 gene identified in a pedigree affected with congenital contracture arachnodactyly. Zhonghua yi xue yi chuan xue za zhi= Zhonghua yixue yichuanxue zazhi= Chinese journal of medical genetics. 2020 May 1;37(5):497-500.
28.	RET	ret proto-oncogene	2.450571	0.004043	0.376357	• Multiple Endocrine Neoplasia Type 2b	• Makri A, Akshintala S, Derse-Anthony C, Del Rivero J, Widemann B, Stratakis CA, Glod J, Lodish M. Pheochromocytoma in children and adolescents with multiple endocrine neoplasia type 2B. The Journal of Clinical Endocrinology & Metabolism. 2019 Jan;104(1):7-12.
29.	SPTLC3	serine palmitoyltransferase; long chain base subunit 3	2.436806	0.0209	0.590608	• sepsis	• Xu Y, Ku X, Wu C, Cai C, Tang J, Yan W. Exosomal proteome analysis of human plasma to monitor sepsis progression. Biochemical and biophysical research communications. 2018 May 23;499(4):856-61.
30.	SLIT1	slit guidance ligand 1	2.421597	0.014104	0.524759	• Colorectal cancer	• Shuai W, Wu J, Chen S, Liu R, Ye Z, Kuang C, Fu X, Wang G, Li Y, Peng Q, Shi W. SUV39H2 promotes colorectal cancer proliferation and metastasis via tri-methylation of the SLIT1 promoter. Cancer letters. 2018 May 28;422:56-69.
31.	C4B	complement component 4B (Chido blood group)	2.382879	0.024924	0.601934	• Systemic lupus erythematosus	• Pereira KM, Perazzio S, Faria AG, Moreira ES, Santos VC, Grecco M, Silva NP, Andrade LE. Impact of C4, C4A and C4B gene copy number variation in the susceptibility, phenotype and progression of systemic lupus erythematosus. Advances in Rheumatology. 2019 Aug 15;59.
32.	C4B_2	complement component 4B (Chido blood group); copy 2	2.382864	0.024946	0.601934	• Inflammatory eye disease	• Wakefield D, Buckley R, Golding J, McCluskey P, Abi-Hanna D, Charlesworth J, Pussell B. Association of complement allotype C4B2 with anterior uveitis. Human immunology. 1988 Apr 1;21(4):233-7.
33.	CXCL10	chemokine (C-X-C motif) ligand 10	2.374709	0.028002	0.618466	• Lymphoma, squamous cell carcinoma and adenocarcinoma of lung<br>• Rheumatoid arthritis	• Liu M, Guo S, Stiles JK. The emerging role of CXCL10 in cancer. Oncology letters. 2011 Jul 1;2(4):583-9.<br>• Kwak HB, Ha H, Kim HN, Lee JH, Kim HS, Lee S, Kim HM, Kim JY, Kim HH, Song YW, Lee ZH. Reciprocal cross-talk between RANKL and interferon-γ–inducible protein 10 is responsible for bone-erosive experimental arthritis. Arthritis & Rheumatism. 2008 May;58(5):1332-42.
34.	DNAH3	dynein; axonemal; heavy chain 3	2.369723	0.004757	0.398554	• Lung adenocarcinoma	• Ichikawa T, Saruwatari K, Mimaki S, Sugano M, Aokage K, Kojima M, Hishida T, Fujii S, Yoshida J, Kuwata T, Ochiai A. Immunohistochemical and genetic characteristics of lung cancer mimicking organizing pneumonia. Lung Cancer. 2017 Nov 1;113:134-9.

35.	BCL2L14	BCL2-like 14 (apoptosis facilitator)	2.360904	0.002561	0.315501	• Medullary breast carcinoma	• Romero P, Benhamo V, Deniziaut G, Fuhrmann L, Berger F, Manié E, Bhalshankar J, Vacher S, Laurent C, Marangoni E, Gruel N. Medullary breast carcinoma, a triple-negative breast cancer associated with BCLG overexpression. The American journal of pathology. 2018 Oct 1;188(10):2378-91.
36.	CACNA1I	calcium channel; voltage-dependent; T type; alpha 1I subunit	2.350249	0.00711	0.43796	• Schizophrenia<br>• autism spectrum disorder	• Xie Y, Huang D, Wei L, Luo XJ. Further evidence for the genetic association between CACNA1I and schizophrenia. Hereditas. 2018 Dec;155(1):1-5.<br>• Liao X, Li Y. Genetic associations between voltage-gated calcium channels and autism spectrum disorder: a systematic review. Molecular brain. 2020 Dec;13(1):1-0.
37.	HLA-DOB	major histocompatibility complex; class II; DO beta	2.349687	0.015667	0.537947	• HBV, HCV	• Denzin LK, Khan AA, Virdis F, Wilks J, Kane M, Beilinson HA, Dikiy S, Case LK, Roopenian D, Witkowski M, Chervonsky AV. Neutralizing antibody responses to viral infections are linked to the non-classical MHC class II gene H2-Ob. Immunity. 2017 Aug 15;47(2):310-22.
38.	IL1R2	interleukin 1 receptor; type II	2.340864	0.031725	0.640841	• Ulcerative colitis	• Yoshida K, Murayama MA, Shimizu K, Tang C, Katagiri N, Matsuo K, Fukai F, Iwakura Y. IL-1R2 deficiency suppresses dextran sodium sulfate-induced colitis in mice via regulation of microbiota. Biochemical and biophysical research communications. 2018 Feb 12;496(3):934-40.
39.	IGSF3	immunoglobulin superfamily; member 3	2.334047	0.013736	0.521786	• Congenital dacryocystocele	• Foster J, Kapoor S, Diaz-Horta O, Singh A, Abad C, Rastogi A, Moharana R, Tekeli O, Walz K, Tekin M. Identification of an IGSF3 mutation in a family with congenital nasolacrimal duct obstruction. Clinical genetics. 2014 Dec;86(6):589-91.
40.	POU6F1	POU class 6 homeobox 1	2.329138	0.016444	0.5537	• Clear cell ovarian adenocarcinoma	• Suzuki N, Yoshioka N, Uekawa A, Matsumura N, Tozawa A, Koike J, Konishi I, Kiguchi K, Ishizuka B. Transcription factor POU6F1 is important for proliferation of clear cell adenocarcinoma of the ovary and is a potential new molecular target. International Journal of Gynecologic Cancer. 2010 Feb 1;20(2).
41.	AMOTL1	angiomotin like 1	2.327123	0.00597	0.428908	• Gastric oncogenesis<br>• Cervical cancer<br>• Oral squamous cell carcinoma	• Zhou Y, Zhang J, Li H, Huang T, Wong CC, Wu F, Wu M, Weng N, Liu L, Cheng AS, Yu J. AMOTL1 enhances YAP1 stability and promotes YAP1-driven gastric oncogenesis. Oncogene. 2020 May;39(22):4375-89.<br>• Ou R, Lv J, Zhang Q, Lin F, Zhu L, Huang F, Li X, Li T, Zhao L, Ren Y, Xu Y. circAMOTL1 motivates AMOTL1 expression to facilitate cervical cancer growth. Molecular Therapy-Nucleic Acids. 2020 Mar 6;19:50-60.<br>• Liu J, Yang Q, Sun H, Wang X, Saiyin H, Zhang H. The circ-AMOTL1/ENO1 Axis Implicated in the Tumorigenesis of OLP-Associated Oral Squamous Cell Carcinoma. Cancer Management and Research. 2020;12:7219.
42.	CD70	CD70 molecule	2.30225	0.004892	0.405946	• Acute myeloid leukemia	• Riether C, Pabst T, Höpner S, Bacher U, Hinterbrandner M, Banz Y, Müller R, Manz MG, Gharib WH, Francisco D, Bruggmann R. Targeting CD70 with cusatuzumab eliminates acute myeloid leukemia stem cells in patients treated with hypomethylating agents. Nature medicine. 2020 Sep;26(9):1459-67.
43.	IRF4	interferon regulatory factor 4	2.300726	0.021365	0.590608	• Chronic lymphocytic leukemia	• Asslaber D, Qi Y, Maeding N, Steiner M, Denk U, Höpner JP, Hartmann TN, Zaborsky N, Greil R, Egle A. B-cell–specific IRF4 deletion accelerates chronic lymphocytic leukemia development by enhanced tumor immune evasion. blood. 2019 Nov 14;134(20):1717-29.
44.	ANKRD33B	ankyrin repeat domain 33B	2.282559	0.009463	0.468511	• Uterine corpus endometrial carcinoma	• Ouyang D, Li R, Li Y, Zhu X. Construction of a competitive endogenous RNA network in uterine corpus endometrial carcinoma. Medical science monitor: international medical journal of experimental and clinical research. 2019;25:7998.

45.	SBK1	SH3 domain binding kinase 1	2.274134	0.043691	0.693116	• Retinoblastoma<br>• Ovarian cancer	• Feng W, Zhu R, Ma J, Song H. LncRNA ELFN1-AS1 promotes retinoblastoma growth and invasion via regulating miR-4270/SBK1 axis. Cancer Management and Research. 2021;13:1067.<br>• Li N, Zhan X. Identification of clinical trait–related lncRNA and mRNA biomarkers with weighted gene co-expression network analysis as useful tool for personalized medicine in ovarian cancer. EPMA Journal. 2019 Sep;10(3):273-90.
46.	EXOC3L4	exocyst complex component 3-like 4	2.272082	0.035588	0.664195	• Alzheimer's disease	• Miller JE, Shivakumar MK, Lee Y, Han S, Horgousluoglu E, Risacher SL, Saykin AJ, Nho K, Kim D. Rare variants in the splicing regulatory elements of EXOC3L4 are associated with brain glucose metabolism in Alzheimer’s disease. BMC medical genomics. 2018 Sep;11(3):45-52.
47.	SLC38A5	solute carrier family 38; member 5	2.260717	0.003929	0.376357	• Cancer	• Sniegowski T, Korac K, Bhutia YD, Ganapathy V. SLC6A14 and SLC38A5 Drive the Glutaminolysis and Serine–Glycine–One-Carbon Pathways in Cancer. Pharmaceuticals. 2021 Mar;14(3):216.
48.	LRRC1	leucine rich repeat containing 1	2.238902	0.024073	0.599752	• Juvenile myoclonic epilepsy<br>• breast cancer	• Suzuki T, Morita R, Sugimoto Y, Sugawara T, Bai DS, Alonso ME, Medina MT, Bailey JN, Rasmussen A, Ramos-Peek J, Cordova S. Identification and mutational analysis of candidate genes for juvenile myoclonic epilepsy on 6p11–p12: LRRC1, GCLC, KIAA0057 and CLIC5. Epilepsy research. 2002 Jul 1;50(3):265-75.<br>• Almeida LL, Sebbagh M, Bertucci F, Finetti P, Wicinski J, Marchetto S, Castellano R, Josselin E, Charafe-Jauffret E, Ginestier C, Borg JP. The SCRIB paralog LANO/LRRC1 regulates breast cancer stem cell fate through WNT/β-catenin signaling. Stem cell reports. 2018 Nov 13;11(5):1040-50.
49.	RNF43	ring finger protein 43	2.216395	0.004034	0.376357	• Colorectal and endometrial cancer	• Giannakis M, Hodis E. Jasmine Mu X, Yamauchi M, Rosenbluh J, Cibulskis K, Saksena G, Lawrence MS, Qian ZR, Nishihara R, et al. RNF43 is frequently mutated in colorectal and endometrial cancers. Nat Genet. 2014;46(12):1264-6.
50.	TNFRSF11A	tumor necrosis factor receptor superfamily; member 11a; NFKB activator	2.20101	0.001036	0.224527	• Dysosteosclerosis	• Guo L, Elcioglu NH, Karalar OK, Topkar MO, Wang Z, Sakamoto Y, Matsumoto N, Miyake N, Nishimura G, Ikegawa S. Dysosteosclerosis is also caused by TNFRSF11A mutation. Journal of human genetics. 2018 Jun;63(6):769-74.

51.	EEF1E1-BLOC1S5	EEF1E1-BLOC1S5 readthrough (NMD candidate)	-819.53	4.42E-07	0.00555	• Cystic fibrosis of lung airway and parenchyma tissues	• Kumar P, Sen C, Peters K, Frizzell RA, Biswas R. Comparative analyses of long non-coding RNA profiles in vivo in cystic fibrosis lung airway and parenchyma tissues. Respiratory research. 2019 Dec;20(1):1-1.
52.	RPS10-NUDT3	RPS10-NUDT3 readthrough	-301.102	0.00231	0.304988	• Pediatric-onset type 2 diabetes	• Miranda-Lora AL, Molina-Díaz M, Cruz M, Sánchez-Urbina R, Martínez-Rodríguez NL, López-Martínez B, Klünder-Klünder M. Genetic polymorphisms associated with pediatric-onset type 2 diabetes: A family-based transmission disequilibrium test and case-control study. Pediatric diabetes. 2019 May;20(3):239-45.
53.	FABP4	fatty acid binding protein 4; adipocyte	-20.4982	0.000384	0.155867	• Malignant neoplasm of breast	• Cui Y, Song M, Kim SY. Prognostic significance of fatty acid binding protein-4 in the invasive ductal carcinoma of the breast. Pathology international. 2019 Feb;69(2):68-75.
54.	SERPINB2	serpin peptidase inhibitor; clade B (ovalbumin); member 2	-19.2608	7.82E-06	0.014033	• Kidney injury<br>• Aging	• Sen P, Helmke A, Liao CM, Sörensen-Zender I, Rong S, Bräsen JH, Melk A, Haller H, von Vietinghoff S, Schmitt R. SerpinB2 regulates immune response in kidney injury and aging. Journal of the American Society of Nephrology. 2020 May 1;31(5):983-95.

55.	CEACAM8	carcinoembryonic antigen-related cell adhesion molecule 8	-8.93941	0.006643	0.434797	• Colorectal cancer	• Hu X, Li YQ, Ma XJ, Zhang L, Cai SJ, Peng JJ. A risk signature with inflammatory and T immune cells infiltration in colorectal cancer predicting distant metastases and efficiency of chemotherapy. Frontiers in oncology. 2019 Aug 13;9:704.
56.	BEX2	brain expressed X-linked 2	-7.61027	0.000304	0.127407	• Breast carcinoma	• Naderi A. Molecular functions of brain expressed X-linked 2 (BEX2) in malignancies. Experimental cell research. 2019 Mar 15;376(2):221-6.
57.	AKR1C1	aldo-keto reductase family 1; member C1	-6.73425	0.00021	0.097575	• Non-small cell lung cancer<br>• Cervical cancer	• Hong ZH, Chang LL, Fang-Jie YA, Yan HU, Chen-Ming ZE, Tian-Yi ZH, Tao YU, Mei-Dan YI, Ji CA, Qiao-Jun HE, Bo YA. AKR1C1 activates STAT3 to promote the metastasis of non-small cell lung cancer. Theranostics. 2018;8(3):676.<br>• Wei X, Wei Z, Li Y, Tan Z, Lin C. AKR1C1 Contributes to Cervical Cancer Progression via Regulating TWIST1 Expression. Biochemical Genetics. 2021 Apr;59(2):516-30.
58.	ANKRD1	ankyrin repeat domain 1 (cardiac muscle)	-5.96424	0.002779	0.330014	• diastolic dysfunction<br>• Pancreatic cancer	• Piroddi N, Pesce P, Scellini B, Manzini S, Ganzetti GS, Badi I, Menegollo M, Cora V, Tiso S, Cinquetti R, Monti L. Myocardial overexpression of ANKRD1 causes sinus venosus defects and progressive diastolic dysfunction. Cardiovascular research. 2020 Jul 1;116(8):1458-72.<br>• Hui B, Ji H, Xu Y, Wang J, Ma Z, Zhang C, Wang K, Zhou Y. RREB1-induced upregulation of the lncRNA AGAP2-AS1 regulates the proliferation and migration of pancreatic cancer partly through suppressing ANKRD1 and ANGPTL4. Cell death & disease. 2019 Feb 27;10(3):1-5.
59.	CXCL6	chemokine (C-X-C motif) ligand 6	-5.87435	0.008196	0.459985	• Diabetic nephropathy<br>• Esophageal squamous cell carcinoma<br>• Liver fibrosis	• Sun MY, Wang SJ, Li XQ, Shen YL, Lu JR, Tian XH, Rahman K, Zhang LJ, Nian H, Zhang H. CXCL6 promotes renal interstitial fibrosis in diabetic nephropathy by activating JAK/STAT3 signaling pathway. Frontiers in pharmacology. 2019 Mar 25;10:224.<br>• Zheng S, Shen T, Liu Q, Liu T, Tuerxun A, Zhang Q, Yang L, Han X, Lu X. CXCL6 fuels the growth and metastases of esophageal squamous cell carcinoma cells both in vitro and in vivo through upregulation of PD-L1 via activation of STAT3 pathway. Journal of Cellular Physiology. 2021 Jul;236(7):5373-86.<br>• Cai X, Li Z, Zhang Q, Qu Y, Xu M, Wan X, Lu L. CXCL 6-EGFR-induced Kupffer cells secrete TGF-β1 promoting hepatic stellate cell activation via the SMAD 2/BRD 4/C-MYC/EZH 2 pathway in liver fibrosis. Journal of cellular and molecular medicine. 2018 Oct;22(10):5050-61.
60.	TUBB4A	tubulin; beta 4A class IVa	-5.26986	0.029523	0.629087	• Leukodystrophy	• Tonduti D, Aiello C, Renaldo F, Dorboz I, Saaman S, Rodriguez D, Fettah H, Elmaleh M, Biancheri R, Barresi S, Boccone L. TUBB4A-related hypomyelinating leukodystrophy: New insights from a series of 12 patients. European Journal of Paediatric Neurology. 2016 Mar 1;20(2):323-30.
61.	ARFGEF3	ARFGEF family member 3	-4.9482	0.000732	0.187894	• Breast cancer<br>• Hermansky-Pudlak Syndrome 3	• Kim JW, Akiyama M, Park JH, Lin ML, Shimo A, Ueki T, Daigo Y, Tsunoda T, Nishidate T, Nakamura Y, Katagiri T. Activation of an estrogen/estrogen receptor signaling by BIG3 through its inhibitory effect on nuclear transport of PHB2/REA in breast cancer. Nature Precedings. 2009 Jan 30:1-.<br>• Gillingham AK, Bertram J, Begum F, Munro S. In vivo identification of GTPase interactors by mitochondrial relocalization and proximity biotinylation. Elife. 2019;8.
62.	KCP	kielin/chordin-like protein	-4.76385	6.50E-05	0.058331	• heart failure	• Ye J, Wang Z, Wang M, Xu Y, Zeng T, Ye D, Liu J, Jiang H, Lin Y, Wan J. Increased kielin/chordin-like protein levels are associated with the severity of heart failure. Clinica Chimica Acta. 2018 Nov 1;486:381-6.<br>• Soofi A, Wolf KI, Emont MP, Qi N, Martinez-Santibanez G, Grimley E, Ostwani W, Dressler GR. The kielin/chordin-like protein (KCP) attenuates

						• Obesity and metabolic syndrome	high-fat diet-induced obesity and metabolic syndrome in mice. Journal of Biological Chemistry. 2017 Jun 2;292(22):9051-62.
63.	AOX1	aldehyde oxidase 1	-4.75817	1.60E-05	0.022358	• Malignant neoplasm of prostate	• Li W, Middha M, Bicak M, Sjoberg DD, Vertosick E, Dahlin A, Häggström C, Hallmans G, Rönn AC, Stattin P, Melander O. Genome-wide scan identifies role for AOX1 in prostate cancer survival. European urology. 2018 Dec 1;74(6):710-9.
64.	CSF2	colony stimulating factor 2 (granulocyte-macrophage)	-4.27739	0.038968	0.679209	• Acute kidney injury<br>• Colorectal cancer<br>• Epithelial ovarian cancer	• Li Y, Zhai P, Zheng Y, Zhang J, Kellum JA, Peng Z. Csf2 attenuated sepsis-induced acute kidney injury by promoting alternative macrophage transition. Frontiers in Immunology. 2020 Jul 7;11:1415.<br>• Xu Z, Zhang Y, Xu M, Zheng X, Lin M, Pan J, Ye C, Deng Y, Jiang C, Lin Y, Lu X. Demethylation and overexpression of CSF2 are involved in immune response, chemotherapy resistance, and poor prognosis in colorectal cancer. OncoTargets and therapy. 2019;12:11255.<br>• Li X, Wang J, Wu W, Gao H, Liu N, Zhan G, Li L, Han L, Guo X. Myeloid-derived suppressor cells promote epithelial ovarian cancer cell stemness by inducing the CSF2/p-STAT3 signalling pathway. The FEBS journal. 2020 Dec;287(23):5218-35.
65.	RGPD1	RANBP2-like and GRIP domain containing 1	-4.09398	0.047011	0.699157	• Fragile X syndrome	• Liu XS, Wu H, Krzisch M, Wu X, Graef J, Muffat J, Hnisz D, Li CH, Yuan B, Xu C, Li Y. Rescue of fragile X syndrome neurons by DNA methylation editing of the FMR1 gene. Cell. 2018 Feb 22;172(5):979-92.
66.	ABCG2	ATP-binding cassette; sub-family G (WHITE); member 2 (Junior blood group)	-4.01534	0.009078	0.464732	• Cancer	• Toyoda Y, Takada T, Suzuki H. Inhibitors of human ABCG2: from technical background to recent updates with clinical implications. Frontiers in pharmacology. 2019 Mar 5;10:208.
67.	ZNF703	zinc finger protein 703	-3.97768	0.000102	0.069972	• Papillary thyroid carcinoma<br>• Ovarian cancer<br>• Breast cancer	• Yang X, Liu G, Zang L, Li D, Yu F, Xiang X, Li W. ZNF703 is overexpressed in papillary thyroid carcinoma tissues and mediates K1 cell proliferation. Pathology & Oncology Research. 2020 Jan;26(1):355-64.<br>• Wang S, Wang C, Hu Y, Li X, Jin S, Liu O, Gou R, Zhuang Y, Guo Q, Nie X, Zhu L. ZNF703 promotes tumor progression in ovarian cancer by interacting with HE4 and epigenetically regulating PEA15. Journal of Experimental & Clinical Cancer Research. 2020 Dec;39(1):1-9.<br>• Klæstad E, Sawicka JE, Engstrøm MJ, Ytterhus B, Valla M, Bofin AM. ZNF703 gene copy number and protein expression in breast cancer; associations with proliferation, prognosis and luminal subtypes. Breast Cancer Research and Treatment. 2021 Feb;186(1):65-77.
68.	ABLIM3	actin binding LIM protein family; member 3	-3.8122	0.008242	0.459985	• Breast cancer	• Wu JR, Zhao Y, Zhou XP, Qin X. Estrogen receptor 1 and progesterone receptor are distinct biomarkers and prognostic factors in estrogen receptor-positive breast cancer: Evidence from a bioinformatic analysis. Biomedicine & Pharmacotherapy. 2020 Jan 1;121:109647.
69.	KCNJ1	potassium channel; inwardly rectifying subfamily J; member 1	-3.80254	0.008528	0.461936	• Clear cell renal cell carcinoma<br>• Bartter syndrome type-2	• Guo Z, Liu J, Zhang L, Su B, Xing Y, He Q, Ci W, Li X, Zhou L. KCNJ1 inhibits tumor proliferation and metastasis and is a prognostic factor in clear cell renal cell carcinoma. Tumor Biology. 2015 Feb;36(2):1251-9.<br>• Zuo J, Guo W, Wang S, Lang Y, Wang S, Shi X, Zhang R, Zhao X, Han Y, Shao L. Eight novel KCNJ1 variants and parathyroid hormone overaction or resistance in 5 probands with Bartter syndrome type 2. Clinica Chimica Acta. 2020 Dec 1;511:248-54.
70.	RPLP0P2	ribosomal protein; large; P0 pseudogene 2	-3.77371	0.002414	0.312795	• Lung adenocarcinoma	• Xu G, Chen J, Pan Q, Huang K, Pan J, Zhang W, Chen J, Yu F, Zhou T, Wang Y. Long noncoding RNA expression profiles of lung adenocarcinoma ascertained by microarray analysis. PloS one. 2014 Aug 4;9(8):e104044.

71.	SERPINB7	serpin peptidase inhibitor; clade B (ovalbumin); member 7	-3.69158	0.024279	0.599752	• Nagashima-type palmoplantar keratoderma	• Hashimoto T, Teye K, Numata S, Suga Y, Hamada T, Ishii N. Detection of SERPINB 7 mutation can distinguish Nagashima-type palmoplantar keratoderma from other keratodermas with palmoplantar lesions. Clinical and experimental dermatology. 2017 Apr;42(3):342-5.
72.	MME	membrane metallo-endopeptidase	-3.60086	0.004138	0.3796	• Autosomal dominant axonal polyneuropathies<br>• spinocerebellar ataxia	• Auer-Grumbach M, Toegel S, Schabhüttl M, Weinmann D, Chiari C, Bennett DL, Beetz C, Klein D, Andersen PM, Böhme I, Fink-Puches R. Rare variants in MME, encoding metalloprotease neprilysin, are linked to late-onset autosomal-dominant axonal polyneuropathies. The American Journal of Human Genetics. 2016 Sep 1;99(3):607-23.<br>• Depondt C, Donatello S, Rai M, Wang FC, Manto M, Simonis N, Pandolfo M. MME mutation in dominant spinocerebellar ataxia with neuropathy (SCA43). Neurology Genetics. 2016 Oct 1;2(5).
73.	IL36G	interleukin 36; gamma	-3.59503	0.019942	0.582003	• Plaque psoriasis	• Traks T, Keermann M, Prans E, Karelson M, Loite U, Kõks G, Silm H, Kõks S, Kingo K. Polymorphisms in IL36G gene are associated with plaque psoriasis. BMC medical genetics. 2019 Dec;20(1):1-8.
74.	KCNJ5	potassium channel; inwardly rectifying subfamily J; member 5	-3.55492	0.017464	0.562597	• Hyperaldosteronism	• Rege J, Turcu AF, Rainey WE. Primary aldosteronism diagnostics: KCNJ5 mutations and hybrid steroid synthesis in aldosterone-producing adenomas. Gland surgery. 2020 Feb;9(1):3.
75.	CYGB	cytoglobin	-3.35625	0.034927	0.664045	• Breast cancer<br>• Non-alcoholic steatohepatitis<br>• chronic kidney disease	• Feng Y, Wu M, Li S, He X, Tang J, Peng W, Zeng B, Deng C, Ren G, Xiang T. The epigenetically downregulated factor CYGB suppresses breast cancer through inhibition of glucose metabolism. Journal of Experimental & Clinical Cancer Research. 2018 Dec;37(1):1-6.<br>• Enomoto KI, Yoshizato K, Pinzani M, Kawada N. TGF-β1-driven reduction of cytoglobin leads to oxidative DNA damage in stellate cells during non-alcoholic steatohepatitis. Journal of Hepatology. 2020;73(4):882-95.<br>• Randi EB, Vervaet B, Tsachaki M, Porto E, Vermeylen S, Lindenmeyer MT, Thuy LT, Cohen CD, Devuyst O, Kistler AD, Szabo C. The antioxidative role of cytoglobin in podocytes: implications for a role in chronic kidney disease. Antioxidants & redox signaling. 2020 Jun 1;32(16):1155-71.
76.	MMP8	matrix metallopeptidase 8	-3.34764	0.007378	0.440784	• Melanoma	• Palavalli LH, Prickett TD, Wunderlich JR, Wei X, Burrell AS, Porter-Gill P, Davis S, Wang C, Cronin JC, Agrawal NS, Lin JC. Analysis of the matrix metalloproteinase family reveals that MMP8 is often mutated in melanoma. Nature genetics. 2009 May;41(5):518-20.
77.	SPOCD1	SPOC domain containing 1	-3.24462	3.81E-05	0.043482	• Ovarian cancer<br>• Osteosarcoma	• Liu D, Yang Y, Yan A, Yang Y. SPOCD1 accelerates ovarian cancer progression and inhibits cell apoptosis via the PI3K/AKT pathway. OncoTargets and therapy. 2020;13:351.<br>• Liang J, Zhao H, Hu J, Liu Y, Li Z. SPOCD1 promotes cell proliferation and inhibits cell apoptosis in human osteosarcoma. Molecular medicine reports. 2018 Feb 1;17(2):3218-25.
78.	TM4SF19	transmembrane 4 L six family member 19	-3.19737	6.01E-06	0.012594	• Leishmaniasis<br>• Non-small cell lung cancer	• Christensen SM, Belew AT, El-Sayed NM, Tafuri WL, Silveira FT, Mosser DM. Host and parasite responses in human diffuse cutaneous leishmaniasis caused by L. amazonensis. PLoS neglected tropical diseases. 2019 Mar 7;13(3):e0007152.<br>• Chung CT, Yeh KC, Lee CH, Chen YY, Ho PJ, Chang KY, Chen CH, Lai YK, Chen CT. Molecular profiling of afatinib-resistant non-small cell lung cancer cells in vivo derived from mice. Pharmacological Research. 2020 Nov 1;161:105183.
79.	CXADR	coxsackie virus and adenovirus receptor	-3.14291	0.048543	0.70861	• Type-1 diabetes	• Vehik K, Lynch KF, Wong MC, Tian X, Ross MC, Gibbs RA, Ajami NJ, Petrosino JF, Rewers M, Toppari J, Ziegler AG. Prospective virome

						• Breast cancer	analyses in young children at increased genetic risk for type 1 diabetes. Nature medicine. 2019 Dec;25(12):1865-72.<br>• Nilchian A, Johansson J, Ghalali A, Asanin ST, Santiago A, Rosencrantz O, Sollerbrant K, Vincent CT, Sund M, Stenius U, Fuxe J. CXADR-mediated formation of an AKT inhibitory signalosome at tight junctions controls epithelial–mesenchymal plasticity in breast cancer. Cancer research. 2019 Jan 1;79(1):47-60.
80.	SHB	Src homology 2 domain containing adaptor protein B	-3.13842	0.002314	0.304988	• Acute myeloid leukemia<br>• Lung metastasis	• Jamalpour M, Li X, Cavelier L, Gustafsson K, Mostoslavsky G, Höglund M, Welsh M. Tumor SHB gene expression affects disease characteristics in human acute myeloid leukemia. Tumor Biology. 2017 Oct;39(10):1010428317720643.<br>• He Q, Li X, He L, Li Y, Betsholtz C, Welsh M. Pericyte dysfunction due to Shb gene deficiency increases B16F10 melanoma lung metastasis. International journal of cancer. 2020 Nov 1;147(9):2634-44.
81.	NT5DC2	5'-nucleotidase domain containing 2	-3.10375	0.023624	0.599752	• Hepatocellular carcinoma<br>• Non-small cell lung cancer	• Li KS, Zhu XD, Liu HD, Zhang SZ, Li XL, Xiao N, Liu XF, Xu B, Lei M, Zhang YY, Shi WK. NT5DC2 promotes tumor cell proliferation by stabilizing EGFR in hepatocellular carcinoma. Cell death & disease. 2020 May 7;11(5):1-5.<br>• Jin X, Liu X, Zhang Z, Xu L. NT5DC2 suppression restrains progression towards metastasis of non-small-cell lung cancer through regulation p53 signaling. Biochemical and Biophysical Research Communications. 2020 Dec 10;533(3):354-61.
82.	ZHX1-C8orf76	ZHX1-C8orf76 readthrough	-3.0728	0.000934	0.215637	• NA	• NA
83.	CD36	CD36 molecule (thrombospondin receptor)	-3.06598	0.000944	0.215637	• Atherosclerosis<br>• Cancer	• Silverstein RL, Febbraio M. CD36 and atherosclerosis. Current opinion in lipidology. 2000 Oct 1;11(5):483-91.<br>• Wang J, Li Y. CD36 tango in cancer: signaling pathways and functions. Theranostics. 2019;9(17):4893.
84.	VCAN	versican	-3.06328	5.78E-06	0.012594	• Gastric cancer<br>• Glioma	• Li W, Han F, Fu M, Wang Z. High expression of VCAN is an independent predictor of poor prognosis in gastric cancer. Journal of International Medical Research. 2020 Jan;48(1):0300060519891271.<br>• Zhu C, Mao X, Zhao H. The circ_VCAN with radioresistance contributes to the carcinogenesis of glioma by regulating microRNA-1183. Medicine. 2020 Feb;99(8).
85.	PDK4	pyruvate dehydrogenase kinase; isozyme 4	-3.06194	0.006116	0.429353	• Hyperglycemia	• Putra SE, Singajaya S, Thesman F, Pranoto DA, Sanjaya R, Vianney YM, Artadana IB. Aberrant PDK4 promoter methylation preceding hyperglycemia in a mouse model. Applied biochemistry and biotechnology. 2020 Mar;190(3):1023-34.
86.	HPGDS	hematopoietic prostaglandin D synthase	-2.95511	0.018292	0.568893	• Colorectal cancer	• Tippin BL, Levine AJ, Materi AM, Song WL, Keku TO, Goodman JE, Sansbury LB, Das S, Dai A, Kwong AM, Lin AM. Hematopoietic prostaglandin D synthase (HPGDS): A high stability, Val187Ile isoenzyme common among African Americans and its relationship to risk for colorectal cancer. Prostaglandins & other lipid mediators. 2012 Jan 1;97(1-2):22-8.
87.	PTPN13	protein tyrosine phosphatase; non-receptor type 13 (APO-1/CD95 (Fas)-associated phosphatase)	-2.91822	0.002263	0.304988	• Cancer	• Mcheik S, Aptecar L, Coopman P, D'hondt V, Freiss G. Dual role of the PTPN13 tyrosine phosphatase in cancer. Biomolecules. 2020 Dec;10(12):1659.
88.	FHL1	four and a half LIM domains 1	-2.90421	0.025691	0.603467	• Chikungunya virus infection	• Meertens L, Hafirassou ML, Couderc T, Bonnet-Madin L, Kril V, Kümmerer BM, Labeau A, Brugier A, Simon-Loriere E, Burlaud-Gaillard J,

						• Acute myeloid leukemia	Doyen C. FHL1 is a major host factor for chikungunya virus infection. Nature. 2019 Oct;574(7777):259-63.<br>• Fu Y, Xu M, Cui Z, Yang Z, Zhang Z, Yin X, Huang X, Zhou M, Wang X, Chen C. Genome-wide identification of FHL1 as a powerful prognostic candidate and potential therapeutic target in acute myeloid leukaemia. EBioMedicine. 2020 Feb 1;52:102664.
89.	MMP10	matrix metallopeptidase 10	-2.82436	0.003318	0.353326	• Asthma<br>• Nasopharyngeal carcinoma	• Kuo CH, Pavlidis S, Zhu J, Loza M, Baribaud F, Rowe A, Pandis I, Gibeon D, Hoda U, Sousa A, Wilson SJ. Contribution of airway eosinophils in airway wall remodeling in asthma: Role of MMP-10 and MET. Allergy. 2019 Jun;74(6):1102-12.<br>• Wang D, Luo H, Huo Z, Chen M, Han Z, Hung M, Su B, Li Y, Wang X, Guo X, Xiao H. Irradiation-induced dynamic changes of gene signatures reveal gain of metastatic ability in nasopharyngeal carcinoma. American journal of cancer research. 2019;9(3):479.
90.	TMEM45B	transmembrane protein 45B	-2.80813	0.008341	0.461753	• Prostate cancer<br>• Gastric cancer	• Luo F, Yang K, Wang YZ, Lin D. Tmem45b is a novel predictive biomarker for prostate cancer progression and metastasis. Neoplasma. 2018 Sep 19;65(5):815-21.<br>• Shen K, Yu W, Yu Y, Liu X, Cui X. Knockdown of TMEM45B inhibits cell proliferation and invasion in gastric cancer. Biomedicine & Pharmacotherapy. 2018 Aug 1;104:576-81.
91.	ACKR3	atypical chemokine receptor 3	-2.80155	0.044469	0.695291	• Autoimmune diseases	• García-Cuesta EM, Santiago CA, Vallejo-Díaz J, Juarranz Y, Rodríguez-Frade JM, Mellado M. The role of the CXCL12/CXCR4/ACKR3 axis in autoimmune diseases. Frontiers in endocrinology. 2019 Aug 27;10:585.
92.	UCHL1	ubiquitin carboxyl-terminal esterase L1 (ubiquitin thiolesterase)	-2.7898	0.013925	0.522381	• Axonal injury<br>• Breast cancer<br>• Amyotropic lateral sclerosis	• Liu H, Povysheva N, Rose ME, Mi Z, Banton JS, Li W, Chen F, Reay DP, Barrionuevo G, Zhang F, Graham SH. Role of UCHL1 in axonal injury and functional recovery after cerebral ischemia. Proceedings of the National Academy of Sciences. 2019 Mar 5;116(10):4643-50.<br>• Liu S, González-Prieto R, Zhang M, Geurink PP, Kooij R, Iyengar PV, van Dinther M, Bos E, Zhang X, Le Dévédec SE, van de Water B. Deubiquitinase Activity Profiling Identifies UCHL1 as a Candidate Oncoprotein That Promotes TGFb-Induced Breast Cancer Metastasis A C.<br>• Li R, Wang J, Xie W, Liu J, Wang C. UCHL1 from serum and CSF is a candidate biomarker for amyotrophic lateral sclerosis. Annals of Clinical and Translational Neurology. 2020 Aug;7(8):1420-8.
93.	VLDLR	very low density lipoprotein receptor	-2.69453	0.026499	0.60877	• dysequilibrium syndrome	• Kizhakkedath P, John A, Al-Gazali L, Ali BR. Degradation routes of trafficking-defective VLDLR mutants associated with Dysequilibrium syndrome. Scientific reports. 2018 Jan 25;8(1):1-2.<br>• Micalizzi A, Moroni I, Ginevrino M, Biagini T, Mazza T, Romani M, Valente EM. Very mild features of dysequilibrium syndrome associated with a novel VLDLR missense mutation. neurogenetics. 2016 Jul;17(3):191-5.
94.	DPYSL3	dihydropyrimidinase-like 3	-2.6169	0.011763	0.49942	• Neoplasm Metastasis	• Yang Y, Jiang Y, Xie D, Liu M, Song N, Zhu J, Fan J, Zhu C. Inhibition of cell-adhesion protein DPYSL3 promotes metastasis of lung cancer. Respiratory research. 2018 Dec;19(1):1-7.
95.	MET	MET proto-oncogene; receptor tyrosine kinase	-2.60267	0.007078	0.43796	• Non-small-cell carcinoma<br>• Lung adenocarcinoma	• Zhuo M, Liang Z, Yi Y, Wu N, Yang X, Zhong J, Chen X, Huang Y, Yu Z, Liu C, Zeng X. Analysis of MET kinase domain rearrangement in NSCLC. Lung Cancer. 2020 Jul 1;145:140-3.<br>• Seo JS, Ju YS, Lee WC, Shin JY, Lee JK, Bleazard T, Lee J, Jung YJ, Kim JO, Shin JY, Yu SB. The transcriptional landscape and mutational profile of lung adenocarcinoma. Genome research. 2012 Nov 1;22(11):2109-19.

96.	AFAP1L1	actin filament associated protein 1-like 1	-2.58822	0.017222	0.562597	• Spindle cell sarcoma<br>• Non-small-cell Lung carcinoma<br>• colorectal cancer	• Furu M, Kajita Y, Nagayama S, Ishibe T, Shima Y, Nishijo K, Uejima D, Takahashi R, Aoyama T, Nakayama T, Nakamura T. Identification of AFAP1L1 as a prognostic marker for spindle cell sarcomas. Oncogene. 2011 Sep;30(38):4015-25.<br>• Wang M, Han X, Sun W, Li X, Jing G, Zhang X. Actin filament-associated protein 1-like 1 mediates proliferation and survival in non-small cell lung cancer cells. Medical science monitor: international medical journal of experimental and clinical research. 2018;24:215.<br>• Takahashi R, Nagayama S, Furu M, Kajita Y, Jin Y, Kato T, Imoto S, Sakai Y, Toguchida J. AFAP 1L1, a novel associating partner with vinculin, modulates cellular morphology and motility, and promotes the progression of colorectal cancers. Cancer medicine. 2014 Aug;3(4):759-74.
97.	SDS	serine dehydratase	-2.58505	0.00086	0.211918	• Autoinflammation	• Çağdaş D, Sürücü N, Tan Ç, Kayaoğlu B, Özgül RK, Akkaya-Ulum YZ, Aydınoğlu AT, Aytaç S, Gümrük F, Balci-Hayta B, Balci-Peynircioğlu B. Autoinflammation in addition to combined immunodeficiency: SLC29A3 gene defect. Molecular immunology. 2020 May 1;121:28-37.
98.	FCMR	Fc fragment of IgM receptor	-2.54778	0.001009	0.222496	• cervical cancer<br>• Kawasaki disease	• Jiang J, Wu RH, Zhou HL, Li ZM, Kou D, Deng Z, Dong M, Chen LH. TGIF2 promotes cervical cancer metastasis by negatively regulating FCMR. European review for medical and pharmacological sciences. 2020 Jun 1;24(11):5953-62.<br>• Chang LS, Guo MM, Yan JH, Huang YH, Lo MH, Kuo HC. Low FCMR mRNA expression in leukocytes of patients with Kawasaki disease six months after disease onset. Pediatric Allergy and Immunology. 2020 Jul;31(5):554-9.
99.	ROR1-AS1	ROR1 antisense RNA 1	-2.53669	0.023882	0.599752	• Colorectal cancer<br>• Osteosarcoma	• Liao T, Maierdan SL, Lv C. ROR1-AS1 promotes tumorigenesis of colorectal cancer via targeting Wnt/β-catenin. European Review for Medical and Pharmacological Sciences. 2020 Jul 1;24(14):7561-.<br>• Wu X, Yan L, Liu Y, Shang L. LncRNA ROR1-AS1 accelerates osteosarcoma invasion and proliferation through modulating miR-504. Aging (Albany NY). 2021 Jan 15;13(1):219.
100.	GNA14	guanine nucleotide binding protein (G protein); alpha 14	-2.51375	0.004649	0.398554	• Anastomosing hemangioma<br>• Hepatocellular carcinoma	• Liau JY, Tsai JH, Lan J, Chen CC, Wang YH, Lee JC, Huang HY. GNA11 joins GNAQ and GNA14 as a recurrently mutated gene in anastomosing hemangioma. Virchows Archiv. 2020 Mar;476(3):475-81.<br>• Yu T, Lu S, Xie W. Downregulation of GNA14 in hepatocellular carcinoma indicates an unfavorable prognosis. Oncology letters. 2020 Jul 1;20(1):165-72.

Table 3: Top 100 genes affected in IFN-λ4 vs IFN-λ3 (M1-MDM)

S.No	Gene_symbol	Name	Foldchange	PValue	FDR	Associated disease	References
1.	AMOTL2	angiomotin like 2	23.94947	1.86E-10	2.92E-07	• Malignant neoplasm of liver<br>• Glioblastoma	• Han H, Yang B, Wang W. Angiomotin-like 2 interacts with and negatively regulates AKT. Oncogene. 2017 Aug;36(32):4662-9.<br>• Artinian N, Cloninger C, Holmes B, Benavides-Serrato A, Bashir T, Gera J. Phosphorylation of the Hippo Pathway Component AMOTL2 by the mTORC2 Kinase Promotes YAP Signaling, Resulting in Enhanced

						• Colorectal carcinoma	Glioblastoma Growth and Invasiveness* . Journal of Biological Chemistry. 2015 Aug 7;290(32):19387-401.<br>• Rotoli D, Morales M, Ávila J, Maeso MD, García MD, Mobasheri A, Martín-Vasallo P. Commitment of scaffold proteins in the onco-biology of human colorectal cancer and liver metastases after oxaliplatin-based chemotherapy. International journal of molecular sciences. 2017 Apr;18(4):891.
2.	CTGF	connective tissue growth factor	21.60929	2.51E-06	0.000524	• Chronic kidney disease (CKD)	• Kok HM, Falke LL, Goldschmeding R, Nguyen TQ. Targeting CTGF, EGF and PDGF pathways to prevent progression of kidney disease. Nature Reviews Nephrology. 2014 Dec;10(12):700-11.
3.	CXCL13	chemokine (C-X-C motif) ligand 13	20.62044	1.71E-08	1.13E-05	• Rheumatoid arthritis<br>• Breast carcinoma	• Bao YQ, Wang JP, Dai ZW, Mao YM, Wu J, Guo HS, Xia YR, Ye DQ. Increased circulating CXCL13 levels in systemic lupus erythematosus and rheumatoid arthritis: a meta-analysis. Clinical rheumatology. 2020 Jan;39(1):281-90.<br>• Razis E, Kalogeras KT, Kotsantis I, Koliou GA, Manousou K, Wirtz R, Veltrup E, Patsea H, Poulakaki N, Dionysopoulos D, Pervana S. The role of CXCL13 and CXCL9 in early breast cancer. Clinical breast cancer. 2020 Feb 1;20(1):e36-53.
4.	LIFR	leukemia inhibitory factor receptor alpha	15.74502	5.88E-08	3.21E-05	• Stüve-Wiedemann syndrome (SWS)<br>• Cakut syndrome	• Begam MA, Alsafi W, Bekdache GN, Chedid F, Al-Gazali L, Mirghani HM. Stuve–Wiedemann syndrome: a skeletal dysplasia characterized by bowed long bones. Ultrasound in obstetrics & gynecology. 2011 Nov;38(5):553-8<br>• Kosfeld A, Brand F, Weiss AC, Kreuzer M, Goerk M, Martens H, Schubert S, Schäfer AK, Riehmer V, Hennies I, Bräsen JH. Mutations in the leukemia inhibitory factor receptor (LIFR) gene and Lifr deficiency cause urinary tract malformations. Human molecular genetics. 2017 May 1;26(9):1716-31.
5.	SLC8A3 (NCX-3)	solute carrier family 8 (sodium/calcium exchanger); member 3	14.49298	2.14E-06	0.000463	• Seizures	• Martinez Y, N'Gouemo P. Blockade of the sodium calcium exchanger exhibits anticonvulsant activity in a pilocarpine model of acute seizures in rats. Brain research. 2010 Dec 17;1366:211-6.
6.	ACKR3 (CXCR7)	atypical chemokine receptor 3	13.79251	5.46E-06	0.000945	• Neoplasm metastasis	• Qian T, Liu Y, Dong Y, Zhang L, Dong Y, Sun Y, Sun D. CXCR7 regulates breast tumour metastasis and angiogenesis in vivo and in vitro. Molecular medicine reports. 2018 Mar 1;17(3):3633-9.
7.	LBP	lipopolysaccharide binding protein	12.88769	4.75E-05	0.00439	• Obesity	• Kellerer T, Brandl B, Büttner J, Lagkouvardos I, Hauner H, Skurk T. Impact of laparoscopic sleeve gastrectomy on gut permeability in morbidly obese subjects. Obesity surgery. 2019 Jul;29(7):2132-43.
8.	FOLR2	folate receptor 2 (fetal)	10.52442	6.28E-07	0.000193	•<br>• Non-alcoholic Fatty Liver Disease	• Lake AD, Hardwick RN, Leamon CP, Low PS, Cherrington NJ. Folate receptor-beta expression as a diagnostic target in human & rodent nonalcoholic steatohepatitis. Toxicology and applied pharmacology. 2019 Apr 1;368:49-54.
9.	CXCL12	chemokine (C-X-C motif) ligand 12	10.47408	0.000694	0.022375	• HIV infection<br>• Cancer	• Restrepo C, Gutierrez-Rivas M, Pacheco YM, García M, Blanco J, Medrano LM, Navarrete-Muñoz MA, Gutiérrez F, Miralles P, Dalmau D, Gómez JL. Genetic variation in CCR2 and CXCL12 genes impacts on CD4 restoration in patients initiating cART with advanced immunesupression. PloS one. 2019 Mar 28;14(3):e0214421.<br>• Mousavi A. CXCL12/CXCR4 signal transduction in diseases and its molecular approaches in targeted-therapy. Immunology letters. 2020 Jan 1;217:91-115.
10.	DLL4	delta-like 4 (Drosophila)	10.41865	0.005953	0.076214	• Breast cancer	• Zhou R, Wang S, Wen H, Wang M, Wu M. The bispecific antibody HB-32, blockade of both VEGF and DLL4 shows potent anti-angiogenic activity in

							vitro and anti-tumor activity in breast cancer xenograft models. Experimental cell research. 2019 Jul 15;380(2):141-8.t.
11.	RNASE2 (EDN)	ribonuclease; RNase A family; 2 (liver; eosinophil-derived neurotoxin)	10.09168	3.59E-11	9.01E-08	• Asthma	• Lee YJ, Fujisawa T, Kim CK. Biomarkers for recurrent wheezing and asthma in preschool children. Allergy, asthma & immunology research. 2019 Jan 1;11(1):16-28.
12.	FFAR3 (GPR41)	free fatty acid receptor 3	9.6308	2.82E-07	0.000101	• Liver carcinoma<br>• Asthma<br>• Eosinophilic esophagitis	• Kobayashi M, Mikami D, Uwada J, Yazawa T, Kamiyama K, Kimura H, Taniguchi T, Iwano M. A short-chain fatty acid, propionate, enhances the cytotoxic effect of cisplatin by modulating GPR41 signaling pathways in HepG2 cells. Oncotarget. 2018 Jul 31;9(59):31342.<br>• Halnes I, Baines KJ, Berthon BS, MacDonald-Wicks LK, Gibson PG, Wood LG. Soluble fibre meal challenge reduces airway inflammation and expression of GPR43 and GPR41 in asthma. Nutrients. 2017 Jan;9(1):57.<br>• Wen T, Aronow BJ, Rochman Y, Rochman M, Kiran KC, Dexheimer PJ, Putnam P, Mukkada V, Foote H, Rehn K, Darko S. Single-cell RNA sequencing identifies inflammatory tissue T cells in eosinophilic esophagitis. The Journal of clinical investigation. 2019 May 1;129(5):2014-28.
13.	LOC400043	NA	9.354088	0.000139	0.008137	• Gastric cancer	• Jafarzadeh M, Soltani BM. Long noncoding RNA LOC400043 (LINC02381) inhibits gastric cancer progression through regulating Wnt signaling pathway. Frontiers in oncology. 2020 Oct 23;10:2189.
14.	ACHE	acetylcholinesterase (Yt blood group)	9.254527	8.65E-08	4.18E-05	• Alzheimer's disease<br>• Amyloidosis<br>• Persenile dementia	• El-Sayed NF, El-Hussieny M, Ewies EF, Fouad MA, Boulos LS. New phosphazine and phosphazide derivatives as multifunctional ligands targeting acetylcholinesterase and β-Amyloid aggregation for treatment of Alzheimer's disease. Bioorganic chemistry. 2020 Jan 1;95:103499.<br>• Fawzi SF, Menze ET, Tadros MG. Deferiprone ameliorates memory impairment in Scopolamine-treated rats: The impact of its iron-chelating effect on β-amyloid disposition. Behavioural brain research. 2020 Jan 27;378:112314.<br>• Tan EC, Johnell K, Bell JS, Garcia-Ptacek S, Fastbom J, Nordström P, Eriksdotter M. Do acetylcholinesterase inhibitors prevent or delay psychotropic prescribing in people with dementia? Analyses of the Swedish Dementia Registry. The American journal of geriatric psychiatry. 2020 Jan 1;28(1):108-17.
15.	LUM	lumican	9.223119	0.000358	0.014809	• Fibrosis	• Naito Z, Ishiwata T, Lu YP, Teduka K, Fujii T, Kawahara K, Sugisaki Y. Transient and ectopic expression of lumican by acinar cells in L-arginine-induced acute pancreatitis. Experimental and molecular pathology. 2003 Feb 1;74(1):33-9.
16.	ROR2	receptor tyrosine kinase-like orphan receptor 2	8.897842	2.04E-07	8.55E-05	• Robinow syndrome	• Yang K, Zhu J, Tan Y, Sun X, Zhao H, Tang G, Zhang D, Qi H. Whole-exome sequencing identified compound heterozygous variants in ROR2 gene in a fetus with Robinow syndrome. Journal of clinical laboratory analysis. 2020 Feb;34(2):e23074.
17.	OSMR	oncostatin M receptor	8.803864	2.95E-05	0.003168	• Familial primary cutaneous amyloidosis	• Lin MW, Lee DD, Liu TT, Lin YF, Chen SY, Huang CC, Weng HY, Liu YF, Tanaka A, Arita K, Lai-Cheong J. Novel IL31RA gene mutation and ancestral OSMR mutant allele in familial primary cutaneous amyloidosis. European journal of human genetics. 2010 Jan;18(1):26-32.
18.	SLIT2	slit guidance ligand 2	8.080843	8.80E-05	0.006083	• Liver carcinoma	• Sun G, Zhang C, Feng M, Liu W, Xie H, Qin Q, Zhao E, Wan L. Methylation analysis of p16, SLIT2, SCARA5, and Runx3 genes in hepatocellular carcinoma. Medicine. 2017 Oct;96(41).

19.	GJA4	gap junction protein; alpha 4; 37kDa	7.900719	0.008197	0.092828	• Coronary artery disease	• Han Y, Xi S, Zhang X, Yan C, Yang Y, Kang J. Association of connexin 37 gene polymorphisms with risk of coronary artery disease in northern Han Chinese. Cardiology. 2008;110(4):260-5.
20.	ARNT2	aryl-hydrocarbon receptor nuclear translocator 2	7.420742	1.73E-05	0.002069	• Obesity<br>• Septo-optic dysplasia	• Turer EE, San Miguel M, Wang KW, McAlpine W, Ou F, Li X, Tang M, Zang Z, Wang J, Hayse B, Evers B. A viable hypomorphic Arnt2 mutation causes hyperphagic obesity, diabetes and hepatic steatosis. Disease models & mechanisms. 2018 Dec 1;11(12):dmm035451.<br>• Webb EA, AlMutair A, Kelberman D, Bacchelli C, Chanudet E, Lescai F, Andoniadou CL, Banyan A, Alsawaid A, Alrifai MT, Alahmesh MA. ARNT2 mutation causes hypopituitarism, post-natal microcephaly, visual and renal anomalies. Brain. 2013 Oct 1;136(10):3096-105.
21.	FCAMR	Fc receptor; IgA; IgM; high affinity	6.832784	0.000249	0.011695	• Coronary Arteriosclerosis	• Ward-Caviness CK, Neas LM, Blach C, Haynes CS, LaRocque-Abramson K, Grass E, Dowdy ZE, Devlin RB, Diaz-Sanchez D, Cascio WE, Miranda ML. A genome-wide trans-ethnic interaction study links the PIGR-FCAMR locus to coronary atherosclerosis via interactions between genetic variants and residential exposure to traffic. PloS one. 2017 Mar 29;12(3):e0173880.
22.	EPHA7	EPH receptor A7	6.811255	0.000279	0.012655	• Colorectal carcinoma<br>• Small cell carcinoma of lungs	• Üçüncü M, Serilmez M, Sarı M, Bademler S, Karabulut S. The diagnostic significance of PDGF, EphA7, CCR5, and CCL5 levels in colorectal cancer. Biomolecules. 2019<br>• Peifer M, Fernández-Cuesta L, Sos ML, George J, Seidel D, Kasper LH, Plenker D, Leenders F, Sun R, Zander T, Menon R. Integrative genome analyses identify key somatic driver mutations of small-cell lung cancer. Nature genetics. 2012 Oct;44(10):1104-10.Sep;9(9):464.
23.	PLOD2	procollagen-lysine; 2-oxoglutarate 5-dioxygenase 2	6.766755	5.79E-09	5.10E-06	• Bruck syndrome 2	• Puig-Hervás MT, Temtamy S, Aglan M, Valencia M, Martínez-Glez V, Ballesta-Martínez MJ, López-González V, Ashour AM, Amr K, Pulido V, Guillén-Navarro E. Mutations in PLOD2 cause autosomal-recessive connective tissue disorders within the Bruck syndrome—osteogenesis imperfecta phenotypic spectrum. Human mutation. 2012 Oct;33(10):1444-9.
24.	SOGA3	SOGA family member 3	6.532425	3.22E-05	0.00338	• NA	• NA
25.	ADAM23	ADAM metallopeptidase domain 23	6.493315	4.97E-05	0.004494	• Ovarian cancer<br>• Canine idiopathic epilepsy	• Ma R, Tang Z, Ye X, Cheng H, Chang X, Cui H. Low levels of ADAM23 expression in epithelial ovarian cancer are associated with poor survival. Pathology-Research and Practice. 2018 Aug 1;214(8):1115-22.<br>• Koskinen LL, Seppälä EH, Weissl J, Jokinen TS, Viitmaa R, Hänninen RL, Quignon P, Fischer A, André C, Lohi H. ADAM23 is a common risk gene for canine idiopathic epilepsy. BMC genetics. 2017 Dec;18(1):1-5.
26.	IGFBP3	insulin-like growth factor binding protein 3	6.429492	7.21E-07	0.000199	• Colorectal carcinoma	• Wang W, Wu BQ, Chen GB, Zhou Y, Li ZH, Zhang JL, Ding YL, Zhang P, Wang JQ. Meta-analysis of the association of IGFBP3 and IGF1 polymorphisms with susceptibility to colorectal cancer. Neoplasma. 2018 Sep 4;65(6):855-64.
27.	CCR4	chemokine (C-C motif) receptor 4	6.351891	7.48E-06	0.001146	• Adult T-cell leukaemia	• Ishitsuka K, Yurimoto S, Tsuji Y, Iwabuchi M, Takahashi T, Tobinai K. Safety and effectiveness of mogamulizumab in relapsed or refractory adult T-cell leukemia-lymphoma. European journal of haematology. 2019 May;102(5):407-15.
28.	PCDHGB7	protocadherin gamma subfamily B; 7	6.321596	2.03E-06	0.000458	• Breast cancer	• Hou S, Shan M, Gao C, Feng X, Yang Y, Zhang R, He Y, Zhang G, Zhang L. PCDHGB7 increases chemosensitivity to carboplatin by inhibiting hspa9 via inducing apoptosis in breast Cancer. Disease markers. 2019 Oct;2019.

						• Cervical cancer<br>• Non-Hodgkin's lymphoma	• Dong S, Lu Q, Xu P, Chen L, Duan X, Mao Z, Zhang B, Sui L, Wang Y, Yu W. Hypermethylated PCDHGB7 as a universal cancer only marker and its application in early cervical cancer screening. Clinical and Translational Medicine. 2021 Jun;11(6).<br>• Shi H, Guo J, Duff DJ, Rahmatpanah F, Chitima-Matsiga R, Al-Kuhlani M, Taylor KH, Sjahputera O, Andreski M, Wooldridge JE, Caldwell CW. Discovery of novel epigenetic markers in non-Hodgkin's lymphoma. Carcinogenesis. 2007 Jan 1;28(1):60-70.
29.	APBA2	amyloid beta (A4) precursor protein-binding; family A; member 2	6.201211	1.08E-05	0.001511	• Schizophrenia<br>• Dermatologic disorders	• Kirov G, Gumus D, Chen W, Norton N, Georgieva L, Sari M, O'Donovan MC, Erdogan F, Owen MJ, Ropers HH, Ullmann R. Comparative genome hybridization suggests a role for NRXN1 and APBA2 in schizophrenia. Human molecular genetics. 2008 Feb 1;17(3):458-65.<br>• Kibriya MG, Jasmine F, Parvez F, Argos M, Roy S, Paul-Brutus R, Islam T, Ahmed A, Rakibuz-Zaman M, Shinkle J, Slavkovich V. Association between genome-wide copy number variation and arsenic-induced skin lesions: a prospective study. Environmental Health. 2017 Dec;16(1):1-3.
30.	SERPINB7	serpin peptidase inhibitor; clade B (ovalbumin); member 7	5.968245	0.003151	0.052938	• Nagashima-type palmoplantar keratoderma	• Hashimoto T, Teye K, Numata S, Suga Y, Hamada T, Ishii N. Detection of SERPINB 7 mutation can distinguish Nagashima-type palmoplantar keratoderma from other keratodermas with palmoplantar lesions. Clinical and experimental dermatology. 2017 Apr;42(3):342-5.
31.	RET	ret proto-oncogene	5.895764	6.93E-08	3.56E-05	• Multiple Endocrine Neoplasia Type 2b	• Makri A, Akshintala S, Derse-Anthony C, Del Rivero J, Widemann B, Stratakis CA, Glod J, Lodish M. Pheochromocytoma in children and adolescents with multiple endocrine neoplasia type 2B. The Journal of Clinical Endocrinology & Metabolism. 2019 Jan;104(1):7-12.
32.	COL1A2	collagen; type I; alpha 2	5.819357	0.022908	0.165643	• Osteogenesis imperfecta type III	• Augusciak-Duma A, Witecka J, Sieroń AL, Janeczko M, Pietrzyk JJ, Ochman K, Galicka A, Borszewska-Kornacka MK, Pilch J, Jakubowska-Pietkiewicz E. Mutations in COL1A1 and COL1A2 Genes Associated with Osteogenesis Imperfecta (OI) Types I or III. Acta Biochimica Polonica. 2018 May 27;65(1):79-86.
33.	HPSE	heparanase	5.773246	5.31E-14	6.68E-10	• Neoplasm Metastasis	• Masola V, Zaza G, Gambaro G, Franchi M, Onisto M. Role of heparanase in tumor progression: Molecular aspects and therapeutic options. InSeminars in cancer biology 2020 May 1 (Vol. 62, pp. 86-98). Academic Press.
34.	ADAM19	ADAM metallopeptidase domain 19	5.705057	8.81E-05	0.006083	• COPD<br>• Colorectal Carcinoma	• Sakornsakolpat P, Prokopenko D, Lamontagne M, Reeve NF, Guyatt AL, Jackson VE, Shrine N, Qiao D, Bartz TM, Kim DK, Lee MK. Genetic landscape of chronic obstructive pulmonary disease identifies heterogeneous cell-type and phenotype associations. Nature genetics. 2019 Mar;51(3):494-505.<br>• Zhang Q, Yu L, Qin D, Huang R, Jiang X, Zou C, Tang Q, Chen Y, Wang G, Wang X, Gao X. Role of microRNA-30c targeting ADAM19 in colorectal cancer. PLoS One. 2015 Mar 23;10(3):e0120698.
35.	CGNL1	cingulin-like 1	5.473852	0.001956	0.040222	• Schizophrenia	• Rees E, Walters JT, Chambert KD, O'Dushlaine C, Szatkiewicz J, Richards AL, Georgieva L, Mahoney-Davies G, Legge SE, Moran JL, Genovese G. CNV analysis in a large schizophrenia sample implicates deletions at 16p12. 1 and SLC1A1 and duplications at 1p36. 33 and CGNL1. Human molecular genetics. 2014 Mar 15;23(6):1669-76.
36.	MT1A	metallothionein 1A	5.109762	0.000137	0.008042	• Liver carcinoma	• Wong RH, Huang CH, Yeh CB, Lee HS, Chien MH, Yang SF. Effects of metallothionein-1 genetic polymorphism and cigarette smoking on the development of hepatocellular carcinoma. Annals of surgical oncology. 2013 Jun;20(6):2088-95.
37.	TIMD4	T-cell immunoglobulin	4.982616	0.010472	0.1063	• Coronary heart disease and ischemic stroke	• Khounphinith E, Yin RX, Cao XL, Huang F, Wu JZ, Li H. TIMD4 rs6882076 SNP is associated with decreased levels of triglycerides and the risk of

		and mucin domain containing 4					coronary heart disease and ischemic stroke. International journal of medical sciences. 2019;16(6):864.
38.	FSTL1	follistatin-like 1	4.975326	7.14E-06	0.001122	• Asthma<br>• Autoimmune diseases<br>• Osteoarthritis	• Liu T, Liu Y, Miller M, Cao L, Zhao J, Wu J, Wang J, Liu L, Li S, Zou M, Xu J. Autophagy plays a role in FSTL1-induced epithelial mesenchymal transition and airway remodeling in asthma. American Journal of Physiology-Lung Cellular and Molecular Physiology. 2017 Jul 1;313(1):L27-40.<br>• Li X, Fang Y, Jiang D, Dong Y, Liu Y, Zhang S, Guo J, Qi C, Zhao C, Jiang F, Jin Y. Targeting FSTL1 for multiple fibrotic and systemic autoimmune diseases. Molecular Therapy. 2021 Jan 6;29(1):347-64.<br>• Li W, Alahdal M, Deng Z, Liu J, Zhao Z, Cheng X, Chen X, Li J, Yin J, Li Y, Wang G. Molecular functions of FSTL1 in the osteoarthritis. International immunopharmacology. 2020 Jun 1;83:106465.
39.	CXCL9	chemokine (C-X-C motif) ligand 9	4.821908	1.33E-08	9.31E-06	• Breast carcinoma	• Chen E, Qin X, Peng K, Xu X, Li W, Cheng X, Tang C, Cui Y, Wang Z, Liu T. Identification of potential therapeutic targets among CXC chemokines in breast tumor microenvironment using integrative bioinformatics analysis. Cellular Physiology and Biochemistry. 2018;45(5):1731-46.
40.	IBSP	integrin-binding sialoprotein	4.751937	0.031387	0.198408	• Oesophageal squamous cell carcinoma<br>• Colorectal cancer	• Wang M, Liu B, Li D, Wu Y, Wu X, Jiao S, Xu C, Yu S, Wang S, Yang J, Li Y. Upregulation of IBSP expression predicts poor prognosis in patients with esophageal squamous cell carcinoma. Frontiers in oncology. 2019 Oct 25;9:1117.<br>• Chen Y, Qin Y, Dai M, Liu L, Ni Y, Sun Q, Li L, Zhou Y, Qiu C, Jiang Y. IBSP, a potential recurrence biomarker, promotes the progression of colorectal cancer via Fyn/β-catenin signaling pathway. Cancer Medicine. 2021 May 13.
41.	C4B	complement component 4B (Chido blood group)	4.679674	0.000167	0.008833	• Systemic lupus erythematosus	• Pereira KM, Perazzio S, Faria AG, Moreira ES, Santos VC, Grecco M, Silva NP, Andrade LE. Impact of C4, C4A and C4B gene copy number variation in the susceptibility, phenotype and progression of systemic lupus erythematosus. Advances in Rheumatology. 2019 Aug 15;59.
42.	C4B_2	complement component 4B (Chido blood group); copy 2	4.679668	0.000167	0.008833	• Inflammatory eye disease	• Wakefield D, Buckley R, Golding J, McCluskey P, Abi-Hanna D, Charlesworth J, Pussell B. Association of complement allotype C4B2 with anterior uveitis. Human immunology. 1988 Apr 1;21(4):233-7.
43.	FAM26E	family with sequence similarity 26; member E	4.671327	0.003533	0.056607	• Spinocerebellar ataxia type-2	• Swarup V, Singh R, Singh HN, Faruq M, Srivastava AK. Identification of MicroRNAs and their target genes modulating Spinocerebellar Ataxia type-2 (SCA2) pathogenesis. Parkinsonism & Related Disorders. 2018 Jan 1;46:e5-6.
44.	PTCHD4	patched domain containing 4	4.640985	0.015915	0.135409	• Pancreatic ductal adenocarcinoma	• Huang MH, Chou YW, Li MH, Shih TE, Lin SZ, Chuang HM, Chiou TW, Su HL, Harn HJ. Epigenetic targeting DNMT1 of pancreatic ductal adenocarcinoma using interstitial control release biodegrading polymer reduced tumor growth through hedgehog pathway inhibition. Pharmacological research. 2019 Jan 1;139:50-61
45.	MUCL1	mucin-like 1	4.633763	0.002697	0.04837	• Breast Carcinoma	• Conley SJ, Bosco E, Tice D, Hollingsworth R, Herbst R, Xiao Z. Characterization of mucin-like 1 (MUCL1) in breast cancer and its novel role as a potent activator of cell proliferation.
46.	ANGPTL4	angiopoietin-like 4	4.540763	0.003634	0.057738	• Coronary artery disease	• Lotta LA, Stewart ID, Sharp SJ, Day FR, Burgess S, Luan JA, Bowker N, Cai L, Li C, Wittemans LB, Kerrison ND. Association of genetically enhanced lipoprotein lipase–mediated lipolysis and low-density lipoprotein cholesterol–lowering alleles with risk of coronary disease and type 2 diabetes. JAMA cardiology. 2018 Oct 1;3(10):957-66.
47.	NNMT	nicotinamide N-methyltransferase	4.362524	0.009481	0.100814	• Liver carcinoma	• Li J, You S, Zhang S, Hu Q, Wang F, Chi X, Zhao W, Xie C, Zhang C, Yu Y, Liu J. Elevated N-methyltransferase expression induced by hepatic

							stellate cells contributes to the metastasis of hepatocellular carcinoma via regulation of the CD44v3 isoform. Molecular oncology. 2019 Sep;13(9):1993-2009.
48.	VCAN	versican	4.329384	6.09E-09	5.10E-06	• Gastric cancer<br>• Glioma	• Li W, Han F, Fu M, Wang Z. High expression of VCAN is an independent predictor of poor prognosis in gastric cancer. Journal of International Medical Research. 2020 Jan;48(1):0300060519891271.<br>• Zhu C, Mao X, Zhao H. The circ_VCAN with radioresistance contributes to the carcinogenesis of glioma by regulating microRNA-1183. Medicine. 2020 Feb;99(8).
49.	OSR2	odd-skipped related transciption factor 2	4.273591	0.019394	0.150632	• Endometriosis	• Aghajanova L, Tatsumi K, Horcajadas JA, Zamah AM, Esteban FJ, Herndon CN, Conti M, Giudice LC. Unique transcriptome, pathways, and networks in the human endometrial fibroblast response to progesterone in endometriosis. Biology of reproduction. 2011 Apr 1;84(4):801-15.
50.	ADM	adrenomedullin	4.270044	1.84E-05	0.002146	• Sepsis	• Lemasle L, Blet A, Geven C, Cherifa M, Deniau B, Hollinger A, Fournier MC, Monnet X, Rennuit I, Darmon M, Laterre PF. Bioactive adrenomedullin, organ support therapies, and survival in the critically ill: results from the French and European outcome registry in ICU study. Critical care medicine. 2020 Jan 1;48(1):49-55

51.	EEF1E1-BLOC1S5	EEF1E1-BLOC1S5 readthrough (NMD candidate)	-702.578	6.85E-07	0.000196	• Cystic fibrosis of lung airway and parenchyma tissues	• Kumar P, Sen C, Peters K, Frizzell RA, Biswas R. Comparative analyses of long non-coding RNA profiles in vivo in cystic fibrosis lung airway and parenchyma tissues. Respiratory research. 2019 Dec;20(1):1-1.
52.	RPS10-NUDT3	RPS10-NUDT3 readthrough	-155.093	0.004791	0.067953	• Paediatric-onset type 2 diabetes	• Miranda-Lora AL, Molina-Díaz M, Cruz M, Sánchez-Urbina R, Martínez-Rodríguez NL, López-Martínez B, Klünder-Klünder M. Genetic polymorphisms associated with pediatric-onset type 2 diabetes: A family-based transmission disequilibrium test and case-control study. Pediatric diabetes. 2019 May;20(3):239-45.
53.	TBC1D3H	TBC1 domain family; member 3H	-109.147	0.005198	0.071109	• NA	• NA
54.	ATP1B2	ATPase; Na+/K+ transporting; beta 2 polypeptide	-13.4416	0.000143	0.008274	• Oesophageal squamous cell carcinoma	• Wu C, Wang Z, Song X, Feng XS, Abnet CC, He J, Hu N, Zuo XB, Tan W, Zhan Q, Hu Z. Joint analysis of three genome-wide association studies of esophageal squamous cell carcinoma in Chinese populations. Nature genetics. 2014 Sep;46(9):1001-6.
55.	GTF2H2C	GTF2H2 family member C	-12.227	0.008495	0.09482	• Coronary heart disease	• Fragou D, Pakkidi E, Aschner M, Samanidou V, Kovatsi L. Smoking and DNA methylation: Correlation of methylation with smoking behavior and association with diseases and fetus development following prenatal exposure. Food and Chemical Toxicology. 2019 Jul 1;129:312-27.
56.	CPNE6	copine VI (neuronal)	-7.51288	0.001389	0.032584	• Refractory epilepsy	• Zhu B, Zha J, Long Y, Hu X, Chen G, Wang X. Increased expression of copine VI in patients with refractory epilepsy and a rat model. Journal of the neurological sciences. 2016 Jan 15;360:30-6.
57.	CD1E	CD1e molecule	-6.44149	0.002925	0.050776	• Guillain-Barre syndrome	• Jaramillo-Valverde L, Levano KS, Villanueva I, Hidalgo M, Cornejo M, Mazzetti P, Cornejo-Olivas M, Sanchez C, Poterico JA, Valdivia-Silva J, Guio H. Guillain–Barre syndrome outbreak in Peru: Association with polymorphisms in IL-17, ICAM1, and CD1. Molecular genetics & genomic medicine. 2019 Oct;7(10):e00960.
58.	CCL24	chemokine (C-C motif) ligand 24	-5.93285	1.93E-08	1.21E-05	• Dermal and pulmonary fibrosis	• Mor A, Salto MS, Katav A, Barashi N, Edelshtein V, Manetti M, Levi Y, George J, Matucci-Cerinic M. Blockade of CCL24 with a monoclonal antibody ameliorates experimental dermal and

						• Sarcoidosis<br><br>• Hepatocellular carcinoma	pulmonary fibrosis. Annals of the rheumatic diseases. 2019 Sep 1;78(9):1260-8.<br>• Meguro A, Ishihara M, Petrek M, Yamamoto K, Takeuchi M, Mrazek F, Kolek V, Benicka A, Yamane T, Shibuya E, Yoshino A. Genetic control of CCL24, POR, and IL23R contributes to the pathogenesis of sarcoidosis. Communications biology. 2020 Aug 21;3(1):1-0.<br>• Jin L, Liu WR, Tian MX, Jiang XF, Wang H, Zhou PY, Ding ZB, Peng YF, Dai Z, Qiu SJ, Zhou J. CCL24 contributes to HCC malignancy via RhoB-VEGFA-VEGFR2 angiogenesis pathway and indicates poor prognosis. Oncotarget. 2017 Jan 17;8(3):5135.
59.	CCL1	chemokine (C-C motif) ligand 1	-5.68683	7.85E-09	6.17E-06	• Liver carcinoma	• Wiedemann GM, Röhrle N, Makeschin MC, Fesseler J, Endres S, Mayr D, Anz D. Peritumoural CCL1 and CCL22 expressing cells in hepatocellular carcinomas shape the tumour immune infiltrate. Pathology. 2019 Oct 1;51(6):586-92.
60.	GPR35	G protein-coupled receptor 35	-5.64183	0.000315	0.01384	• Hypertension	• Divorty N, Milligan G, Graham D, Nicklin SA. The Orphan Receptor GPR35 Contributes to Angiotensin II–Induced Hypertension and Cardiac Dysfunction in Mice. American journal of hypertension. 2018 Aug 3;31(9):1049-58.
61.	ENHO (Adropin)	energy homeostasis associated	-5.43628	0.012122	0.11576	• Coronary heart disease	• Zheng J, Liu M, Chen L, Yin F, Zhu X, Gou J, Zeng W, Lv Z. Association between serum adropin level and coronary artery disease: a systematic review and meta-analysis. Cardiovascular diagnosis and therapy. 2019 Feb;9(1):1.
62.	GPD1	glycerol-3-phosphate dehydrogenase 1 (soluble)	-4.70662	0.003184	0.053343	• Transient infantile hypertriglyceridemia	• Li N, Chang G, Xu Y, Ding Y, Li G, Yu T, Yao R, Li J, Shen Y, Wang X, Wang J. Biallelic mutations in GPD1 gene in a Chinese boy mainly presented with obesity, insulin resistance, fatty liver, and short stature. American Journal of Medical Genetics Part A. 2017 Dec;173(12):3189-94.
63.	CYP4F22	cytochrome P450; family 4; subfamily F; polypeptide 22	-4.64276	0.015289	0.133321	• Congenital Nonbullous Ichthyosiform Erythroderma	• Gruber R, Rainer G, Weiss A, Udvardi A, Thiele H, Eckl KM, Schupart R, Nürnberg P, Zschocke J, Schmuth M, Volc-Platzer B. Morphological alterations in two siblings with autosomal recessive congenital ichthyosis associated with CYP 4F22 mutations. British Journal of Dermatology. 2017 Apr;176(4):1068-73.
64.	MMP12	matrix metallopeptidase 12	-4.43504	0.00261	0.048185	• Pulmonary emphysema	• Doyle AD, Mukherjee M, LeSuer WE, Bittner TB, Pasha SM, Frere JJ, Neely JL, Kloeber JA, Shim KP, Ochkur SI, Ho T. Eosinophil-derived IL-13 promotes emphysema. European Respiratory Journal. 2019 May 1;53(5).
65.	OLFM2	olfactomedin 2	-4.42071	0.000348	0.01447	• Glaucoma, Open-Angle	• Holt R, Iseri SU, Wyatt AW, Bax DA, Diaz DG, Santos C, Broadgate S, Dunn R, Bruty J, Wallis Y, McMullan D. Identification and functional characterisation of genetic variants in OLFM2 in children with developmental eye disorders. Human genetics. 2017 Jan 1;136(1):119-27.
66.	CCND2	cyclin D2	-4.19314	3.33E-09	3.49E-06	• Hepatitis B virus infection	• Song CL, Ren JH, Ran LK, Li YG, Li XS, Chen X, Li WY, Huang AL, Chen J. Cyclin D2 plays a regulatory role in HBV replication. Virology. 2014 Aug 1;462:149-57.
67.	OVOL1	ovo-like zinc finger 1	-4.17588	0.001431	0.033125	• Atopic dermatitis<br><br>• Epidermal hyperplasia	• Furue K, Ito T, Tsuji G, Ulzii D, Vu YH, Kido-Nakahara M, Nakahara T, Furue M. The IL-13–OVOL 1–FLG axis in atopic dermatitis. Immunology. 2019 Dec;158(4):281-6.<br>• Sun P, Vu R, Dragan M, Haensel D, Gutierrez G, Nguyen Q, Greenberg E, Chen Z, Wu J, Atwood S, Pearlman E. OVOL1 Regulates Psoriasis-Like Skin Inflammation and Epidermal Hyperplasia. Journal of Investigative Dermatology. 2021 Jun 1;141(6):1542-52.

						• Pilomatricoma	• Ito T, Tsuji G, Ohno F, Uchi H, Nakahara T, Hashimoto-Hachiya A, Yoshida Y, Yamamoto O, Oda Y, Furue M. Activation of the OVOL1-OVOL2 axis in the hair bulb and in pilomatricoma. The American journal of pathology. 2016 Apr 1;186(4):1036-43.
68.	DIRAS2	DIRAS family; GTP-binding RAS-like 2	-3.96911	8.77E-05	0.006083	• Attention deficit hyperactive disorder	• Grünewald L, Becker N, Camphausen A, Aet OL, Lesch KP, Freudenberg F, Reif A. Expression of the ADHD candidate gene Diras2 in the brain. Journal of Neural Transmission. 2018 Jun;125(6):913-23.
69.	CLEC10A	C-type lectin domain family 10; member A	-3.953	0.0011	0.028377	• Malignant neoplasms	• Kurze AK, Buhs S, Eggert D, Oliveira-Ferrer L, Müller V, Niendorf A, Wagener C, Nollau P. Immature O-glycans recognized by the macrophage glycoreceptor CLEC10A (MGL) are induced by 4-hydroxy-tamoxifen, oxidative stress and DNA-damage in breast cancer cells. Cell Communication and Signaling. 2019 Dec;17(1):1-8.
70.	ALDH1A2	aldehyde dehydrogenase 1 family; member A2	-3.93964	1.29E-05	0.001687	• Prostatic neoplasm<br>• Degenerative polyarthritis	• Kim H, Lapointe J, Kaygusuz G, Ong DE, Li C, van de Rijn M, Brooks JD, Pollack JR. The retinoic acid synthesis gene ALDH1a2 is a candidate tumor suppressor in prostate cancer. Cancer research. 2005 Sep 15;65(18):8118-24.<br>• Shepherd C, Zhu D, Skelton AJ, Combe J, Threadgold H, Zhu L, Vincent TL, Stuart P, Reynard LN, Loughlin J. Functional characterization of the osteoarthritis genetic risk residing at ALDH1A2 identifies rs12915901 as a key target variant. Arthritis & Rheumatology. 2018 Oct;70(10):1577-87.
71.	LSR	lipolysis stimulated lipoprotein receptor	-3.84141	2.66E-07	0.000101	• Epithelial ovarian cancer<br>• Endometrial cancer<br>• Pancreatic cancer	• Takahashi Y, Serada S, Ohkawara T, Fujimoto M, Hiramatsu K, Ueda Y, Kimura T, Takemori H, Naka T. LSR promotes epithelial ovarian cancer cell survival under energy stress through the LKB1-AMPK pathway. Biochemical and Biophysical Research Communications. 2021 Jan 22;537:93-9.<br>• Konno T, Kohno T, Okada T, Shimada H, Satohisa S, Kikuchi S, Saito T, Kojima T. ASPP2 suppression promotes malignancy via LSR and YAP in human endometrial cancer. Histochemistry and cell biology. 2020 Aug;154(2):197-213.<br>• Kyuno T, Kyuno D, Kohno T, Konno T, Kikuchi S, Arimoto C, Yamaguchi H, Imamura M, Kimura Y, Kondoh M, Takemasa I. Tricellular tight junction protein LSR/angulin-1 contributes to the epithelial barrier and malignancy in human pancreatic cancer cell line. Histochemistry and cell biology. 2020 Jan;153(1):5-16.
72.	NDRG2	NDRG family member 2	-3.79439	0.000308	0.013618	• Malignant tumour of colon	• Lorentzen A, Vogel LK, Lewinsky RH, Sæbø M, Skjelbred CF, Godiksen S, Hoff G, Tveit KM, Lothe IM, Ikdahl T, Kure EH. Expression of NDRG2 is down-regulated in high-risk adenomas and colorectal carcinoma. BMC cancer. 2007 Dec;7(1):1-8.
73.	NPR1	natriuretic peptide receptor 1	-3.6717	5.61E-07	0.00018	• Hypertension	• Kumar P, Gogulamudi VR, Periasamy R, Raghavaraju G, Subramanian U, Pandey KN. Inhibition of HDAC enhances STAT acetylation, blocks NF-κB, and suppresses the renal inflammation and fibrosis in Npr1 haplotype male mice. American Journal of Physiology-Renal Physiology. 2017 Sep 1;313(3):F781-95.
74.	DPYSL3	dihydropyrimidinase-like 3	-3.55485	0.001028	0.02767	• Neoplasm Metastasis	• Yang Y, Jiang Y, Xie D, Liu M, Song N, Zhu J, Fan J, Zhu C. Inhibition of cell-adhesion protein DPYSL3 promotes metastasis of lung cancer. Respiratory research. 2018 Dec;19(1):1-7.
75.	TINAGL1	tubulointerstitial nephritis antigen-like 1	-3.55444	0.037687	0.219	• Liver carcinoma	• Sun L, Dong Z, Gu H, Guo Z, Yu Z. TINAGL1 promotes hepatocellular carcinogenesis through the activation of TGF-β signaling-medicated VEGF expression. Cancer management and research. 2019;11:767.

76.	PMF1-BGLAP	PMF1-BGLAP readthrough	-3.5388	0.013415	0.123601	• Ischemic stroke	• Malik R, Chauhan G, Traylor M, Sargurupremraj M, Okada Y, Mishra A, Rutten-Jacobs L, Giese AK, Van Der Laan SW, Gretarsdottir S, Anderson CD. Multiancestry genome-wide association study of 520,000 subjects identifies 32 loci associated with stroke and stroke subtypes. Nature genetics. 2018 Apr;50(4):524-37.
77.	CACNA1G	calcium channel; voltage-dependent; T type; alpha 1G subunit	-3.45763	0.013242	0.122761	• Spinocerebellar ataxia 42	• Barresi S, Dentici ML, Manzoni F, Bellacchio E, Agolini E, Pizzi S, Ciolfi A, Tarnopolsky M, Brady L, Garone G, Novelli A. Infantile-onset syndromic cerebellar ataxia and CACNA1G mutations. Pediatric neurology. 2020 Mar 1;104:40-5.
78.	RAB7B	RAB7B; member RAS oncogene family	-3.39873	0.001126	0.028876	• Haemolytic uremic syndrome<br>• Acute promyelocytic leukaemia	• Manzano AF, Lorenzo AF, Bocanegra V, Costantino VV, Cacciamani V, Benardon ME, Valles PG. Rab7b participation on the TLR4 (Toll-like receptor) endocytic pathway in Shiga toxin-associated Hemolytic Uremic Syndrome (HUS). Cytokine. 2019 Sep 1;121:154732.<br>• Yang M, Chen T, Han C, Li N, Wan T, Cao X. Rab7b, a novel lysosome-associated small GTPase, is involved in monocytic differentiation of human acute promyelocytic leukemia cells. Biochemical and biophysical research communications. 2004 Jun 4;318(3):792-9.
79.	ADGRA2 (TEM5)	adhesion G protein-coupled receptor A2	-3.39395	0.000704	0.022566	• Colorectal carcinoma	• Pietrzyk Ł, Wdowiak P. Serum TEM5 and TEM7 concentrations correlate with clinicopathologic features and poor prognosis of colorectal cancer patients. Advances in medical sciences. 2019 Sep 1;64(2):402-8.
80.	DOCK3	dedicator of cytokinesis 3	-3.3661	0.000557	0.019491	• Hypotonia	• Wiltrout K, Ferrer A, van de Laar I, Namekata K, Harada T, Klee EW, Zimmerman MT, Cousin MA, Kempainen JL, Babovic-Vuksanovic D, van Slegtenhorst MA. Variants in DOCK3 cause developmental delay and hypotonia. European Journal of Human Genetics. 2019 Aug;27(8):1225-34.
81.	TACSTD2	tumor-associated calcium signal transducer 2	-3.30367	0.005669	0.074814	• Familial Gelatinous Drop-Like Corneal Dystrophy	• Cabral-Macias J, Zenteno JC, Ramirez-Miranda A, Navas A, Bermudez-Magner JA, Boullosa-Graña VM, Graue-Hernandez EO, Buentello-Volante B. Familial Gelatinous Drop-Like Corneal Dystrophy Caused by a Novel Nonsense TACSTD2 Mutation. Cornea. 2016 May 9;35(7):987-90.
82.	DBN1	drebrin 1	-3.29592	0.002803	0.049403	• Leukaemia<br>• Luminal breast cancer<br>• Colon cancer	• Maki H, Yoshimi A, Shimada T, Arai S, Morita K, Kamikubo Y, Ikegawa M, Kurokawa M. Physical interaction between BAALC and DBN1 induces chemoresistance in leukemia. Experimental Hematology. 2021 Feb 1;94:31-6.<br>• Alfarsi LH, El Ansari R, Masisi BK, Parks R, Mohammed OJ, Ellis IO, Rakha EA, Green AR. Integrated analysis of key differentially expressed genes identifies DBN1 as a predictive marker of response to endocrine therapy in luminal breast cancer. Cancers. 2020 Jun;12(6):1549.<br>• Han Z, Huang H, Zhang T. Downregulation of DBN1 is related to vincristine resistance in colon cancer cells. Journal of cancer research and therapeutics. 2019 Jan 1;15(1):38.
83.	ITGA11	integrin; alpha 11	-3.27158	0.015834	0.135409	• Non-Small Cell Lung Carcinoma	• Ando T, Kage H, Matsumoto Y, Zokumasu K, Yotsumoto T, Maemura K, Amano Y, Watanabe K, Nakajima J, Nagase T, Takai D. Integrin α11 in non–small cell lung cancer is associated with tumor progression and postoperative recurrence. Cancer science. 2020 Jan;111(1):200-8.

84.	TSPAN32	tetraspanin 32	-3.26774	0.017728	0.143732	• Systemic lupus erythematosus<br>• Multiple sclerosis	• Fagone P, Mangano K, Di Marco R, Reyes-Castillo Z, Muñoz-Valle JF, Nicoletti F. Altered Expression of TSPAN32 during B Cell Activation and Systemic Lupus Erythematosus. Genes. 2021 Jun;12(6):931.<br>• Basile MS, Mazzon E, Mangano K, Pennisi M, Petralia MC, Lombardo SD, Nicoletti F, Fagone P, Cavalli E. Impaired expression of Tetraspanin 32 (TSPAN32) in memory T cells of patients with multiple sclerosis. Brain sciences. 2020 Jan;10(1):52.
85.	LOC100506585	NA	-3.26479	0.000886	0.02531	• Non-Small Cell Lung Carcinoma	• Yu H, Xu Q, Liu F, Ye X, Wang J, Meng X. Identification and validation of long noncoding RNA biomarkers in human non–small-cell lung carcinomas. Journal of thoracic oncology. 2015 Apr 1;10(4):645-54.
86.	OTOF	otoferlin	-3.26297	4.41E-05	0.004235	• Deafness (auditory neuropathy)	• Wu CC, Hsu CJ, Huang FL, Lin YH, Lin YH, Liu TC, Wu CM. Timing of cochlear implantation in auditory neuropathy patients with OTOF mutations: Our experience with 10 patients. Clinical Otolaryngology. 2018 Feb;43(1):352-7.
87.	RASAL1	RAS protein activator like 1 (GAP1 like)	-3.24669	0.049943	0.253899	• Hereditary breast cancer<br>• Colon cancer<br>• Liver fibrosis	• Isidori F, Bozzarelli I, Ferrari S, Godino L, Innella G, Turchetti D, Bonora E. RASAL1 and ROS1 Gene Variants in Hereditary Breast Cancer. Cancers. 2020 Sep;12(9):2539.<br>• Wang G, Li Z, Li X, Zhang C, Peng L. RASAL1 induces to downregulate the SCD1, leading to suppression of cell proliferation in colon cancer via LXRα/SREBP1c pathway. Biological research. 2019 Dec;52(1):1-1.<br>• Takata A, Otsuka M, Kishikawa T, Yamagami M, Ishibashi R, Sekiba K, Suzuki T, Ohno M, Yamashita Y, Abe T, Masuzaki R. RASAL1 is a potent regulator of hepatic stellate cell activity and liver fibrosis. Oncotarget. 2017 Sep 12;8(39):64840.
88.	GP1BA	glycoprotein Ib (platelet); alpha polypeptide	-3.22184	0.044186	0.238626	• Bernard-Soulier syndrome	• Özdemir ZC, Kar YD, Ceylaner S, Bör Ö. A novel mutation in the GP1BA gene in Bernard–Soulier syndrome. Blood Coagulation & Fibrinolysis. 2020 Jan 1;31(1):83-6.
89.	CRABP2	cellular retinoic acid binding protein 2	-3.1874	0.001878	0.039107	• Breast cancer<br>• Lung cancer<br>• Hepatocellular carcinoma	• Feng X, Zhang M, Wang B, Zhou C, Mu Y, Li J, Liu X, Wang Y, Song Z, Liu P. CRABP2 regulates invasion and metastasis of breast cancer through hippo pathway dependent on ER status. Journal of Experimental & Clinical Cancer Research. 2019 Dec;38(1):1-8.<br>• Wu JI, Lin YP, Tseng CW, Chen HJ, Wang LH. Crabp2 promotes metastasis of lung cancer cells via HuR and integrin β1/FAK/ERK signaling. Scientific reports. 2019 Jan 29;9(1):1-4.<br>• Chen Q, Tan L, Jin Z, Liu Y, Zhang Z. Downregulation of CRABP2 inhibit the tumorigenesis of hepatocellular carcinoma in vivo and in vitro. BioMed Research International. 2020 Jun 24;2020.
90.	CD300LB	CD300 molecule-like family member b	-3.18396	0.023592	0.168452	• Sepsis	• Voss OH, Murakami Y, Pena MY, Lee HN, Tian L, Margulies DH, Street JM, Yuen PS, Qi CF, Krzewski K, Coligan JE. Lipopolysaccharide-induced binding of receptor CD300b to Toll-like receptor 4 alters signaling to drive lethal cytokine responses that enhance septic shock. Immunity. 2016 Jun 21;44(6):1365.
91.	GIPC3	GIPC PDZ domain containing family; member 3	-3.17796	0.000169	0.008911	• Loss of hearing<br>• Melanoma	• Charizopoulou N, Lelli A, Schraders M, Ray K, Hildebrand MS, Ramesh A, Srisailapathy CS, Oostrik J, Admiraal RJ, Neely HR, Latoche JR. Gipc3 mutations associated with audiogenic seizures and sensorineural hearing loss in mouse and human. Nature communications. 2011 Feb 15;2(1):1-2.<br>• Patmanathan SN, Tong BT, Teo JH, Ting YZ, Tan NS, Sim SH, Ta YC, Woo WM. A PDZ Protein GIPC3 Positively Modulates Hedgehog

							Signaling and Melanoma Growth. Journal of Investigative Dermatology. 2021 Jul 2.
92.	SLC51B	solute carrier family 51; beta subunit	-3.17781	0.045616	0.242588	• Cholestasis	• Sultan M, Rao A, Elpeleg O, Vaz FM, Abu-Libdeh B, Karpen SJ, Dawson PA. Organic solute transporter-β (SLC51B) deficiency in two brothers with congenital diarrhea and features of cholestasis. Hepatology. 2018 Aug;68(2):590-8.
93.	SNAI3	snail family zinc finger 3	-3.17107	8.88E-05	0.006096	• Hepatocellular carcinoma	• Li Y, Guo D, Lu G, Chowdhury AT, Zhang D, Ren M, Chen Y, Wang R, He S. LncRNA SNAI3-AS1 promotes PEG10-mediated proliferation and metastasis via decoying of miR-27a-3p and miR-34a-5p in hepatocellular carcinoma. Cell Death & Disease. 2020 Aug 11;11(8):1-1.
94.	TIE1	tyrosine kinase with immunoglobulin-like and EGF-like domains 1	-3.15196	9.07E-05	0.006196	• Liver fibrogenesis<br>• Breast cancer	• Xu M, Xu HH, Lin Y, Sun X, Wang LJ, Fang ZP, Su XH, Liang XJ, Hu Y, Liu ZM, Cheng Y. LECT2, a ligand for Tie1, plays a crucial role in liver fibrogenesis. Cell. 2019 Sep 5;178(6):1478-92.<br>• Tiainen L, Korhonen EA, Leppänen VM, Luukkaala T, Hämäläinen M, Tanner M, Lahdenperä O, Vihinen P, Jukkola A, Karihtala P, Aho S. High baseline Tie1 level predicts poor survival in metastatic breast cancer. BMC cancer. 2019 Dec;19(1):1-0.
95.	DHRS9	dehydrogenase/reductase (SDR family) member 9	-3.14989	0.002854	0.0501	• Pancreatic cancer<br>• Oral squamous cell carcinoma<br>• Rheumatoid arthritis	• Li HB, Zhou J, Zhao F, Yu J, Xu L. Prognostic Impact of DHRS9 Overexpression in Pancreatic Cancer. Cancer Management and Research. 2020;12:5997.<br>• Shimomura H, Sasahira T, Nakashima C, Shimomura-Kurihara M, Kirita T. Downregulation of DHRS9 is associated with poor prognosis in oral squamous cell carcinoma. Pathology. 2018 Oct 1;50(6):642-7.<br>• Zhang D, Li Z, Zhang R, Yang X, Zhang D, Li Q, Wang C, Yang X, Xiong Y. Identification of differentially expressed and methylated genes associated with rheumatoid arthritis based on network. Autoimmunity. 2020 Aug 17;53(6):303-13.
96.	AQP3	aquaporin 3 (Gill blood group)	-3.11088	6.87E-05	0.005431	• Oral lichen planus<br>• Lung cancer<br>• Pancreatic ductal adeno carcinoma	• Agha-Hosseini F, Barati H, Moosavi MS. Aquaporin3 (AQP3) expression in oral epithelium in oral lichen planus. Experimental and molecular pathology. 2020 Aug 1;115:104441.<br>• Liu C, Liu L, Zhang Y, Jing H. Molecular mechanism of AQP3 in regulating differentiation and apoptosis of lung cancer stem cells through Wnt/GSK-3β/β-Catenin pathway. JBUON. 2020 Jul 1;25:1714-20<br>• Zou W, Yang Z, Li D, Liu Z, Zou Q, Yuan Y. AQP1 and AQP3 expression are associated with severe symptoms and poor-prognosis of the pancreatic ductal adenocarcinoma. Applied immunohistochemistry & molecular morphology. 2019 Jan 1;27(1):40-7.
97.	RASGRF1	Ras protein-specific guanine nucleotide-releasing factor 1	-3.08959	0.000536	0.019029	• Myopia	• Kunceviciene E, Sriubiene M, Liutkeviciene R, Miceikiene IT, Smalinskiene A. Heritability of myopia and its relation with GDJ2 and RASGRF1 genes in Lithuania. BMC ophthalmology. 2018 Dec;18(1):1-6.
98.	SORBS1	sorbin and SH3 domain containing 1	-3.06725	0.012602	0.119161	• Colorectal cancer<br>• Diabetes	• Cho WC, Jang JE, Kim KH, Yoo BC, Ku JL. SORBS1 serves a metastatic role via suppression of AHNAK in colorectal cancer cell lines. International journal of oncology. 2020 May 1;56(5):1140-51.<br>• Chang TJ, Wang WC, Hsiung CA, He CT, Lin MW, Sheu WH, Chang YC, Quertermous T, Chen YD, Rotter JI, Chuang LM. Genetic variation of SORBS1 gene is associated with glucose homeostasis

							and age at onset of diabetes: A SAPPHIRe Cohort Study. Scientific reports. 2018 Jul 12;8(1):1-0.
99.	ST3GAL6-AS1	ST3GAL6 antisense RNA 1	-2.99635	0.00521	0.071163	• Myeloma<br>• Colorectal cancer	• Ronchetti D, Todoerti K, Vinci C, Favasuli V, Agnelli L, Manzoni M, Pelizzoni F, Chiaramonte R, Platonova N, Giuliani N, Tassone P. Expression pattern and biological significance of the lncRNA ST3GAL6-AS1 in multiple myeloma. Cancers. 2020 Apr;12(4):782.<br>• Hu J, Shan Y, Ma J, Pan Y, Zhou H, Jiang L, Jia L. LncRNA ST3Gal6-AS1/ST3Gal6 axis mediates colorectal cancer progression by regulating α-2, 3 sialylation via PI3K/Akt signaling. International journal of cancer. 2019 Jul 15;145(2):450-60.
100.	FAM135B	family with sequence similarity 135; member B	-2.95951	0.001031	0.02767	• Oesophageal carcinoma	• Bi L, Wang H, Tian Y. Silencing FAM135B enhances radiosensitivity of esophageal carcinoma cell. Gene. 2021 Mar 10;772:145358.

Table 4: Top 100 genes affected by IFN-λ3 (IFN-λ3 vs NT; M2-MDM)

S.No	Gene_symbol	Name	Foldchange	PValue	FDR	Associated Disease	Reference
1.	PPBP	pro-platelet basic protein (chemokine (C-X-C motif) ligand 7)	280.9127	3.30E-17	5.92E-14	• Malignant neoplasm of lung,	• Du Q, Li E, Liu Y, Xie W, Huang C, Song J, Zhang W, Zheng Y, Wang H, Wang Q. CTAPIII/CXCL 7: a novel biomarker for early diagnosis of lung cancer. Cancer medicine. 2018 Feb;7(2):325-35.
2.	GTF2H2C	GTF2H2 family member C	133.659	7.25E-05	0.000982	• Coronary heart disease	• Chhabra D, Sharma S, Kho AT, Gaedigk R, Vyhlidal CA, Leeder JS, Morrow J, Carey VJ, Weiss ST, Tantisira KG, DeMeo DL. Fetal lung and placental methylation is associated with in utero nicotine exposure. Epigenetics. 2014 Nov 2;9(11):1473-84..
3.	RGS18	regulator of G-protein signaling 18	47.45056	1.27E-10	2.34E-08	• Stomach carcinoma	• Su C, Li H, Peng Z, Ke D, Fu H, Zheng X. Identification of plasma RGS18 and PPBP mRNAs as potential biomarkers for gastric cancer using transcriptome arrays. Oncology letters. 2019 Jan 1;17(1):247-55.
4.	SPATA12	spermatogenesis associated 12	30.027	1.98E-07	9.10E-06	• Tumor progression	• Liu Z, Lin Y, Liu X, Yu W, Zhang Y, Li D. Experimental study of inhibition of tumor cell proliferation by a novel gene SPATA12. Zhong nan da xue xue bao. Yi xue ban= Journal of Central South University. Medical sciences. 2012 Mar 1;37(3):222-7.
5.	ALK	anaplastic lymphoma receptor tyrosine kinase	26.89163	0.000575	0.004879	• Neuroblastoma,<br>• Adenocarcinoma of lung	• Sano R, Krytska K, Larmour CE, Raman P, Martinez D, Ligon GF, Lillquist JS, Cucchi U, Orsini P, Rizzi S, Pawel BR. An antibody-drug conjugate directed to the ALK receptor demonstrates efficacy in preclinical models of neuroblastoma. Science translational medicine. 2019 Mar 13;11(483).<br>• Deng H, Li B, Li L, Peng J, Lv T, Liu Y, Ding C. Clinical observation of crizotinib in the treatment of ALK-positive advanced non-small cell lung cancer. Pathology-Research and Practice. 2019 Dec 1;215(12):152695.
6.	TMEM37	transmembrane protein 37	22.69274	4.84E-10	6.99E-08	• Type-2 diabetes<br>• Colon cancer	• Solimena M, Schulte AM, Marselli L, Ehehalt F, Richter D, Kleeberg M, Mziaut H, Knoch KP, Parnis J, Bugliani M, Siddiq A. Systems biology of the IMIDIA biobank from organ donors and pancreatectomised patients defines a novel transcriptomic signature of islets from individuals with type 2 diabetes. Diabetologia. 2018 Mar;61(3):641-57.

							• Li C, Shen Z, Zhou Y, Yu W. Independent prognostic genes and mechanism investigation for colon cancer. Biological Research. 2018;51.
7.	F13A1	coagulation factor XIII; A1 polypeptide	15.9446	1.95E-06	5.70E-05	• Hereditary Factor XIII Deficiency	• Jia S, He Y, Lu M, Liao N, Lei Y, Lauriane N, Liang K, Wei H. Identification of novel pathogenic F13A1 mutation and novel NBEAL2 gene missense mutation in a pedigree with hereditary congenital factor XIII deficiency. Gene. 2019 Jun 20;702:143-7.
8.	ASIC1	acid sensing (proton gated) ion channel 1	15.75164	4.28E-05	0.000641	• Stomach carcinoma	• Heydari-Mehrabadi A, Tamandani DM, Baranzehi T, Hemati S. Analysis of polymorphism and expression profile of ASIC1 and IL-6 genes in patients with gastric cancer. Asian Pacific journal of cancer prevention: APJCP. 2018;19(12):3451.
9.	CXCL5	chemokine (C-X-C motif) ligand 5	15.66281	7.38E-14	3.76E-11	• Pneumonia	• Traber KE, Hilliard KL, Allen E, Wasserman GA, Yamamoto K, Jones MR, Mizgerd JP, Quinton LJ. Induction of STAT3-dependent CXCL5 expression and neutrophil recruitment by oncostatin-M during pneumonia. American journal of respiratory cell and molecular biology. 2015 Oct;53(4):479-88.
10.	FAXDC2	fatty acid hydroxylase domain containing 2	13.9745	7.75E-12	2.32E-09	• Parkinson's Disease	• Santiago JA, Bottero V, Potashkin JA. Evaluation of RNA blood biomarkers in the Parkinson's disease biomarkers program. Frontiers in aging neuroscience. 2018 May 29;10:157.
11.	E2F2	E2F transcription factor 2	12.6205	3.73E-05	0.000575	• Non-Small Cell Lung Carcinoma	• Sun CC, Zhou Q, Hu W, Li SJ, Zhang F, Chen ZL, Li G, Bi ZY, Bi YY, Gong FY, Bo T. Transcriptional E2F1/2/5/8 as potential targets and transcriptional E2F3/6/7 as new biomarkers for the prognosis of human lung carcinoma. Aging (Albany NY). 2018 May;10(5):973.
12.	ADORA3	adenosine A3 receptor	11.98983	8.33E-05	0.001093	• Liver carcinoma<br>• Ulcerative colitis<br>• Inflammatory bowel disease	• Ayoub BM, Attia YM, Ahmed MS. Structural re-positioning, in silico molecular modelling, oxidative degradation, and biological screening of linagliptin as adenosine 3 receptor (ADORA3) modulators targeting hepatocellular carcinoma. Journal of enzyme inhibition and medicinal chemistry. 2018 Jan 1;33(1):858-66.<br>• Ren TH, Lv MM, An XM, Leung WK, Seto WK. Activation of adenosine A3 receptor inhibits inflammatory cytokine production in colonic mucosa of patients with ulcerative colitis by down-regulating the nuclear factor-kappa B signaling. Journal of digestive diseases. 2020 Jan;21(1):38-45<br>• Ren T, Qiu Y, Wu W, Feng X, Ye S, Wang Z, Tian T, He Y, Yu C, Zhou Y. Activation of adenosine A3 receptor alleviates TNF-α-induced inflammation through inhibition of the NF-κB signaling pathway in human colonic epithelial cells. Mediators of inflammation. 2014 Oct;2014.
13.	VNN1	vanin 1	11.76491	1.67E-05	0.00031	• Colorectal cancer<br>• Rectal cancer	• Løvf M, Nome T, Bruun J, Eknæs M, Bakken AC, Mpindi JP, Kilpinen S, Rognum TO, Nesbakken A, Kallioniemi O, Lothe RA. A novel transcript, VNN1-AB, as a biomarker for colorectal cancer. International journal of cancer. 2014 Nov 1;135(9):2077-84.<br>• Chai CY, Zhang Y, Song J, Lin SC, Sun S, Chang IW. VNN1 overexpression is associated with poor response to preoperative chemoradiotherapy and adverse prognosis in patients with rectal cancers. American journal of translational research. 2016;8(10):4455.
14.	TRPV4	transient receptor potential cation channel; subfamily V; member 4	11.66826	8.81E-13	3.73E-10	• fibrosis Pulmonary hypertension,<br>• acute lung injury, oedema	• Zhan L, Li J. The role of TRPV4 in fibrosis. Gene. 2018 Feb 5;642:1-8.<br>• Grace MS, Bonvini SJ, Belvisi MG, McIntyre P. Modulation of the TRPV4 ion channel as a therapeutic target for disease. Pharmacology & therapeutics. 2017 Sep 1;177:9-22.
15.	CA12	carbonic anhydrase XII	10.70786	8.60E-07	2.94E-05	• Breast Cancer	• Tafreshi NK, Lloyd MC, Proemsey JB, Bui MM, Kim J, Gillies RJ, Morse DL. Evaluation of CAIX and CAXII expression in breast cancer at varied O 2 levels: CAIX is the superior surrogate imaging

							biomarker of tumor hypoxia. Molecular imaging and biology. 2016 Apr 1;18(2):219-31.
16.	JPH4	junctophilin 4	10.56623	1.75E-07	8.19E-06	• Endometrial carcinoma	• Chung TK, Cheung TH, Huen NY, Wong KW, Lo KW, Yim SF, Siu NS, Wong YM, Tsang PT, Pang MW, Yu MY. Dysregulated microRNAs and their predicted targets associated with endometrioid endometrial adenocarcinoma in Hong Kong women. International journal of cancer. 2009 Mar 15;124(6):1358-65.
17.	GAPLINC	gastric adenocarcinoma associated; positive CD44 regulator; long intergenic non-coding RNA	10.38591	8.17E-07	2.82E-05	• Neoplasm Metastasis	• Zhang W, Yao H, Wu Y. Poor expression of long-chain noncoding RNA GAPLINC inhibits epithelial–mesenchymal transition, and invasion and migration of hepatocellular carcinoma cells. Anti-cancer drugs. 2019 Sep 1;30(8):784-94.
18.	CCL13	chemokine (C-C motif) ligand 13	10.2884	5.08E-08	2.89E-06	• Asthma	• Winkler C, Witte L, Moraw N, Faulenbach C, Müller M, Holz O, Schaumann F, Hohlfeld JM. Impact of endobronchial allergen provocation on macrophage phenotype in asthmatics. BMC immunology. 2014 Dec;15(1):1-1.
19.	FHAD1	forkhead-associated (FHA) phosphopeptide binding domain 1	10.13259	0.000231	0.002389	• Congestive Heart Failure	• Sung YJ, de Las Fuentes L, Winkler TW, Chasman DI, Bentley AR, Kraja AT, Ntalla I, Warren HR, Guo X, Schwander K, Manning AK. A multi-ancestry genome-wide study incorporating gene–smoking interactions identifies multiple new loci for pulse pressure and mean arterial pressure. Human molecular genetics. 2019 Aug 1;28(15):2615-33.
20.	NRCAM	neuronal cell adhesion molecule	9.618943	4.44E-08	2.57E-06	• Malignant neoplasm of breast,<br>• Colorectal carcinoma	• Yu JC, Hsiung CN, Hsu HM, Bao BY, Chen ST, Hsu GC, Chou WC, Hu LY, Ding SL, Cheng CW, Wu PE. Genetic variation in the genome-wide predicted estrogen response element-related sequences is associated with breast cancer development. Breast Cancer Research. 2011 Feb;13(1):1-8.<br>• Yi JM, Dhir M, Van Neste L, Downing SR, Jeschke J, Glöckner SC, de Freitas Calmon M, Hooker CM, Funes JM, Boshoff C, Smits KM. Genomic and epigenomic integration identifies a prognostic signature in colon cancer. Clinical Cancer Research. 2011 Mar 15;17(6):1535-45.
21.	C10orf128	chromosome 10 open reading frame 128	9.488851	6.16E-10	8.51E-08	• Aero-digestive squamous cell carcinoma	• Lesseur C, Ferreiro-Iglesias A, McKay JD, Bossé Y, Johansson M, Gaborieau V, Landi MT, Christiani DC, Caporaso NC, Bojesen SE, Amos CI. Genome-wide association meta-analysis identifies pleiotropic risk loci for aerodigestive squamous cell cancers. PLoS genetics. 2021 Mar 5;17(3):e1009254.
22.	PPM1N	protein phosphatase; Mg2+/Mn2+ dependent; 1N (putative)	9.426772	1.21E-07	6.00E-06	• Alzheimer's Disease	• Li QS, Parrado AR, Samtani MN, Narayan VA, Alzheimer’s Disease Neuroimaging Initiative. Variations in the FRA10AC1 fragile site and 15q21 are associated with cerebrospinal fluid Aβ1-42 level. PloS one. 2015 Aug 7;10(8):e0134000.
23.	COLEC12	collectin sub-family member 12	9.409228	2.37E-09	2.48E-07	• Diabetic Retinopathy	• Peng D, Wang J, Zhang R, Jiang F, Tang S, Chen M, Yan J, Sun X, Wang S, Wang T, Yan D. Common variants in or near ZNRF1, COLEC12, SCYL1BP1 and API5 are associated with diabetic retinopathy in Chinese patients with type 2 diabetes. Diabetologia. 2015 Jun;58(6):1231-8.
24.	MYO7B	myosin VIIB	9.164846	9.25E-05	0.001182	• Chronic Kidney Disease (CKD)<br>• Retinal Pigmentosa	• Yoshida T, Kato K, Yokoi K, Oguri M, Watanabe S, Metoki N, Yoshida H, Satoh K, Aoyagi Y, Nozawa Y, Yamada Y. Association of gene polymorphisms with chronic kidney disease in Japanese individuals. International journal of molecular medicine. 2009 Oct 1;24(4):539-47.<br>• He Y, Zhang X, Wang Y, Wang X, Liu Y, Yan T, Liu F, Liu C, Kong J. Mutations in MYO7B gene, a new suspected pathogenic gene, result in Autosomal Recessive Retinal Pigmentosa in a Chinese family. Investigative Ophthalmology & Visual Science. 2019 Jul 22;60(9):4481-.

25.	PDK4	pyruvate dehydrogenase kinase; isozyme 4	9.096747	3.06E-07	1.28E-05	• Liver carcinoma<br>• Diabetes Mellitus	• Song K, Kwon H, Han C, Zhang J, Dash S, Lim K, Wu T. Active glycolytic metabolism in CD133 (+) hepatocellular cancer stem cells: regulation by MIR-122. Oncotarget. 2015 Dec 1;6(38):40822.<br>• Putra SE, Singajaya S, Thesman F, Pranoto DA, Sanjaya R, Vianney YM, Artadana IB. Aberrant PDK4 promoter methylation preceding hyperglycemia in a mouse model. Applied biochemistry and biotechnology. 2020 Mar;190(3):1023-34.
26.	TLE3	transducin-like enhancer of split 3	8.903495	6.32E-12	1.94E-09	• Colorectal cancer<br>• Prostate cancer	• Liu L, Zhang Y, Wong CC, Zhang J, Dong Y, Li X, Kang W, Chan FK, Sung JJ, Yu J. RNF6 promotes colorectal cancer by activating the Wnt/β-catenin pathway via ubiquitination of TLE3. Cancer research. 2018 Apr 15;78(8):1958-71.<br>• Palit SA, Vis D, Stelloo S, Lieftink C, Prekovic S, Bekers E, Hofland I, Šuštić T, Wolters L, Beijersbergen R, Bergman AM. TLE3 loss confers AR inhibitor resistance by facilitating GR-mediated human prostate cancer cell growth. Elife. 2019 Dec 19;8:e47430.
27.	MARCO	macrophage receptor with collagenous structure	8.866188	4.30E-18	9.00E-15	• Hepatocellular carcinoma,	• Xiao Y, Chen B, Yang K, Wang Q, Liu P, Gu Y, Zhong Q, Liu Z, He Y, Liu Q. Down-regulation of MARCO associates with tumor progression in hepatocellular carcinoma. Experimental cell research. 2019 Oct 15;383(2):111542.
28.	LOC100506585	NA	8.79595	2.05E-08	1.31E-06	• uveal melanoma	• Qi Y, Yao R, Zhang W, Cui Q, Zhang F. Knockdown of Long Non-Coding RNA LOC100132707 Inhibits the Migration of Uveal Melanoma Cells via Silencing JAK2. OncoTargets and therapy. 2020;13:12955.
29.	FCGR3B	Fc fragment of IgG; low affinity IIIb; receptor (CD16b)	8.756927	0.000234	0.002411	• Lupus Erythematosus, Systemic	• Guo X, Fang X, He G, Zaman MH, Fei X, Qiao W, Deng GM. The role of neutrophils in skin damage induced by tissue-deposited lupus IgG. Immunology. 2018 Aug;154(4):604-12.
30.	ITGB3	integrin; beta 3 (platelet glycoprotein IIIa; antigen CD61)	8.199548	1.15E-08	8.20E-07	• Glanzmann thrombasthenia (GT)	• Lu Z, Nikuze L, Zhong Z, Li F, Zhang F, Liang K, Wei M, Wei H. Identification of one novel pathogenic ITGB3 mutation and two known mutations in two Chinese pedigrees with hereditary Glanzmann thrombasthenia. Platelets. 2020 Apr 2;31(3):355-9.
31.	PVALB	parvalbumin	8.096056	0.008801	0.039587	• Schizophrenia	• Shepard R, Heslin K, Hagerdorn P, Coutellier L. Downregulation of Npas4 in parvalbumin interneurons and cognitive deficits after neonatal NMDA receptor blockade: relevance for schizophrenia. Translational psychiatry. 2019 Feb 21;9(1):1-1.
32.	CACNA2D3	calcium channel; voltage-dependent; alpha 2/delta subunit 3	8.081992	0.012913	0.053016	• Esophageal Squamous Cell Carcinoma	• Nie C, Qin X, Li X, Tian B, Zhao Y, Jin Y, Li Y, Wang Q, Zeng D, Hong A, Chen X. CACNA2D3 enhances the chemosensitivity of esophageal squamous cell carcinoma to cisplatin via inducing Ca2+-mediated apoptosis and suppressing PI3K/Akt pathways. Frontiers in oncology. 2019 Apr 2;9:185.
33.	MRC1	mannose receptor; C type 1	7.965123	1.47E-16	1.84E-13	• Leprosy<br>• Asthma	• Wang D, Feng JQ, Li YY, Zhang DF, Li XA, Li QW, Yao YG. Genetic variants of the MRC1 gene and the IFNG gene are associated with leprosy in Han Chinese from Southwest China. Human genetics. 2012 Jul;131(7):1251-60.<br>• Hattori T, Konno S, Hizawa N, Isada A, Takahashi A, Shimizu K, Shimizu K, Gao P, Beaty TH, Barnes KC, Huang SK. Genetic variants in the mannose receptor gene (MRC1) are associated with asthma in two independent populations. Immunogenetics. 2009 Dec;61(11):731-8.
34.	MS4A6A	membrane-spanning 4-domains; subfamily A; member 6A	7.951355	1.02E-10	1.93E-08	• Alzheimer's Disease	• Cáceres A, Vargas JE, González JR. APOE and MS4A6A interact with GnRH signaling in Alzheimer's disease: Enrichment of epistatic effects. Alzheimer's & Dementia. 2017 Apr 1;13(4):493-7.

35.	CEACAM3	carcinoembryonic antigen-related cell adhesion molecule 3	7.786579	0.00017	0.001889	• Colorectal carcinoma	• Kim BC, Bae JH, Park SM, Won DY, Lee IK. Is ascites CEA a risk factor for peritoneal carcinomatosis in colorectal cancer?: a long-term follow-up study. International journal of colorectal disease. 2020 Jan;35(1):147-55.
36.	BNC2	basonuclin 2	7.778072	0.001585	0.010634	• Congenital Lower Urinary-Tract Obstruction (LUTO)<br>• Ovarian Cancer<br>• Adolescent Idiopathic Scoliosis	• Kolvenbach CM, Dworschak GC, Frese S, Japp AS, Schuster P, Wenzlitschke N, Yilmaz Ö, Lopes FM, Pryalukhin A, Schierbaum L, van der Zanden LF. Rare variants in BNC2 are implicated in autosomal-dominant congenital lower urinary-tract obstruction. The American Journal of Human Genetics. 2019 May 2;104(5):994-1006.<br>• Goode EL, Chenevix-Trench G, Song H, Ramus SJ, Notaridou M, Lawrenson K, Widschwendter M, Vierkant RA, Larson MC, Kjaer SK, Birrer MJ. A genome-wide association study identifies susceptibility loci for ovarian cancer at 2q31 and 8q24. Nature genetics. 2010 Oct;42(10):874-9.<br>• Ogura Y, Kou I, Miura S, Takahashi A, Xu L, Takeda K, Takahashi Y, Kono K, Kawakami N, Uno K, Ito M. A functional SNP in BNC2 is associated with adolescent idiopathic scoliosis. The American Journal of Human Genetics. 2015 Aug 6;97(2):337-42.
37.	LOC101929532	NA	7.760505	7.97E-07	2.77E-05	• NA	• NA
38.	IQCD	IQ motif containing D	7.738262	0.000169	0.001879	• Familial ventricular fibrillation	• Zhang P, Jiang W, Luo N, Zhu W, Fan L. Corrigendum to: IQ motif containing D (IQCD), a new acrosomal protein involved in the acrosome reaction and fertilisation. Reproduction, Fertility and Development. 2019;31(5):1033-.
39.	TLR5	toll-like receptor 5	7.706124	0.000119	0.001442	• Melioidosis<br>• Systemic Lupus Erythematosus (SLE)<br>• Cystic Fibrosis	• Birnie E, Weehuizen TA, Lankelma JM, de Jong HK, Koh GC, van Lieshout MH, Roelofs JJ, Budding AE, de Vos AF, van der Poll T, Wiersinga WJ. Role of toll-like receptor 5 (TLR5) in experimental melioidosis. Infection and immunity. 2019 Aug 1;87(8):e00409-18.<br>• Demirci FY, Manzi S, Ramsey-Goldman R, Kenney M, Shaw PS, Dunlop-Thomas CM, Kao AH, Rhew EY, Bontempo F, Kammerer C, Kamboh MI. Association study of Toll-like receptor 5 (TLR5) and Toll-like receptor 9 (TLR9) polymorphisms in systemic lupus erythematosus. The Journal of rheumatology. 2007 Aug 1;34(8):1708-11.<br>• Blohmke CJ, Victor RE, Hirschfeld AF, Elias IM, Hancock DG, Lane CR, Davidson AG, Wilcox PG, Smith KD, Overhage J, Hancock RE. Innate immunity mediated by TLR5 as a novel antiinflammatory target for cystic fibrosis lung disease. The Journal of Immunology. 2008 Jun 1;180(11):7764-73.
40.	TSPAN15	tetraspanin 15	7.683728	7.59E-08	4.01E-06	• Venous Thromboembolism<br>• Oesophageal Squamous Cell Carcinoma (OSCC)	• Germain M, Chasman DI, De Haan H, Tang W, Lindström S, Weng LC, De Andrade M, De Visser MC, Wiggins KL, Suchon P, Saut N. Meta-analysis of 65,734 individuals identifies TSPAN15 and SLC44A2 as two susceptibility loci for venous thromboembolism. The American Journal of Human Genetics. 2015 Apr 2;96(4):532-42.<br>• Zhang B, Zhang Z, Li L, Qin YR, Liu H, Jiang C, Zeng TT, Li MQ, Xie D, Li Y, Guan XY. TSPAN15 interacts with BTRC to promote oesophageal squamous cell carcinoma metastasis via activating NF-κB signaling. Nature communications. 2018 Apr 12;9(1):1-2.
41.	CIDEB	cell death-inducing DFFA-like effector b	7.666822	1.69E-10	2.80E-08	• Hepatitis B,	• Yasumoto J, Kasai H, Yoshimura K, Otoguro T, Watashi K, Wakita T, Yamashita A, Tanaka T, Takeda S, Moriishi K. Hepatitis B virus prevents excessive viral production via reduction of cell death-inducing DFF45-like effectors. Journal of General Virology. 2017 Jul 1;98(7):1762-73.

						• Fatty liver disease	• Li JZ, Ye J, Xue B, Qi J, Zhang J, Zhou Z, Li Q, Wen Z, Li P. Cideb regulates diet-induced obesity, liver steatosis, and insulin sensitivity by controlling lipogenesis and fatty acid oxidation. Diabetes. 2007 Oct 1;56(10):2523-32.
42.	SLC46A2	solute carrier family 46; member 2	7.527794	7.40E-05	0.000996	• NA	• NA
43.	EVI2A	ecotropic viral integration site 2A	7.518463	7.47E-09	5.98E-07	• Endometrial Carcinoma,	• Cawthon RM, O'Connell P, Buchberg AM, Viskochil D, Weiss RB, Culver M, Stevens J, Jenkins NA, Copeland NG, White R. Identification and characterization of transcripts from the neurofibromatosis 1 region: the sequence and genomic structure of EVI2 and mapping of other transcripts. Genomics. 1990 Aug 1;7(4):555-65.
44.	ACE	angiotensin I converting enzyme	7.120147	4.10E-18	9.00E-15	• Alzheimer's disease	• Quitterer U, AbdAlla S. Improvements of symptoms of Alzheimers disease by inhibition of the angiotensin system. Pharmacological research. 2020 Apr 1;154:104230.
45.	LY75-CD302	LY75-CD302 readthrough	6.793712	0.0281	0.095224	• Familial classical Hodgkin lymphoma	• Ristolainen H, Kilpivaara O, Kamper P, Taskinen M, Saarinen S, Leppä S, d'Amore F, Aaltonen LA. Identification of homozygous deletion in ACAN and other candidate variants in familial classical Hodgkin lymphoma by exome sequencing.
46.	PRAM1	PML-RARA regulated adaptor molecule 1	6.712835	1.23E-06	3.97E-05	• Acute Promyelocytic Leukemia<br>• Leukemia	• Gameiro P, Vieira S, Carrara P, Silva AL, Diamond J, de Sousa AB, Mehta AB, Prentice HG, Guimaraes JE, Hoffbrand AV, Foroni L. The PML-RAR alpha transcript in long-term follow-up of acute promyelocytic leukemia patients. haematologica. 2001 Jan 1;86(6):577-85.<br>• Barragán E, Bolufer P, Martın G, Cervera J, Moreno I, Capote FJ, Rosique P, Sanz MA. Identification of two atypical PML−RAR α transcripts in two patients with acute promyelocytic leukemia. Leukemia research. 2002 May 1;26(5):439-42.
47.	LRMP	lymphoid-restricted membrane protein	6.707103	2.76E-06	7.49E-05	• Lung Cancer	• Manenti G, Galbiati F, Pettinicchio A, Spinola M, Piconese S, Leoni VP, Conti B, Ravagnani F, Incarbone M, Pastorino U, Dragani TA. A V141L polymorphism of the human LRMP gene is associated with survival of lung cancer patients. Carcinogenesis. 2006 Jul 1;27(7):1386-90.
48.	ATP6V0D2	ATPase; H+ transporting; lysosomal 38kDa; V0 subunit d2	6.597816	1.99E-07	9.10E-06	• Stomach Cancer<br>• Pancreatic And Kidney Cancer	• Fukamachi T, Ikeda S, Saito H, Tagawa M, Kobayashi H. Expression of acidosis-dependent genes in human cancer nests. Molecular and clinical oncology. 2014 Nov 1;2(6):1160-6.<br>• Yang J, Guo F, Yuan L, Lv G, Gong J, Chen J. Elevated expression of the V-ATPase D2 subunit triggers increased energy metabolite levels in KrasG12D-driven cancer cells. Journal of cellular biochemistry. 2019 Jul;120(7):11690-701.
49.	MMP8	matrix metallopeptidase 8	6.564966	0.000111	0.001363	• Melanoma<br>• Inflammatory Bowel Disease	• Vihinen P, Koskivuo I, Syrjänen K, Tervahartiala T, Sorsa T, Pyrhönen S. Serum matrix metalloproteinase-8 is associated with ulceration and vascular invasion of malignant melanoma. Melanoma research. 2008 Aug 1;18(4):268-73.<br>• Schmidt J, Weigert M, Leuschner C, Hartmann H, Raddatz D, Haak R, Mausberg RF, Kottmann T, Schmalz G, Ziebolz D. Active matrix metalloproteinase-8 and periodontal bacteria—interlink between periodontitis and inflammatory bowel disease?. Journal of periodontology. 2018 Jun;89(6):699-707.
50.	AMICA1	adhesion molecule; interacts with CXADR antigen 1	6.515126	3.66E-06	9.45E-05	• Kawasaki Disease	• Kentsis A, Shulman A, Ahmed S, Brennan E, Monuteaux MC, Lee YH, Lipsett S, Paulo JA, Dedeoglu F, Fuhlbrigge R, Bachur R. Urine proteomics for discovery of improved diagnostic markers of Kawasaki disease. EMBO molecular medicine. 2013 Feb;5(2):210-20.

51.	H3F3A	H3 histone; family 3A	-747.187	0.002392	0.01467	• Chondroblastoma<br>• Pediatric Brain And Bone Malignancies	• Righi A, Mancini I, Gambarotti M, Picci P, Gamberi G, Marraccini C, Dei Tos AP, Simi L, Pinzani P, Franchi A. Histone 3.3 mutations in giant cell tumor and giant cell–rich sarcomas of bone. Human pathology. 2017 Oct 1;68:128-35.<br>• Shi L, Shi J, Shi X, Li W, Wen H. Histone H3. 3 G34 mutations alter histone H3K36 and H3K27 methylation in cis. Journal of molecular biology. 2018 May 25;430(11):1562-5.
52.	CCL19	chemokine (C-C motif) ligand 19	-48.3947	1.58E-13	7.35E-11	• Pneumonia	• Hoffmann-Vold AM, Hesselstrand R, Fretheim H, Ueland T, Andreassen AK, Brunborg C, Palchevskiy V, Midtvedt Ø, Garen T, Aukrust P, Belperio JA. CCL21 as a potential serum biomarker for pulmonary arterial hypertension in systemic sclerosis. Arthritis & Rheumatology. 2018 Oct;70(10):1644-53.
53.	KIAA1644	KIAA1644	-32.2811	0.000138	0.001611	• NA	• NA
54.	FCAMR	Fc receptor; IgA; IgM; high affinity	-28.1209	9.66E-09	7.24E-07	• Coronary Arteriosclerosis	• Ward-Caviness CK, Neas LM, Blach C, Haynes CS, LaRocque-Abramson K, Grass E, Dowdy ZE, Devlin RB, Diaz-Sanchez D, Cascio WE, Miranda ML. A genome-wide trans-ethnic interaction study links the PIGR-FCAMR locus to coronary atherosclerosis via interactions between genetic variants and residential exposure to traffic. PloS one. 2017 Mar 29;12(3):e017388
55.	CAMK2A	calcium/calmodulin-dependent protein kinase II alpha	-26.0324	4.40E-06	0.000109	• Bipolar disorder	• Li H, Zhou DS, Chang H, Wang L, Liu W, Dai SX, Zhang C, Cai J, Liu W, Li X, Fan W. Interactome analyses implicated CAMK2A in the genetic predisposition and pharmacological mechanism of bipolar disorder. Journal of psychiatric research. 2019 Aug 1;115:165-75.
56.	EBF4	early B-cell factor 4	-21.6916	1.58E-07	7.49E-06	• NA	• NA
57.	SLC8A3	solute carrier family 8 (sodium/calcium exchanger); member 3	-19.9602	1.34E-08	9.21E-07	• Rheumatoid arthritis	• Julià A, González I, Fernández-Nebro A, Blanco F, Rodriguez L, González A, Cañete JD, Maymó J, Alperi-López M, Olivé A, Corominas H. A genome-wide association study identifies SLC8A3 as a susceptibility locus for ACPA-positive rheumatoid arthritis. Rheumatology. 2016 Jun 1;55(6):1106-11.
58.	MMP10	matrix metallopeptidase 10	-19.4389	3.83E-12	1.30E-09	• Asthma,<br>• Neoplasm metastasis	• Kuo CH, Pavlidis S, Zhu J, Loza M, Baribaud F, Rowe A, Pandis I, Gibeon D, Hoda U, Sousa A, Wilson SJ. Contribution of airway eosinophils in airway wall remodeling in asthma: Role of MMP-10 and MET. Allergy. 2019 Jun;74(6):1102-12.<br>• Wang D, Luo H, Huo Z, Chen M, Han Z, Hung M, Su B, Li Y, Wang X, Guo X, Xiao H. Irradiation-induced dynamic changes of gene signatures reveal gain of metastatic ability in nasopharyngeal carcinoma. American journal of cancer research. 2019;9(3):479.
59.	ADAM19	ADAM metallopeptidase domain 19	-16.5674	3.63E-09	3.34E-07	• COPD,<br>• Colorectal Carcinoma	• Sakornsakolpat P, Prokopenko D, Lamontagne M, Reeve NF, Guyatt AL, Jackson VE, Shrine N, Qiao D, Bartz TM, Kim DK, Lee MK. Genetic landscape of chronic obstructive pulmonary disease identifies heterogeneous cell-type and phenotype associations. Nature genetics. 2019 Mar;51(3):494-505.<br>• Zhang Q, Yu L, Qin D, Huang R, Jiang X, Zou C, Tang Q, Chen Y, Wang G, Wang X, Gao X. Role of microRNA-30c targeting ADAM19 in colorectal cancer. PLoS One. 2015 Mar 23;10(3):e0120698.
60.	GJA4	gap junction protein; alpha 4; 37kDa	-16.0479	0.000454	0.004066	• Hypertensive disease,	• Le Gal L, Pellegrin M, Santoro T, Mazzolai L, Kurtz A, Meda P, Wagner C, Haefliger JA. Connexin37-Dependent Mechanisms Selectively Contribute to Modulate Angiotensin II-Mediated

						• Coronary artery disease	Hypertension. Journal of the American Heart Association. 2019 Apr 16;8(8):e010823.<br>• Han Y, Xi S, Zhang X, Yan C, Yang Y, Kang J. Association of connexin 37 gene polymorphisms with risk of coronary artery disease in northern Han Chinese. Cardiology. 2008;110(4):260-5.
61.	IRF4	interferon regulatory factor 4	-15.5475	1.51E-11	3.88E-09	• Chronic lymphocytic leukemia	• Asslaber D, Qi Y, Maeding N, Steiner M, Denk U, Höpner JP, Hartmann TN, Zaborsky N, Greil R, Egle A. B-cell–specific IRF4 deletion accelerates chronic lymphocytic leukemia development by enhanced tumor immune evasion. blood. 2019 Nov 14;134(20):1717-29.
62.	ANGPTL2	angiopoietin-like 2	-14.0844	3.76E-06	9.65e-05	• Dermatomyositis<br>• Type 2 Diabetes Mellitus<br>• Ovarian Cancer	• Ogata A, Endo M, Aoi J, Takahashi O, Kadomatsu T, Miyata K, Tian Z, Jinnin M, Fukushima S, Ihn H, Oike Y. The role of angiopoietin-like protein 2 in pathogenesis of dermatomyositis. Biochemical and biophysical research communications. 2012 Feb 17;418(3):494-9.<br>• Yoshinaga T, Niou T, Niihara T, Kajiya Y, Hori E, Tomiyoshi A, Tokudome E, Nishimata H, Takei T, Yoshida M. Angiopoietin-like protein 2 is a useful biomarker for pancreatic cancer that is associated with type 2 diabetes mellitus and inflammation. Journal of Cancer. 2018;9(24):4736.<br>• Kikuchi R, Tsuda H, Kozaki KI, Kanai Y, Kasamatsu T, Sengoku K, Hirohashi S, Inazawa J, Imoto I. Frequent inactivation of a putative tumor suppressor, angiopoietin-like protein 2, in ovarian cancer. Cancer research. 2008 Jul 1;68(13):5067-75.
63.	NR4A3	nuclear receptor subfamily 4; group A; member 3	-13.0153	6.29E-09	5.20E-07	• Extraskeletal Myxoid Chondrosarcoma	• Urbini M, Astolfi A, Pantaleo MA, Serravalle S, Dei Tos AP, Picci P, Indio V, Sbaraglia M, Benini S, Righi A, Gambarotti M. HSPA 8 as a novel fusion partner of NR 4 A 3 in extraskeletal myxoid chondrosarcoma. Genes, Chromosomes and Cancer. 2017 Jul;56(7):582-6.
64.	LOC440896	NA	-11.4647	5.18E-08	2.93E-06	• Neuroblastoma	• Sathipati SY, Sahu D, Huang HC, Lin Y, Ho SY. Identification and characterization of the lncRNA signature associated with overall survival in patients with neuroblastoma. Scientific reports. 2019 Mar 26;9(1):1-3.
65.	LINC00158	long intergenic non-protein coding RNA 158	-11.3156	1.99E-07	9.10E-06	• Endometrial Carcinoma	• Cai Y, Hao M, Chang Y, Liu Y. Up-Regulation of LINC00158 Contributes to Endometrial Carcinoma Progression by Interacting with HMGB2.
66.	ADIRF	adipogenesis regulatory factor	-9.99448	4.87E-07	1.84E-05	• Prostate carcinoma	• Meng J, Wang LH, Zou CL, Dai SM, Zhang J, Lu Y. C10orf116 gene copy number loss in prostate cancer: clinicopathological correlations and prognostic significance. Medical science monitor: international medical journal of experimental and clinical research. 2017;23:5176.
67.	CCR7	chemokine (C-C motif) receptor 7	-9.80353	2.56E-07	1.12E-05	• Adult T-cell leukemia	• Kataoka K, Nagata Y, Kitanaka A, Shiraishi Y, Shimamura T, Yasunaga JI, Totoki Y, Chiba K, Sato-Otsubo A, Nagae G, Ishii R. Integrated molecular analysis of adult T cell leukemia/lymphoma. Nature genetics. 2015 Nov;47(11):1304-15.
68.	FSCN1	fascin actin-bundling protein 1	-9.7114	3.46E-10	5.12E-08	• Breast Carcinoma	• Tampaki EC, Tampakis A, Nonni A, von Flüe M, Patsouris E, Kontzoglou K, Kouraklis G. Combined Fascin-1 and MAP17 expression in breast cancer identifies patients with high risk for disease recurrence. Molecular diagnosis & therapy. 2019 Oct;23(5):635-44.

69.	ARSI	arylsulfatase family; member I	-9.44029	0.004234	0.022672	• Retinitis Pigmentosa	• Oshikawa M, Usami R, Kato S. Characterization of the arylsulfatase I (ARSI) gene preferentially expressed in the human retinal pigment epithelium cell line ARPE-19. Molecular vision. 2009;15:482.
70.	SLCO5A1	solute carrier organic anion transporter family; member 5A1	-9.2481	2.39E-09	2.49E-07	• Mesomelia-synostoses syndrome (MSS)	• Kohmoto T, Naruto T, Watanabe M, Fujita Y, Ujiro S, Okamoto N, Horikawa H, Masuda K, Imoto I. A 590 kb deletion caused by non-allelic homologous recombination between two LINE-1 elements in a patient with mesomelia-synostosis syndrome. American Journal of Medical Genetics Part A. 2017 Apr;173(4):1082-6.
71.	SYNPO2	synaptopodin 2	-8.94943	3.57E-14	2.37E-11	• Prostate Cancer	• Kai F, Fawcett JP, Duncan R. Synaptopodin-2 induces assembly of peripheral actin bundles and immature focal adhesions to promote lamellipodia formation and prostate cancer cell migration. Oncotarget. 2015 May 10;6(13):11162.
72.	TMCC2	transmembrane and coiled-coil domain family 2	-8.87334	5.93E-05	0.000832	• Neurodegeneration	• Hopkins PC. Neurodegeneration in a Drosophila model for the function of TMCC2, an amyloid protein precursor-interacting and apolipoprotein E-binding protein. PLoS One. 2013 Feb 7;8(2):e55810.
73.	TMEM97	transmembrane protein 97	-8.62361	1.77E-08	1.16E-06	• Cancer	• Nicholson HE, Alsharif WF, Comeau AB, Mesangeau C, Intagliata S, Mottinelli M, McCurdy CR, Bowen WD. Divergent cytotoxic and metabolically stimulative functions of sigma-2 receptors: structure-activity relationships of 6-acetyl-3-(4-(4-(4-fluorophenyl) piperazin-1-yl) butyl) benzo [d] oxazol-2 (3H)-one (SN79) derivatives. Journal of Pharmacology and Experimental Therapeutics. 2019 Feb 1;368(2):272-81.
74.	EDN1	endothelin 1	-8.51709	2.77E-12	9.94E-10	• Auriculo-condylar syndrome	• Romanelli Tavares VL, Zechi-Ceide RM, Bertola DR, Gordon CT, Ferreira SG, Hsia GS, Yamamoto GL, Ezquina SA, Kokitsu-Nakata NM, Vendramini-Pittoli S, Freitas RS. Targeted molecular investigation in patients within the clinical spectrum of auriculocondylar syndrome. American Journal of Medical Genetics Part A. 2017 Apr;173(4):938-45.
75.	NTN1	netrin 1	-8.43453	1.37E-05	0.000268	• Subarachnoid Hemorrhage	• Xie Z, Huang L, Enkhjargal B, Reis C, Wan W, Tang J, Cheng Y, Zhang JH. Intranasal administration of recombinant Netrin-1 attenuates neuronal apoptosis by activating DCC/APPL-1/AKT signaling pathway after subarachnoid hemorrhage in rats. Neuropharmacology. 2017 Jun 1;119:123-33.
76.	LAMP3	lysosomal-associated membrane protein 3	-7.99458	9.69E-15	8.12E-12	• Parkinson disease	• Li NN, Tan EK, Chang XL, Mao XY, Zhao DM, Zhang JH, Liao Q, Peng R. MCCC 1/LAMP 3 reduces risk of sporadic P arkinson's disease in H an C hinese. Acta Neurologica Scandinavica. 2013 Aug;128(2):136-9.
77.	UBD	ubiquitin D	-7.92319	4.64E-09	4.00E-07	• Kidney diseases	• Zhang JY, Wang M, Tian L, Genovese G, Yan P, Wilson JG, Thadhani R, Mottl AK, Appel GB, Bick AG, Sampson MG. UBD modifies APOL1-induced kidney disease risk. Proceedings of the National Academy of Sciences. 2018 Mar 27;115(13):3446-51.
78.	GUCY1A3	guanylate cyclase 1; soluble; alpha 3	-7.64045	1.83E-09	2.06E-07	• Moyamoya disease (MMD)	• Wallace S, Guo DC, Regalado E, Mellor-Crummey L, Bamshad M, Nickerson DA, Dauser R, Hanchard N, Marom R, Martin E, Berka V. Disrupted nitric oxide signaling due to GUCY1A3 mutations increases risk for moyamoya disease, achalasia and hypertension. Clinical genetics. 2016 Oct;90(4):351-60.
79.	CXCL13	chemokine (C-X-C motif) ligand 13	-7.48785	5.98E-05	0.000837	• Rheumatoid Arthritis	• Bao YQ, Wang JP, Dai ZW, Mao YM, Wu J, Guo HS, Xia YR, Ye DQ. Increased circulating CXCL13 levels in systemic lupus erythematosus

						• Breast Carcinoma	and rheumatoid arthritis: a meta-analysis. Clinical rheumatology. 2020 Jan;39(1):281-90.<br>• Razis E, Kalogeras KT, Kotsantis I, Koliou GA, Manousou K, Wirtz R, Veltrup E, Patsea H, Poulakaki N, Dionysopoulos D, Pervana S. The role of CXCL13 and CXCL9 in early breast cancer. Clinical breast cancer. 2020 Feb 1;20(1):e36-53.
80.	ADAMTS4	ADAM metallopeptidase with thrombospondin type 1 motif; 4	-7.45918	1.29E-05	0.000254	• Degenerative polyarthritis<br>• Alzheimer's disease	• Meng P, Zhang Y, Wei H, Tan S, Guo X, Wang S, Yu Y. ADAMTS4 and ADAMTS5 may be considered as new molecular therapeutic targets for cartilage damages with Kashin-Beck Disease. Medical hypotheses. 2020 Feb 1;135:109440.<br>• Walter S, Jumpertz T, Hüttenrauch M, Ogorek I, Gerber H, Storck SE, Zampar S, Dimitrov M, Lehmann S, Lepka K, Berndt C. The metalloprotease ADAMTS4 generates N-truncated Aβ4–x species and marks oligodendrocytes as a source of amyloidogenic peptides in Alzheimer's disease. Acta neuropathologica. 2019 Feb;137(2):239-57.
81.	OSR2	odd-skipped related transciption factor 2	-7.34523	0.002004	0.012767	• Endometriosis	• Aghajanova L, Tatsumi K, Horcajadas JA, Zamah AM, Esteban FJ, Herndon CN, Conti M, Giudice LC. Unique transcriptome, pathways, and networks in the human endometrial fibroblast response to progesterone in endometriosis. Biology of reproduction. 2011 Apr 1;84(4):801-15.
82.	SLITRK5	SLIT and NTRK-like family; member 5	-6.85177	0.001368	0.009548	• Obsessive-compulsive disorder (OCD)	• Todorov G, Mayilvahanan K, Ashurov D, Cunha C. RETRACTED ARTICLE: Amelioration of obsessive-compulsive disorder in three mouse models treated with one epigenetic drug: unraveling the underlying mechanism. Scientific reports. 2019 Jun 19;9(1):1-3.
83.	CCL22	chemokine (C-C motif) ligand 22	-6.70789	7.40E-06	0.000165	• Atopic dermatitis	• Miake S, Tsuji G, Takemura M, Hashimoto-Hachiya A, Vu YH, Furue M, Nakahara T. IL-4 augments IL-31/IL-31 receptor alpha interaction leading to enhanced Ccl 17 and Ccl 22 production in dendritic cells: implications for atopic dermatitis. International journal of molecular sciences. 2019 Jan;20(16):4053.
84.	VTN	vitronectin	-6.55394	9.67E-09	7.24E-07	• Lung Fibrosis<br>• Chronic Obstructive Pulmonary Disease (COPD)<br>• Type 2 Diabetes Mellitus	• Shen TL, Liu MN, Zhang Q, Feng W, Yu W, Fu XL, Cai XW. The positive role of vitronectin in radiation induced lung toxicity: the in vitro and in vivo mechanism study. Journal of translational medicine. 2018 Dec;16(1):1-2.<br>• Salazar-Peláez LM, Abraham T, Herrera AM, Correa MA, Ortega JE, Paré PD, Seow CY. Vitronectin expression in the airways of subjects with asthma and chronic obstructive pulmonary disease. PloS one. 2015 Mar 13;10(3):e0119717.<br>• Alessi MC, Nicaud V, Scroyen I, Lange C, Saut N, Fumeron F, Marre M, Lantieri O, Fontaine-Bisson B, Juhan-Vague I, Balkau B. Association of vitronectin and plasminogen activator inhibitor-1 levels with the risk of metabolic syndrome and type 2 diabetes mellitus. Thrombosis and haemostasis. 2011;106(09):416-22.
85.	ADAM11	ADAM metallopeptidase domain 11	-6.55304	1.74E-06	5.18E-05	• Tuberculosis,<br>• Atopic dermatitis, Eczema	• Luo J, Zhang M, Yan B, Li F, Guan S, Chang K, Jiang W, Xu H, Yuan T, Chen M, Deng S. Diagnostic performance of plasma cytokine biosignature combination and MCP-1 as individual biomarkers for differentiating stages Mycobacterium tuberculosis infection. Journal of Infection. 2019 Apr 1;78(4):281-91.<br>• Kim WH, An HJ, Kim JY, Gwon MG, Gu H, Lee SJ, Park JY, Park KD, Han SM, Kim MK, Park KK. Apamin inhibits TNF-α-and IFN-γ-induced inflammatory cytokines and chemokines via suppressions of NF-κB

							signaling pathway and STAT in human keratinocytes. Pharmacological Reports. 2017 Oct 1;69(5):1030-5.
86.	ETV3L	ets variant 3-like	-6.04055	8.36E-08	4.32E-06	• Currarino Syndrome<br>• Attention-Deficit/Hyperactivity Disorder (ADHD)	• Holm I, Spildrejorde M, Stadheim B, Eiklid KL, Samarakoon PS. Whole exome sequencing of sporadic patients with Currarino Syndrome: a report of three trios. Gene. 2017 Aug 15;624:50-5<br>• Anney RJ, Lasky-Su J, Ó'Dúshláine C, Kenny E, Neale BM, Mulligan A, Franke B, Zhou K, Chen W, Christiansen H, Arias-Vásquez A. Conduct disorder and ADHD: evaluation of conduct problems as a categorical and quantitative trait in the international multicentre ADHD genetics study. American Journal of Medical Genetics Part B: Neuropsychiatric Genetics. 2008 Dec 5;147(8):1369-78..
87.	SRGAP1	SLIT-ROBO Rho GTPase activating protein 1	-5.90558	5.09E-05	0.000739	• Papillary thyroid carcinoma (PTC)	• He H, Bronisz A, Liyanarachchi S, Nagy R, Li W, Huang Y, Akagi K, Saji M, Kula D, Wojcicka A, Sebastian N. SRGAP1 is a candidate gene for papillary thyroid carcinoma susceptibility. The Journal of Clinical Endocrinology & Metabolism. 2013 May 1;98(5):E973-80.
88.	SLIT2	slit guidance ligand 2	-5.8822	0.000572	0.004863	• Liver carcinoma	• Sun G, Zhang C, Feng M, Liu W, Xie H, Qin Q, Zhao E, Wan L. Methylation analysis of p16, SLIT2, SCARA5, and Runx3 genes in hepatocellular carcinoma. Medicine. 2017 Oct;96(41).
89.	TGFA	transforming growth factor; alpha	-5.78336	2.40E-06	6.74E-05	• Hepatocellular Carcinoma<br>• Nonsyndromic Cleft Lip<br>• Thyroid Carcinogenesis	• Daveau M, Scotte M, François A, Coulouarn C, Ros G, Tallet Y, Hiron M, Hellot MF, Salier JP. Hepatocyte growth factor, transforming growth factor α, and their receptors as combined markers of prognosis in hepatocellular carcinoma. Molecular Carcinogenesis: Published in cooperation with the University of Texas MD Anderson Cancer Center. 2003 Mar;36(3):130-41.<br>• Yan C, Deng-Qi H, Li-Ya C, Mang Y, Ke-Hu Y. Transforming growth factor alpha Taq I polymorphisms and nonsyndromic cleft lip and/or palate risk: a meta-analysis. The Cleft Palate-Craniofacial Journal. 2018 Jul;55(6):814-20.<br>• Aasland R, Akslen LA, Varhaug JE, Lillehaug JR. Co-expression of the genes encoding transforming growth factor-α and its receptor in papillary carcinomas of the thyroid. International journal of cancer. 1990 Sep 15;46(3):382-7.
90.	PSD	pleckstrin and Sec7 domain containing	-5.71228	5.77E-06	0.000135	• Gastric Cancer<br>• Colorectal Cancer<br>• Papillary Thyroid Cancer	• Zhu X, Liu J, Xu X, Zhang C, Dai D. The Pleckstrin and Sec7 domain-containing gene as a novel epigenetic modification marker in human gastric cancer and its clinical significance. International journal of oncology. 2015 Jan 1;46(1):195-204.<br>• Kato T, Suzuki K, Okada S, Kamiyama H, Maeda T, Saito M, Koizumi K, Miyaki Y, Konishi F. Aberrant methylation of PSD disturbs Rac1-mediated immune responses governing neutrophil chemotaxis and apoptosis in ulcerative colitis-associated carcinogenesis. International journal of oncology. 2012 Apr 1;40(4):942-50.<br>• Jin L, Zheng D, Bhandari A, Chen D, Xia E, Guan Y, Wen J, Wang O. PSD3 is an oncogene that promotes proliferation, migration, invasion, and G1/S transition while inhibits apoptotic in papillary thyroid cancer. Journal of Cancer. 2021 Jan 1;12(18):5413-22.
91.	ACHE	acetylcholinesterase (Yt blood group)	-5.70369	4.23E-06	0.000105	• Alzheimer's disease,<br>• Amyloidoisis,	• El-Sayed NF, El-Hussieny M, Ewies EF, Fouad MA, Boulos LS. New phosphazine and phosphazide derivatives as multifunctional ligands targeting acetylcholinesterase and β-Amyloid aggregation for treatment of Alzheimer's disease. Bioorganic chemistry. 2020 Jan 1;95:103499.<br>• Fawzi SF, Menze ET, Tadros MG. Deferiprone ameliorates memory impairment in Scopolamine-treated rats: The impact of its iron-

						• Persenile dementia	chelating effect on β-amyloid disposition. Behavioural brain research. 2020 Jan 27;378:112314.<br>• Tan EC, Johnell K, Bell JS, Garcia-Ptacek S, Fastbom J, Nordström P, Eriksdotter M. Do acetylcholinesterase inhibitors prevent or delay psychotropic prescribing in people with dementia? Analyses of the Swedish Dementia Registry. The American journal of geriatric psychiatry. 2020 Jan 1;28(1):108-17.
92.	BCL2L14	BCL2-like 14 (apoptosis facilitator)	-5.52096	1.72E-08	1.13E-06	• Hyperprolactinemia<br>• Endometrioma<br>• Carcinoma	• Newey PJ, Gorvin CM, Cleland SJ, Willberg CB, Bridge M, Azharuddin M, Drummond RS, van der Merwe PA, Klenerman P, Bountra C, Thakker RV. Mutant prolactin receptor and familial hyperprolactinemia. New England Journal of Medicine. 2013 Nov 21;369(21):2012-20.<br>• Aghajanova L, Tatsumi K, Horcajadas JA, Zamah AM, Esteban FJ, Herndon CN, Conti M, Giudice LC. Unique transcriptome, pathways, and networks in the human endometrial fibroblast response to progesterone in endometriosis. Biology of reproduction. 2011 Apr 1;84(4):801-15.<br>• Papaconstantinou AD, Snyderwine EG. Proliferation and apoptosis in PhIP-induced rat mammary gland carcinomas with elevated phosphotyrosine-STAT5a. FEBS letters. 2007 Jan 9;581(1):29-33.
93.	LINC00504	long intergenic non-protein coding RNA 504	-5.35038	0.000246	0.002496	• Non-Small Cell Lung Carcinoma,<br>• Colon carcinoma	• Ma HP, Wang LX, Li W, Guo HH, Wu Y, Li XY. Upregulation of LINC00504 is associated with aggressive progression and poor prognosis in non-small cell lung cancer. Eur Rev Med Pharmacol Sci. 2020 Jan 1;24(2):699-703.<br>• Feng J, Ma J, Liu S, Wang J, Chen Y. A noncoding RNA LINC00504 interacts with c-Myc to regulate tumor metabolism in colon cancer. Journal of cellular biochemistry. 2019 Sep;120(9):14725-34.
94.	ZNF366	zinc finger protein 366 DC-SCRIPT	-5.33286	2.37E-14	1.75E-11	• Breast Cancer	• Ansems M, Søndergaard JN, Sieuwerts AM, Looman MW, Smid M, de Graaf AM, de Weerd V, Zuidscherwoude M, Foekens JA, Martens JW, Adema GJ. DC-SCRIPT is a novel regulator of the tumor suppressor gene CDKN2B and induces cell cycle arrest in ERα-positive breast cancer cells. Breast cancer research and treatment. 2015 Feb;149(3):693-703.
95.	CXCL9	chemokine (C-X-C motif) ligand 9	-5.22891	1.93E-09	2.11E-07	• Breast cancer	• Razis E, Kalogeras KT, Kotsantis I, Koliou GA, Manousou K, Wirtz R, Veltrup E, Patsea H, Poulakaki N, Dionysopoulos D, Pervana S. The role of CXCL13 and CXCL9 in early breast cancer. Clinical breast cancer. 2020 Feb 1;20(1):e36-53.
96.	PDZD2	PDZ domain containing 2	-5.22174	0.001584	0.010634	• Prostate Tumorigenesis	• He F, Fang L, Yin Q. miR-363 acts as a tumor suppressor in osteosarcoma cells by inhibiting PDZD2. Oncology reports. 2019 May 1;41(5):2729-38.
97.	EBI3	Epstein-Barr virus induced 3	-5.16749	1.75E-14	1.38E-11	• Rheumatoid Arthritis<br>• Colorectal Cancer	• Filková M, Vernerová Z, Hulejová H, Prajzlerová K, Veigl D, Pavelka K, Vencovský J, Šenolt L. Pro-inflammatory effects of interleukin-35 in rheumatoid arthritis. Cytokine. 2015 May 1;73(1):36-43.<br>• Liang Y, Chen Q, Du W, Chen C, Li F, Yang J, Peng J, Kang D, Lin B, Chai X, Zhou K. Epstein-Barr virus-induced gene 3 (EBI3) blocking leads to induce antitumor cytotoxic T lymphocyte response and suppress tumor growth in colorectal cancer by bidirectional reciprocal-regulation STAT3 signaling pathway. Mediators of inflammation. 2016 May 10;2016.
98.	ANKRD33B	ankyrin repeat domain 33B	-5.09279	9.01E-07	3.06E-05	• NA	• NA

99.	RCAN2	regulator of calcineurin 2	-5.0281	0.000263	0.002631	• Gastric Cancer<br>• stomach carcinoma	• Hattori Y, Sentani K, Shinmei S, Oo HZ, Hattori T, Imai T, Sekino Y, Sakamoto N, Oue N, Niitsu H, Hinoi T. Clinicopathological significance of RCAN2 production in gastric carcinoma. Histopathology. 2019 Feb;74(3):430-42<br>• Hattori Y, Sentani K, Shinmei S, Oo HZ, Hattori T, Imai T, Sekino Y, Sakamoto N, Oue N, Niitsu H, Hinoi T. Clinicopathological significance of RCAN2 production in gastric carcinoma. Histopathology. 2019 Feb;74(3):430-42.
100.	PRLR	prolactin receptor	-5.02517	1.13E-09	1.35E-07	• Colon cancer	• Bhatavdekar J, Patel D, Ghosh N, Vora H, Shah N, Karelia N, Balar D, Chikhlikar P, Dave R. Interrelationship of prolactin and its receptor in carcinoma of colon and rectum: a preliminary report. Journal of surgical oncology. 1994 Apr;55(4):246-9.

Table 5: Top genes affected by IFN-λ4 (IFN-λ4 vs NT; M2-MDM)

S.No	Gene symbol	Name	Foldchange	PValue	FDR	Associated Disease	Reference
1.	CLEC5A	C-type lectin domain family 5; member A	3.410393	0.007795	1	• Sever dengue and Dengue shock syndrome<br>• Rheumatoid Arthritis	• Chen ST, Lin YL, Huang MT, Wu MF, Cheng SC, Lei HY, Lee CK, Chiou TW, Wong CH, Hsieh SL. CLEC5A is critical for dengue-virus-induced lethal disease. Nature. 2008 May;453(7195):672-6.<br>• Chen DY, Yao L, Chen YM, Lin CC, Huang KC, Chen ST, Lan JL, Hsieh SL. A potential role of myeloid DAP12-associating lectin (MDL)-1 in the regulation of inflammation in rheumatoid arthritis patients. PLoS One. 2014 Jan 21;9(1):e86105.
2.	PLEKHA5	pleckstrin homology domain containing; family A member 5	2.940726	0.005293	1	• Melanoma Brain Metastasis	• Jilaveanu LB, Parisi F, Barr ML, Zito CR, Cruz-Munoz W, Kerbel RS, Rimm DL, Bosenberg MW, Halaban R, Kluger Y, Kluger HM. PLEKHA5 as a biomarker and potential mediator of melanoma brain metastasis. Clinical Cancer Research. 2015 May 1;21(9):2138-47.
3.	ARAP3	ArfGAP with RhoGAP domain; ankyrin repeat and PH domain 3	2.451104	0.009496	1	• Breast Cancer<br>• Anthrax	• Han JJ, Du BR, Zhang CH. Bioinformatic analysis of prognostic value of ARAP3 in breast cancer and the associated signaling pathways. Eur Rev Med Pharmacol Sci. 2017 May 1;21(10):2405-12.<br>• Lu Q, Wei W, Kowalski PE, Chang AC, Cohen SN. EST-based genome-wide gene inactivation identifies ARAP3 as a host protein affecting cellular susceptibility to anthrax toxin. Proceedings of the National Academy of Sciences. 2004 Dec 7;101(49):17246-51.
4.	PCDHGA11	protocadherin gamma subfamily A; 11	2.434315	0.047776	1	• Astrocytoma and Glioma	• Waha A, Güntner S, Huang TH, Yan PS, Arslan B, Pietsch T, Wiestler OD, Waha A. Epigenetic Silencing of the Protocadherin Family Member PCDH-γ-All in Astrocytomas. Neoplasia. 2005 Mar 1;7(3):193-9.
5.	IL1R2	interleukin 1 receptor; type II	2.218644	0.033769	1	• Ulcerative colitis<br>• Ankylosing Spondylitis	• Yoshida K, Murayama MA, Shimizu K, Tang C, Katagiri N, Matsuo K, Fukai F, Iwakura Y. IL-1R2 deficiency suppresses dextran sodium sulfate-induced colitis in mice via regulation of microbiota. Biochemical and biophysical research communications. 2018 Feb 12;496(3):934-40.<br>• Reveille JD, Sims AM, Danoy P, Evans DM, Leo P, Pointon JJ, Jin R, Zhou X, Bradbury LA, Appleton LH, Davis JC. Genome-wide

						• Sepsis	association study of ankylosing spondylitis identifies non-MHC susceptibility loci. Nature genetics. 2010 Feb;42(2):123.<br>• Lang Y, Jiang Y, Gao M, Wang W, Wang N, Wang K, Zhang H, Chen G, Liu K, Liu M, Yang M. Interleukin-1 receptor 2: A new biomarker for sepsis diagnosis and gram-negative/gram-positive bacterial differentiation. Shock. 2017 Jan 1;47(1):119-24
6.	FBP1	fructose-1;6-bisphosphatase 1	2.097735	0.030762	1	• Liver Carcinoma<br>• Tumour progression	• Yang J, Wang C, Zhao F, Luo X, Qin M, Arunachalam E, Ge Z, Wang N, Deng X, Jin G, Cong W. Loss of FBP1 facilitates aggressive features of hepatocellular carcinoma cells through the Warburg effect. Carcinogenesis. 2017 Feb 1;38(2):134-43.<br>• Hirata H, Sugimachi K, Komatsu H, Ueda M, Masuda T, Uchi R, Sakimura S, Nambara S, Saito T, Shinden Y, Iguchi T. Decreased expression of fructose-1, 6-bisphosphatase associates with glucose metabolism and tumor progression in hepatocellular carcinoma. Cancer research. 2016 Jun 1;76(11):3265-76.
7.	SDC2	syndecan 2	2.061122	0.005596	1	• Osteosarcoma<br>• Hepatitis C Virus Infection<br>• Colorectal Cancer	• Modrowski D, Orosco A, Thévenard J, Fromigué O, Marie PJ. Syndecan-2 overexpression induces osteosarcoma cell apoptosis: implication of syndecan-2 cytoplasmic domain and JNK signaling. Bone. 2005 Aug 1;37(2):180-9.<br>• Fan H, Qiao L, Kang KD, Fan J, Wei W, Luo G. Attachment and postattachment receptors important for hepatitis C virus infection and cell-to-cell transmission. Journal of virology. 2017 Jun 9;91(13):e00280-17.<br>• Zhao G, Li H, Yang Z, Wang Z, Xu M, Xiong S, Li S, Wu X, Liu X, Wang Z, Zhu Y. Multiplex methylated DNA testing in plasma with high sensitivity and specificity for colorectal cancer screening. Cancer medicine. 2019 Sep;8(12):5619-28.
8.	PDGFRA	platelet-derived growth factor receptor; alpha polypeptide	1.993987	0.003407	1	• Gastrointestinal Stromal Tumors<br>• Acute Myeloid Leukaemia	• Liu W, Zeng X, Yin Y, Li C, Yang W, Wan W, Shi L, Wang G, Tao K, Zhang P. Targeting the WEE1 kinase strengthens the antitumor activity of imatinib via promoting KIT autophagic degradation in gastrointestinal stromal tumors. Gastric Cancer. 2020 Jan;23(1):39-51.<br>• Coccaro N, Anelli L, Orsini P, Zagaria A, Minervini A, Impera L, Tota G, Minervini CF, Cumbo C, Parciante E, Coserva MR. A complex and cryptic intrachromosomal rearrangement generating the FIP1L1_PDGFRA in adult acute myeloid leukemia. Cancer genetics. 2019 Nov 1;239:8-12.
9.	CXCL8	chemokine (C-X-C motif) ligand 8	1.951685	0.033416	1	• Colon Adenocarcinoma<br>• Rheumatoid Arthritis	• Zhao QQ, Jiang C, Gao Q, Zhang YY, Wang G, Chen XP, Wu SB, Tang J. Gene expression and methylation profiles identified CXCL3 and CXCL8 as key genes for diagnosis and prognosis of colon adenocarcinoma. Journal of cellular physiology. 2020 May;235(5):4902-12.<br>• Sato H, Muraoka S, Kusunoki N, Masuoka S, Yamada S, Ogasawara H, Imai T, Akasaka Y, Tochigi N, Takahashi H, Tsuchiya K. Resistin upregulates chemokine production by fibroblast-like synoviocytes from patients with rheumatoid arthritis. Arthritis research & therapy. 2017 Dec;19(1):1-0.
10.	TRIB2	tribbles pseudokinase 2	1.910104	0.030882	1	• Myeloid leukemia<br>• Colorectal cancer	• Salomé M, Magee A, Yalla K, Chaudhury S, Sarrou E, Carmody RJ, Keeshan K. A Trib2-p38 axis controls myeloid leukaemia cell cycle and stress response signalling. Cell death & disease. 2018 Apr 18;9(5):1-7.

						• Liver cancer	• Hou Z, Guo K, Sun X, Hu F, Chen Q, Luo X, Wang G, Hu J, Sun L. TRIB2 functions as novel oncogene in colorectal cancer by blocking cellular senescence through AP4/p21 signaling. Molecular cancer. 2018 Dec;17(1):1-5.<br>• Guo S, Chen Y, Yang Y, Zhang X, Ma L, Xue X, Qiao Y, Wang J. TRIB2 modulates proteasome function to reduce ubiquitin stability and protect liver cancer cells against oxidative stress. Cell death & disease. 2021 Jan 7;12(1):1-8
11.	NOTCH3	notch 3	1.860591	0.010832	1	• Cerebral Autosomal Dominant Arteriopathy With Subcortical Infarcts And Leucoencephalopathy (CADASIL)<br>• Ovarian Cancer	• Yadav S, Bentley P, Srivastava P, Prasad K, Sharma P. The first Indian-origin family with genetically proven cerebral autosomal dominant arteriopathy with subcortical infarcts and leukoencephalopathy (CADASIL). Journal of Stroke and Cerebrovascular Diseases. 2013 Jan 1;22(1):28-31.<br>• Park JT, Li M, Nakayama K, Mao TL, Davidson B, Zhang Z, Kurman RJ, Eberhart CG, Shih IM, Wang TL. Notch3 gene amplification in ovarian cancer. Cancer research. 2006 Jun 15;66(12):6312-8.
12.	LUCAT1	lung cancer associated transcript 1 (non-protein coding)	1.804475	0.038373	1	• Cervical Cancer<br>• Breast Cancer<br>• Prostate Cancer	• Wang AH, Zhao JM, Du J, Pang QX, Wang MQ. Long noncoding RNA LUCAT1 promotes cervical cancer cell proliferation and invasion by upregulating MTA1. European Review for Medical and Pharmacological Sciences. 2020 Sep 1;24(17):8623-.<br>• Li YL, Wang XM, Qiao GD, Zhang S, Wang J, Cong YZ, Zhu SG. Up-regulated lnc-lung cancer associated transcript 1 enhances cell migration and invasion in breast cancer progression. Biochemical and biophysical research communications. 2020 Jan 8;521(2):271-8.<br>• Liu C, Wang L, Li YW, Cui YS, Wang YQ, Liu S. Long noncoding RNA LUCAT1 promotes migration and invasion of prostate cancer cells by inhibiting KISS1 expression. Eur Rev Med Pharmacol Sci. 2019 Apr 1;23(8):3277-83.
13.	SHISA4	shisa family member 4	1.799915	0.047915	1	• NA	• NA
14.	GTSF1	gametocyte specific factor 1	1.712871	0.048731	1	• Liver Cancer	• Gao DY, Ling Y, Lou XL, Wang YY, Liu LM. GTSF1 gene may serve as a novel potential diagnostic biomarker for liver cancer. Oncology letters. 2018 Mar 1;15(3):3133-40.
15.	ENKD1	enkurin domain containing 1	1.699913	0.032605	1	• Non-small cell lung cancer (NSCLC)	• Song T, Zhou P, Sun C, He N, Li H, Ran J, Zhou J, Wu Y, Liu M. Enkurin domain containing 1 (ENKD1) regulates the proliferation, migration and invasion of non-small cell lung cancer cells. Asia-Pacific Journal of Clinical Oncology. 2021 Mar 15.
16.	MEP1A	meprin A; alpha (PABA peptide hydrolase)	1.696078	0.037797	1	• Colorectal Cancer<br>• Inflammatory Bowel Disease	• Wang X, Chen J, Wang J, Yu F, Zhao S, Zhang Y, Tang H, Peng Z. Correction to: Metalloproteases meprin-α (MEP1A) is a prognostic biomarker and promotes proliferation and invasion of colorectal cancer. BMC cancer. 2018 Dec;18(1):1-2.<br>• Banerjee S, Oneda B, Yap LM, Jewell DP, Matters GL, Fitzpatrick LR, Seibold F, Sterchi EE, Ahmad T, Lottaz D, Bond JS. MEP1A allele for meprin A metalloprotease is a susceptibility gene for inflammatory bowel disease. Mucosal immunology. 2009 May;2(3):220-31.
17.	ZMYND19	zinc finger; MYND-type containing 19	1.597825	0.038092	1	• Hepatocellular Carcinoma	• Zhu Q, Luo Z, Lu G, Gui F, Wu J, Li F, Ni Y. LncRNA FABP5P3/miR-589-5p/ZMYND19 axis contributes to hepatocellular carcinoma cell proliferation, migration and invasion. Biochemical and biophysical research communications. 2018 Apr 6;498(3):551-8.

18.	NCF2	neutrophil cytosolic factor 2	1.567903	0.028205	1	• Chronic Granulomatous Disease<br>• Liver Fibrosis	• Vignesh P, Rawat A, Kumar A, Suri D, Gupta A, Lau YL, Chan KW, Singh S. Chronic granulomatous disease due to neutrophil cytosolic factor (NCF2) gene mutations in three unrelated families. Journal of clinical immunology. 2017 Feb;37(2):109-12.<br>• Kong M, Chen X, Lv F, Ren H, Fan Z, Qin H, Yu L, Shi X, Xu Y. Serum response factor (SRF) promotes ROS generation and hepatic stellate cell activation by epigenetically stimulating NCF1/2 transcription. Redox biology. 2019 Sep 1;26:101302.
19.	ARG2	arginase 2	1.514896	0.033111	1	• Asthma<br>• Multiple Sclerosis	• Vonk JM, Postma DS, Maarsingh H, Bruinenberg M, Koppelman GH, Meurs H. Arginase 1 and arginase 2 variations associate with asthma, asthma severity and β2 agonist and steroid response. Pharmacogenetics and genomics. 2010 Mar 1;20(3):179-86.<br>• Cha L, Jones AP, Trend S, Byrne SN, Fabis-Pedrini MJ, Carroll WM, Lucas RM, Cole JM, Booth DR, Kermode AG, Hart PH. Tryptophan and arginine catabolic enzymes and regulatory cytokines in clinically isolated syndrome and multiple sclerosis. Clinical & translational immunology. 2018;7(8):e1037.
20.	TNKS1BP1	tankyrase 1 binding protein 1; 182kDa	1.501487	0.014716	1	• Cancer Cell Invasion<br>• Lung Adenocarcinoma	• Ohishi T, Yoshida H, Katori M, Migita T, Muramatsu Y, Miyake M, Ishikawa Y, Saiura A, Iemura SI, Natsume T, Seimiya H. Tankyrase-binding protein TNKS1BP1 regulates actin cytoskeleton rearrangement and cancer cell invasion. Cancer research. 2017 May 1;77(9):2328-38.<br>• Tan W, Guan H, Zou LH, Wang Y, Liu XD, Rang WQ, Zhou PK, Pei HD, Zhong CG. Overexpression of TNKS1BP1 in lung cancers and its involvement in homologous recombination pathway of DNA double-strand breaks. Cancer medicine. 2017 Feb;6(2):483-93.
21.	TNFRSF10C	tumor necrosis factor receptor superfamily; member 10c; decoy without an intracellular domain	-1.50106	0.030787	1	• Cervical Cancer<br>• Pancreatic Cancer	• Narayan G, Xie D, Ishdorj G, Scotto L, Mansukhani M, Pothuri B, Wright JD, Kaufmann AM, Schneider A, Arias-Pulido H, Murty VV. Epigenetic inactivation of TRAIL decoy receptors at 8p12-21.3 commonly deleted region confers sensitivity to A po2L/trail-C isplatin combination therapy in cervical cancer. Genes, Chromosomes and Cancer. 2016 Feb;55(2):177-89.<br>• Cai HH, Sun YM, Miao Y, Gao WT, Peng Q, Yao J, Zhao HL. Aberrant methylation frequency of TNFRSF10C promoter in pancreatic cancer cell lines. Hepatobiliary & Pancreatic Diseases International. 2011 Feb 1;10(1):95-100.
22.	NOD1	nucleotide-binding oligomerization domain containing 1	-1.5529	0.022004	1	• Crohn's Disease<br>• Inflammatory Bowel Disease<br>• Periodontitis<br>• Asthma	• Sorbara MT, Ellison LK, Ramjeet M, Travassos LH, Jones NL, Girardin SE, Philpott DJ. The protein ATG16L1 suppresses inflammatory cytokines induced by the intracellular sensors Nod1 and Nod2 in an autophagy-independent manner. Immunity. 2013 Nov 14;39(5):858-73.<br>• Lu Y, Zheng Y, Coyaud É, Zhang C, Selvabaskaran A, Yu Y, Xu Z, Weng X, Chen JS, Meng Y, Warner N. Palmitoylation of NOD1 and NOD2 is required for bacterial sensing. Science. 2019 Oct 25;366(6464):460-7.<br>• Loos BG, Fiebig A, Nothnagel M, Jepsen S, Groessner-Schreiber B, Franke A, Jervøe-Storm PM, Schenck K, Van der Velden U, Schreiber S. NOD1 gene polymorphisms in relation to aggressive periodontitis. Innate immunity. 2009 Aug;15(4):225-32.<br>• Hysi P, Kabesch M, Moffatt MF, Schedel M, Carr D, Zhang Y, Boardman B, Von Mutius E, Weiland SK, Leupold W, Fritzsch C.

							NOD1 variation, immunoglobulin E and asthma. Human molecular genetics. 2005 Apr 1;14(7):935-41.
23.	BIVM-ERCC5	BIVM-ERCC5 readthrough	-1.55677	0.036419	1	• Xeroderma Pigmentosum<br>• Breast Cancer	• uspikel T, Clarkson SG. Mutations that disable the DNA repair gene XPG in a xeroderma pigmentosum group G patient. Human molecular genetics. 1994 Jun 1;3(6):963-7.<br>• Ge J, Liu H, Qian D, Wang X, Moorman PG, Luo S, Hwang S, Wei Q. Genetic variants of genes in the NER pathway associated with risk of breast cancer: A large-scale analysis of 14 published GWAS datasets in the DRIVE study. International journal of cancer. 2019 Sep 1;145(5):1270-9.
24.	HSPA2	heat shock 70kDa protein 2	-1.60919	0.006893	1	• Renal Cell Carcinoma<br>• Ovarian Cancer	• Gaudin C, Kremer F, Angevin E, Scott V, Triebel F. A hsp70-2 mutation recognized by CTL on a human renal cell carcinoma. The Journal of Immunology. 1999 Feb 1;162(3):1730-8.<br>• Gupta N, Jagadish N, Surolia A, Suri A. Heat shock protein 70-2 (HSP70-2) a novel cancer testis antigen that promotes growth of ovarian cancer. American journal of cancer research. 2017;7(6):1252.
25.	Mar-03	membrane-associated ring finger (C3HC4) 3; E3 ubiquitin protein ligase	-1.62811	0.027437	1	• Alzheimer's Disease	• Von Rotz RC, Kins S, Hipfel R, Von Der Kammer H, Nitsch RM. The novel cytosolic RING finger protein dactylidin is up-regulated in brains of patients with Alzheimer's disease. European Journal of Neuroscience. 2005 Mar;21(5):1289-98.
26.	NET1	neuroepithelial cell transforming 1	-1.64547	0.047965	1	• Malignant neoplasm of breast	• Dutertre M, Gratadou L, Dardenne E, Germann S, Samaan S, Lidereau R, Driouch K, de la Grange P, Auboeuf D. Estrogen regulation and physiopathologic significance of alternative promoters in breast cancer. Cancer research. 2010 May 1;70(9):3760-70.
27.	GADD45A	growth arrest and DNA-damage-inducible; alpha	-1.68291	0.026549	1	• Ovarian Cancer<br>• Prostate Cancer<br>• Breast Cancer	• Jiang F, Li P, Fornace AJ, Nicosia SV, Bai W. G2/M arrest by 1, 25-dihydroxyvitamin D3 in ovarian cancer cells mediated through the induction of GADD45 via an exonic enhancer. Journal of Biological Chemistry. 2003 Nov 28;278(48):48030-40.<br>• Ramachandran K, Gopisetty G, Gordian E, Navarro L, Hader C, Reis IM, Schulz WA, Singal R. Methylation-mediated repression of GADD45α in prostate cancer and its role as a potential therapeutic target. Cancer research. 2009 Feb 15;69(4):1527-35.<br>• Sensi E, Tancredi M, Aretini P, Cipollini G, Collecchi P, Naccarato AG, Viacava P, Bevilacqua G, Caligo MA. Clinicopathological significance of GADD45 gene alterations in human familial breast carcinoma. Breast cancer research and treatment. 2004 Sep;87(2):197-201.
28.	SOCS3	suppressor of cytokine signaling 3	-1.69345	0.003628	1	• Hepatocellular Carcinoma<br>• Type 2 Diabetes Mellitus<br>• Polycystic Ovary Syndrome	• Jiang BG, Wang N, Huang J, Yang Y, Sun LL, Pan ZY, Zhou WP. Tumor SOCS3 methylation status predicts the treatment response to TACE and prognosis in HCC patients. Oncotarget. 2017 Apr 25;8(17):28621.<br>• Liu X, Qian X, Tu R, Mao Z, Huo W, Zhang H, Jiang J, Zhang X, Tian Z, Li Y, Wang C. SOCS3 methylation mediated the effect of sedentary time on type 2 diabetes mellitus: The Henan Rural Cohort study. Nutrition, Metabolism and Cardiovascular Diseases. 2020 Apr 12;30(4):634-43.<br>• González F, Considine RV, Abdelhadi OA, Acton AJ. Saturated fat ingestion promotes lipopolysaccharide-mediated inflammation and insulin resistance in polycystic ovary syndrome. The Journal of Clinical Endocrinology & Metabolism. 2019 Mar;104(3):934-46.

29.	FAM134B	family with sequence similarity 134; member B	-1.86998	0.022027	1	• Hereditary sensory and autonomic neuropathy type II (HSAN II)<br>• Colorectal Cancer	• Kurth I, Pamminger T, Hennings JC, Soehendra D, Huebner AK, Rotthier A, Baets J, Senderek J, Topaloglu H, Farrell SA, Nürnberg G. Mutations in FAM134B, encoding a newly identified Golgi protein, cause severe sensory and autonomic neuropathy. Nature genetics. 2009 Nov;41(11):1179-81.<br>• Kasem K, Gopalan V, Salajegheh A, Lu CT, Smith RA, Lam AK. The roles of JK-1 (FAM134B) expressions in colorectal cancer. Experimental cell research. 2014 Aug 1;326(1):166-73.
30.	ANKRD22	ankyrin repeat domain 22	-1.91806	0.002439	1	• Prostate Cancer<br>• Lung Cancer	• Qiu Y, Yang S, Pan T, Yu L, Liu J, Zhu Y, Wang H. ANKRD22 is involved in the progression of prostate cancer. Oncology letters. 2019 Oct 1;18(4):4106-13.<br>• Yin J, Fu W, Dai L, Jiang Z, Liao H, Chen W, Pan L, Zhao J. ANKRD22 promotes progression of non-small cell lung cancer through transcriptional up-regulation of E2F1. Scientific reports. 2017 Jun 30;7(1):1-1.
31.	ALDH1A2	aldehyde dehydrogenase 1 family; member A2	-2.12572	0.017627	1	• Schizophrenia,<br>• Prostatic neoplasm,<br>• Degenerative polyarthritis	• Chan RF, Shabalin AA, Montano C, Hannon E, Hultman CM, Fallin MD, Feinberg AP, Mill J, Van Den Oord EJ, Aberg KA. Independent methylome-wide association studies of schizophrenia detect consistent case–control differences. Schizophrenia bulletin. 2020 Feb 26;46(2):319-27.<br>• Kim H, Lapointe J, Kaygusuz G, Ong DE, Li C, van de Rijn M, Brooks JD, Pollack JR. The retinoic acid synthesis gene ALDH1a2 is a candidate tumor suppressor in prostate cancer. Cancer research. 2005 Sep 15;65(18):8118-24.<br>• Shepherd C, Zhu D, Skelton AJ, Combe J, Threadgold H, Zhu L, Vincent TL, Stuart P, Reynard LN, Loughlin J. Functional characterization of the osteoarthritis genetic risk residing at ALDH1A2 identifies rs12915901 as a key target variant. Arthritis & Rheumatology. 2018 Oct;70(10):1577-87.
32.	RNASEK-C17orf49	RNASEK-C17orf49 readthrough	-2.15793	0.025219	1	• Prostate Cancer	• Sun S, Zhong X, Wang C, Sun H, Wang S, Zhou T, Zou R, Lin L, Sun N, Sun G, Wu Y. BAP18 coactivates androgen receptor action and promotes prostate cancer progression. Nucleic acids research. 2016 Sep 30;44(17):8112-28.
33.	GPX8	glutathione peroxidase 8 (putative)	-2.57192	0.015076	1	• NA	• NA
34.	FCAMR	Fc receptor; IgA; IgM; high affinity	-2.87411	0.034976	1	• Coronary Arteriosclerosis	• Ward-Caviness CK, Neas LM, Blach C, Haynes CS, LaRocque-Abramson K, Grass E, Dowdy ZE, Devlin RB, Diaz-Sanchez D, Cascio WE, Miranda ML. A genome-wide trans-ethnic interaction study links the PIGR-FCAMR locus to coronary atherosclerosis via interactions between genetic variants and residential exposure to traffic. PloS one. 2017 Mar 29;12(3):e0173880.
35.	LBP	lipopolysaccharide binding protein	-3.08656	0.032509	1	• Chronic Hepatitis C<br>• Sepsis	• Nien HC, Hsu SJ, Su TH, Yang PJ, Sheu JC, Wang JT, Chow LP, Chen CL, Kao JH, Yang WS. High serum lipopolysaccharide-binding protein level in chronic hepatitis C viral infection is reduced by anti-viral treatments. PloS one. 2017 Jan 20;12(1):e0170028.<br>• Zeng L, Gu W, Zhang AQ, Zhang M, Zhang LY, Du DY, Huang SN, Jiang JX. A functional variant of lipopolysaccharide binding protein predisposes to sepsis and organ dysfunction in patients with major trauma. Annals of surgery. 2012 Jan 1;255(1):147-57.
36.	SNORD3A	small nucleolar RNA; C/D box 3A	-3.35462	0.001409	1	• Creutzfeldt-Jakob disease	• Cohen E, Avrahami D, Frid K, Canello T, Levy Lahad E, Zeligson S, Perlberg S, Chapman J, Cohen OS, Kahana E, Lavon I. Snord 3A: a

							molecular marker and modulator of prion disease progression. PLoS One. 2013 Jan 21;8(1):e54433.
37.	LY75-CD302	LY75-CD302 readthrough	-145.615	3.46E-05	0.144833	• Familial classical Hodgkin lymphoma	• Ristolainen H, Kilpivaara O, Kamper P, Taskinen M, Saarinen S, Leppä S, d'Amore F, Aaltonen LA. Identification of homozygous deletion in ACAN and other candidate variants in familial classical Hodgkin lymphoma by exome sequencing.
38.	EEF1E1-BLOC1S5	EEF1E1-BLOC1S5 readthrough (NMD candidate)	-231.847	1.54E-05	0.096851	• Cystic fibrosis of lung airway and parenchyma tissues	• Kumar P, Sen C, Peters K, Frizzell RA, Biswas R. Comparative analyses of long non-coding RNA profiles in vivo in cystic fibrosis lung airway and parenchyma tissues. Respiratory research. 2019 Dec;20(1):1-1.
39.	RGPD8	RANBP2-like and GRIP domain containing 8	-757.257	0.001271	1	• NA	• NA

Table 6: Top 100 genes affected in IFN-λ4 vs IFN-λ3 (M2-MDM)

S.No	Gene symbol	Name	Foldchange	PValue	FDR	Associated Disease	Reference
1.	KIAA1644	KIAA1644	42.57416	4.67E-05	0.001079	• Spontaneous preterm birth (SPTB)	• Tiensuu H, Haapalainen AM, Karjalainen MK, Pasanen A, Huusko JM, Marttila R, Ojaniemi M, Muglia LJ, Hallman M, Rämet M. Risk of spontaneous preterm birth and fetal growth associates with fetal SLIT2. PLoS genetics. 2019 Jun 13;15(6):e1008107.
2.	CCL19	chemokine (C-C motif) ligand 19	28.38406	4.08E-11	1.56E-08	• Pneumonia	• Hoffmann-Vold AM, Hesselstrand R, Fretheim H, Ueland T, Andreassen AK, Brunborg C, Palchevskiy V, Midtvedt Ø, Garen T, Aukrust P, Belperio JA. CCL21 as a potential serum biomarker for pulmonary arterial hypertension in systemic sclerosis. Arthritis & Rheumatology. 2018 Oct;70(10):1644-53.
3.	MMP10	matrix metallopeptidase 10	15.67925	8.99E-11	3.14E-08	• Asthma	• Kuo CH, Pavlidis S, Zhu J, Loza M, Baribaud F, Rowe A, Pandis I, Gibeon D, Hoda U, Sousa A, Wilson SJ. Contribution of airway eosinophils in airway wall remodeling in asthma: Role of MMP-10 and MET. Allergy. 2019 Jun;74(6):1102-12.
4.	IRF4	interferon regulatory factor 4	15.59398	1.45E-11	6.30E-09	• Chronic lymphocytic leukemia	• Asslaber D, Qi Y, Maeding N, Steiner M, Denk U, Höpner JP, Hartmann TN, Zaborsky N, Greil R, Egle A. B-cell–specific IRF4 deletion accelerates chronic lymphocytic leukemia development by enhanced tumor immune evasion. blood. 2019 Nov 14;134(20):1717-29.
5.	ADAM19	ADAM metallopeptidase domain 19	14.48353	1.44E-08	1.81E-06	• COPD<br>• Colorectal Carcinoma	• Sakornsakolpat P, Prokopenko D, Lamontagne M, Reeve NF, Guyatt AL, Jackson VE, Shrine N, Qiao D, Bartz TM, Kim DK, Lee MK. Genetic landscape of chronic obstructive pulmonary disease identifies heterogeneous cell-type and phenotype associations. Nature genetics. 2019 Mar;51(3):494-505.<br>• Zhang Q, Yu L, Qin D, Huang R, Jiang X, Zou C, Tang Q, Chen Y, Wang G, Wang X, Gao X. Role of microRNA-30c targeting ADAM19 in colorectal cancer. PLoS One. 2015 Mar 23;10(3):e0120698.
6.	GJA4	gap junction protein; alpha 4; 37kDa	12.92545	0.001052	0.010261	• Coronary artery disease	• Han Y, Xi S, Zhang X, Yan C, Yang Y, Kang J. Association of connexin 37 gene polymorphisms with risk of coronary artery disease in northern Han Chinese. Cardiology. 2008;110(4):260-5.

7.	NR4A3	nuclear receptor subfamily 4; group A; member 3	12.44311	1.04E-08	1.40E-06	• Extraskeletal Myxoid Chondrosarcoma	• Urbini M, Astolfi A, Pantaleo MA, Serravalle S, Dei Tos AP, Picci P, Indio V, Sbaraglia M, Benini S, Righi A, Gambarotti M. HSPA 8 as a novel fusion partner of NR 4 A 3 in extraskeletal myxoid chondrosarcoma. Genes, Chromosomes and Cancer. 2017 Jul;56(7):582-6.
8.	EDN1	endothelin 1	12.05188	7.98E-16	1.55E-12	• Auriculo-condylar syndrome	• Gordon CT, Petit F, Kroisel PM, Jakobsen L, Zechi-Ceide RM, Oufadem M, Bole-Feysot C, Pruvost S, Masson C, Tores F, Hieu T. Mutations in endothelin 1 cause recessive auriculocondylar syndrome and dominant isolated question-mark ears. The American Journal of Human Genetics. 2013 Dec 5;93(6):1118-25.
9.	SLC8A3 (NCX-3)	solute carrier family 8 (sodium/calcium exchanger); member 3	11.72561	1.71E-06	8.04E-05	• Seizures	• Martinez Y, N'Gouemo P. Blockade of the sodium calcium exchanger exhibits anticonvulsant activity in a pilocarpine model of acute seizures in rats. Brain research. 2010 Dec 17;1366:211-6.
10.	NTN1	netrin 1	11.67039	8.54E-07	4.62E-05	• Subarachnoid Hemorrhage	• Xie Z, Huang L, Enkhjargal B, Reis C, Wan W, Tang J, Cheng Y, Zhang JH. Intranasal administration of recombinant Netrin-1 attenuates neuronal apoptosis by activating DCC/APPL-1/AKT signaling pathway after subarachnoid hemorrhage in rats. Neuropharmacology. 2017 Jun 1;119:123-33.
11.	CCR7	chemokine (C-C motif) receptor 7	11.39522	5.35E-08	5.42E-06	• Adult T-cell leukemia	• Kataoka K, Nagata Y, Kitanaka A, Shiraishi Y, Shimamura T, Yasunaga JI, Totoki Y, Chiba K, Sato-Otsubo A, Nagae G, Ishii R. Integrated molecular analysis of adult T cell leukemia/lymphoma. Nature genetics. 2015 Nov;47(11):1304-15.
12.	CAMK2A	calcium/calmodulin-dependent protein kinase II alpha	11.3648	0.000359	0.004666	• Bipolar disorder	• Li H, Zhou DS, Chang H, Wang L, Liu W, Dai SX, Zhang C, Cai J, Liu W, Li X, Fan W. Interactome analyses implicated CAMK2A in the genetic predisposition and pharmacological mechanism of bipolar disorder. Journal of psychiatric research. 2019 Aug 1;115:165-75.
13.	LINC00158	long intergenic non-protein coding RNA 158	10.32393	6.13E-07	3.46E-05	• Endometrial Carcinoma	• Cai Y, Hao M, Chang Y, Liu Y. Up-Regulation of LINC00158 Contributes to Endometrial Carcinoma Progression by Interacting with HMGB2.
14.	LOC440896	NA	10.22915	1.85E-07	1.40E-05	• Neuroblastoma	• Sathipati SY, Sahu D, Huang HC, Lin Y, Ho SY. Identification and characterization of the lncRNA signature associated with overall survival in patients with neuroblastoma. Scientific reports. 2019 Mar 26;9(1):1-3.
15.	CHST3	carbohydrate (chondroitin 6) sulfotransferase 3	10.09935	0.001719	0.014823	• Humero-Spinal Dysostosis<br>• Lumbar Disc Degeneration	• Hermanns P, Unger S, Rossi A, Perez-Aytes A, Cortina H, Bonafé L, Boccone L, Setzu V, Dutoit M, Sangiorgi L, Pecora F. Congenital joint dislocations caused by carbohydrate sulfotransferase 3 deficiency in recessive Larsen syndrome and humero-spinal dysostosis. The American Journal of Human Genetics. 2008 Jun 6;82(6):1368-74.<br>• Song YQ, Karasugi T, Cheung KM, Chiba K, Ho DW, Miyake A, Kao PY, Sze KL, Yee A, Takahashi A, Kawaguchi Y. Lumbar disc degeneration is linked to a carbohydrate sulfotransferase 3 variant. The Journal of clinical investigation. 2013 Nov 1;123(11):4909-17.
16.	FCAMR	Fc receptor; IgA; IgM; high affinity	9.784227	2.99E-05	0.000768	• Coronary Arteriosclerosis	• Ward-Caviness CK, Neas LM, Blach C, Haynes CS, LaRocque-Abramson K, Grass E, Dowdy ZE, Devlin RB, Diaz-Sanchez D, Cascio WE, Miranda ML. A genome-wide trans-ethnic interaction study links the PIGR-FCAMR locus to coronary atherosclerosis via interactions between genetic variants and residential exposure to traffic. PloS one. 2017 Mar 29;12(3):e0173880.

17.	SYNPO2	synaptopodin 2	9.025416	2.92E-14	4.08E-11	• Atrial fibrillation	• Nielsen JB, Thorolfsdottir RB, Fritsche LG, Zhou W, Skov MW, Graham SE, Herron TJ, McCarthy S, Schmidt EM, Sveinbjornsson G, Surakka I. Biobank-driven genomic discovery yields new insight into atrial fibrillation biology. Nature genetics. 2018 Sep;50(9):1234-9.
18.	FSCN1	fascin actin-bundling protein 1	8.489981	2.61E-09	4.83E-07	• Breast Carcinoma	• Tampaki EC, Tampakis A, Nonni A, von Flüe M, Patsouris E, Kontzoglou K, Kouraklis G. Combined Fascin-1 and MAP17 expression in breast cancer identifies patients with high risk for disease recurrence. Molecular diagnosis & therapy. 2019 Oct;23(5):635-44.
19.	SLCO5A1	solute carrier organic anion transporter family; member 5A1	8.085139	1.63E-08	1.99E-06	• Mesomelia-synostoses syndrome (MSS)	• Kohmoto T, Naruto T, Watanabe M, Fujita Y, Ujiro S, Okamoto N, Horikawa H, Masuda K, Imoto I. A 590 kb deletion caused by non-allelic homologous recombination between two LINE-1 elements in a patient with mesomelia-synostosis syndrome. American Journal of Medical Genetics Part A. 2017 Apr;173(4):1082-6.
20.	CHKB-CPT1B	CHKB-CPT1B readthrough (NMD candidate)	7.938763	0.046143	0.155414	• Narcolepsy<br>• Metabolic Syndrome X	• Miyagawa T, Kawashima M, Nishida N, Ohashi J, Kimura R, Fujimoto A, Shimada M, Morishita S, Shigeta T, Lin L, Hong SC. Variant between CPT1B and CHKB associated with susceptibility to narcolepsy. Nature genetics. 2008 Nov;40(11):1324-8.<br>• Auinger A, Rubin D, Sabandal M, Helwig U, Rüther A, Schreiber S, Foelsch UR, Döring F, Schrezenmeir J. A common haplotype of carnitine palmitoyltransferase 1b is associated with the metabolic syndrome. British journal of nutrition. 2013 Mar;109(5):810-5.
21.	UBD	ubiquitin D	7.937495	4.54E-09	7.60E-07	• Kidney diseases	• Zhang JY, Wang M, Tian L, Genovese G, Yan P, Wilson JG, Thadhani R, Mottl AK, Appel GB, Bick AG, Sampson MG. UBD modifies APOL1-induced kidney disease risk. Proceedings of the National Academy of Sciences. 2018 Mar 27;115(13):3446-51.
22.	LAMP3	lysosomal-associated membrane protein 3	7.684912	2.67E-14	4.08E-11	• Parkinson disease	• Li NN, Tan EK, Chang XL, Mao XY, Zhao DM, Zhang JH, Liao Q, Peng R. MCCC 1/LAMP 3 reduces risk of sporadic P arkinson's disease in Han Chinese. Acta Neurologica Scandinavica. 2013 Aug;128(2):136-9.
23.	ADIRF	adipogenesis regulatory factor	7.60691	7.51E-06	0.000269	• Prostate carcinoma	• Meng J, Wang LH, Zou CL, Dai SM, Zhang J, Lu Y. C10orf116 gene copy number loss in prostate cancer: clinicopathological correlations and prognostic significance. Medical science monitor: international medical journal of experimental and clinical research. 2017;23:5176.
24.	SLIT2	slit guidance ligand 2	7.242537	0.000133	0.002264	• Liver carcinoma	• Sun G, Zhang C, Feng M, Liu W, Xie H, Qin Q, Zhao E, Wan L. Methylation analysis of p16, SLIT2, SCARA5, and Runx3 genes in hepatocellular carcinoma. Medicine. 2017 Oct;96(41).
25.	TMEM97 (Sigma 2 receptor)	transmembrane protein 97	6.991606	3.15E-07	2.02E-05	• Malignant neoplasms	• Alamri MA, Ates-Alagoz Z, Adejare A. Bicycloheptylamine-Doxorubicin Conjugate: Synthesis and Anticancer Activities in σ2 Receptor-Expressing Cell Lines. Medicinal Chemistry. 2020 Mar 1;16(2):192-201
26.	SERPINB7	serpin peptidase inhibitor; clade B (ovalbumin); member 7	6.695769	0.001797	0.015289	• Nagashima-type palmoplantar keratoderma	• Hashimoto T, Teye K, Numata S, Suga Y, Hamada T, Ishii N. Detection of SERPINB 7 mutation can distinguish Nagashima-type palmoplantar keratoderma from other keratodermas with palmoplantar lesions. Clinical and experimental dermatology. 2017 Apr;42(3):342-5.
27.	ADAM11	ADAM metallopeptidase domain 11	6.688311	1.37E-06	6.73E-05	• Neoplasms	• Wu E, Croucher PI, McKie N. Expression of members of the novel membrane linked metalloproteinase family ADAM in cells derived from a range of haematological malignancies. Biochemical and biophysical research communications. 1997 Jun 18;235(2):437-42.

28.	SRGAP1	SLIT-ROBO Rho GTPase activating protein 1	6.302447	2.64E-05	0.000713	• Papillary thyroid carcinoma (PTC)	• He H, Bronisz A, Liyanarachchi S, Nagy R, Li W, Huang Y, Akagi K, Saji M, Kula D, Wojcicka A, Sebastian N. SRGAP1 is a candidate gene for papillary thyroid carcinoma susceptibility. The Journal of Clinical Endocrinology & Metabolism. 2013 May 1;98(5):E973-80.
29.	COL1A2	collagen; type I; alpha 2	6.017135	0.019178	0.083048	• Osteogenesis imperfecta type III	• Augusciak-Duma A, Witecka J, Sieroń AL, Janeczko M, Pietrzyk JJ, Ochman K, Galicka A, Borszewska-Kornacka MK, Pilch J, Jakubowska-Pietkiewicz E. Mutations in COL1A1 and COL1A2 Genes Associated with Osteogenesis Imperfecta (OI) Types I or III. Acta Biochimica Polonica. 2018 May 27;65(1):79-86.
30.	ETV3L	ets variant 3-like	5.956911	1.05E-07	8.97E-06	• Currarino Syndrome	• Holm I, Spildrejorde M, Stadheim B, Eiklid KL, Samarakoon PS. Whole exome sequencing of sporadic patients with Currarino Syndrome: a report of three trios. Gene. 2017 Aug 15;624:50-5
31.	TMCC2	transmembrane and coiled-coil domain family 2	5.726476	0.001075	0.010438	• Neurodegeneration	• Hopkins PC. Neurodegeneration in a Drosophila model for the function of TMCC2, an amyloid protein precursor-interacting and apolipoprotein E-binding protein. PLoS One. 2013 Feb 7;8(2):e55810.
32.	GUCY1A3	guanylate cyclase 1; soluble; alpha 3	5.666615	2.75E-07	1.84E-05	• Moyamoya disease (MMD)	• Wallace S, Guo DC, Regalado E, Mellor-Crummey L, Bamshad M, Nickerson DA, Dauser R, Hanchard N, Marom R, Martin E, Berka V. Disrupted nitric oxide signaling due to GUCY1A3 mutations increases risk for moyamoya disease, achalasia and hypertension. Clinical genetics. 2016 Oct;90(4):351-60.
33.	CCL22	chemokine (C-C motif) ligand 22	5.481067	5.21E-05	0.001155	• Atopic dermatitis	• Miake S, Tsuji G, Takemura M, Hashimoto-Hachiya A, Vu YH, Furue M, Nakahara T. IL-4 augments IL-31/IL-31 receptor alpha interaction leading to enhanced Ccl 17 and Ccl 22 production in dendritic cells: implications for atopic dermatitis. International journal of molecular sciences. 2019 Jan;20(16):4053.
34.	UPB1	ureidopropionase; beta	5.390989	0.000254	0.003661	• B-Ureidopropionase Deficienc	• Shu J, Lv X, Jiang S, Zhang Y, Zhang C, Meng Y, Situ A, Xu H, Song L. Genetic analysis of the UPB1 gene in two new Chinese families with β-ureidopropionase deficiency and the carrier frequency of the mutation c. 977G> A in Northern China. Child's Nervous System. 2014 Dec 1;30(12):2109-14.
35.	LSP1	lymphocyte-specific protein 1	5.375649	8.21E-19	2.58e-15	• Breast Cancer	• Chen MB, Li C, Shen WX, Guo YJ, Shen W, Lu PH. Association of a LSP1 gene rs3817198T> C polymorphism with breast cancer risk: evidence from 33,920 cases and 35,671 controls. Molecular biology reports. 2011 Oct;38(7):4687-95.
36.	ADAMTS4	ADAM metallopeptidase with thrombospondin type 1 motif; 4	5.369273	0.000285	0.003965	• Degenerative polyarthritis<br>• Alzheimer's disease	• Meng P, Zhang Y, Wei H, Tan S, Guo X, Wang S, Yu Y. ADAMTS4 and ADAMTS5 may be considered as new molecular therapeutic targets for cartilage damages with Kashin-Beck Disease. Medical hypotheses. 2020 Feb 1;135:109440.<br>• Walter S, Jumpertz T, Hüttenrauch M, Ogorek I, Gerber H, Storck SE, Zampar S, Dimitrov M, Lehmann S, Lepka K, Berndt C. The metalloprotease ADAMTS4 generates N-truncated Aβ4–x species and marks oligodendrocytes as a source of amyloidogenic peptides in Alzheimer's disease. Acta neuropathologica. 2019 Feb;137(2):239-57.
37.	PRLR	prolactin receptor	5.311721	3.36E-10	8.80E-08	• Colon cancer	• Bhatavdekar J, Patel D, Ghosh N, Vora H, Shah N, Karelia N, Balar D, Chikhlikar P, Dave R. Interrelationship of prolactin and its receptor in carcinoma of colon and rectum: a preliminary report. Journal of surgical oncology. 1994 Apr;55(4):246-9.

38.	LINC00996	long intergenic non-protein coding RNA 996	5.295232	0.002205	0.017851	• Colorectal Cancer	• Ge H, Yan Y, Di Wu YH, Tian F. Potential role of LINC00996 in colorectal cancer: a study based on data mining and bioinformatics. OncoTargets and therapy. 2018;11:4845.
39.	BCL2L14	BCL2-like 14 (apoptosis facilitator)	5.170166	5.48E-08	5.51E-06	• Triple negative breast cancer	• Romero P, Benhamo V, Deniziaut G, Fuhrmann L, Berger F, Manié E, Bhalshankar J, Vacher S, Laurent C, Marangoni E, Gruel N. Medullary breast carcinoma, a triple-negative breast cancer associated with BCLG overexpression. The American journal of pathology. 2018 Oct 1;188(10):2378-91
40.	LUM	lumican	5.139085	0.002149	0.017477	• Liver cirrhosis,<br>• Fibrosis	• Ma J, Sanda M, Wei R, Zhang L, Goldman R. Quantitative analysis of core fucosylation of serum proteins in liver diseases by LC-MS-MRM. Journal of proteomics. 2018 Oct 30;189:67-74.<br>• Naito Z, Ishiwata T, Lu YP, Teduka K, Fujii T, Kawahara K, Sugisaki Y. Transient and ectopic expression of lumican by acinar cells in L-arginine-induced acute pancreatitis. Experimental and molecular pathology. 2003 Feb 1;74(1):33-9.
41.	ANKRD33B	ankyrin repeat domain 33B	5.122296	8.33E-07	4.55E-05	• NA	• NA
42.	PITPNM3	PITPNM family member 3	5.010004	0.002873	0.021678	• Breast Cancer<br>• Autosomal Dominant Cone Dystrophy	• Chen J, Yao Y, Gong C, Yu F, Su S, Chen J, Liu B, Deng H, Wang F, Lin L, Yao H. CCL18 from tumor-associated macrophages promotes breast cancer metastasis via PITPNM3. Cancer cell. 2011 Apr 12;19(4):541-55.<br>• Köhn L, Kadzhaev K, Burstedt MS, Haraldsson S, Hallberg B, Sandgren O, Golovleva I. Mutation in the PYK2-binding domain of PITPNM3 causes autosomal dominant cone dystrophy (CORD5) in two Swedish families. European journal of human genetics. 2007 Jun;15(6):664-71.
43.	EBI3	Epstein-Barr virus induced 3	4.937463	7.91E-14	7.64E-11	• Colorectal carcinoma	• Liang Y, Chen Q, Du W, Chen C, Li F, Yang J, Peng J, Kang D, Lin B, Chai X, Zhou K. Epstein-Barr virus-induced gene 3 (EBI3) blocking leads to induce antitumor cytotoxic T lymphocyte response and suppress tumor growth in colorectal cancer by bidirectional reciprocal-regulation STAT3 signaling pathway. Mediators of inflammation. 2016 May 10;2016.
44.	CXCL10	chemokine (C-X-C motif) ligand 10	4.900546	9.24E-05	0.001752	• Influenza	• To KK, Lu L, Fong CH, Wu AK, Mok KY, Yip CC, Ke YH, Sze KH, Lau SK, Hung IF, Yuen KY. Rhinovirus respiratory tract infection in hospitalized adult patients is associated with TH2 response irrespective of asthma. Journal of Infection. 2018 May 1;76(5):465-74.
45.	CXCL13	chemokine (C-X-C motif) ligand 13	4.900079	0.001167	0.01101	• Rheumatoid arthritis<br>• Breast carcinoma	• Bao YQ, Wang JP, Dai ZW, Mao YM, Wu J, Guo HS, Xia YR, Ye DQ. Increased circulating CXCL13 levels in systemic lupus erythematosus and rheumatoid arthritis: a meta-analysis. Clinical rheumatology. 2020 Jan;39(1):281-90.<br>• Razis E, Kalogeras KT, Kotsantis I, Koliou GA, Manousou K, Wirtz R, Veltrup E, Patsea H, Poulakaki N, Dionysopoulos D, Pervana S. The role of CXCL13 and CXCL9 in early breast cancer. Clinical breast cancer. 2020 Feb 1;20(1):e36-53.
46.	TARP	TCR gamma alternate reading frame protein	4.89601	0.000336	0.00448	• Leukemia	• Poopak B, Saki N, Purfatholah AA, Najmabadi H, Mortazavi Y, Arzanian MT, Khosravipour G, Haghnejad F, Salari F, Shahjahani M. Pattern of immunoglobulin and T-cell receptor-δ/γ gene rearrangements in Iranian children with B-precursor acute lymphoblastic leukemia. Hematology. 2014 Jul 1;19(5):259-66.
47.	SLITRK5	SLIT and NTRK-like family; member 5	4.865018	0.008038	0.045042	• Obsessive-compulsive disorder (OCD)	• Song M, Mathews CA, Stewart SE, Shmelkov SV, Mezey JG, Rodriguez-Flores JL, Rasmussen SA, Britton JC, Oh YS, Walkup JT,

							Lee FS. Rare synaptogenesis-impairing mutations in SLITRK5 are associated with obsessive compulsive disorder. PloS one. 2017 Jan 13;12(1):e0169994.
48.	CXCL11	chemokine (C-X-C motif) ligand 11	4.799455	0.000307	0.004193	• Celiac disease	• Haghbin M, Rostami-Nejad M, Forouzesh F, Sadeghi A, Rostami K, Aghamohammadi E, Asadzadeh-Aghdaei H, Masotti A, Zali MR. The role of CXCR3 and its ligands CXCL10 and CXCL11 in the pathogenesis of celiac disease. Medicine. 2019 Jun;98(25).
49.	MIR155HG	MIR1 55 host gene	4.703522	1.14E-07	9.48E-06	• Burkitt Lymphoma	• Kluiver J, Haralambieva E, de Jong D, Blokzijl T, Jacobs S, Kroesen BJ, Poppema S, van den Berg A. Lack of BIC and microRNA miR-155 expression in primary cases of Burkitt lymphoma. Genes, Chromosomes and Cancer. 2006 Feb;45(2):147-53.
50.	LINC00504	long intergenic non-protein coding RNA 504	4.698613	0.000779	0.008284	• Non-Small Cell Lung Carcinoma<br>• Colon carcinoma	• Ma HP, Wang LX, Li W, Guo HH, Wu Y, Li XY. Upregulation of LINC00504 is associated with aggressive progression and poor prognosis in non-small cell lung cancer. Eur Rev Med Pharmacol Sci. 2020 Jan 1;24(2):699-703.<br>• Feng J, Ma J, Liu S, Wang J, Chen Y. A noncoding RNA LINC00504 interacts with c-Myc to regulate tumor metabolism in colon cancer. Journal of cellular biochemistry. 2019 Sep;120(9):14725-34.

51.	LY75-CD302	LY75-CD302 readthrough	-989.265	9.65E-08	8.42E-06	• Familial classical Hodgkin lymphoma	• Ristolainen H, Kilpivaara O, Kamper P, Taskinen M, Saarinen S, Leppä S, d'Amore F, Aaltonen LA. Identification of homozygous deletion in ACAN and other candidate variants in familial classical Hodgkin lymphoma by exome sequencing.
52.	PPBP (CTAPIII)	pro-platelet basic protein (chemokine (C-X-C motif) ligand 7)	-272.27	4.10E-17	1.03E-13	• Malignant neoplasm of lung,	• Du Q, Li E, Liu Y, Xie W, Huang C, Song J, Zhang W, Zheng Y, Wang H, Wang Q. CTAPIII/CXCL 7: a novel biomarker for early diagnosis of lung cancer. Cancer medicine. 2018 Feb;7(2):325-35.
53.	CA12	carbonic anhydrase XII	-17.4539	1.03E-08	1.39E-06	• Lung neoplasms	• Xiong D, Pan J, Zhang Q, Szabo E, Miller MS, Lubet RA, You M, Wang Y. Bronchial airway gene expression signatures in mouse lung squamous cell carcinoma and their modulation by cancer chemopreventive agents. Oncotarget. 2017 Mar 21;8(12):18885.
54.	F13A1	coagulation factor XIII; A1 polypeptide	-17.0878	1.12E-06	5.76E-05	• Hereditary Factor XIII Deficiency	• Jia S, He Y, Lu M, Liao N, Lei Y, Lauriane N, Liang K, Wei H. Identification of novel pathogenic F13A1 mutation and novel NBEAL2 gene missense mutation in a pedigree with hereditary congenital factor XIII deficiency. Gene. 2019 Jun 20;702:143-7.
55.	PVALB	parvalbumin	-16.8867	0.000833	0.00866	• Parkinson disease	• Soós J, Engelhardt JI, Siklós L, Havas L, Majtényi K. The expression of PARP, NF-κB and parvalbumin is increased in Parkinson disease. Neuroreport. 2004 Aug 6;15(11):1715-8.
56.	SPATA12	spermatogenesis associated 12	-14.047	6.36E-06	0.000238	• Tumor progression	• Liu Z, Lin Y, Liu X, Yu W, Zhang Y, Li D. Experimental study of inhibition of tumor cell proliferation by a novel gene SPATA12. Zhong nan da xue xue bao. Yi xue ban= Journal of Central South University. Medical sciences. 2012 Mar 1;37(3):222-7.
57.	CCL13	chemokine (C-C motif) ligand 13	-14.0324	1.57E-09	3.29E-07	• Asthma	• Winkler C, Witte L, Moraw N, Faulenbach C, Müller M, Holz O, Schaumann F, Hohlfeld JM. Impact of endobronchial allergen provocation on macrophage phenotype in asthmatics. BMC immunology. 2014 Dec;15(1):1-1.
58.	ALK	anaplastic lymphoma receptor tyrosine kinase	-13.6253	0.00311	0.022872	• Adenocarcinoma of lung	• Qiu T, Li W, Zhang F, Wang B, Ying J. Major challenges in accurate mutation detection of multifocal lung adenocarcinoma by next-generation sequencing. Cancer biology & therapy. 2020 Feb 1;21(2):170-7.

59.	EDNRB	endothelin receptor type B	-12.8236	0.000898	0.0091	• Asthma<br>• Waardenburg-Hirschsprung Disease<br>• Hypertension	• Taillé C, Guénégou A, Almolki A, Piperaud M, Leynaert B, Vuillaumier S, Neukirch F, Boczkowski J, Aubier M, Benessiano J, Crestani B. ET B receptor polymorphism is associated with airway obstruction. BMC pulmonary medicine. 2007 Dec;7(1):1-9.<br>• Edery P, Attie T, Amiel J, Pelet A, Eng C, Hofstra RM, Martelli H, Bidaud C, Munnich A, Lyonnet S. Mutation of the endothelin-3 gene in the Waardenburg-Hirschsprung disease (Shah-Waardenburg syndrome). Nature genetics. 1996 Apr;12(4):442-4.<br>• Li J, Cao YX, Liu H, Xu CB. Enhanced G-protein coupled receptors-mediated contraction and reduced endothelium-dependent relaxation in hypertension. European journal of pharmacology. 2007 Feb 28;557(2-3):186-94.
60.	TMEM37	transmembrane protein 37	-12.0911	1.19E-07	9.70E-06	• Type-2 diabetes<br>• Colon cancer	• Solimena M, Schulte AM, Marselli L, Ehehalt F, Richter D, Kleeberg M, Mziaut H, Knoch KP, Parnis J, Bugliani M, Siddiq A. Systems biology of the IMIDIA biobank from organ donors and pancreatectomised patients defines a novel transcriptomic signature of islets from individuals with type 2 diabetes. Diabetologia. 2018 Mar;61(3):641-57.<br>• Li C, Shen Z, Zhou Y, Yu W. Independent prognostic genes and mechanism investigation for colon cancer. Biological Research. 2018;51.
61.	ASIC1	acid sensing (proton gated) ion channel 1	-11.9264	0.000119	0.0021	• Stomach carcinoma	• Heydari-Mehrabadi A, Tamandani DM, Baranzehi T, Hemati S. Analysis of polymorphism and expression profile of ASIC1 and IL-6 genes in patients with gastric cancer. Asian Pacific journal of cancer prevention: APJCP. 2018;19(12):3451.
62.	NRCAM	neuronal cell adhesion molecule	-10.9853	9.22E-09	1.27E-06	• Malignant neoplasm of breast<br>• Colorectal carcinoma	• Yu JC, Hsiung CN, Hsu HM, Bao BY, Chen ST, Hsu GC, Chou WC, Hu LY, Ding SL, Cheng CW, Wu PE. Genetic variation in the genome-wide predicted estrogen response element-related sequences is associated with breast cancer development. Breast Cancer Research. 2011 Feb;13(1):1-8.<br>• Yi JM, Dhir M, Van Neste L, Downing SR, Jeschke J, Glöckner SC, de Freitas Calmon M, Hooker CM, Funes JM, Boshoff C, Smits KM. Genomic and epigenomic integration identifies a prognostic signature in colon cancer. Clinical Cancer Research. 2011 Mar 15;17(6):1535-45.
63.	ADORA3	adenosine A3 receptor	-10.5494	0.000154	0.002522	• Liver carcinoma<br>• Ulcerative colitis<br>• Inflammatory bowel disease	• Ayoub BM, Attia YM, Ahmed MS. Structural re-positioning, in silico molecular modelling, oxidative degradation, and biological screening of linagliptin as adenosine 3 receptor (ADORA3) modulators targeting hepatocellular carcinoma. Journal of enzyme inhibition and medicinal chemistry. 2018 Jan 1;33(1):858-66.<br>• Ren TH, Lv MM, An XM, Leung WK, Seto WK. Activation of adenosine A3 receptor inhibits inflammatory cytokine production in colonic mucosa of patients with ulcerative colitis by down-regulating the nuclear factor-kappa B signaling. Journal of digestive diseases. 2020 Jan;21(1):38-45.<br>• Ren T, Qiu Y, Wu W, Feng X, Ye S, Wang Z, Tian T, He Y, Yu C, Zhou Y. Activation of adenosine A3 receptor alleviates TNF-α-induced inflammation through inhibition of the NF-κB signaling pathway in human colonic epithelial cells. Mediators of inflammation. 2014 Oct;2014.

64.	RGS18	regulator of G-protein signaling 18	-10.1647	2.49E-06	0.000111	• Stomach carcinoma	• Su C, Li H, Peng Z, Ke D, Fu H, Zheng X. Identification of plasma RGS18 and PPBP mRNAs as potential biomarkers for gastric cancer using transcriptome arrays. Oncology letters. 2019 Jan 1;17(1):247-55.
65.	CXCL5	chemokine (C-X-C motif) ligand 5	-9.99121	1.13E-10	3.54E-08	• Pneumonia	• Traber KE, Hilliard KL, Allen E, Wasserman GA, Yamamoto K, Jones MR, Mizgerd JP, Quinton LJ. Induction of STAT3-dependent CXCL5 expression and neutrophil recruitment by oncostatin-M during pneumonia. American journal of respiratory cell and molecular biology. 2015 Oct;53(4):479-88.
66.	COL5A2	collagen; type V; alpha 2	-9.37249	4.12E-06	0.000165	• Ehlers-Danlos syndrome (EDS)<br>• Colorectal Cancer	• Richards AJ, Martin S, Nicholls AC, Harrison JB, Pope FM, Burrows NP. A single base mutation in COL5A2 causes Ehlers-Danlos syndrome type II. Journal of medical genetics. 1998 Oct 1;35(10):846-8.<br>• Fischer H, Stenling R, Rubio C, Lindblom A. Colorectal carcinogenesis is associated with stromal expression of COL11A1 and COL5A2. Carcinogenesis. 2001 Jun 1;22(6):875-8.
67.	GAPLINC	gastric adenocarcinoma associated; positive CD44 regulator; long intergenic non-coding RNA	-8.77823	2.56E-06	0.000113	• Neoplasm Metastasis	• Zhang W, Yao H, Wu Y. Poor expression of long-chain noncoding RNA GAPLINC inhibits epithelial–mesenchymal transition, and invasion and migration of hepatocellular carcinoma cells. Anti-cancer drugs. 2019 Sep 1;30(8):784-94.
68.	ITGB3	integrin; beta 3 (platelet glycoprotein IIIa; antigen CD61)	-8.33151	8.90E-09	1.26E-06	• Glanzmann thrombasthenia (GT)	• Lu Z, Nikuze L, Zhong Z, Li F, Zhang F, Liang K, Wei M, Wei H. Identification of one novel pathogenic ITGB3 mutation and two known mutations in two Chinese pedigrees with hereditary Glanzmann thrombasthenia. Platelets. 2020 Apr 2;31(3):355-9.
69.	COLEC12	collectin sub-family member 12	-8.10608	1.59E-08	1.96E-06	• Diabetic Retinopathy	• Peng D, Wang J, Zhang R, Jiang F, Tang S, Chen M, Yan J, Sun X, Wang S, Wang T, Yan D. Common variants in or near ZNRF1, COLEC12, SCYL1BP1 and API5 are associated with diabetic retinopathy in Chinese patients with type 2 diabetes. Diabetologia. 2015 Jun;58(6):1231-8.
70.	MS4A6A	membrane-spanning 4-domains; subfamily A; member 6A	-7.95797	9.85E-11	3.35E-08	• Alzheimer's Disease	• Cáceres A, Vargas JE, González JR. APOE and MS4A6A interact with GnRH signaling in Alzheimer's disease: Enrichment of epistatic effects. Alzheimer's & Dementia. 2017 Apr 1;13(4):493-7.
71.	JPH4	junctophilin 4	-7.92674	1.25E-06	6.27E-05	• Endometrial carcinoma	• Chung TK, Cheung TH, Huen NY, Wong KW, Lo KW, Yim SF, Siu NS, Wong YM, Tsang PT, Pang MW, Yu MY. Dysregulated microRNAs and their predicted targets associated with endometrioid endometrial adenocarcinoma in Hong Kong women. International journal of cancer. 2009 Mar 15;124(6):1358-65.
72.	FAXDC2	fatty acid hydroxylase domain containing 2	-7.68315	1.27E-08	1.63E-06	• Parkinson's Disease	• Santiago JA, Bottero V, Potashkin JA. Evaluation of RNA blood biomarkers in the Parkinson's disease biomarkers program. Frontiers in aging neuroscience. 2018 May 29;10:157.
73.	PDK4	pyruvate dehydrogenase kinase; isozyme 4	-7.61853	1.82E-06	8.47E-05	• Liver carcinoma,<br>• Diabetes Mellitus	• Song K, Kwon H, Han C, Zhang J, Dash S, Lim K, Wu T. Active glycolytic metabolism in CD133 (+) hepatocellular cancer stem cells: regulation by MIR-122. Oncotarget. 2015 Dec 1;6(38):40822.<br>• Putra SE, Singajaya S, Thesman F, Pranoto DA, Sanjaya R, Vianney YM, Artadana IB. Aberrant PDK4 promoter methylation preceding hyperglycemia in a mouse model. Applied biochemistry and biotechnology. 2020 Mar;190(3):1023-34.
74.	MMP8	matrix metallopeptidase 8	-7.61242	3.69E-05	0.000905	• Melanoma	• Vihinen P, Koskivuo I, Syrjänen K, Tervahartiala T, Sorsa T, Pyrhönen S. Serum matrix metalloproteinase-8 is associated with ulceration and vascular invasion of malignant melanoma. Melanoma research. 2008 Aug 1;18(4):268-73.

						• Inflammatory Bowel Disease	• Schmidt J, Weigert M, Leuschner C, Hartmann H, Raddatz D, Haak R, Mausberg RF, Kottmann T, Schmalz G, Ziebolz D. Active matrix metalloproteinase-8 and periodontal bacteria—interlink between periodontitis and inflammatory bowel disease?. Journal of periodontology. 2018 Jun;89(6):699-707.
75.	PPFIA4	protein tyrosine phosphatase; receptor type; f polypeptide (PTPRF); interacting protein (liprin); alpha 4	-7.06275	4.40E-05	0.001033	• Colon cancer	• Huang J, Yang M, Liu Z, Li X, Wang J, Fu N, Cao T, Yang X. PPFIA4 Promotes Colon Cancer Cell Proliferation and Migration by Enhancing Tumor Glycolysis. Frontiers in oncology. 2021 May 20;11:1776.
76.	FPR1	formyl peptide receptor 1	-6.98912	2.57E-05	0.000699	• Aggressive Periodontitis<br>• Cervical Cancer	• Gunji T, Onouchi Y, Nagasawa T, Katagiri S, Watanabe H, Kobayashi H, Arakawa S, Noguchi K, Hata A, Izumi Y, Ishikawa I. Functional polymorphisms of the FPR1 gene and aggressive periodontitis in Japanese. Biochemical and biophysical research communications. 2007 Dec 7;364(1):7-13.<br>• Cao G, Zhang Z. FPR1 mediates the tumorigenicity of human cervical cancer cells. Cancer management and research. 2018;10:5855.
77.	IQCD	IQ motif containing D	-6.91666	0.000291	0.00403	• Familial ventricular fibrillation	• Zhang P, Jiang W, Luo N, Zhu W, Fan L. Corrigendum to: IQ motif containing D (IQCD), a new acrosomal protein involved in the acrosome reaction and fertilisation. Reproduction, Fertility and Development. 2019;31(5):1033-..
78.	VNN1	vanin 1	-6.91545	0.000445	0.005445	• Colorectal cancer<br>• Rectal cancer	• Løvf M, Nome T, Bruun J, Eknæs M, Bakken AC, Mpindi JP, Kilpinen S, Rognum TO, Nesbakken A, Kallioniemi O, Lothe RA. A novel transcript, VNN1-AB, as a biomarker for colorectal cancer. International journal of cancer. 2014 Nov 1;135(9):2077-84.<br>• Chai CY, Zhang Y, Song J, Lin SC, Sun S, Chang IW. VNN1 overexpression is associated with poor response to preoperative chemoradiotherapy and adverse prognosis in patients with rectal cancers. American journal of translational research. 2016;8(10):4455.
79.	TRPV4	transient receptor potential cation channel; subfamily V; member 4	-6.69711	7.07E-09	1.07E-06	• fibrosis<br>• Pulmonary hypertension, acute lung injury, oedema	• Zhan L, Li J. The role of TRPV4 in fibrosis. Gene. 2018 Feb 5;642:1-8.<br>• Grace MS, Bonvini SJ, Belvisi MG, McIntyre P. Modulation of the TRPV4 ion channel as a therapeutic target for disease. Pharmacology & therapeutics. 2017 Sep 1;177:9-22.
80.	C10orf128	chromosome 10 open reading frame 128	-6.64313	6.48E-08	6.21E-06	• Aero-digestive squamous cell carcinoma	• Lesseur C, Ferreiro-Iglesias A, McKay JD, Bossé Y, Johansson M, Gaborieau V, Landi MT, Christiani DC, Caporaso NC, Bojesen SE, Amos CI. Genome-wide association meta-analysis identifies pleiotropic risk loci for aerodigestive squamous cell cancers. PLoS genetics. 2021 Mar 5;17(3):e1009254.
81.	MRC1	mannose receptor; C type 1	-6.44862	5.52E-14	6.43E-11	• Leprosy<br>• Asthma	• Wang D, Feng JQ, Li YY, Zhang DF, Li XA, Li QW, Yao YG. Genetic variants of the MRC1 gene and the IFNG gene are associated with leprosy in Han Chinese from Southwest China. Human genetics. 2012 Jul;131(7):1251-60.<br>• Hattori T, Konno S, Hizawa N, Isada A, Takahashi A, Shimizu K, Shimizu K, Gao P, Beaty TH, Barnes KC, Huang SK. Genetic variants in the mannose receptor gene (MRC1) are associated with asthma in two independent populations. Immunogenetics. 2009 Dec;61(11):731-8.
82.	TLE3	transducin-like enhancer of split 3	-6.26732	3.10E-09	5.56E-07	• Colorectal cancer	• Liu L, Zhang Y, Wong CC, Zhang J, Dong Y, Li X, Kang W, Chan FK, Sung JJ, Yu J. RNF6 promotes colorectal cancer by activating the

						• Prostate cancer	Wnt/β-catenin pathway via ubiquitination of TLE3. Cancer research. 2018 Apr 15;78(8):1958-71.<br>• Palit SA, Vis D, Stelloo S, Lieftink C, Prekovic S, Bekers E, Hofland I, Šuštić T, Wolters L, Beijersbergen R, Bergman AM. TLE3 loss confers AR inhibitor resistance by facilitating GR-mediated human prostate cancer cell growth. Elife. 2019 Dec 19;8:e47430.
83.	IGFBP2	insulin-like growth factor binding protein 2; 36kDa	-6.1432	4.87E-06	0.000193	• Bipolar Disorder<br>• Glioblastom<br>• Astrocytoma	• Bezchlibnyk YB, Xu L, Wang JF, Young LT. Decreased expression of insulin-like growth factor binding protein 2 in the prefrontal cortex of subjects with bipolar disorder and its regulation by lithium treatment. Brain research. 2007 May 25;1147:213-7<br>• Wang H, Wang H, Shen W, Huang H, Hu L, Ramdas L, Zhou YH, Liao WS, Fuller GN, Zhang W. Insulin-like growth factor binding protein 2 enhances glioblastoma invasion by activating invasion-enhancing genes. Cancer research. 2003 Aug 1;63(15):4315-21<br>• Becher OJ, Peterson KM, Khatua S, Santi MR, MacDonald TJ. IGFBP2 is overexpressed by pediatric malignant astrocytomas and induces the repair enzyme DNA-PK. Journal of child neurology. 2008 Oct;23(10):1205-13.
84.	LOC100996455	NA	-6.10151	4.80E-05	0.001095	○ NA	▪ NA
85.	ACE	angiotensin I converting enzyme	-6.04821	8.66E-16	1.55E-12	• Alzheimer's disease	• Quitterer U, AbdAlla S. Improvements of symptoms of Alzheimers disease by inhibition of the angiotensin system. Pharmacological research. 2020 Apr 1;154:104230.
86.	SGCG	sarcoglycan; gamma (35kDa dystrophin-associated glycoprotein)	-5.9913	0.001088	0.010525	• Muscular Dystrophies	• Spinazzola JM, Smith TC, Liu M, Luna EJ, Barton ER. Gamma-sarcoglycan is required for the response of archvillin to mechanical stimulation in skeletal muscle. Human molecular genetics. 2015 May 1;24(9):2470-81.
87.	MARCO	macrophage receptor with collagenous structure	-5.95806	3.73E-13	2.93E-10	• Hepatocellular carcinoma,	• Xiao Y, Chen B, Yang K, Wang Q, Liu P, Gu Y, Zhong Q, Liu Z, He Y, Liu Q. Down-regulation of MARCO associates with tumor progression in hepatocellular carcinoma. Experimental cell research. 2019 Oct 15;383(2):111542.
88.	GGTA1P	glycoprotein; alpha-galactosyltransferase 1 pseudogene	-5.90238	1.46E-06	7.06E-05	• Lung Cancer<br>• Breast Cancer	• Zhou W, Liu T, Saren G, Liao L, Fang W, Zhao H. Comprehensive analysis of differentially expressed long non-coding RNAs in non-small cell lung cancer. Oncology letters. 2019 Aug 1;18(2):1145-56.<br>• Tian T, Gong Z, Wang M, Hao R, Lin S, Liu K, Guan F, Xu P, Deng Y, Song D, Li N. Identification of long non-coding RNA signatures in triple-negative breast cancer. Cancer cell international. 2018 Dec;18(1):1-0.
89.	LOC101929532	NA	-5.8984	8.49E-06	0.000293	• NA	○ NA
90.	CEACAM3	carcinoembryonic antigen-related cell adhesion molecule 3	-5.67511	0.00087	0.008903	• Colorectal carcinoma	• Kim BC, Bae JH, Park SM, Won DY, Lee IK. Is ascites CEA a risk factor for peritoneal carcinomatosis in colorectal cancer?: a long-term follow-up study. International journal of colorectal disease. 2020 Jan;35(1):147-55.
91.	CIDEB	cell death-inducing DFFA-like effector b	-5.657	2.12E-08	2.52E-06	• Hepatitis B<br>• Fatty liver disease	• Yasumoto J, Kasai H, Yoshimura K, Otoguro T, Watashi K, Wakita T, Yamashita A, Tanaka T, Takeda S, Moriishi K. Hepatitis B virus prevents excessive viral production via reduction of cell death-inducing DFF45-like effectors. Journal of General Virology. 2017 Jul 1;98(7):1762-73.<br>• Li JZ, Ye J, Xue B, Qi J, Zhang J, Zhou Z, Li Q, Wen Z, Li P. Cideb regulates diet-induced obesity, liver steatosis, and insulin

							sensitivity by controlling lipogenesis and fatty acid oxidation. Diabetes. 2007 Oct 1;56(10):2523-32
92.	CLEC1A	C-type lectin domain family 1; member A	-5.56198	1.10E-08	1.45E-06	• NA	▪ NA
93.	SERPINE1	serpin peptidase inhibitor; clade E (nexin; plasminogen activator inhibitor type 1); member 1	-5.53344	0.000279	0.003917	• Obesity-Induced Diabetes<br>• Thrombosis And Atherosclerosis	• Coudriet GM, Stoops J, Orr AV, Bhushan B, Koral K, Lee S, Previte DM, Dong HH, Michalopoulos GK, Mars WM, Piganelli JD. A noncanonical role for plasminogen activator inhibitor type 1 in obesity-induced diabetes. The American journal of pathology. 2019 Jul 1;189(7):1413-22.<br>• Zhu C, Shen H, Zhu L, Zhao F, Shu Y. Plasminogen activator inhibitor 1 promotes immunosuppression in human non-small cell lung cancers by enhancing TGF-B1 expression in macrophage. Cellular Physiology and Biochemistry. 2017;44(6):2201-11.
94.	MUCL1	mucin-like 1	-5.53318	0.001189	0.011132	• Breast Carcinoma	• Conley SJ, Bosco E, Tice D, Hollingsworth R, Herbst R, Xiao Z. Characterization of mucin-like 1 (MUCL1) in breast cancer and its novel role as a potent activator of cell proliferation.
95.	E2F2	E2F transcription factor 2	-5.42335	0.002588	0.020163	• Non-Small Cell Lung Carcinoma	• Sun CC, Zhou Q, Hu W, Li SJ, Zhang F, Chen ZL, Li G, Bi ZY, Bi YY, Gong FY, Bo T. Transcriptional E2F1/2/5/8 as potential targets and transcriptional E2F3/6/7 as new biomarkers for the prognosis of human lung carcinoma. Aging (Albany NY). 2018 May;10(5):973.
96.	FCGR3B	Fc fragment of IgG; low affinity IIIb; receptor (CD16b)	-5.3552	0.003056	0.022566	• Lupus Erythematosus Systemic	• Guo X, Fang X, He G, Zaman MH, Fei X, Qiao W, Deng GM. The role of neutrophils in skin damage induced by tissue-deposited lupus IgG. Immunology. 2018 Aug;154(4):604-12.
97.	CACNA2D3	calcium channel; voltage-dependent; alpha 2/delta subunit 3	-5.32181	0.039144	0.138414	• Esophageal Squamous Cell Carcinoma	• Nie C, Qin X, Li X, Tian B, Zhao Y, Jin Y, Li Y, Wang Q, Zeng D, Hong A, Chen X. CACNA2D3 enhances the chemosensitivity of esophageal squamous cell carcinoma to cisplatin via inducing Ca2+-mediated apoptosis and suppressing PI3K/Akt pathways. Frontiers in oncology. 2019 Apr 2;9:185.
98.	KCNJ15	potassium channel; inwardly rectifying subfamily J; member 15	-5.27447	0.000381	0.004888	• Type 2 Diabetes	• Okamoto K, Iwasaki N, Doi K, Noiri E, Iwamoto Y, Uchigata Y, Fujita T, Tokunaga K. Inhibition of glucose-stimulated insulin secretion by KCNJ15, a newly identified susceptibility gene for type 2 diabetes. Diabetes. 2012 Jul 1;61(7):1734-41.
99.	NDP	Norrie disease (pseudoglioma)	-5.27422	4.90E-06	0.000193	• Norrie Disease<br>• Familial Exudative Vitreoretinopath	• Torrente I, Mangino M, Gennarelli M, Novelli G, Giannotti A, Vadalà P, Dallapiccola B. Two new missense mutations (A105T and C110G) in the norrin gene in two Italian families with Norrie disease and familial exudative vitreoretinopathy. American journal of medical genetics. 1997 Oct 17;72(2):242-4.<br>• Nikopoulos K, Gilissen C, Hoischen A, van Nouhuys CE, Boonstra FN, Blokland EA, Arts P, Wieskamp N, Strom TM, Ayuso C, Tilanus MA. Next-generation sequencing of a 40 Mb linkage interval reveals TSPAN12 mutations in patients with familial exudative vitreoretinopathy. The American Journal of Human Genetics. 2010 Feb 12;86(2):240-7.
100.	EVI2A	ecotropic viral integration site 2A	-5.1707	1.44E-06	7.02E-05	• Endometrial Carcinoma	• Cawthon RM, O'Connell P, Buchberg AM, Viskochil D, Weiss RB, Culver M, Stevens J, Jenkins NA, Copeland NG, White R. Identification and characterization of transcripts from the neurofibromatosis 1 region: the sequence and genomic structure of EVI2 and mapping of other transcripts. Genomics. 1990 Aug 1;7(4):555-65.
