# Supplementary material for: Distinct molecular phenotypes involving several human diseases are induced by IFN-λ3 and IFN-λ4 in monocyte-derived macrophages
Source: Genes Immun. 2022 Feb 3;23(2):73–84. doi: 10.1038/s41435-022-00164-w (PMC9042695; doi:10.1038/s41435-022-00164-w)
Supplement: Supplementary file 3 — Supplemental Data 1 [file 41435_2022_164_MOESM3_ESM.pdf]

Supplementary Data 1

M1-MDM KEGG enrichment (IFN-L3 vs NT)

PATH_ID	Description	GeneRatio	BgRatio	pvalue	p.adjust	qvalue	geneID	Count
hsa04978	Mineral absorption	14/527	59/8101	1.78E-05	0.005585	0.00509	ATP1B1/ATP1B2/ATP2B1/FXYD2/HMOX2/MT1A /MT1E/MT1F/MT1G/MT1H/MT1M/MT1X/ MT2A/SLC8A3	14
hsa04640	Hematopoietic cell lineage	18/527	99/8101	5.82E-05	0.009101	0.008294	CD14/CD1A/CD1B/CD1C/CD1E/CD33/CD36/CD38 /CD55/CR1/CSF1/GP1BA/HLA-DMB/HLA-DOB/ IL1R2/IL2RA/IL6/ITGA3	18
hsa05146	Amoebiasis	18/527	102/8101	8.73E-05	0.009107	0.0083	ARG2/CD14/CD1A/CD1B/CD1C/CD1E/CXCL2 /CXCL3/GNA14/GNA15/IL12B/IL1R2/IL6/ LAMB1/PLCB2/RAB7B/SERPINB9/TLR2	18
hsa04061	Viral protein interaction with cytokine and cytokine receptor	16/527	100/8101	0.000669	0.041728	0.03803	ACKR3/CCL1/CCL20/CCL22/CCL24/CCL4/CCL4L2 /CCL8/CSF1/CXCL13/CXCL2/CXCL3/CXCL9/ IL2RA/IL6/TNFRSF10C	16
hsa05323	Rheumatoid arthritis	15/527	93/8101	0.000896	0.041728	0.03803	ACP5/ATP6V0E2/CCL20/CSF1/CTSK/CTSL/CXCL2 /CXCL3/HLA-DMB/HLA-DOB/IL6/MMP1/ TLR2/TNFRSF11A/VEGFA	15
hsa05134	Legionellosis	11/527	57/8101	0.000947	0.041728	0.03803	BNIP3/CD14/CR1/CXCL2/CXCL3/CYCS/ HSPA8/IL12B/IL6/NFKBIA/TLR2	11
hsa01230	Biosynthesis of amino acids	13/527	75/8101	0.000978	0.041728	0.03803	ALDOC/ARG2/BCAT1/BCAT2/CBS/ENO2/GAPDH /GOT2/GPT2/IDH3A/PGK1/PSPH/SDS	13

hsa04610	Complement and coagulation cascades	14/527	85/8101	0.001067	0.041728	0.03803	C3AR1/C5/CD55/CFB/CFD/CFH/CR1/PLAT/PLAU/PLAUR/PROS1/SERPINB2/THBD/VSIG4	14
hsa05150	Staphylococcus aureus infection	15/527	96/8101	0.001253	0.042929	0.039125	C3AR1/C5/CFB/CFD/CFH/FCAR/FCGR2A/FCGR3A/FCGR3B/FPR1/FPR2/HLA-DMB/HLA-DOB/KRT23/SELPLG	15
hsa04060	Cytokine-cytokine receptor interaction	33/527	295/8101	0.001487	0.042929	0.039125	ACKR3/ACVR2B/CCL1/CCL20/CCL22/CCL24/CCL4/CCL4L2/CCL8/CSF1/CXCL13/CXCL2/CXCL3/CXCL9/IL12B/IL1R2/IL1RN/IL21R/IL27RA/IL2RA/IL31RA/IL6/LEPR/LIFR/OSM/OSMR/PRLR/TNFRSF10C/TNFRSF11A/TNFRSF18/TNFRSF21/TNFRSF4/TNFRSF8	33
hsa04512	ECM-receptor interaction	14/527	88/8101	0.001509	0.042929	0.039125	CD36/COL6A1/COL6A3/COL9A2/DAG1/GP1BA/HMMR/IBSP/ITGA11/ITGA3/ITGAV/ITGB7/ITGB8/LAMB1	14
hsa05202	Transcriptional misregulation in cancer	23/527	192/8101	0.00319	0.083141	0.075773	ARNT2/BCL2L1/BIRC3/CCND2/CD14/CDKN1B/CDKN2C/CEBPA/FUT8/GADD45A/HPGD/IGFBP3/IL1R2/IL6/ITGB7/LMO2/MAF/MEF2C/MLLT3/PER2/PLAT/PLAU/RUNX2	23
hsa00534	Glycosaminoglycan biosynthesis - heparan sulfate / heparin	6/527	24/8101	0.003615	0.083141	0.075773	HS3ST1/HS3ST3A1/HS3ST3B1/HS6ST1/NDST2/XYLT1	6
hsa04380	Osteoclast differentiation	17/527	128/8101	0.003719	0.083141	0.075773	ACP5/CSF1/CTSK/FCGR2A/FCGR3A/FCGR3B/FOSL2/FYN/GAB2/LILRA2/LILRA5/MAP3K14/NCF2/NFKBIA/PPP3CA/SOCS3/TNFRSF11A	17
hsa00270	Cysteine and methionine metabolism	9/527	50/8101	0.004433	0.092505	0.084307	APIP/BCAT1/BCAT2/CBS/GOT2/LDHA/SDS/SRM/TST	9

hsa00330	Arginine and proline metabolism	9/527	51/8101	0.005081	0.0994	0.090591	ALDH1B1/ARG2/AZIN2/GOT2/MAOA/P4HA1/SAT2 /SMOX/SRM	9
hsa01210	2-Oxocarboxylic acid metabolism	5/527	19/8101	0.006193	0.11403	0.103926	BCAT1/BCAT2/GOT2/GPT2/IDH3A	5
hsa04974	Protein digestion and absorption	14/527	103/8101	0.006598	0.11474	0.104572	ATP1B1/ATP1B2/COL23A1/COL6A1/COL6A3 /COL7A1/COL8A2/COL9A2/DPP4/FXYD2 /MEP1A/SLC16A10/SLC1A5/SLC8A3	14
hsa05166	Human T-cell leukemia virus 1 infection	24/527	219/8101	0.008088	0.129638	0.11815	ADCY6/ANAPC1/BCL2L1/CCND1/CCND2/CDKN2B /CDKN2C/CHEK2/EGR1/EGR2/ETS1/HLA-DMB/HLA-DOB /IL1R2/IL2RA/IL6/JAK3/KAT2A/MAP3K14/NFKBIA/ PPP3CA/SLC2A1/SMAD3/ZFP36	24
hsa05321	Inflammatory bowel disease	10/527	65/8101	0.008729	0.129638	0.11815	HLA-DMB/HLA-DOB/IL12B/IL21R/IL6 /MAF/NOD2/RORA/SMAD3/TLR2	10
hsa04145	Phagosome	18/527	152/8101	0.009601	0.129638	0.11815	ATP6V0E2/CD14/CD36/CTSL/FCAR/FCGR2A /FCGR3A/FCGR3B/HLA-DMB/HLA- DOB/ ITGAV/MARCO/NCF2/RAB7B/TLR2/ TUBA4A/TUBB3/TUBB4A	18
hsa00770	Pantothenate and CoA biosynthesis	5/527	21/8101	0.009731	0.129638	0.11815	ALDH1B1/BCAT1/BCAT2/VNN2/VNN3	5
hsa04666	Fc gamma R-mediated phagocytosis	13/527	97/8101	0.009752	0.129638	0.11815	FCGR2A/FCGR3A/FCGR3B/GAB2/GSN/LIM K1/ LIMK2/MARCKS/MYO10/PLA2G4B/PLD1/ VAV3/WASF1	13
hsa05140	Leishmaniasis	11/527	77/8101	0.010601	0.129638	0.11815	CR1/FCGR2A/FCGR3A/FCGR3B/HLA-DMB/HLA-DOB /IL12B/NCF2/NFKBIA/PTGS2/TLR2	11

hsa05412	Arrhythmogenic right ventricular cardiomyopathy	11/527	77/8101	0.010601	0.129638	0.11815	CACNA2D4/CACNB3/DAG1/DSG2/ITGA11 /ITGA3/ITGAV/ITGB7/ITGB8/SLC8A3/TCF7L1	11
hsa04066	HIF-1 signaling pathway	14/527	109/8101	0.010769	0.129638	0.11815	ALDOC/CDKN1B/EGLN1/ENO2/GAPDH/HIF1A/IL6/ LDHA/PDK1/PFKFB3/PGK1/SLC2A1/TIMP1/VEGFA	14
hsa03020	RNA polymerase	6/527	31/8101	0.013435	0.15113	0.137738	POLR1A/POLR1B/POLR1C/POLR1E/POLR3B/POLR3D	6
hsa00260	Glycine; serine and threonine metabolism	7/527	40/8101	0.01352	0.15113	0.137738	CBS/CHDH/GCSH/MAOA/PIPOX/PSPH/SDS	7
hsa05222	Small cell lung cancer	12/527	92/8101	0.015622	0.16861	0.153668	BCL2L1/BIRC3/CCND1/CDKN1B/CDKN2B/CYCS /GADD45A/ITGA3/ITGAV/LAMB1/NFKBIA/PTGS2	12
hsa04514	Cell adhesion molecules	17/527	149/8101	0.016437	0.171098	0.155936	ALCAM/CD226/CLDN1/HLA-DMB/HLA-DOB/ICOSLG /ITGAV/ITGB7/ITGB8/NLGN2/NTNG2/PDCD1LG2/ PTPRM/SELL/SELPLG/SPN/VCAN	17
hsa04064	NF-kappa B signaling pathway	13/527	104/8101	0.016946	0.171098	0.155936	BCL2L1/BIRC3/CCL4/CCL4L2/CD14/CXCL2/CXCL3 /GADD45A/MAP3K14/NFKBIA/PLAU/PTGS2/TNFRSF11A	13
hsa04115	p53 signaling pathway	10/527	73/8101	0.01908	0.186625	0.170087	BCL2L1/CCND1/CCND2/CCNG2/CDK1/CHEK2/CYCS /GADD45A/IGFBP3/PMAIP1	10
hsa04961	Endocrine and otherfactor-regulated calcium reabsorption	8/527	53/8101	0.020183	0.191432	0.174468	ADCY6/ATP1B1/ATP1B2/ATP2B1/FXYD2/KL/PLCB2/SLC8A 3	8

hsa04921	Oxytocin signaling pathway	17/527	154/8101	0.022071	0.203187	0.185181	ADCY6/CACNA2D4/CACNB3/CCND1/CD38/EEF2K/GUCY1A3/KCNJ5/MEF2C/MYLK/NPR1/PIK3CG/PLA2G4B/PLCB2/PPP3CA/PTGS2/RGS2	17
hsa04668	TNF signaling pathway	13/527	112/8101	0.02944	0.263281	0.23995	BIRC3/CCL20/CSF1/CXCL2/CXCL3/IL6/MAP3K14/MAP3K8/NFKBIA/NOD2/PGAM5/PTGS2/SOCS3	13
hsa04625	C-type lectin receptor signaling pathway	12/527	104/8101	0.036986	0.321576	0.293079	CARD9/CCL22/CLEC4D/EGR2/IL12B/IL6/LSP1/MAP3K14/NFKBIA/NLRP3/PPP3CA/PTGS2	12
hsa05416	Viral myocarditis	8/527	60/8101	0.039312	0.33256	0.30309	CCND1/CD55/CXADR/CYCS/DAG1/FYN/HLA-DMB/HLA-DOB	8
hsa00230	Purine metabolism	14/527	130/8101	0.042608	0.343488	0.31305	ADCY6/ADSSL1/ATIC/GUCY1A3/NME1/NPR1/NT5E/PAPSS2/PDE4D/PDE6G/PDE8A/PFAS/PGM1/PGM2	14
hsa04062	Chemokine signaling pathway	19/527	192/8101	0.0437	0.343488	0.31305	ADCY6/CCL1/CCL20/CCL22/CCL24/CCL4/CCL4L2/CCL8/CXCL13/CXCL2/CXCL3/CXCL9/GNG12/JAK3/NFKBIA/PIK3CG/PLCB2/PXN/VAV3	19
hsa04659	Th17 cell differentiation	12/527	107/8101	0.044574	0.343488	0.31305	HIF1A/HLA-DMB/HLA-DOB/IL21R/IL27RA/IL2RA/IL6/JAK3/NFKBIA/PPP3CA/RORA/SMAD3	12
hsa04068	FoxO signaling pathway	14/527	131/8101	0.044994	0.343488	0.31305	BNIP3/CCND1/CCND2/CCNG2/CDKN1B/CDKN2B/CDKN2D/GADD45A/HOMER1/HOMER2/IL6/IRS2/SMAD3/SOD2	14
hsa04710	Circadian rhythm	5/527	31/8101	0.047718	0.35561	0.324097	NPAS2/NR1D1/PER1/PER2/RORA	5

M1-MDM KEGG enrichment (IFN-L4 vs NT)

PATH_ID	Description	GeneRatio	BgRatio	pvalue	p.adjust	qvalue	geneID	Count
hsa04060	Cytokine-cytokine receptor interaction	28/195	295/8101	3.51E-10	9.48E-08	9.05E-08	ACKR3/ACVR2B/CCL17/CCL19/CCL24/CCR4/CD70/CSF2/CXCL1/CXCL10/CXCL11/CXCL3/CXCL6/CXCL9/EBI3/IFNLR1/IL1R2/IL36G/LEPR/NGFR/PRLR/TGFBR2/TNFRSF10C/TNFRSF11A/TNFRSF12A/TNFRSF21/TNFSF18/TNFSF9	28
hsa04061	Viral protein interaction with cytokine and cytokine receptor	12/195	100/8101	4.44E-06	0.0005991	0.0005723	ACKR3/CCL17/CCL19/CCL24/CCR4/CXCL1/CXCL10/CXCL11/CXCL3/CXCL6/CXCL9/TNFRSF10C	12
hsa04640	Hematopoietic cell lineage	10/195	99/8101	0.0001256	0.0113075	0.0108006	CD14/CD1B/CD36/CSF2/FLT3/HLA-DOB/IL1R2/ITGA6/KITLG/MME	10
hsa04657	IL-17 signaling pathway	9/195	94/8101	0.0004104	0.0277043	0.0264622	CCL17/CSF2/CXCL1/CXCL10/CXCL3/CXCL6/S100A8/S100A9/TRAF5	9
hsa04062	Chemokine signaling pathway	13/195	192/8101	0.0007164	0.0332658	0.0317744	BCAR1/CCL17/CCL19/CCL24/CCR4/CXCL1/CXCL10/CXCL11/CXCL3/CXCL6/CXCL9/GNGT2/GRK5	13
hsa05146	Amoebiasis	9/195	102/8101	0.0007493	0.0332658	0.0317744	ARG2/CD14/CD1B/CSF2/CXCL1/CXCL3/GNA14/IL1R2/LAMA3	9
hsa04064	NF-kappa B signaling pathway	9/195	104/8101	0.0008624	0.0332658	0.0317744	BIRC3/CCL19/CD14/CXCL1/CXCL3/EDARADD/GADD45B/TNFRSF11A/TRAF5	9

hsa05323	Rheumatoid arthritis	7/195	93/8101	0.0069583	0.234844	0.2243149	ATP6V0D2/CSF2/CXCL1/CXCL3/CXCL6 /HLA-DOB/TNFRSF11A	7
hsa01230	Biosynthesis of amino acids	6/195	75/8101	0.0091573	0.2747176	0.2624008	ACO1/ALDOC/ARG2/BCAT1/ENO2/SDS	6
hsa05134	Legionellosis	5/195	57/8101	0.011676	0.3035712	0.2899608	BNIP3/CD14/CXCL1/CXCL3/NLRC4	5
hsa04360	Axon guidance	10/195	182/8101	0.0123677	0.3035712	0.2899608	ABLIM3/EPHA7/MET/NRP1/PDK1/PPP3CA /SEMA3A/SEMA7A/SLIT1/WNT5A	10
hsa04610	Complement and coagulation cascades	6/195	85/8101	0.0163447	0.3512239	0.335477	C4B/C5AR1/CLU/SERPINB2/TFPI/VTN	6
hsa00380	Tryptophan metabolism	4/195	42/8101	0.0178553	0.3512239	0.335477	ALDH1B1/AOX1/CYP1B1/EHHADH	4
hsa04668	TNF signaling pathway	7/195	112/8101	0.0182116	0.3512239	0.335477	BIRC3/CSF2/CXCL1/CXCL10/CXCL3/CXCL6/TRAF5	7
hsa05222	Small cell lung cancer	6/195	92/8101	0.0232411	0.4183404	0.3995844	BIRC3/GADD45B/ITGA6/ITGAV/LAMA3/TRAF5	6
hsa05230	Central carbon metabolism in cancer	5/195	70/8101	0.0263215	0.4279672	0.4087796	FLT3/MET/PDGFRB/PDK1/RET	5
hsa00280	Valine; leucine and isoleucine degradation	4/195	48/8101	0.0277874	0.4279672	0.4087796	ALDH1B1/AOX1/BCAT1/EHHADH	4

hsa04020	Calcium signaling pathway	11/195	240/8101	0.0301323	0.4279672	0.4087796	ADRB2/ATP2A3/CACNA1I/GNA14/MCOLN1/MET/MST1/P2RX1/PDGFRB/PPP3CA/RET	11
hsa00564	Glycerophospholipid metabolism	6/195	98/8101	0.0305238	0.4279672	0.4087796	CDS1/JMJD7-PLA2G4B/MBOAT2/PLA1A/PLB1/PNPLA6	6
hsa04979	Cholesterol metabolism	4/195	50/8101	0.0317013	0.4279672	0.4087796	CD36/CYP27A1/LRP1/OSBPL5	4
hsa03320	PPAR signaling pathway	5/195	76/8101	0.0358495	0.4544757	0.4340996	CD36/CYP27A1/EHHADH/FABP4/PLIN2	5
hsa01523	Antifolate resistance	3/195	31/8101	0.0376635	0.4544757	0.4340996	ABCC4/ABCG2/FOLR2	3
hsa04215	Apoptosis - multiple species	3/195	32/8101	0.0408452	0.4544757	0.4340996	BIRC3/NGFR/PMAIP1	3
hsa04630	JAK-STAT signaling pathway	8/195	162/8101	0.0416228	0.4544757	0.4340996	AOX1/CSF2/FHL1/IFNLR1/LEPR/PDGFRB/PIM1/PRLR	8
hsa05202	Transcriptional misregulation in cancer	9/195	192/8101	0.0420811	0.4544757	0.4340996	BIRC3/CD14/CSF2/FLT3/GADD45B/IL1R2/MET/NGFR/TGFBR2	9

M1-MDM KEGG enrichment (IFN-L4 vs IFNL-L3)

PATH_ID	Description	GeneRatio	BgRatio	pvalue	p.adjust	qvalue	geneID	Count
hsa04061	Viral protein interaction with cytokine and cytokine receptor	28/554	100/8101	5.80E-11	1.85E-08	1.59E-08	ACKR3/CCL1/CCL13/CCL17/CCL19/CCL22/CCL24/CCL3 /CCL4/CCL4L2/CCL5/CCL8/CCR4/CCR5/CCR6/CCR7/CSF1/CXC L10/CXCL11/CXCL12/CXCL13/CXCL2/ CXCL9/IL10/IL2RA/IL6/IL6R/TNFSF10	28
hsa04062	Chemokine signaling pathway	38/554	192/8101	1.64E-09	2.62E-07	2.25E-07	BCAR1/CCL1/CCL13/CCL17/CCL19/CCL22/CCL24 /CCL3/CCL4/CCL4L2/CCL5/CCL8/CCR4/CCR5 /CCR6/CCR7/CXCL10/CXCL11/CXCL12/CXCL13 /CXCL2/CXCL9/FGR/GNG12/GNG2/GRK5/ GRK6/JAK3/NFKBIA/PIK3CG/PIK3R6/PLCB2/PLCB4/PTK2B/PX N/RAC2/RELA/VAV3	38
hsa04060	Cytokine-cytokine receptor interaction	46/554	295/8101	8.50E-08	9.04E-06	7.75E-06	ACKR3/BMP2/CCL1/CCL13/CCL17/CCL19/CCL22 /CCL24/CCL3/CCL4/CCL4L2/CCL5/CCL8/CCR4/ CCR5/CCR6/CCR7/CSF1/CXCL10/CXCL11/CXCL12/ CXCL13/CXCL2/CXCL9/FAS/IFNLR1/IL10/IL12B/ IL15RA/IL1RN/IL21R/IL27RA/IL2RA/IL31RA/IL6 /IL6R/LIFR/OSM/OSMR/PRLR/TNFRSF18/TNFRSF21/TNFRSF4 /TNFRSF8/TNFSF10/TNFSF13	46
hsa04978	Mineral absorption	14/554	59/8101	3.13E-05	0.002493	0.002139	ATP1B1/ATP1B2/ATP2B4/FXYD2/HMOX2/MT1A /MT1E/MT1F/MT1G/MT1H/MT1M/MT1X/MT2A/SLC8A3	14

hsa04064	NF-kappa B signaling pathway	19/554	104/8101	6.72E-05	0.004286	0.003677	CARD11/CCL13/CCL19/CCL4/CCL4L2/CD14/CXCL12 /CXCL2/DDX58/EDARADD/GADD45A/GADD45B/LBP/NFKB2/ NFKBIA/PTGS2/RELA/TRADD/TRAF5	19
hsa05146	Amoebiasis	18/554	102/8101	0.000165	0.008759	0.007515	CD14/CD1B/CD1C/CD1D/CD1E/COL1A2/CXCL2 /IL10/IL12B/IL6/LAMA3/LAMB1/PLCB2/PLCB4/ RAB7B/RELA/SERPINB9/TLR2	18
hsa05323	Rheumatoid arthritis	16/554	93/8101	0.000505	0.020105	0.017249	ACP5/ATP6V0E2/CCL3/CCL5/CSF1/CTSK/CTSL/ CXCL12/CXCL2/HLA-DMB/HLA-DRA/IL6/MMP1 /TLR2/TNFSF13/VEGFA	16
hsa05133	Pertussis	14/554	76/8101	0.000549	0.020105	0.017249	C1QC/C2/C3/C4B/CD14/IL10/IL12B/IL6/IRF8/MAPK11 /NLRP3/NOD1/RELA/SERPING1	14
hsa04620	Toll-like receptor signaling pathway	17/554	104/8101	0.00063	0.020105	0.017249	CCL3/CCL4/CCL4L2/CCL5/CD14/CTSK/CXCL10/ CXCL11/CXCL9/IL12B/IL6/LBP/MAPK11/ NFKBIA/RELA/TLR2/TLR7	17
hsa05140	Leishmaniasis	14/554	77/8101	0.00063	0.020105	0.017249	C3/FCGR1A/FCGR2A/FCGR3A/HLA-DMB/HLA-DRA /IL10/IL12B/MAPK11/NCF2/NFKBIA/PTGS2/RELA/TLR2	14
hsa04623	Cytosolic DNA-sensing pathway	12/554	63/8101	0.00099	0.028712	0.024634	AIM2/CCL4/CCL4L2/CCL5/CXCL10/DDX58/IL6/ MB21D1/NFKBIA/POLR3B/RELA/TMEM173	12
hsa05202	Transcriptional misregulation in cancer	25/554	192/8101	0.001329	0.032473	0.02786	ARNT2/BMP2K/CCND2/CD14/CEBPA/DDIT3/FCGR1A /FUT8/GADD45A/GADD45B/HHEX/HPGD/IGFBP3/IL6/ ITGB7/KLF3/LMO2/MEF2C/MLLT3/NUPR1/PAX5/PLAT/RELA/ RUNX2/SPINT1	25

hsa05163	Human cytomegalovirus infection	28/554	225/8101	0.001419	0.032473	0.02786	BCAR1/CCL3/CCL4/CCL4L2/CCL5/CCND1/CCR5/CTNNB1/CXCL12/FAS/GNG12/GNG2/IL6/IL6R/MAPK11/MB21D1/NFKBIA/PLCB2/PLCB4/PTGS2/PTK2B/PXN/RAC2/RELA/TMEM173/TRADD/TRAF5/VEGFA	28
hsa05134	Legionellosis	11/554	57/8101	0.001425	0.032473	0.02786	BNIP3/C3/CD14/CXCL2/IL12B/IL6/NFKB2/NFKBIA/NLRC4/RELA/TLR2	11
hsa04610	Complement and coagulation cascades	14/554	85/8101	0.00172	0.034844	0.029894	C1QC/C2/C3/C3AR1/C4B/CFB/CFD/CFH/CLU/F3/PLAT/SERPINF2/SERPING1/VTN	14
hsa04625	C-type lectin receptor signaling pathway	16/554	104/8101	0.001748	0.034844	0.029894	CARD9/CCL17/CCL22/CLEC7A/EGR2/IL10/IL12B/IL6/LSP1/MAPK11/NFKB2/NFKBIA/NLRP3/PTGS2/RELA/RRAS2	16
hsa05150	Staphylococcus aureus infection	15/554	96/8101	0.002057	0.038604	0.03312	C1QC/C2/C3/C3AR1/C4B/CFB/CFD/CFH/FCGR1A/FCGR2A/FCGR3A/HLA-DMB/HLA-DRA/IL10/KRT23	15
hsa04512	ECM-receptor interaction	14/554	88/8101	0.002411	0.04273	0.03666	COL1A2/COL6A1/COL6A2/DAG1/GP1BA/IBSP/ITGA11/ITGA2/ITGA7/ITGB7/ITGB8/LAMA3/LAMB1/VTN	14
hsa04640	Hematopoietic cell lineage	15/554	99/8101	0.002802	0.047042	0.04036	CD14/CD1B/CD1C/CD1D/CD1E/CD38/CSF1/FCGR1A/GP1BA/HLA-DMB/HLA-DRA/IL2RA/IL6/IL6R/ITGA2	15
hsa01523	Antifolate resistance	7/554	31/8101	0.004181	0.066691	0.057217	ABCC2/ABCC3/FOLR2/IL6/RELA/SHMT2/SLC19A1	7
hsa04657	IL-17 signaling pathway	14/554	94/8101	0.004481	0.068062	0.058393	CCL17/CXCL10/CXCL2/IL6/MAPK11/MMP1/NFKBIA/PTGS2/RELA/S100A8/S100A9/TRADD/TRAF3IP2/TRAF5	14

hsa04672	Intestinal immune network for IgA production	9/554	49/8101	0.005353	0.077612	0.066587	CXCL12/HLA-DMB/HLA-DRA/ICOSLG/IL10/IL15RA/IL6/ITGB7/TNFSF13	9
hsa04621	NOD-like receptor signaling pathway	22/554	181/8101	0.005875	0.081482	0.069907	AIM2/ANTXR2/CARD16/CARD9/CASP5/CCL5/CXCL2/GBP7/IL6/MAPK11/MEFV/NAMPT/NFKBIA/NLRC4/NLRP3/NOD1/PANX1/PLCB2/PLCB4/RELA/TMEM173/TRAF 5	22
hsa04630	JAK-STAT signaling pathway	20/554	162/8101	0.007092	0.094266	0.080875	CCND1/CCND2/IFNLR1/IL10/IL12B/IL15RA/IL21R/IL27RA/IL2RA/IL6/IL6R/JAK3/LIFR/OSM/OSMR/PIM1/PRLR/PTPN2/SOCS2/SOCS3	20
hsa04068	FoxO signaling pathway	17/554	131/8101	0.007721	0.095698	0.082104	BNIP3/CCND1/CCND2/CCNG2/CDKN2B/CDKN2D/FOXO4/GADD45A/GADD45B/HOMER1/IL10/IL6/MAPK11/PCK2/SGK1/SMAD3/TNFSF10	17
hsa04933	AGE-RAGE signaling pathway in diabetic complications	14/554	100/8101	0.0078	0.095698	0.082104	CCND1/COL1A2/EDN1/EGR1/F3/IL6/MAPK11/MMP2/PIM1/PLCB2/PLCB4/RELA/SMAD3/VEGFA	14
hsa04668	TNF signaling pathway	15/554	112/8101	0.00901	0.105516	0.090527	CCL5/CSF1/CXCL10/CXCL2/EDN1/FAS/IL6/MAPK11/NFKBIA/PTGS2/RELA/RPS6KA5/SOCS3/TRADD/TRAF5	15
hsa05142	Chagas disease	14/554	102/8101	0.009262	0.105516	0.090527	C1QC/C3/CCL3/CCL5/FAS/IL10/IL12B/IL6/MAPK11/NFKBIA/PLCB2/PLCB4/RELA/TLR2	14
hsa04974	Protein digestion and absorption	14/554	103/8101	0.010069	0.109007	0.093522	ATP1B1/ATP1B2/COL1A2/COL24A1/COL6A1/COL6A2/COL8A2/DPP4/FXYD2/MEP1A/SLC16A10/SLC1A5/SLC7A8/SLC8A3	14

hsa04151	PI3K-Akt signaling pathway	36/554	354/8101	0.010251	0.109007	0.093522	BRCA1/CCND1/CCND2/CCNE1/COL1A2/COL6A1/COL6A2 /CSF1/DDIT4/EPHA2/EREG/GNG12/GNG2/IBSP/IL2RA/ IL6/IL6R/ITGA11/ITGA2/ITGA7/ITGB7/ITGB8/JAK3/LAMA3/ LAMB1/OSM/OSMR/PCK2/PIK3CG/PIK3R6/PRLR/RELA /SGK1/TLR2/VEGFA/VTN	36
hsa04371	Apelin signaling pathway	17/554	137/8101	0.011893	0.116654	0.100083	CCND1/CTGF/EGR1/GNG12/GNG2/HDAC4/MEF2C /NOTCH3/PIK3CG/PIK3R6/PLAT/PLCB2/PLCB4/ RRAS2/RYR1/SLC8A3/SMAD3	17
hsa05135	Yersinia infection	17/554	137/8101	0.011893	0.116654	0.100083	BAIAP2/BCAR1/ELMO2/FCGR2A/IL10/IL6/LIMK1 /MAPK11/MEFV/NFKBIA/NLRC4/NLRP3/PTK2B /PXN/RAC2/RELA/VAV3	17
hsa05321	Inflammatory bowel disease	10/554	65/8101	0.012176	0.116654	0.100083	HLA-DMB/HLA-DRA/IL10/IL12B/IL21R/IL6/RELA/ RORA/SMAD3/TLR2	10
hsa04360	Axon guidance	21/554	182/8101	0.012433	0.116654	0.100083	CXCL12/ENAH/EPHA2/EPHA7/EPHB6/LIMK1/LIMK2 /NRP1/NTNG2/PLXNA1/PLXNA2/PLXNC1/RAC2/ RND1/ROBO3/SEMA4A/SEMA4B/SEMA4C/SLIT2/ SRGAP1/UNC5C	21
hsa04973	Carbohydrate digestion and absorption	8/554	47/8101	0.01336	0.119544	0.102562	AMY2B/ATP1B1/ATP1B2/FXYD2/HK3/PLCB2 /PLCB4/SLC2A5	8
hsa04380	Osteoclast differentiation	16/554	128/8101	0.013504	0.119544	0.102562	ACP5/CSF1/CTSK/FCGR1A/FCGR2A/FCGR3A/GAB2 /LILRA2/LILRA5/LILRB5/MAPK11/NCF2/NFKB2/ NFKBIA/RELA/SOCS3	16
hsa04659	Th17 cell differentiation	14/554	107/8101	0.013866	0.119544	0.102562	HLA-DMB/HLA-DRA/IL21R/IL27RA/IL2RA/IL6/IL6R /IRF4/JAK3/MAPK11/NFKBIA/RELA/RORA/SMAD3	14

hsa05171	Coronavirus disease - COVID-19	25/554	232/8101	0.015421	0.126547	0.10857	C1QC/C2/C3/C3AR1/C4B/CFB/CFD/CXCL10/DDX58/FCGR2A/IL12B/IL6/IL6R/ISG15/MAPK11/MB21D1/MMP1/NFKBIA/NLRP3/NRP1/RELA/RPS10-NUDT3/TLR2/TLR7/TMEM173	25
hsa04145	Phagosome	18/554	152/8101	0.015471	0.126547	0.10857	ATP6V0E2/C3/CD14/CLEC7A/CTSL/FCGR1A/FCGR2A/FCGR3A/HLA-DMB/HLA-DRA/ITGA2/MRC1/NCF2/RAB7B/TLR2/TUBA4A/TUBB3/TUBB6	18
hsa04510	Focal adhesion	22/554	201/8101	0.018862	0.148851	0.127706	BCAR1/CCND1/CCND2/COL1A2/COL6A1/COL6A2/CTNNB1/IBSP/ITGA11/ITGA2/ITGA7/ITGB7/ITGB8/LAMA3/LAMB1/PXN/RAC2/RASGRF1/TLN2/VAV3/VEGFA/VTN	22
hsa05169	Epstein-Barr virus infection	22/554	202/8101	0.019862	0.148851	0.127706	CCND1/CCND2/CCNE1/CXCL10/DDX58/FAS/GADD45A/GADD45B/HLA-DMB/HLA-DRA/IL6/ISG15/JAK3/MAPK11/NEDD4/NFKB2/NFKBIA/RELA/RUNX3/TLR2/TRADD/TRAF5	22
hsa04622	RIG-I-like receptor signaling pathway	10/554	70/8101	0.019908	0.148851	0.127706	CXCL10/DDX58/DHX58/IL12B/ISG15/MAPK11/NFKBIA/RELA/TMEM173/TRADD	10
hsa05145	Toxoplasmosis	14/554	112/8101	0.020065	0.148851	0.127706	CCR5/HLA-DMB/HLA-DRA/IL10/IL12B/LAMA3/LAMB1/LDLR/MAPK11/NFKBIA/PIK3CG/PIK3R6/RELA/TLR2	14
hsa00534	Glycosaminoglycan biosynthesis - heparan sulfate / heparin	5/554	24/8101	0.02107	0.149622	0.128367	EXTL2/HS3ST3A1/HS3ST3B1/NDST2/XYLT1	5
hsa05152	Tuberculosis	20/554	180/8101	0.021107	0.149622	0.128367	C3/CARD9/CD14/CLEC7A/FCGR1A/FCGR2A/FCGR3A/HLA-DMB/HLA-DRA/IL10/IL12B/IL6/IRAK2/LBP/LSP1/MAPK11/MRC1/RELA/TLR2/TRADD	20

hsa05222	Small cell lung cancer	12/554	92/8101	0.022261	0.154376	0.132446	CCND1/CCNE1/CDKN2B/GADD45A/GADD45B/ITGA2/LAMA3/LAMB1/NFKBIA/PTGS2/RELA/TRAF5	12
hsa00360	Phenylalanine metabolism	4/554	17/8101	0.025194	0.170998	0.146706	IL4I1/MAOA/MAOB/MIF	4
hsa04961	Endocrine and other factor-regulated calcium reabsorption	8/554	53/8101	0.026351	0.175125	0.150247	ATP1B1/ATP1B2/ATP2B4/FXYD2/KL/PLCB2/PLCB4/SLC8A3	8
hsa04010	MAPK signaling pathway	29/554	294/8101	0.028695	0.186811	0.160274	CACNA1G/CACNA1I/CD14/CDC25B/CSF1/DDIT3/DUSP1/DUSP4/DUSP7/EPHA2/EREG/FAS/GADD45A/GADD45B/GNG12/MAP3K13/MAPK11/MEF2C/NFKB2/PLA2G4B/PTPN7/RAC2/RASGRF1/RELA/RPS6KA5/RRAS2/STK3/TRADD/VEGFA	29
hsa04666	Fc gamma R-mediated phagocytosis	12/554	97/8101	0.032183	0.205328	0.17616	FCGR1A/FCGR2A/FCGR3A/GAB2/GSN/LIMK1/LIMK2/MARCKS/MYO10/PLA2G4B/RAC2/VAV3	12
hsa05412	Arrhythmogenic right ventricular cardiomyopathy	10/554	77/8101	0.036051	0.223058	0.191371	ATP2A3/CTNNB1/DAG1/ITGA11/ITGA2/ITGA7/ITGB7/ITGB8/SLC8A3/TCF7L1	10
hsa00250	Alanine; aspartate and glutamate metabolism	6/554	37/8101	0.037759	0.223058	0.191371	ADSSL1/ASNS/ASS1/GPT2/IL4I1/RIMKLB	6
hsa05143	African trypanosomiasis	6/554	37/8101	0.037759	0.223058	0.191371	FAS/IL10/IL12B/IL6/PLCB2/PLCB4	6
hsa05216	Thyroid cancer	6/554	37/8101	0.037759	0.223058	0.191371	CCND1/CTNNB1/GADD45A/GADD45B/RET/TCF7L1	6

hsa05167	Kaposi sarcoma-associated herpes virus infection	20/554	193/8101	0.04042	0.234435	0.201132	C3/CCND1/CCR4/CCR5/CTNNB1/CXCL2/FAS/ GNG12/GNG2/IL6/MAPK11/NFKBIA/PIK3CG /PIK3R6/PTGS2/RELA/TCF7L1/TRADD/VEGFA/ZFP36	20
hsa04110	Cell cycle	14/554	124/8101	0.043206	0.245979	0.211036	ANAPC1/CCND1/CCND2/CCNE1/CDC14B/CDC25B /CDKN1C/CDKN2B/CDKN2D/DBF4/GADD45A/ GADD45B/MCM2/SMAD3	14
hsa05166	Human T-cell leukemia virus 1 infection	22/554	219/8101	0.043952	0.245979	0.211036	ANAPC1/CCND1/CCND2/CCNE1/CDKN2B/EGR1 /EGR2/HLA-DMB/HLA-DRA/IL15RA/IL2RA/IL6/ JAK3/KAT2A/NFKB2/NFKBIA/NRP1/RELA/ SLC25A4/SMAD3/TLN2/ZFP36	22
hsa04658	Th1 and Th2 cell differentiation	11/554	92/8101	0.048439	0.26323	0.225837	DLL4/HLA-DMB/HLA-DRA/IL12B/IL2RA/JAK3/MAPK11 /NFKBIA/NOTCH3/RELA/RUNX3	11
hsa05131	Shigellosis	24/554	246/8101	0.048685	0.26323	0.225837	BCAR1/BNIP3/C3/CCL5/CD14/ELMO2/FOXO4 /HK3/MAPK11/MB21D1/NFKBIA/NLRC4/NLRP3 /NOD1/PFN2/PLCB2/PLCB4/PXN/RELA/ RPS6KA5/TLN2/TMEM173/TRADD/TRAF5	24

M1-MDM Reactome enrichment (IFN-L3 vs NT)

PATH_ID	Description	GeneRatio	BgRatio	pvalue	p.adjust	qvalue	geneID	Count
1442490	Collagen degradation	15/470	62/6750	1.54E-05	0.012297	0.012132	ADAM10/COL23A1/COL6A1/COL6A3/COL7A1/COL8A2/COL9A2/CTSK/ CTSL/MMP1/MMP10/MMP12/MMP19/MMP2/MMP8MMP8	15
1474228	Degradation of the extracellular matrix	19/470	109/6750	0.00016	0.063649	0.06279	ADAM10/ADAM15/ADAM8/COL23A1/COL6A1/COL6A3/ COL7A1/COL8A2/COL9A2/CTSK/CTSL/LAMB1/MMP1/MMP10/M MP12/MMP19/MMP2/MMP8/TIMP1	19

1368108	BMAL1:CLOCK; NPAS2 activates circadian gene expression	6/470	18/6750	0.000995	0.205507	0.202735	NAMPT/NPAS2/NR1D1/PER1/PER2/RORA	**6**
1474244	Extracellular matrix organization	31/470	249/6750	0.001033	0.205507	0.202735	ADAM10/ADAM15/ADAM8/ADAMTS2/COL23A1/COL6A1/COL6A3/COL7A1/COL8A2/COL9A2/CTSK/CTSL/DAG1/EFEMP2/IBSP/ITGA11/ITGA3/ITGAV/ITGB7/ITGB8/LAMB1/LUM/MMP1/MMP10/MMP12/MMP19/MMP2/MMP8/PLOD2/TIMP1/VCAN	31
216083	Integrin cell surface interactions	14/470	83/6750	0.001593	0.212229	0.209366	COL23A1/COL6A1/COL6A3/COL7A1/COL8A2/COL9A2/DAG1/IBSP/ITGA11/ITGA3/ITGAV/ITGB7/ITGB8/LUM	14
168898	Toll-Like Receptors Cascades	17/470	111/6750	0.0016	0.212229	0.209366	BIRC3/CD14/CD36/CDK1/CTSK/CTSL/DUSP4/DUSP7/IRAK2/LGMN/MEF2C/NFKBIA/NOD2/PELI1/PELI2/TLR2/TLR7	17
166658	Complement cascade	8/470	36/6750	0.002773	0.308973	0.304805	C5/CD55/CFB/CFD/CFH/CR1/FCN1/PROS1	**8**
202733	Cell surface interactions at the vascular wall	15/470	99/6750	0.003287	0.308973	0.304805	ATP1B1/ATP1B2/CEACAM8/CXADR/FYN/ITGA3/ITGAV/MERTK/MMP1/PROS1/SELL/SELPLG/SPN/THBD/TREM1	15
166058	MyD88:Mal cascade initiated on plasma membrane	12/470	74/6750	0.004696	0.308973	0.304805	CD14/CD36/CDK1/DUSP4/DUSP7/IRAK2/MEF2C/NFKBIA/NOD2/PELI1/PELI2/TLR2	12
168179	Toll Like	12/470	74/6750	0.004696	0.308973	0.304805	CD14/CD36/CDK1/DUSP4/DUSP7/IRAK2/MEF2C/NFKBIA/NOD2/PELI1/PELI2/TLR2	12

	Receptor TLR1:TLR2 Cascade							
168188	Toll Like Receptor TLR6:TLR2 Cascade	12/470	74/6750	0.00469621889992871	0.308972945343179	0.304805365281687	CD14/CD36/CDK1/DUSP4/DUSP7/IRAK2/MEF2C/NFKBIA/NOD2/PELI1/PELI2/TLR2	12
**181438**	Toll Like Receptor 2 (TLR2) Cascade	12/470	74/6750	0.00469621889992871	0.308972945343179	0.304805365281687	CD14/CD36/CDK1/DUSP4/DUSP7/IRAK2/MEF2C/NFKBIA/NOD2/PELI1/PELI2/TLR2	12
**977606**	Regulation of Complement cascade	6/470	24/6750	0.00504604056464991	0.308972945343179	0.304805365281687	C5/CD55/CFB/CFH/CR1/PROS1	6
**425366**	Transport of glucose and other sugars; bile salts and organic acids; metal ions and amine compounds	14/470	95/6750	0.00572069189733502	0.325262196448477	0.320874898903152	CP/RHCG/SLC11A1/SLC16A7/SLC22A16/SLC22A4/SLC2A1/SLC2A13/SLC2A3/SLC2A5/SLC30A3/SLC39A8/SLC47A1/SLC6A12	14
**1679131**	Trafficking and processing of endosomal TLR	4/470	12/1/6750	0.00732471819872573	0.388698379079046	0.383455422894695	CTSK/CTSL/LGMN/TLR7	4
**109582**	Hemostasis	45/470	450/6750	0.00782558813213462	0.389323009573697	0.384071628063975	AKAP1/ATP1B1/ATP1B2/ATP2B1/CD36/CEACAM8/CFD/CXADR/DAGLA/DOCK6/FYN/GNA14/GNA15/GP1BA/GUCY1A3/HMG20B/HSPA5/ITGA3/ITGAV/KCNMB3/MERTK/MMP1/P2RY1/PIK3CG/PLAT/PLAU/PLAUR/PRKAR2B/PROS1/PTGIR/RHOB/SELL/SELPLG/SERPINB2/SERPINE2/SLC8A3/SPN/SRGN/TBXA2R/THBD/TIMP1/TREM1/TUBA4A/VAV3/VEGFA	45
**1592389**	Activation of Matrix Metalloproteinases	6/470	27/6750	0.00929162875607344	0.435066852343204	0.429198455234105	CTSK/MMP1/MMP10/MMP2/MMP8/TIMP1	6
**166054**	Activated TLR4 signalling	13/470	93/6750	0.0117520737963916	0.519702818995983	0.512692810064802	BIRC3/CD14/CD36/CDK1/DUSP4/DUSP7/IRAK2/MEF2C/NFKBIA/NOD2/PELI1/PELI2/TLR2	13
**449147**	Signaling by Interleukins	14/470	107/6750	0.0158874745902904	0.632321488693559	0.623792423387193	CDK1/FYN/GAB2/IL1R2/IL1RN/IL2RA/IL6/IRAK2/JAK3/MAP3K8/NOD2/PELI1/PELI2/SOCS3	14
**351202**	Metabolism of polyamines	4/470	15/6750	0.0170973040927769	0.648069240850021	0.639327762065744	APIP/AZIN2/SMOX/SRM	4

**975138**	TRAF6 mediated induction of NFkB and MAP kinases upon TLR7/8 or 9 activation	10/470	68/6750	0.0183836233598932	0.665152917930681	0.65618100605169	CDK1/DUSP4/DUSP7/IRAK2/MEF2C/NFKBIA/NOD2/PELI1/PELI2/TLR7	10
168181	Toll Like Receptor 7/8 (TLR7/8) Cascade	10/470	69/6750	0.0202125872610145	0.67038414415698	0.661341670908631	CDK1/DUSP4/DUSP7/IRAK2/MEF2C/NFKBIA/NOD2/PELI1/PELI2 /TLR7	10
975155	MyD88 dependent cascade initiated on endosome	10/470	69/6750	0.020213	0.670384	0.661342	CDK1/DUSP4/DUSP7/IRAK2/MEF2C/NFKBIA/NOD2/PELI1 /PELI2/TLR7	10
71387	Metabolism of carbohydrates	26/470	247/6750	0.02174	0.692205	0.682868	ALDOC/CHST14/DSE/ENO2/GAPDH/GOT2/HAS3/HMMR/ HPSE/HS3ST1/HS3ST3A1/HS3ST3B1/HS6ST1/LUM/NDST2/NUP18 8/NUP35/PAPSS2/PFKFB3/PGK1/PGM1/PGM2/SLC2A1/SLC2A3/S LC2A5/VCAN	26
168138	Toll Like Receptor 9 (TLR9) Cascade	10/470	71/6750	0.024264	0.705688	0.696169	CDK1/DUSP4/DUSP7/IRAK2/MEF2C/NFKBIA/NOD2 /PELI1/PELI2/TLR7	10
1989781	PPARA activates gene expression	7/470	42/6750	0.024407	0.705688	0.696169	ANGPTL4/ANKRD1/CD36/CTGF/NPAS2/PLIN2/TNFRSF21	**7**
210991	Basigin interactions	5/470	25/6750	0.026643	0.705688	0.696169	ATP1B1/ATP1B2/ITGA3/MMP1/SPN	**5**
381426	Regulation of Insulin-like Growth Factor (IGF) transport and uptake by Insulin-like Growth Factor Binding Proteins (IGFBPs)	4/470	17/6750	0.026696	0.705688	0.696169	IGFBP2/IGFBP3/MMP1/MMP2	**4**

392518	Signal amplification	4/470	17/6750	0.026696	0.705688	0.696169	GNA14/GNA15/P2RY1/TBXA2R	**4**
418592	ADP signalling through P2Y purinoceptor 1	3/470	Oct-50	0.027843	0.705688	0.696169	GNA14/GNA15/P2RY1	**3**
400253	Circadian Clock	6/470	35/6750	0.031866	0.705688	0.696169	NAMPT/NPAS2/NR1D1/PER1/PER2/RORA	**6**
375165	NCAM signaling for neurite out-growth	9/470	64/6750	0.031984	0.705688	0.696169	CACNA1G/CACNB3/CDK1/COL6A1/COL6A3/COL9A2 /FYN/SPTBN1/ST8SIA4	9
446652	Interleukin-1 signaling	7/470	45/6750	0.034355	0.705688	0.696169	IL1R2/IL1RN/IRAK2/MAP3K8/NOD2/PELI1/PELI2	**7**
2022928	HS-GAG biosynthesis	5/470	27/6750	0.036132	0.705688	0.696169	HS3ST1/HS3ST3A1/HS3ST3B1/HS6ST1/NDST2	**5**
70171	Glycolysis	5/470	27/6750	0.036132	0.705688	0.696169	ALDOC/ENO2/GAPDH/PFKFB3/PGK1	**5**
176974	Unwinding of DNA	3/470	11/1/6750	0.036348	0.705688	0.696169	GINS1/MCM2/MCM8	**3**

2022923	Dermatan sulfate biosynthesis	3/470	11/1/6750	0.036348	0.705688	0.696169	CHST14/DSE/VCAN	3
5602498	MyD88 deficiency (TLR2/4)	3/470	11/1/6750	0.036348	0.705688	0.696169	CD14/CD36/TLR2	**3**
5603041	IRAK4 deficiency (TLR2/4)	3/470	11/1/6750	0.036348	0.705688	0.696169	CD14/CD36/TLR2	**3**
73817	Purine ribonucleoside monophosphate biosynthesis	3/470	11/1/6750	0.036348	0.705688	0.696169	ADSSL1/ATIC/PFAS	**3**
380108	Chemokine receptors bind chemokines	8/470	56/6750	0.038633	0.732055	0.72218	ACKR3/CCL20/CCL22/CCL4/CXCL13/CXCL2/CXCL3/CXCL9	**8**
168142	Toll Like Receptor 10 (TLR10) Cascade	9/470	67/6750	0.041385	0.732055	0.72218	CDK1/DUSP4/DUSP7/IRAK2/MEF2C/NFKBIA/NOD2/PELI1/PELI2	9
168176	Toll Like Receptor 5 (TLR5) Cascade	9/470	67/6750	0.041385	0.732055	0.72218	CDK1/DUSP4/DUSP7/IRAK2/MEF2C/NFKBIA/NOD2/PELI1/PELI2	9
975871	MyD88 cascade initiated on plasma membrane	9/470	67/6750	0.041385	0.732055	0.72218	CDK1/DUSP4/DUSP7/IRAK2/MEF2C/NFKBIA/NOD2/PELI1/PELI2	9
419037	NCAM1 interactions	6/470	38/6750	0.045529	0.73944	0.729466	CACNA1G/CACNB3/COL6A1/COL6A3/COL9A2/ST8SIA4	**6**
1502540	Signaling by Activin	3/470	12/1/6750	0.046023	0.73944	0.729466	ACVR2B/FST/SMAD3	**3**
428790	Facilitative Na+-independent	3/470	12/1/6750	0.046023	0.73944	0.729466	SLC2A1/SLC2A3/SLC2A5	**3**

	glucose transporters							
381070	IRE1alpha activates chaperones	8/470	58/6750	0.046243	0.73944	0.729466	DNAJB9/HSPA5/HYOU1/PLA2G4B/PPP2R5B/SRPRB/TSPYL2/WIPI1	8
73777	RNA Polymerase I Chain Elongation	5/470	29/6750	0.047461	0.73944	0.729466	GTF2H2C/POLR1A/POLR1B/POLR1C/POLR1E	**5**

M1-MDM Reactome enrichment (IFN-L4 vs NT)

PATH_ID	Description	GeneRatio	BgRatio	pvalue	p.adjust	qvalue	geneID	Count
380108	Chemokine receptors bind chemokines	10/155	56/6750	4.38E-07	0.000177	0.000166	ACKR3/CCL17/CCL19/CCR4/CXCL1/CXCL10/CXCL11 /CXCL3/CXCL6/CXCL9	10
202733	Cell surface interactions at the vascular wall	9/155	99/6750	0.000418	0.084564	0.078904	CEACAM8/CXADR/GAS6/ITGA6/ITGAV/MERTK /SELPLG/SLC7A6/TREM1	9
917937	Iron uptake and transport	5/155	42/6750	0.002565	0.240229	0.224152	ABCG2/ATP6V0D2/CP/CYBRD1/MCOLN1	5
109582	Hemostasis	20/155	450/6750	0.003181	0.240229	0.224152	ABCC4/APBB1IP/ATP2A3/BCAR1/CD36/CEACAM8 /CLU/CXADR/GAS6/GNA14/HSPA5/ITGA6/ITGAV/ MERTK/P2RX1/SELPLG/SERPINB2/SLC7A6/TFPI/TREM1	20
375276	Peptide ligand-binding receptors	11/155	191/6750	0.004348	0.240229	0.224152	ACKR3/C5AR1/CCL17/CCL19/CCR4/CXCL1/CXCL10 /CXCL11/CXCL3/CXCL6/CXCL9	11
399719	Trafficking of AMPA receptors	4/155	30/6750	0.004603	0.240229	0.224152	AKAP5/DLG4/EPB41L1/MYO6	4
399721	Glutamate Binding; Activation of AMPA Receptors and Synaptic Plasticity	4/155	30/6750	0.004603	0.240229	0.224152	AKAP5/DLG4/EPB41L1/MYO6	4
352230	Amino acid transport across the plasma membrane	4/155	31/6750	0.005192	0.240229	0.224152	SLC16A10/SLC38A1/SLC38A5/SLC7A6	4

399956	CRMPs in Sema3A signaling	3/155	16/6750	0.005338	0.240229	0.224152	DPYSL3/NRP1/SEMA3A	3
211976	Endogenous sterols	3/155	17/6750	0.006374	0.251792	0.23494	CYP1B1/CYP27A1/CYP7B1	3
418594	G alpha (i) signalling events	11/155	203/6750	0.006839	0.251792	0.23494	ACKR3/C5AR1/CCL19/CCR4/CXCL1/CXCL10/CXCL11 /CXCL3/CXCL6/CXCL9/HCAR2	11
373076	Class A/1 (Rhodopsin-like receptors)	14/155	307/6750	0.010671	0.355592	0.331793	ACKR3/ADRB2/C5AR1/CCL17/CCL19/CCR4/CXCL1 /CXCL10/CXCL11/CXCL3/CXCL6/CXCL9/GPBAR1/HCAR2	14
1474244	Extracellular matrix organization	12/155	249/6750	0.011789	0.355592	0.331793	COL23A1/COL5A3/FBN2/ITGA6/ITGAV/LAMA3/MMP10 /MMP7/MMP8/PLOD2/VCAN/VTN	12
1474228	Degradation of the extracellular matrix	7/155	109/6750	0.012412	0.355592	0.331793	COL23A1/COL5A3/FBN2/LAMA3/MMP10/MMP7/MMP8	7
1442490	Collagen degradation	5/155	62/6750	0.013533	0.355592	0.331793	COL23A1/COL5A3/MMP10/MMP7/MMP8	5
1989781	PPARA activates gene expression	4/155	42/6750	0.015208	0.355592	0.331793	ANKRD1/CD36/PLIN2/TNFRSF21	4
373755	Semaphorin interactions	5/155	65/6750	0.016357	0.355592	0.331793	DPYSL3/MET/NRP1/SEMA3A/SEMA7A	5
977606	Regulation of Complement cascade	3/155	24/6750	0.016876	0.355592	0.331793	C4B/C4B_2/VTN	3
3000178	ECM proteoglycans	5/155	67/6750	0.018444	0.355592	0.331793	COL5A3/ITGAV/LAMA3/VCAN/VTN	**4**

3000170	Syndecan interactions	3/155	25/6750	0.018861	0.355592	0.331793	COL5A3/ITGAV/VTN	3
446107	Type I hemidesmosome assembly	2/155	101/6750	0.02089	0.355592	0.331793	ITGA6/LAMA3	2
1474290	Collagen formation	5/155	70/6750	0.021891	0.355592	0.331793	COL23A1/COL5A3/ITGA6/LAMA3/PLOD2	5
1592389	Activation of Matrix Metalloproteinases	3/155	27/6750	0.023202	0.355592	0.331793	MMP10/MMP7/MMP8	3
157052	NICD traffics to nucleus	2/155	111/6750	0.025152	0.355592	0.331793	MAML3/NOTCH3	2
350054	Notch-HLH transcription pathway	2/155	111/6750	0.025152	0.355592	0.331793	MAML3/NOTCH3	2
381042	PERK regulates gene expression	2/155	111/6750	0.025152	0.355592	0.331793	HERPUD1/HSPA5	2
5602498	MyD88 deficiency (TLR2/4)	2/155	111/6750	0.025152	0.355592	0.331793	CD14/CD36	2
5603041	IRAK4 deficiency (TLR2/4)	2/155	111/6750	0.025152	0.355592	0.331793	CD14/CD36	2
425374	**Amino acid and oligopeptide** SLC transporters	4/155	49/6750	0.025462	0.355592	0.331793	SLC16A10/SLC38A1/SLC38A5/SLC7A6	4

194138	Signaling by VEGF	6/155	100/6750	0.027067	0.365408	0.340952	AXL/BCAR1/ITGAV/NCKAP1/NRP1/SHB		6
204174	Regulation of pyruvate dehydrogenase (PDH) complex	2/155	**121/6750**	**0.029734**	0.387828	0.361872	PDK1/PDK4		2
3000157	Laminin interactions	3/155	30/6750	0.030643	0.387828	0.361872	ITGA6/ITGAV/LAMA3		3
3000171	Non-integrin membrane-ECM interactions	4/155	53/6750	0.032816	0.397497	0.370894	COL5A3/ITGAV/LAMA3/VTN		4
1296065	Inwardly rectifying K+ channels	3/155	31/6750	0.03337	0.397497	0.370894	GNGT2/KCNJ1/KCNJ5		3
500792	GPCR ligand binding	15/155	392/6750	0.035022	0.40288	0.375917	ACKR3/ADRB2/C5AR1/CCL17/CCL19/CCR4/CXCL1/CXCL10/CXCL11 /CXCL3/CXCL6/CXCL9/GPBAR1/HCAR2/WNT5A		15
422475	Axon guidance	12/155	292/6750	0.035812	0.40288	0.375917	ABLIM3/CACNA1I/COL5A3/DLG4/DPYSL3/EPHA7 /ITGAV/MET/NRP1/SEMA3A/SEMA7A/SLIT1		12
112314	Neurotransmitter Receptor Binding And Downstream Transmission In The Postsynaptic Cell	7/155	137/6750	0.037849	0.40499	0.377885	AKAP5/CHRNA1/DLG4/EPB41L1/GNGT2/KCNJ5/MYO6		7

114608	Platelet degranulation	5/155	81/6750	0.037999	0.40499	0.377885	ABCC4/CD36/CLU/GAS6/HSPA5	5
399955	SEMA3A-Plexin repulsion signaling by inhibiting Integrin adhesion	2/155	14/6750	0.03979	0.413209	0.385554	NRP1/SEMA3A	2
216083	Integrin cell surface interactions	5/155	83/6750	0.04154	0.420597	0.392448	COL23A1/COL5A3/ITGA6/ITGAV/VTN	5
1266738	Developmental Biology	16/155	438/6750	0.04304	0.425152	0.396698	ABLIM3/ACVR2B/CACNA1I/CD36/COL5A3/DLG4/DPYSL3/EPAS1/EPHA7/FABP4/ITGAV/MET/NRP1/SEMA3A/SEMA7A/SLIT1	16
372708	p130Cas linkage to MAPK signaling for integrins	2/155	15/6750	0.045233	0.428439	0.399765	APBB1IP/BCAR1	2
2129379	Molecules associated with elastic fibres	3/155	35/6750	0.045489	0.428439	0.399765	FBN2/ITGAV/VTN	3
76005	Response to elevated platelet cytosolic Ca2+	5/155	86/6750	0.047216	0.429745	0.400984	ABCC4/CD36/CLU/GAS6/HSPA5	5
400206	Regulation of lipid metabolism by Peroxisome proliferator-activated receptor alpha (PPARalpha)	4/155	60/6750	0.048388	0.429745	0.400984	ANKRD1/CD36/PLIN2/TNFRSF21	4
166658	Complement cascade	3/155	36/6750	0.048815	0.429745	0.400984	C4B/C4B_2/VTN	3

M1-MDM Reactome enrichment (IFN-L4 vs IFN-L3)

PATH_ID	Description	GeneRatio	BgRatio	pvalue	p.adjust	qvalue	geneID	Count
380108	Chemokine receptors bind chemokines	17/492	56/6750	2.39E-07	0.000187	0.000179	ACKR3/CCL17/CCL19/CCL22/CCL3/CCL4/CCL5/CCR4/CCR5/ CCR6/CCR7/CXCL10/CXCL11/CXCL12/CXCL13/CXCL2/CXCL9	17
166663	Initial triggering of complement	8/492	19/6750	2.77E-05	0.010855	0.010361	C1QC/C2/C3/C4B/C4B_2/CFB/CFD/FCN1	8
166658	Complement cascade	10/492	36/6750	0.000174	0.045409	0.043342	C1QC/C2/C3/C4B/C4B_2/CFB/CFD/CFH/FCN1/VTN	10
1989781	PPARA activates gene expression	10/492	42/6750	0.000677	0.118931	0.113519	ABCB4/ANGPTL4/CTGF/GLIPR1/GRHL1/ME1/NPAS2/RGL1/ TNFRSF21	10
381426	Regulation of Insulin-like Growth Factor (IGF) transport and uptake by Insulin-like Growth Factor Binding Proteins (IGFBPs)	6/492	17/6750	0.000899	0.118931	0.113519	IGFBP2/IGFBP3/IGFBP4/IGFBP6/MMP1/MMP2	6
168898	Toll-Like Receptors Cascades	18/492	111/6750	0.00101	0.118931	0.113519	CD14/CTSK/CTSL/DUSP4/DUSP7/IRAK2/LBP/LGMN/MAPK1 1/ MEF2C/NFKB2/NFKBIA/NOD1/PELI1/RELA/RPS6KA5/TLR2/T LR7	18

422475	Axon guidance	36/492	292/6750	0.001158	0.118931	0.113519	ALCAM/CACNA1G/CACNA1I/COL6A1/COL6A2/DPYSL3/ENAH/EPHA2/EPHA7/EPHB6/EVL/ITGA2/ITSN1/LAMB1/LIMK1/MMP2/MYH10/MYH11/MYO10/NRP1/PLXNA1/PLXNA2/PLXNC1/RAC2/RHOB/RND1/ROBO3/RPS6KA5/SEMA4A/SIAH2/SLIT2/SPTBN1/SRGAP1/ST8SIA4/UNC5C/VAV3	36
977606	Regulation of Complement cascade	7/492	24/6750	0.001215	0.118931	0.113519	C2/C3/C4B/C4B_2/CFB/CFH/VTN	7
1442490	Collagen degradation	12/492	62/6750	0.001473	0.128133	0.122302	COL1A2/COL6A1/COL6A2/COL8A2/CTSK/CTSL/FURIN/MMP1/MMP12/MMP19/MMP2/MMP8	12
373755	Semaphorin interactions	12/492	65/6750	0.002249	0.133326	0.127258	DPYSL3/LIMK1/MYH10/MYH11/NRP1/PLXNA1/PLXNA2/PLXNC1/RAC2/RHOB/RND1/SEMA4A	12

166058	MyD88:Mal cascade initiated on plasma membrane	13/492	74/6750	0.002384	0.133326	0.127258	CD14/DUSP4/DUSP7/IRAK2/MAPK11/MEF2C/NFKB2/NFKBIA/NOD1/PELI1/RELA/RPS6KA5/TLR2	13
168179	Toll Like Receptor TLR1:TLR2 Cascade	13/492	74/6750	0.002384	0.133326	0.127258	CD14/DUSP4/DUSP7/IRAK2/MAPK11/MEF2C/NFKB2/NFKBIA/NOD1/PELI1/RELA/RPS6KA5/TLR2	13
168188	Toll Like Receptor TLR6:TLR2 Cascade	13/492	74/6750	0.002384	0.133326	0.127258	CD14/DUSP4/DUSP7/IRAK2/MAPK11/MEF2C/NFKB2/NFKBIA/NOD1/PELI1/RELA/RPS6KA5/TLR2	13
181438	Toll Like Receptor 2 (TLR2) Cascade	13/492	74/6750	0.002384	0.133326	0.127258	CD14/DUSP4/DUSP7/IRAK2/MAPK11/MEF2C/NFKB2/NFKBIA/NOD1/PELI1/RELA/RPS6KA5/TLR2	13
	TRAF6 mediated induction of NFkB and MAPkinases						DUSP4/DUSP7/IRAK2/MAPK11/MEF2C/NFKB2/NFKBIA/NOD1	
975138	upon TLR7/8 or 9 activation	12/492	68/6750	0.003331	0.170233	0.162487	/PELI1/RELA/RPS6KA5/TLR7	12
168181	Toll Like Receptor 7/8 (TLR7/8) Cascade	12/492	69/6750	0.003773	0.170233	0.162487	DUSP4/DUSP7/IRAK2/MAPK11/MEF2C/NFKB2/NFKBIA/NOD1/PELI1/RELA/RPS6KA5/TLR7	12
	MyD88 dependent cascade						DUSP4/DUSP7/IRAK2/MAPK11/MEF2C/NFKB2/NFKBIA/NOD1/PELI1	
975155	initiated on endosome	12/492	69/6750	0.003773	0.170233	0.162487	/RELA/RPS6KA5/TLR7	12
416572	Sema4D induced cell migration and growth-cone collapse	6/492	22/6750	0.003962	0.170233	0.162487	LIMK1/MYH10/MYH11/RAC2/RHOB/RND1	6

1474244	Extracellular matrix organization	30/492	249/6750	0.004131	0.170233	0.162487	ADAM15/BMP2/COL1A2/COL24A1/COL6A1/COL6A2/COL8A2/CTSK/CTSL /DAG1/EMILIN1/FBN2/FURIN/IBSP/ITGA11/ITGA2/ITGA7 /ITGB 7/ITGB8/LAMA3/LAMB1/LUM/MMP1/MMP12/MMP19/ MMP 2/MMP8/PLOD2/VCAN/VTN	30
168138	Toll Like Receptor 9 (TLR9) Cascade	12/492	71/6750	0.004799	0.187883	0.179333	DUSP4/DUSP7/IRAK2/MAPK11/MEF2C/NFKB2/NFKBIA/NOD1 /PELI1/RELA/RPS6KA5/TLR7	12
1474228	Degradation of the extracellular matrix	16/492	109/6750	0.005276	0.190646	0.18197	ADAM15/COL1A2/COL6A1/COL6A2/COL8A2/CTSK/CTSL/FBN2 / FURIN/LAMA3/LAMB1/MMP1/MMP12/MMP19/ MMP2/MMP 8	16
352230	Amino acid transport across the plasma membrane	7/492	31/6750	0.005916	0.190646	0.18197	SLC16A10/SLC1A5/SLC38A5/SLC6A12/SLC7A1 /SLC7A6/SLC7A8	7

198753	ERK/MAPK targets	5/492	17/6750	0.005978	0.190646	0.18197	DUSP4/DUSP7/MAPK11/MEF2C/RPS6KA5	5
622312	Inflammasomes	5/492	17/6750	0.005978	0.190646	0.18197	AIM2/MEFV/NLRC4/NLRP3/PANX1	5
397795	G-protein beta:gamma signalling	4/492	Nov-50	0.006087	0.190646	0.18197	GNG2/PIK3CG/PIK3R6/PLCB2	4
168180	TRAF6 Mediated Induction of proinflammatory cytokines	10/492	56/6750	0.006533	0.196752	0.187798	DUSP4/DUSP7/IRAK2/MAPK11/MEF2C/NFKB2 /NFKBIA/NOD1/RELA/RPS6KA5	10
535734	Fatty acid; triacylglycerol; and ketone body metabolism	17/492	123/6750	0.00757	0.198319	0.189294	ABCB4/ANGPTL4/BDH1/CTGF/DGAT2/ELOVL7/GLIPR1/GPAT3/ GPD1/GPD1L/GRHL1/MCEE/ME1/NPAS2/RGL1/TNFRSF21/TXNRD1	17
1368108	BMAL1:CLOCK;NPAS2 activates circadian geneexpression	5/492	18/6750	0.00779	0.198319	0.189294	NAMPT/NOCT/NPAS2/NR1D1/RORA	5
400685	Sema4D in semaphorin signaling	6/492	25/6750	0.007804	0.198319	0.189294	LIMK1/MYH10/MYH11/RAC2/RHOB/RND1	6
168142	Toll Like Receptor 10 (TLR10) Cascade	11/492	67/6750	0.00848	0.198319	0.189294	DUSP4/DUSP7/IRAK2/MAPK11/MEF2C/NFKB2/ NFKBIA/NOD1/PELI1/RELA/RPS6KA5	11
168176	Toll Like Receptor 5 (TLR5) Cascade	11/492	67/6750	0.00848	0.198319	0.189294	DUSP4/DUSP7/IRAK2/MAPK11/MEF2C/NFKB2 /NFKBIA/NOD1/PELI1/RELA/RPS6KA5	11
3000178	ECM proteoglycans	11/492	67/6750	0.00848	0.198319	0.189294	COL1A2/COL6A1/COL6A2/DAG1/IBSP/ITGA2/ITGA7 /LAMA3/LAMB1/VCAN/VTN	11
975871	MyD88 cascade initiated on plasma	11/492	67/6750	0.00848	0.198319	0.189294	DUSP4/DUSP7/IRAK2/MAPK11/MEF2C/NFKB2/NFKBIA/NOD1/PELI1/RELA/RPS6KA5	11

	membrane							
1679131	Trafficking and processing of endosomal TLR	4/492	Dec-50	0.008612	0.198319	0.189294	CTSK/CTSL/LGMN/TLR7	4
166016	Toll Like Receptor 4 (TLR4) Cascade	14/492	96/6750	0.009287	0.207771	0.198316	CD14/DUSP4/DUSP7/IRAK2/LBP/MAPK11/MEF2C/NFKB2 /NFKBIA/NOD1/PELI1/RELA/RPS6KA5/TLR2	14
400206	Regulation of lipid metabolism by Peroxisomeproliferator-activated receptor alpha (PPARalpha)	10/492	60/6750	0.010658	0.229065	0.218641	ABCB4/ANGPTL4/CTGF/GLIPR1/GRHL1/ME1/NPAS2 /RGL1/TNFRSF21/TXNRD1	10

1266738	Developmental Biology	45/492	438/6750	0.010824	0.229065	0.218641	ALCAM/ANGPTL4/CACNA1G/CACNA1I/CEBPA/CEBPD/COL6A1/COL6A2/CTNNB1/DPYSL3/ENAH/EPHA2/EPHA7/EPHB6/EVL/FURIN/ITGA2/ITSN1/KLF5/LAMB1/LIMK1/MAPK11/MEF2C/MMP2/MYH10/MYH11/MYO10/NRP1/PLXNA1/PLXNA2/PLXNC1/RAC2/RHOB/RND1/ROBO3/RPS6KA5/SEMA4A/SIAH2/SLIT2/SMAD3/SPTBN1/SRGAP1/ST8SIA4/UNC5C/VAV3	45
2029481	FCGR activation	4/492	13/6750	0.011734	0.241778	0.230776	FCGR1A/FCGR2A/FCGR3A/FGR	4
198725	Nuclear Events (kinase and transcriptionfactor activation)	5/492	20/6750	0.01249	0.250771	0.239359	DUSP4/DUSP7/MAPK11/MEF2C/RPS6KA5	5
168643	Nucleotide-binding domain; leucine rich repeat containing receptor (NLR) signaling pathways	8/492	44/6750	0.012929	0.253079	0.241562	AIM2/CARD9/IRAK2/MEFV/NLRC4/NLRP3/NOD1/PANX1	8
114604	GPVI-mediated activation cascade	6/492	28/6750	0.013788	0.263325	0.251342	COL1A2/PIK3CG/PIK3R6/RAC2/RHOB/VAV3	6
373076	Class A/1 (Rhodopsin-like receptors)	33/492	307/6750	0.014822	0.27633	0.263755	ACKR3/ADRB2/C3/C3AR1/CCL17/CCL19/CCL22/CCL3/CCL4/CCL5/CCR4/CCR5/CCR6/CCR7/CXCL10/CXCL11/CXCL12/CXCL13/CXCL2/CXCL9/EDN1/FFAR2/FFAR3/GPR132/GPR68/HCAR2/HRH2/HTR7/P2RY2/PTGIR/SSTR2/SUCNR1/TBXA2R	33
399955	SEMA3A-Plexin repulsion signaling by inhibiting Integrin adhesion	4/492	14/6750	0.015499	0.282221	0.269378	NRP1/PLXNA1/PLXNA2/RND1	4

216083	Integrin cell surface interactions	12/492	83/6750	0.016439	0.290922	0.277683	COL1A2/COL6A1/COL6A2/COL8A2/DAG1/IBSP/ITGA11 /ITGA2/ITGB7/ITGB8/LUM/VTN	12
166054	Activated TLR4 signalling	13/492	93/6750	0.01672	0.290922	0.277683	CD14/DUSP4/DUSP7/IRAK2/MAPK11/MEF2C/NFKB2/ NFKBIA/NOD1/PELI1/RELA/RPS6KA5/TLR2	13
445989	TAK1 activates NFkB by phosphorylation and activation of IKKs complex	5/492	22/6750	0.01881	0.320172	0.305602	IRAK2/NFKB2/NFKBIA/NOD1/RELA	5
376176	Signaling by Robo receptor	5/492	23/6750	0.022635	0.357282	0.341023	ENAH/EVL/ROBO3/SLIT2/SRGAP1	5

937061	TRIF-mediated TLR3/TLR4 signaling	11/492	77/6750	0.022925	0.357282	0.341023	CD14/DUSP4/DUSP7/IRAK2/MAPK11/MEF2C/NFKB2/NFKBIA/NOD1/RELA/RPS6KA5	11
1280215	Cytokine Signaling in Immune system	30/492	284/6750	0.024334	0.357282	0.341023	EGR1/FCGR1A/FCGR1B/GAB2/HERC5/HLA-DRA/IFI27/IFIT2/IFITM1/IL1RN/IL2RA/IL6/IL6R/IRAK2/IRF4/IRF8/ISG15/JAK3/MT2A/NEDD4/NOD1/NUP107/NUP188/OASL/PELI1/PRLR/PTK2B/PTPN2/SOCS2/SOCS3	30
166166	MyD88-independent TLR3/TLR4 cascade	11/492	78/6750	0.025007	0.357282	0.341023	CD14/DUSP4/DUSP7/IRAK2/MAPK11/MEF2C/NFKB2/NFKBIA/NOD1/RELA/RPS6KA5	11
168164	Toll Like Receptor 3 (TLR3) Cascade	11/492	78/6750	0.025007	0.357282	0.341023	CD14/DUSP4/DUSP7/IRAK2/MAPK11/MEF2C/NFKB2/NFKBIA/NOD1/RELA/RPS6KA5	11
399954	Sema3A PAK dependent Axon repulsion	4/492	16/6750	0.025096	0.357282	0.341023	LIMK1/NRP1/PLXNA1/PLXNA2	4
399956	CRMPs in Sema3A signaling	4/492	16/6750	0.025096	0.357282	0.341023	DPYSL3/NRP1/PLXNA1/PLXNA2	4
5627117	RHO GTPases Activate ROCKs	4/492	16/6750	0.025096	0.357282	0.341023	LIMK1/MYH10/MYH11/RHOB	4
933542	TRAF6 mediated NF-kB activation	4/492	16/6750	0.025096	0.357282	0.341023	DDX58/NFKB2/NFKBIA/RELA	4
5260271	Diseases of Immune System	5/492	24/6750	0.026931	0.369947	0.353112	CD14/NFKB2/NFKBIA/RELA/TLR2	5
5602358	Diseases associated with theTLR signaling cascade	5/492	24/6750	0.026931	0.369947	0.353112	CD14/NFKB2/NFKBIA/RELA/TLR2	5

140534	Ligand-dependent caspase activation	4/492	17/6750	0.030979	0.400501	0.382276	CD14/FAS/TNFSF10/TRADD	4
1059683	Interleukin-6 signaling	3/492	Oct-50	0.03139	0.400501	0.382276	IL6/IL6R/SOCS3	3
418359	Reduction of cytosolic Ca++ levels	3/492	Oct-50	0.03139	0.400501	0.382276	ATP2A3/ATP2B4/SLC8A3	3
69416	Dimerization of procaspase-8	3/492	Oct-50	0.03139	0.400501	0.382276	FAS/TNFSF10/TRADD	3

210991	Basigin interactions	5/492	25/6750	0.031713	0.400501	0.382276	ATP1B1/ATP1B2/MMP1/SLC7A6/SLC7A8	5
2029482	Regulation of actin dynamics for phagocyticcup formation	8/492	52/6750	0.033054	0.410811	0.392117	BAIAP2/ELMO2/FCGR1A/FCGR2A /FCGR3A/LIMK1/MYO10/VAV3	8
450282	MAPK targets/ Nuclear events mediated by MAP kinases	5/492	26/6750	0.036993	0.445098	0.424844	DUSP4/DUSP7/MAPK11/MEF2C/RPS6KA5	5
375276	Peptide ligand-binding receptors	21/492	191/6750	0.037306	0.445098	0.424844	ACKR3/C3/C3AR1/CCL17/CCL19/CCL22/CCL3/CCL4/ CCL5/CCR4/CCR5/CCR6/CCR7/CXCL10/CXCL11/CXC L12/CXCL13/CXCL2/CXCL9/EDN1/SSTR2	21
194840	Rho GTPase cycle	15/492	125/6750	0.037518	0.445098	0.424844	ARHGAP22/ARHGAP23/DLC1/ITSN1/NET1/OPHN1/ PLEKHG2/RAC2/RHOB /RHOH/SRGAP1/STARD13/TAGAP/TRIP10/VAV3	15
1810476	RIP-mediated NFkB activation via ZBP1	3/492	Nov-50	0.040878	0.463876	0.442767	NFKB2/NFKBIA/RELA	3
3371378	Regulation by c-FLIP	3/492	Nov-50	0.040878	0.463876	0.442767	FAS/TNFSF10/TRADD	3
5218900	CASP8 activity is inhibited	3/492	Nov-50	0.040878	0.463876	0.442767	FAS/TNFSF10/TRADD	3
1630316	Glycosaminoglycan metabolism	14/492	116/6750	0.041749	0.466996	0.445744	B3GNT7/B4GALT5/CHST12/DSE/GPC4/HAS3/HPSE/ HS3ST3A1/HS3ST3B1/LOC101927181/LUM/NDST2/ PAPSS2/VCAN	14
1592389	Activation of Matrix Metalloproteinases	5/492	27/6750	0.042781	0.471801	0.450331	CTSK/FURIN/MMP1/MMP2/MMP8	5

416700	Other semaphorin interactions	4/492	19/6750	0.044979	0.482447	0.460492	PLXNA1/PLXNA2/PLXNC1/SEMA4A	4
499943	Synthesis and interconversion ofnucleotide di- and triphosphates	4/492	19/6750	0.044979	0.482447	0.460492	AK1/CTPS2/GSR/TXNRD1	4
