# Supplementary material for: Distinct molecular phenotypes involving several human diseases are induced by IFN-λ3 and IFN-λ4 in monocyte-derived macrophages
Source: Genes Immun. 2022 Feb 3;23(2):73–84. doi: 10.1038/s41435-022-00164-w (PMC9042695; doi:10.1038/s41435-022-00164-w)
Supplement: Supplementary file 4 — Supplemental Data 2 [file 41435_2022_164_MOESM4_ESM.pdf]

Supplementary data2

IFN-L3 vs NT M2-MDM KEGG Enrichment

PATH_ID	Description	GeneRatio	BgRatio	pvalue	p.adjust	qvalue	geneID	Count
hsa05134	Legionellosis	24/888	57/8101	1.4494709875631E-09	4.69628599970443E-07	3.35666965540927E-07	NLRC4/CD14/TLR4/HSPA2/ITGAM/NFKB2/HSPD1/CYCS/HSPA6/CASP1/CASP3/TLR5/ITGB2/EEF1A1/NFKBIA/CASP9/CR1/CASP7/NAIP/CXCL1/TNF/CR1L/BNIP3/HSPA8	24
hsa05152	Tuberculosis	46/888	180/8101	2.1495475542443E-08	3.48226703787565E-06	2.48894979965122E-06	CLEC7A/MRC1/LSP1/CALML4/CD14/MAPK13/TLR4/ATP6V0D2/TLR6/ITGAM/FCGR3A/CALM3/HSPD1/ITGAX/CAMK2A/CYCS/MAPK3/FCGR2B/CD209/SPHK1/ATP6V0A1/ATP6V0A2/CORO1A/CYP27B1/TRAF6/SYK/CAMK2G/CASP3/TGFB1/ITGB2/HLA-DMB/VDR/FCGR3B/PPP3CA/CASP9/CR1/SPHK2/BAD/PPP3CC/TNF/IL1A/CR1L/IL10/HLA-DMA/LBP/RIPK2	46
hsa04062	Chemokine signaling pathway	45/888	192/8101	4.6675931875667 6E-07	0.000050410006425721	0.0000360305439040241	PPBP/CXCL5/CCL19/CCL7/CXCL16/CXCL9/ARRB2/PIK3CD/CCL13/VAV2/CCR7/PTK2/PLCB2/CXCR4/PIK3R6/ARRB1/MAPK3/CCL22/PIK3CG/RAC2/CXCL13/CXCL10/WAS/CCL23/PIK3R2/CXCL11/GNAQ/NFKBIA/GRK6/CCL18/PRKCB/VAV1/BAD/GNGT2/GNB5/CCL4/CXCL1/PLCG1/PARD3/CCL4L1/CCL4L2/CCL3/CCL8/PRKACB/CXCL6	45

hsa05145	Toxoplasmosis	29/888	112/8101	6.6687727968886 5E-06	0.000529609042081074	0.000378537976796089	BIRC3/MAPK13/TLR4/HSPA2/ALOX5/LDLR/PIK3R6/CYCS/MAPK3/PIK3CG/HSPA6/TRAF6/CASP3/TGFB1/LY96/HLA-DMB/NFKBIA/CASP9/BAD/CD40/LAMB3/TNF/LAMB1/IL10/HSPA8/HLA-DMA/LAMA3/SOCS1/LAMC1	29
hsa05146	Amoebiasis	27/888	102/8101	0.0000088622952675647	0.000529609042081074	0.000378537976796089	CD14/TLR4/PIK3CD/PTK2/PLCB2/IL1R1/ITGAM/IL1R2/GNA11/CASP3/TGFB1/PIK3R2/ITGB2/FN1/GNAQ/PRKCB/CXCL1/LAMB3/TNF/PRDX1/LAMB1/IL10/PRKCA/SERPINB9/LAMA3/LAMC1/PRKACB	27
hsa05133	Pertussis	22/888	76/8101	0.0000128833787310869	0.000529609042081074	0.000378537976796089	CXCL5/FOS/CALML4/CD14/MAPK13/TLR4/ITGAM/CALM3/MAPK3/TRAF6/CASP1/CASP3/ITGB2/LY96/IRF1/CASP7/NOD1/TNF/C1QA/IL1A/IL10/CXCL6	22
hsa04064	NF-kappa B signaling pathway	27/888	104/8101	0.0000130767664711376	0.000529609042081074	0.000378537976796089	CCL19/BIRC3/CD14/TLR4/TRAF5/CCL13/IL1R1/NFKB2/TRAF1/GADD45B/TRAF6/SYK/LY96/RELB/NFKBIA/PRKCB/CD40/TNFAIP3/CCL4/CXCL1/TNF/PLCG1/CCL4L1/CCL4L2/LBP/EDARADD/GADD45G	27
hsa04620	Toll-like receptor signaling pathway	27/888	104/8101	0.0000130767664711376	0.000529609042081074	0.000378537976796089	FOS/CD14/CXCL9/MAPK13/TLR4/PIK3CD/TLR6/SPP1/MAPK3/TRAF6/TLR7/CXCL10/PIK3R2/TLR5/LY96/CXCL11/NFKBIA/CD86/CD80/CD40/CCL4/TNF/CTSK/CCL4L1/CCL4L2/LBP/CCL3	27
hsa05140	Leishmaniasis	22/888	77/8101	0.0000162039315245727	0.000583341534884617	0.000416943267298946	FOS/MAPK13/TLR4/PTPN6/ITGAM/FCGR3A/NCF2/MAPK3/TRAF6/TGFB1/ITGB2/EEF1A1/HLA-	22

							DMB/NFKBIA/FCGR3B/PRKCB/CR1/TNF/IL1A/CR1L/IL10/HLA-DMA	
hsa04061	Viral protein interaction with cytokine and cytokine receptor	25/888	100/8101	0.0000538449712370402	0.00173425323394924	0.00123955721724767	PPBP/CXCL5/CCL19/CCL7/CSF1R/CXCL9/CCL13/CCR7/CXCR4/CCL22/CXCL13/CXCL10/CCL23/CXCL11/CCL18/CCL4/CXCL1/TNF/IL10/TNFRSF1B/CCL4L1/CCL4L2/CCL3/CCL8/CXCL6	25
hsa05132	Salmonella infection	48/888	249/8101	0.0000588789678192644	0.00173425323394924	0.00123955721724767	RALA/FOS/AHNAK2/NLRC4/BIRC3/CD14/MAPK13/TLR4/STX10/PIK3CD/RRAS/HSP90AB1/TLR6/PLEKHM1/TCF7L2/HSP90AA1/CYCS/MAPK3/AHNAK/PIK3CG/TUBB/GSDMD/TUBA1C/MYC/KLC4/DYNC1LI2/TRAF6/TUBA1B/MYL9/CASP1/CASP3/TLR5/LY96/NFKBIA/DYNC2H1/TUBB4B/S100A10/CASP7/NAIP/NOD1/CYTH3/TXN/FLNA/TNF/CASP5/CYFIP2/DYNC2LI1/RIPK2	48
hsa05222	Small cell lung cancer	23/888	92/8101	0.000106023043492665	0.00286262217430194	0.00204605873406897	RXRA/BIRC3/TRAF5/PIK3CD/PTK2/TRAF1/CYCS/MYC/E2F2/CDKN1A/GADD45B/TRAF6/CASP3/PIK3R2/FN1/NFKBIA/CASP9/CDKN2B/LAMB3/LAMB1/LAMA3/LAMC1/GADD45G	23
hsa04928	Parathyroid hormone synthesis; secretion and action	25/888	106/8101	0.000148671013896829	0.00370533911558251	0.00264839053095566	RXRA/FOS/MEF2D/ARRB2/PDE4A/CREB5/KL/PLCB2/AKAP13/ARRB1/MAPK3/SLC9A3R1/CDKN1A/CYP27B1/GNA11/GNAQ/VDR/PRKCB/RUNX2/HBEGF/MMP25/PRKCA/PDE4B/CREB3L4/PRKACB	25
hsa05205	Proteoglycans in cancer	40/888	205/8101	0.000183285666491162	0.0037131219051851	0.00265395327855985	ITGB5/MAPK13/TLR4/VTN/ITGB3/PIK3CD/RRAS/PTPN6/VAV2/PTK2/PPP1CB/CAMK2A/SDC4/MAPK3/M	40

							ET/MMP9/SDC2/MYC/TIMP3/CDKN1A/RPS6/CAMK2G/PDCD4/CASP3/TGFB1/PIK3R2/FN1/PRKCB/VAV1/HBEGF/FLNA/TNF/PLCG1/NUDT16L1/LUM/PLAUR/PRKCA/WNT5A/MMP2/PRKACB	
hsa05418	Fluid shear stress and atherosclerosis	30/888	139/8101	0.000189339454894348	0.0037131219051851	0.00265395327855985	TRPV4/EDN1/FOS/MGST1/CALML4/MAPK13/ITGB3/PIK3CD/PTK2/IL1R1/HSP90AB1/CALM3/HSP90AA1/NCF2/SDC4/MMP9/IL1R2/SDC2/PECAM1/RAC2/MAP3K5/PIK3R2/DUSP1/GSTM4/KLF2/TXN/THBD/TNF/IL1A/MMP2	30
hsa04145	Phagosome	32/888	152/8101	0.000190813666004411	0.0037131219051851	0.00265395327855985	CLEC7A/MARCO/MRC1/ITGB5/CD14/COLEC12/TLR4/ITGB3/ATP6V0D2/OLR1/TLR6/ITGAM/FCGR3A/FCAR/NCF2/FCGR2B/CD209/ATP6V0A1/TUBB/CD36/ATP6V0A2/CORO1A/TUBA1C/DYNC1LI2/ATP6V0E2/TUBA1B/ITGB2/HLA-DMB/DYNC2H1/FCGR3B/TUBB4B/HLA-DMA	32
hsa05202	Transcriptional misregulation in cancer	38/888	192/8101	0.00019482429749428	0.0037131219051851	0.00265395327855985	TGFBR2/FLT1/RXRA/CSF1R/BIRC3/CD14/HHEX/NR4A3/RARA/DUSP6/PTK2/PBX3/ITGAM/TRAF1/MAF/LMO2/LYL1/FLI1/MET/MMP9/IL1R2/ETV5/MYC/CDKN1A/GADD45B/DOT1L/ITGB7/ID2/CD86/CD40/RUNX2/CCNA2/H3F3A/CDKN2C/SUPT3H/ETV7/CCNA1/GADD45G	38
hsa04380	Osteoclast differentiation	28/888	128/8101	0.000241945791672149	0.00435502425009869	0.0031127528753142	TGFBR2/FOS/CSF1R/MAPK13/ITGB3/PIK3CD/IL1R1/NFKB2/FCGR3A/NCF2/MAPK3/FCGR2B/NFATC1/LILRA1/TRAF6/SYK/TGFB1/PIK3R2/REL	28

							B/NFKBIA/FCGR3B/PPP3CA/PPP3CC/TNF/IL1A/CTSK/SOCS3/SOCS1	
hsa00900	Terpenoid backbone biosynthesis	9/888	22/8101	0.000290327867292141	0.0049508541580344	0.00353862220522277	HMGCS1/HMGCR/MVD/IDI1/FDPS/ACAT2/PCYOX1/RCE1/MVK	9
hsa05323	Rheumatoid arthritis	22/888	93/8101	0.000348515144839156	0.00564594534639433	0.00403543851919023	CXCL5/FLT1/FOS/TLR4/TNFSF13/ATP6V0D2/ITGAL/ATP6V0A1/ATP6V0A2/ATP6V0E2/TGFB1/ITGB2/HLA-DMB/CD86/CD80/CXCL1/TNF/IL1A/CTSK/HLA-DMA/CCL3/CXCL6	22
hsa04668	TNF signaling pathway	25/888	112/8101	0.000370349487538425	0.00571396352202141	0.0040840544991706	CXCL5/EDN1/FOS/BIRC3/MAPK13/TRAF5/PIK3CD/CREB5/TRAF1/MAPK3/MMP9/MAP3K5/CXCL10/CASP3/PIK3R2/NFKBIA/IRF1/TNFAIP3/CASP7/CXCL1/TNF/SOCS3/TNFRSF1B/CREB3L4/CXCL6	25
hsa04370	VEGF signaling pathway	16/888	59/8101	0.000438746497969484	0.00646153933373241	0.00461838418915247	MAPK13/PIK3CD/PTK2/PLA2G4A/MAPK3/SPHK1/RAC2/PIK3R2/PPP3CA/CASP9/PRKCB/SPHK2/BAD/PPP3CC/PLCG1/PRKCA	16
hsa05235	PD-L1 expression and PD-1 checkpoint pathway in cancer	21/888	89/8101	0.000488971871171283	0.00688812548954329	0.0049232865747223	FOS/MAPK13/TLR4/PIK3CD/PTPN6/CD4/MAPK3/CD274/NFATC1/MAP3K3/TRAF6/RASGRP1/PIK3R2/NFKBIA/PPP3CA/ALK/BATF3/PPP3CC/PLCG1/NFKBIE/BATF	21
hsa04010	MAPK signaling pathway	51/888	294/8101	0.000535319571117646	0.00722681421008822	0.00516536428271413	TGFBR2/FLT1/FOS/CSF1R/CD14/MAPK13/ARRB2/HSPA2/INSR/RRAS/DUSP6/DUSP2/CDC25B/IL1R1/NFKB2/TGFA/PLA2G4A/RASGRP4/ARRB1/MAPK3/NFATC1/MET/HSPA6/MYC/MAP3K3/RAC2/GADD45B/TRAF6/PDGFC/MAP3K5/RASGRP1/R	51

							APGEF2/DUSP5/MAP3K1/CASP3/TGFB1/RELB/PPP3CA/PRKCB/CACNA2D4/DUSP1/PPP3CC/MAP3K13/FLNA/TNF/IL1A/MAP3K12/HSPA8/PRKCA/PRKACB/GADD45G	
hsa05144	Malaria	14/888	50/8101	0.000695865512920314	0.00901841704744727	0.00644591211968291	TLR4/LRP1/ITGAL/MET/SDC2/CD36/PECAM1/TGFB1/ITGB2/CR1/CD40/TNF/CR1L/IL10	14
hsa05210	Colorectal cancer	20/888	86/8101	0.000804366786832752	0.00999817027706071	0.00714619058139492	TGFBR2/RALA/FOS/PIK3CD/TCF7L2/TGFA/CYCS/MAPK3/MYC/RAC2/CDKN1A/GADD45B/CASP3/TGFB1/PIK3R2/CASP9/BAD/BCL2L11/PMAIP1/GADD45G	20
hsa04068	FoxO signaling pathway	27/888	131/8101	0.000833180856421726	0.00999817027706071	0.00714619058139492	TGFBR2/MAPK13/INSR/PIK3CD/SGK1/FOXO4/FBXO32/IRS2/PRMT1/SGK3/MAPK3/GABARAPL1/PCK2/CDKN1A/GADD45B/TGFB1/PIK3R2/CDKN2B/BCL2L11/KLF2/HOMER3/CAT/BNIP3/S1PR4/IL10/CDKN2D/GADD45G	27
hsa04060	Cytokine-cytokine receptor interaction	50/888	295/8101	0.00103462734569163	0.0117364018156128	0.00838859129121121	PPBP/EBI3/TGFBR2/CXCL5/CCL19/CCL7/CXCL16/CSF1R/PRLR/CXCL9/CCL13/TNFSF12/TNFSF13/CCR7/IL1R1/CD4/CXCR4/IL16/CCL22/TNFSF8/TNFRSF8/IL1R2/IL15RA/BMP2/CXCL13/CXCL10/CCL23/TGFB1/CXCL11/CCL18/CD40/CCL4/CXCL1/TNF/TNFSF15/IL1A/IL17RA/CSF2RA/IL27/RELT/IL10/TNFRSF9/TNFRSF1B/CCL4L1/EPOR/LIFR/CCL4L2/CCL3/CCL8/CXCL6	50
hsa04657	IL-17 signaling pathway	21/888	94/8101	0.00105048040942213	0.0117364018156128	0.00838859129121121	CXCL5/CCL7/FOS/MAPK13/TRAF5/HSP90AB1/HSP90AA1/MAPK3/S100A9/MMP9/TRAF6/CXCL10/CASP3	21

							/S100A8/NFKBIA/TNFAIP3/CXCL1/TNF/IL17RA/MAPK6/CXCL6	
hsa04662	B cell receptor signaling pathway	19/888	82/8101	0.0011288228569 6106	0.0121789319 241715	0.008704889614 4176	CD72/FOS/PIK3CD/PTPN6/VAV2/MAPK3/FCGR2B/NFATC1/LILRA1/RAC2/SYK/PIK3R2/NFKBIA/PPP3CA/PRKCB/VAV1/PPP3CC/NFKBIE/CD22	19
hsa04670	Leukocyte transendothelial migration	24/888	114/8101	0.0011652681779 2999	0.0121789319 241715	0.008704889614 4176	MAPK13/VASP/PIK3CD/VAV2/PTK2/ITGAM/CLDN7/CXCR4/NCF2/ITGAL/MMP9/PECAM1/RAC2/MYL9/TXK/PIK3R2/ITGB2/RASSF5/PRKCB/CLDN23/VAV1/PLCG1/PRKCA/MMP2	24
hsa05163	Human cytomegalovirus infection	40/888	225/8101	0.0012841477383 3652	0.0130019958 506573	0.009293174422 17218	CALML4/MAPK13/TRAF5/ITGB3/PIK3CD/CREB5/PTK2/PLCB2/IL1R1/AKAP13/CALM3/CXCR4/CYCS/MAPK3/NFATC1/MYC/RAC2/E2F2/CDKN1A/GNA11/CASP3/PIK3R2/GNAQ/NFKBIA/EIF4EBP1/PPP3CA/CASP9/PRKCB/MB21D1/PPP3CC/GNGT2/GNB5/CCL4/TNF/PRKCA/CCL4L1/CCL4L2/CREB3L4/CCL3/PRKACB	40
hsa04613	Neutrophil extracellular trap formation	35/888	190/8101	0.0013407058858 4649	0.0131632941 519473	0.009408462356 81744	CLEC7A/SELPLG/MAPK13/HDAC5/TLR4/H2AFY2/ITGB3/PIK3CD/C5AR1/PLCB2/ITGAM/FCGR3A/NCF2/MAPK3/ITGAL/H2AFY/GSDMD/RAC2/SYK/TLR7/CASP1/PIK3R2/FPR3/ITGB2/HIST1H2BK/FCGR3B/PRKCB/FPR2/CR1/FPR1/SIGLEC9/PLCG1/H3F3A/CR1L/PRKCA	35
hsa05166	hsa05166	39/888	219/8101	0.0014211053912 9453	0.0133285538 782763	0.009526581719 3658	TGFBR2/FOS/FDPS/SLC2A1/PIK3CD/CREB5/IL1R1/CD4/NFKB2/NRP1/MAPK3/NFATC1/ITGAL/IL1R2/MYC/MAP3K3/E2F2/IL15RA/CDKN1A/	39

							ZFP36/MAP3K1/TGFB1/PIK3R2/ITGB2/HLA-DMB/RELB/NFKBIA/PPP3CA/CDKN2B/CD40/PPP3CC/MAD1L1/TNF/CCNA2/CDKN2C/HLA-DMA/CCNA1/CREB3L4/PRKACB	
hsa04218	Cellular senescence	30/888	156/8101	0.0014398129189496	0.0133285538782763	0.0095265817193658	TGFBR2/TRPV4/CALML4/MAPK13/PIK3CD/RRAS/PPP1CB/ZFP36L1/CALM3/MAPK3/NFATC1/HIPK2/MYC/E2F2/ZFP36L2/CDKN1A/GADD45B/TGFB1/PIK3R2/RASSF5/EIF4EBP1/PPP3CA/CDKN2B/PPP3CC/IL1A/CCNA2/FOXM1/CCNA1/SERPINE1/GADD45G	30
hsa04666	Fc gamma R-mediated phagocytosis	21/888	97/8101	0.00160267986061399	0.0142159066834254	0.0101608169927017	VASP/PIK3CD/VAV2/FCGR3A/PLA2G4A/MAPK3/FCGR2B/SPHK1/RAC2/SYK/WAS/PIP5K1C/PIK3R2/FCGR3B/PRKCB/SPHK2/VAV1/MYO10/ASAP2/PLCG1/PRKCA	21
hsa05223	Non-small cell lung cancer	17/888	72/8101	0.00162342144224303	0.0142159066834254	0.0101608169927017	RXRA/PIK3CD/TGFA/MAPK3/MET/E2F2/CDKN1A/GADD45B/PIK3R2/RASSF5/CASP9/PRKCB/ALK/BAD/PLCG1/PRKCA/GADD45G	17
hsa04625	C-type lectin receptor signaling pathway	22/888	104/8101	0.0017222851779269	0.0146847473065346	0.0104959207519091	CLEC7A/LSP1/CALML4/MAPK13/PIK3CD/RRAS/NFKB2/CALM3/MAPK3/CCL22/NFATC1/CD209/SYK/CASP1/PIK3R2/RELB/NFKBIA/PPP3CA/IRF1/PPP3CC/TNF/IL10	22
hsa04750	Inflammatory mediator regulation of TRP channels	21/888	98/8101	0.00183465779264711	0.015207227111858	0.0108693631078907	TRPV4/CALML4/MAPK13/PIK3CD/PLCB2/IL1R1/PPP1CB/CALM3/PLA2G4A/CAMK2A/ASIC1/CAMK2G/PIK3R2/GNAQ/PRKCB/PLCG1/PRKCA/TRPV1/P2RY2/PRKACB/HTR2B	21

hsa01524	Platinum drug resistance	17/888	73/8101	0.00190533616386471	0.015207227111858	0.0108693631078907	MGST1/BIRC3/PIK3CD/SLC31A1/CYCS/MAPK3/CDKN1A/MAP3K5/CASP3/PIK3R2/CASP9/BAD/GSTM4/REV3L/BRCA1/TOP2A/PMAIP1	17
hsa04510	Focal adhesion	36/888	201/8101	0.0019243713320561	0.015207227111858	0.0108693631078907	FLT1/ITGB5/BIRC3/VASP/VTN/ITGB3/PIK3CD/VAV2/PTK2/PPP1CB/SPP1/MAPK3/MET/RAC2/PDGFC/MYL9/ITGB7/PIP5K1C/ZYX/PIK3R2/FN1/ITGA1/ITGA9/PRKCB/COL6A2/VAV1/BAD/TNC/LAMB3/FLNA/COL6A1/MYLK/LAMB1/PRKCA/LAMA3/LAMC1	36
hsa04152	AMPK signaling pathway	24/888	120/8101	0.0024232055720679	0.0186933001273809	0.013361033229447	HMGCR/INSR/PIK3CD/ADIPOR1/CREB5/PFKFB4/FASN/IRS2/ULK1/AKT1S1/CD36/PFKP/PCK2/PIK3R2/EIF4EBP1/ACACA/PFKFB2/FBP1/ACACB/SCD/CCNA2/CCNA1/CREB3L4/PPP2R1B	24
hsa05031	Amphetamine addiction	16/888	69/8101	0.00268207704402451	0.0202091386573009	0.0144444785724698	FOS/CALML4/MAOA/CREB5/PPP1CB/CALM3/CAMK2A/CAMK2G/GRIN3A/STX1A/PPP3CA/PRKCB/PPP3CC/PRKCA/CREB3L4/PRKACB	16
hsa04151	PI3K-Akt signaling pathway	56/888	354/8101	0.00275127498598685	0.0202593885331759	0.0144803946630887	FLT1/RXRA/CSF1R/ITGB5/PRLR/TLR4/VTN/INSR/ITGB3/PIK3CD/CREB5/SGK1/PTK2/HSP90AB1/SPP1/TGFA/PIK3R6/HSP90AA1/SGK3/MAPK3/PIK3CG/MET/PCK2/MYC/CDKN1A/RPS6/PDGFC/SYK/ITGB7/PIK3R2/FN1/ITGA1/EIF4EBP1/ITGA9/CASP9/PKN3/COL6A2/BAD/BCL2L11/GNGT2/GNB5/TNC/LAMB3/PHLPP1/COL6A1/LPAR2/PHLPP2/BRCA1/LAMB1/LPAR6/PRKCA/EPOR/LAMA3/LAMC1/CREB3L4/PPP2R1B	56

hsa04514	Cell adhesion molecules	28/888	149/8101	0.0029009969787 2268	0.0208871782 468033	0.014929107258 9237	C10orf54/SELPLG/CD276/NRCAM/CD4/PVRL1/ITGAM/CLDN7/SDC4/CD274/ITGAL/SDC2/PECAM1/PVR/VCAN/ITGB7/ITGB2/HLA-DMB/PTPRM/ITGA9/CD86/CD80/CLDN23/PVRL3/CD40/SDC3/HLA-DMA/CD22	28
hsa00534	Glycosaminoglycan biosynthesis - heparan sulfate / heparin	8/888	24/8101	0.00298178257747521	0.0210021207 630862	0.015011262403 7653	NDST1/EXT1/EXTL2/GLCE/HS3ST3B1/XYLT2/HS2ST1/HS3ST1	8
hsa05142	Chagas disease	21/888	102/8101	0.003068421836217	0.0211525249 986023	0.015118763806 6683	ACE/TGFBR2/FOS/MAPK13/TLR4/PIK3CD/PLCB2/TLR6/MAPK3/TRAF6/GNA11/TGFB1/PIK3R2/GNAQ/NFKBIA/TNF/C1QA/IL10/SERPINE1/PPP2R1B/CCL3	21
hsa04210	Apoptosis	26/888	136/8101	0.0031576264596338	0.0211876203 39336	0.015143848195 7567	FOS/BIRC3/LMNB2/PIK3CD/TRAF1/CYCS/MAPK3/SPTAN1/TUBA1C/GADD45B/TUBA1B/MAP3K5/CASP3/LMNB1/PIK3R2/NFKBIA/CASP9/BAD/BCL2L11/CASP7/CTSC/TNF/CTSK/SEPT4/PMAIP1/GADD45G	26
hsa04066	HIF-1 signaling pathway	22/888	109/8101	0.00320430060687489	0.0211876203 39336	0.015143848195 7567	FLT1/EDN1/SLC2A1/HK3/TLR4/INSR/PIK3CD/CAMK2A/MAPK3/PFKP/CDKN1A/RPS6/CAMK2G/ENO2/PIK3R2/EGLN1/PDK1/EIF4EBP1/PRKCB/PLCG1/PRKCA/SERPINE1	22
hsa00100	Steroid biosynthesis	7/888	20/8101	0.0039836708795335	0.0258141872 993771	0.018450686178 892	FDFT1/MSMO1/SQLE/CYP51A1/CYP27B1/HSD17B7/EBP	7

hsa04015	Rap1 signaling pathway	36/888	210/8101	0.0041164674869 7553	0.0261516757 996092	0.018691906029 6102	FLT1/RALA/CSF1R/CALML4/MAPK13/VASP/INSR/ITGB3/PIK3CD/RRAS/VAV2/APBB1IP/PLCB2/ITGAM/CALM3/RAPGEF5/MAPK3/ITGAL/MET/RAC2/PDGFC/RAPGEF2/PIK3R2/ITGB2/RASSF5/GNAQ/PRKCB/FPR1/VAV1/EVL/LPAR2/PLCG1/ADORA2A/PRKCA/PARD3/RGS14	36
hsa04610	Complement and coagulation cascades	18/888	85/8101	0.0043107695588 4378	0.0268594103 281805	0.019197759168 9399	VTN/C5AR1/VSIG4/ITGAM/F13A1/ITGAX/PROS1/ITGB2/CR1/F8/THBD/C1QA/CR1L/TFPI/PLAUR/CFB/F5/SERPINE1	18
hsa05130	Pathogenic Escherichia coli infection	34/888	197/8101	0.0046767794564 834	0.0285901234 698231	0.020434786105 7865	FOS/MAPK13/TLR4/PTPN6/IL1R1/MYO1F/CLDN7/CYCS/MAPK3/SLC9A3R1/WIPF1/MYH11/TUBB/TUBA1C/MYO1E/TJP1/TRAF6/TUBA1B/CASP1/CASP3/TLR5/NFKBIA/CASP9/MYO1A/CLDN23/TUBB4B/MYO1G/MYO10/CASP7/NAIP/CYTH3/LPAR2/TNF/CYFIP2	34
hsa04640	Hematopoietic cell lineage	20/888	99/8101	0.0047716294179 915	0.0286297765 07949	0.020463128108 3456	CSF1R/CD37/CD14/ITGB3/IL1R1/CD4/CD9/ITGAM/IL1R2/CD36/HLA-DMB/ITGA1/CR1/TNF/IL1A/CSF2RA/CR1L/HLA-DMA/EPOR/CD22	20
hsa05221	Acute myeloid leukemia	15/888	67/8101	0.0051196382663 2672	0.0301593236 052701	0.021556371647 6915	CSF1R/CD14/PIK3CD/RARA/DUSP6/TCF7L2/ITGAM/PPARD/MAPK3/MYC/PIK3R2/EIF4EBP1/BAD/CCNA2/CCNA1	15
hsa04971	Gastric acid secretion	16/888	76/8101	0.0073196724302 2522	0.0423495333 463031	0.030269322079 8787	KCNJ2/CALML4/KCNQ1/PLCB2/CALM3/CAMK2A/SSTR2/CAMK2G/GNAQ/KCNJ10/PRKCB/KCNJ15/CA2/MYLK/PRKCA/PRKACB	16

hsa03008	Ribosome biogenesis in eukaryotes	21/888	110/8101	0.0076505704002 9737	0.0434874528 016903	0.031082649825 7695	GTPBP4/IMP4/NAT10/MPHOSPH10/NOP56/NOB1/DKC1/CIRH1A/GNL2/NOP58/RCL1/NOL6/NHP2/RBM28/RIOK1/HEATR1/UTP14A/GNL3/UTP15/RPP40/SNORD3A	21
hsa04360	Axon guidance	31/888	182/8101	0.0081511748014 0518	0.0452823368 599563	0.032365542914 8485	FES/PIK3CD/RRAS/PTK2/CXCR4/SSH2/CAMK2A/NRP1/MAPK3/NTN1/MET/SEMA6B/RAC2/PLXNB2/SRGAP1/MYL9/CAMK2G/PIK3R2/PDK1/PPP3CA/EPHB6/SEMA4D/SLIT2/PPP3CC/PLCG1/PRKCA/PARD3/WNT5A/SEMA4B/EPHA7/ABLIM3	31
hsa05215	Prostate cancer	19/888	97/8101	0.0082458576380 7846	0.0452823368 599563	0.032365542914 8485	PIK3CD/CREB5/HSP90AB1/TCF7L2/TGFA/HSP90AA1/MAPK3/MMP9/IL1R2/ETV5/E2F2/CDKN1A/PDGFC/PIK3R2/NFKBIA/NKX3-1/CASP9/BAD/CREB3L4	19
hsa04520	Adherens junction	15/888	71/8101	0.0089481453126 3226	0.0483199846 882142	0.034536701206 6509	TGFBR2/INSR/PTPN6/PVRL1/TCF7L2/MAPK3/MET/PVRL4/RAC2/TJP1/WAS/SSX2IP/PTPRM/PVRL3/PARD3	15
hsa04725	Cholinergic synapse	21/888	113/8101	0.0104018758762 95	0.0552493079 331076	0.039489433870 3174	KCNJ2/FOS/PIK3CD/CREB5/KCNQ1/PLCB2/PIK3R6/ACHE/CAMK2A/MAPK3/PIK3CG/GNA11/CAMK2G/PIK3R2/GNAQ/PRKCB/GNGT2/GNB5/PRKCA/CREB3L4/PRKACB	21
hsa04933	AGE-RAGE signaling pathway in diabetic complications	19/888	100/8101	0.0114148098451 53	0.0581196969 694627	0.041541043967 7771	TGFBR2/EDN1/MAPK13/PIK3CD/PLCB2/MAPK3/NFATC1/CASP3/TGFB1/PIK3R2/FN1/PRKCB/THBD/TNF/IL1A/PLCG1/PRKCA/SERPINE1/MMP2	19

hsa04919	Thyroid hormone signaling pathway	22/888	121/8101	0.0114191657806704	0.0581196969694627	0.0415410439677771	SLC16A10/RXRA/SLC2A1/ITGB3/PIK3CD/PLCB2/ATP2A3/MAPK3/NOTCH3/PFKP/MYC/PIK3R2/CASP9/RCAN2/PRKCB/PFKFB2/BAD/PLCG1/PRKCA/THRB/THRA/PRKACB	22
hsa04659	Th17 cell differentiation	20/888	107/8101	0.0114804339692766	0.0581196969694627	0.0415410439677771	TGFBR2/IRF4/RXRA/FOS/MAPK13/RARA/IL1R1/HSP90AB1/CD4/HSP90AA1/MAPK3/NFATC1/TGFB1/HLA-DMB/NFKBIA/PPP3CA/PPP3CC/PLCG1/NFKBIE/HLA-DMA	20
hsa05416	Viral myocarditis	13/888	60/8101	0.0116889046366422	0.0582646938811086	0.0416446804868223	CYCS/ITGAL/EIF4G3/RAC2/CASP3/ITGB2/HLA-DMB/CASP9/CD86/CD80/CD40/HLA-DMA/SGCG	13
hsa04931	Insulin resistance	20/888	108/8101	0.0126873335942716	0.0622832740082424	0.0445169599799004	PYGL/SLC2A1/INSR/PIK3CD/CREB5/SLC27A1/PPP1CB/IRS2/CD36/PCK2/PPP1R3B/SLC27A3/PIK3R2/NFKBIA/PRKCB/ACACB/TNF/SOCS3/TRIB3/CREB3L4	20
hsa04371	Apelin signaling pathway	24/888	137/8101	0.0133082833929209	0.06249106984502	0.0446654820204821	CALML4/MEF2D/HDAC5/SLC8A3/RRAS/ACTA2/PLCB2/SPP1/CALM3/PIK3R6/MAPK3/SPHK1/GABARAPL1/NOTCH3/PIK3CG/RPS6/GNAQ/SPHK2/GNGT2/GNB5/KLF2/MYLK/SERPINE1/PRKACB	24
hsa04910	Insulin signaling pathway	24/888	137/8101	0.0133082833929209	0.06249106984502	0.0446654820204821	CALML4/PYGL/HK3/INSR/PIK3CD/TRIP10/PPP1CB/FASN/IRS2/CALM3/MAPK3/PCK2/PPP1R3B/RPS6/PIK3R2/EIF4EBP1/ACACA/BAD/FBP1/ACACB/SOCS3/SOCS2/SOCS1/PRKACB	24

hsa05135	Yersinia infection	24/888	137/8101	0.0133082833929209	0.06249106984502	0.0446654820204821	FOS/NLRC4/MAPK13/TLR4/PIK3CD/VAV2/PTK2/CD4/MAPK3/NFATC1/WIPF1/RAC2/TRAF6/CASP1/WAS/PIP5K1C/PIK3R2/FN1/GNAQ/NFKBIA/VAV1/TNF/PLCG1/IL10	24
hsa04512	ECM-receptor interaction	17/888	88/8101	0.0137983277556376	0.0638665456118081	0.0456486031013573	ITGB5/VTN/ITGB3/SPP1/SDC4/CD36/ITGB7/FN1/ITGA1/ITGA9/COL6A2/TNC/LAMB3/COL6A1/LAMB1/LAMA3/LAMC1	17
hsa04664	Fc epsilon RI signaling pathway	14/888	68/8101	0.0141912560376398	0.0641641581712837	0.0458613216298974	MAPK13/ALOX5/PIK3CD/VAV2/PLA2G4A/MAPK3/RAC2/SYK/PIK3R2/ALOX5AP/VAV1/TNF/PLCG1/PRKCA	14
hsa05170	Human immunodeficiency virus 1 infection	34/888	212/8101	0.0142587018158408	0.0641641581712837	0.0458613216298974	FOS/CALML4/MAPK13/TLR4/TRAF5/PIK3CD/PTK2/CD4/CALM3/CXCR4/CYCS/MAPK3/NFATC1/RAC2/TRAF6/GNA11/CASP3/PIK3R2/GNAQ/NFKBIA/PPP3CA/CASP9/PRKCB/MB21D1/BAD/PPP3CC/GNGT2/GNB5/TNF/AP1S3/PLCG1/PRKCA/TNFRSF1B/APOBEC3G	34
hsa05214	Glioma	15/888	75/8101	0.0147567978799815	0.0654959248371783	0.0468132016380092	CALML4/PIK3CD/CALM3/TGFA/CAMK2A/MAPK3/E2F2/CDKN1A/GADD45B/CAMK2G/PIK3R2/PRKCB/PLCG1/PRKCA/GADD45G	15
hsa04672	Intestinal immune network for IgA production	11/888	49/8101	0.015110912973668	0.0661612946414651	0.0472887745975384	TNFSF13/CXCR4/IL15RA/ITGB7/TGFB1/HLA-DMB/CD86/CD80/CD40/IL10/HLA-DMA	11
hsa04728	Dopaminergic synapse	23/888	132/8101	0.0161163998692498	0.0691676440106301	0.0494375623207883	FOS/CALML4/MAPK13/ARRB2/MAOA/CREB5/PLCB2/PPP1CB/CALM3/CAMK2A/ARRB1/CAMK2G/GNAQ/PPP3CA/PRKCB/PPP3CC/GNGT2/	23

							GNB5/KCNJ5/PRKCA/CREB3L4/PPP2R1B/PRKACB	
hsa04921	Oxytocin signaling pathway	26/888	154/8101	0.0162245090889132	0.0691676440106301	0.0494375623207883	KCNJ2/FOS/CALML4/GUCY1A3/PLCB2/PPP1CB/CALM3/PLA2G4A/PIK3R6/CAMK2A/MAPK3/NFATC1/PIK3CG/CDKN1A/MYL9/CAMK2G/GNAQ/PPP3CA/PRKCB/CACNA2D4/PPP3CC/KCNJ5/MYLK/PRKCA/MYL6B/PRKACB	26
hsa05161	Hepatitis B	27/888	162/8101	0.0170045421934271				
hsa05131	Shigellosis	38/888	246/8101	0.0177281163304902	0.0736398678343437	0.0526340835723055	NLRC4/CD14/MAPK13/HK3/TLR4/TRAF5/SEPT11/PIK3CD/PTK2/PLCB2/IL1R1/FOXO4/WIPI2/AKT1S1/CYCS/MAPK3/WIPI1/GABARAPL1/SEPT9/TRAF6/MYL9/CASP1/TIFA/PIK3R2/TLR5/UBB/NFKBIA/MB21D1/TECPR1/SEPT3/NAIP/NOD1/CYTH3/TNF/PLCG1/H3F3A/BNIP3/RIPK2	38
hsa04722	Neurotrophin signaling pathway	21/888	119/8101	0.0182652999842459	0.0739744649361958	0.0528732367965013	CALML4/MAPK13/PIK3CD/CALM3/CAMK2A/MAPK3/ARHGDIB/IRAK3/SORT1/MAP3K3/TRAF6/MAP3K5/MAP3K1/CAMK2G/PIK3R2/NFKBIA/BAD/PLCG1/NFKBIE/NGFRAP1/RIPK2	21
hsa04935	Growth hormone synthesis; secretion and action	21/888	119/8101	0.0182652999842459	0.0739744649361958	0.0528732367965013	FOS/MAPK13/PIK3CD/CREB5/PTK2/PLCB2/IRS2/MAPK3/SSTR2/GNA11/MAP3K1/PIK3R2/GNAQ/PRKCB/PLCG1/SOCS3/PRKCA/SOCS2/SOCS1/CREB3L4/PRKACB	21
hsa04215	Apoptosis - multiple species	8/888	32/8101	0.0193792701912077	0.0775170807648308	0.0554053208845444	BIRC3/CYCS/CASP3/CASP9/BCL2L11/CASP7/SEPT4/PMAIP1	8

hsa04658	Th1 and Th2 cell differentiation	17/888	92/8101	0.0209495385145476	0.0827762253501638	0.0591642936225992	FOS/MAPK13/CD4/MAF/MAPK3/NFATC1/NOTCH3/HLA-DMB/RBPJ/NFKBIA/PPP3CA/MAML3/PPP3CC/PLCG1/NFKBIE/RUNX3/HLA-DMA	17
hsa04922	Glucagon signaling pathway	19/888	107/8101	0.0225827994493803	0.0874486990211269	0.0625039434199088	CALML4/PYGL/SLC2A1/CREB5/PLCB2/CALM3/PRMT1/CAMK2A/PFKP/PCK2/CAMK2G/GNAQ/PPP3CA/ACACA/PPP3CC/FBP1/ACACB/CREB3L4/PRKACB	19
hsa04621	NOD-like receptor signaling pathway	29/888	181/8101	0.0226718849314033	0.0874486990211269	0.0625039434199088	NLRC4/BIRC3/MAPK13/TLR4/TRAF5/NLRP1/PSTPIP1/PLCB2/HSP90AB1/TXNIP/HSP90AA1/MAPK3/GABARAPL1/GBP1/GSDMD/TRAF6/CASP1/GBP4/NFKBIA/P2RX7/GBP2/TNFAIP3/NAIP/NOD1/TXN/CXCL1/TNF/CASP5/RIPK2	29
hsa04912	GnRH signaling pathway	17/888	93/8101	0.0231149993579597	0.0881089387291641	0.0629758496439769	CALML4/MAPK13/PLCB2/CALM3/PLA2G4A/CAMK2A/MAPK3/MAP3K3/GNA11/MAP3K1/CAMK2G/GNAQ/PRKCB/HBEGF/PRKCA/MMP2/PRKACB	17
hsa04022	cGMP-PKG signaling pathway	27/888	167/8101	0.0244775224800549	0.0922176428318349	0.06591254523393	CALML4/MEF2D/GUCY1A3/VASP/INSR/SLC8A3/CREB5/PLCB2/ATP2A3/PPP1CB/IRS2/CALM3/PIK3R6/MAPK3/NFATC1/PIK3CG/GNA11/MYL9/ADORA3/GNAQ/PPP3CA/BAD/PPP3CC/MYLK/ATP2B1/GTF2IRD1/CREB3L4	27
hsa04930	Type II diabetes mellitus	10/888	46/8101	0.0248333468445514	0.0661020726654725	0.0661020726654725	HK3/INSR/PIK3CD/IRS2/MAPK3/PIK3R2/TNF/SOCS3/SOCS2/SOCS1	10

hsa04611	Platelet activation	21/888	124/8101	0.0278412438776683	0.101238361409006	0.0723601023716093	PTGS1/GUCY1A3/MAPK13/VASP/ITGB3/PIK3CD/APBB1IP/PLCB2/PPP1CB/TBXAS1/PLA2G4A/PIK3R6/MAPK3/PIK3CG/P2RX1/SYK/RASGRP1/PIK3R2/GNAQ/MYLK/PRKACB	21
hsa00650	Butanoate metabolism	7/888	28/8101	0.0280229687750883	0.101238361409006	0.0723601023716093	HMGCS1/ACAT2/L2HGDH/AACS/ABAT/HADH/OXCT1	7
hsa04720	Long-term potentiation	13/888	67/8101	0.0281217670580572	0.101238361409006	0.0723601023716093	CALML4/PLCB2/PPP1CB/CALM3/CAMK2A/MAPK3/CAMK2G/GNAQ/PPP3CA/PRKCB/PPP3CC/PRKCA/PRKACB	13
hsa05167	Kaposi sarcoma-associated herpesvirus infection	30/888	193/8101	0.0301349064562857	0.107293513097105	0.0766880210570603	FOS/CALML4/MAPK13/PIK3CD/TCF7L2/CALM3/PIK3R6/CYCS/MAPK3/NFATC1/GABARAPL1/PIK3CG/MYC/E2F2/CDKN1A/ZFP36/SYK/CASP3/PIK3R2/UBB/NFKBIA/PPP3CA/CASP9/CD86/PPP3CC/GNGT2/GNB5/CXCL1/CD200R1/PLCG1	30
hsa05150	Staphylococcus aureus infection	17/888	96/8101	0.0306325252989255	0.107879763009259	0.0771070430865402	SELPLG/C5AR1/ITGAM/FCGR3A/FCAR/FCGR2B/ITGAL/FPR3/ITGB2/HLA-DMB/FCGR3B/FPR2/FPR1/C1QA/IL10/HLA-DMA/CFB	17
hsa00280	Valine; leucine and isoleucine degradation	10/888	48/8101	0.0326607306991536	0.112912848736228	0.0807044402923006	HMGCS1/MCCC2/ACAT2/BCAT1/ALDH2/ALDH1B1/AACS/ABAT/HADH/OXCT1	10
hsa04660	T cell receptor signaling pathway	18/888	104/8101	0.0327586659913747	0.112912848736228	0.0807044402923006	FOS/MAPK13/PIK3CD/PTPN6/VAV2/CD4/MAPK3/NFATC1/RASGRP1/PIK3R2/NFKBIA/PPP3CA/VAV1/PPP3CC/TNF/PLCG1/NFKBIE/IL10	18

hsa04071	Sphingolipid signaling pathway	20/888	119/8101	0.0336447174496122	0.114746194249204	0.0820148236998858	MAPK13/PIK3CD/SGPL1/PLCB2/MAPK3/SPHK1/RAC2/CERS6/MAP3K5/SGMS2/ADORA3/PIK3R2/GNAQ/PRKCB/SPHK2/TNF/S1PR4/PRKCA/SGPP2/PPP2R1B	20
hsa03320	PPAR signaling pathway	14/888	76/8101	0.0349300412739759	0.116673539925445	0.0833923937088952	HMGCS1/RXRA/PLTP/SLC27A1/OLR1/PPARD/CD36/PCK2/FABP5/LPL/PLIN2/CYP27A1/ME1/SCD	14
hsa05212	Pancreatic cancer	14/888	76/8101	0.0349300412739759	0.116673539925445	0.0833923937088952	TGFBR2/RALA/PIK3CD/TGFA/MAPK3/RAC2/E2F2/CDKN1A/GADD45B/TGFB1/PIK3R2/CASP9/BAD/GADD45G	14
hsa05120	Epithelial cell signaling in Helicobacter pylori infection	13/888	70/8101	0.0388201043046374	0.128344018313291	0.0917338662408187	MAPK13/ATP6V0D2/ATP6V0A1/MET/ATP6V0A2/ATP6V0E2/TJP1/CASP3/NFKBIA/NOD1/CXCL1/HBEGF/PLCG1	13
hsa04926	Relaxin signaling pathway	21/888	129/8101	0.040812409104064	0.133567884340573	0.0954676236352375	TGFBR2/EDN1/FOS/MAPK13/ARRB2/PIK3CD/CREB5/ACTA2/PLCB2/ARRB1/MAPK3/MMP9/TGFB1/PIK3R2/NFKBIA/GNGT2/GNB5/PRKCA/MMP2/CREB3L4/PRKACB	21
hsa04012	ErbB signaling pathway	15/888	85/8101	0.0417314321378392	0.135209840126599	0.0966412112665751	PIK3CD/PTK2/TGFA/CAMK2A/MAPK3/MYC/CDKN1A/CAMK2G/PIK3R2/EIF4EBP1/PRKCB/BAD/HBEGF/PLCG1/PRKCA	15
hsa04929	GnRH secretion	12/888	64/8101	0.0431356340106604	0.138375697222317	0.0989040071114673	ARRB2/PIK3CD/PLCB2/SPP1/ARRB1/MAPK3/GNA11/PIK3R2/GNAQ/PRKCB/KCNJ5/PRKCA	12
hsa04960	Aldosterone-regulated sodium reabsorption	8/888	37/8101	0.0437295887373893	0.138905752459943	0.0992828640064567	INSR/PIK3CD/SGK1/MAPK3/PIK3R2/PRKCB/PRKCA/NEDD4L	8

hsa04915	Estrogen signaling pathway	22/888	138/8101	0.045191112586208	0.142154567746907	0.101604954205067	FOS/CALML4/HSPA2/PIK3CD/RARA/CREB5/PLCB2/HSP90AB1/CALM3/TGFA/HSP90AA1/MAPK3/MMP9/HSPA6/PIK3R2/GNAQ/HBEGF/KCNJ5/HSPA8/MMP2/CREB3L4/PRKACB	22
hsa04650	Natural killer cell mediated cytotoxicity	21/888	131/8101	0.0470796621012459	0.146671255007728	0.104833255691033	PIK3CD/PTPN6/VAV2/HCST/FCGR3A/MAPK3/NFATC1/ITGAL/RAC2/SYK/CASP3/PIK3R2/ITGB2/FCGR3B/PPP3CA/PRKCB/VAV1/PPP3CC/TNF/PLCG1/PRKCA	21

IFNL4 VS NT   M2-MDM KEGG Enrichment

PATH_ID	Description	GeneRatio	BgRatio	pvalue	p.adjust	qvalue	geneID	Count
hsa05146	Amoebiasis	311/2020	102/8101	0.00189082967039053	0.119945939488296	0.107320051121107	ARG2/CXCL8/IL1R2	3
hsa04064	NF-kappa B signaling pathway	311/2020	104/8101	0.0019990989914716	0.119945939488296	0.107320051121107	CXCL8/GADD45A/LBP	3
hsa05418	Fluid shear stress and atherosclerosis	311/2020	139/8101	0.00455047390605563	0.177541582555718	0.158852994918274	IL1R2/NCF2/SDC2	3
hsa05144	Malaria	211/2020	50/8101	0.0066077719016975	0.177541582555718	0.158852994918274	CXCL8/SDC2	2
hsa05134	Legionellosis	211/2020	57/8101	0.00852052236820644	0.177541582555718	0.158852994918274	CXCL8/HSPA2	2
hsa05202	Transcriptional misregulation in cancer	311/2020	192/8101	0.0111002553827198	0.177541582555718	0.158852994918274	CXCL8/GADD45A/IL1R2	3
hsa05120	Epithelial cell signaling in Helicobacter pylori infection	211/2020	70/8101	0.0126481899861573	0.177541582555718	0.158852994918274	CXCL8/NOD1	2
hsa05218	Melanoma	211/2020	72/8101	0.0133472792080709	0.177541582555718	0.158852994918274	GADD45A/PDGFRA	2
hsa05214	Glioma	211/2020	75/8101	0.0144270105318357	0.177541582555718	0.158852994918274	GADD45A/PDGFRA	2
hsa05133	Pertussis	211/2020	76/8101	0.0147951318796432	0.177541582555718	0.158852994918274	CXCL8/NOD1	2
hsa04144	Endocytosis	311/2020	251/8101	0.0226886464984121	0.228713605302344	0.204638488954728	ARAP3/HSPA2/PDGFRA	3
hsa05215	Prostate cancer	211/2020	97/8101	0.0234355537981403	0.228713605302344	0.204638488954728	IL1R2/PDGFRA	2

hsa04061	Viral protein interaction with cytokine and cytokine receptor	211/2020	100/8101	0.0248058371601275	0.228713605302344	0.204638488954728	CXCL8/TNFRSF10C	2
hsa04620	Toll-like receptor signaling pathway	211/2020	104/8101	0.0266832539519401	0.228713605302344	0.204638488954728	CXCL8/LBP	2
hsa04010	MAPK signaling pathway	311/2020	294/8101	0.0341425349342495	0.258294561331729	0.231105660138915	GADD45A/HSPA2/PDGFRA	3
hsa04060	Cytokine-cytokine receptor interaction	311/2020	295/8101	0.0344392748442305	0.258294561331729	0.231105660138915	CXCL8/IL1R2/TNFRSF10C	3
hsa04380	Osteoclast differentiation	211/2020	128/8101	0.039093274214327	0.275952523865837	0.246904889774697	NCF2/SOCS3	2
hsa04910	Insulin signaling pathway	211/2020	137/8101	0.0442209595578963	0.28906999698497	0.25864157624971	FBP1/SOCS3	2

IFN-L4 vs IFN-L3 M2-MDM KEGG

PATH_ID	Description	GeneRatio	BgRatio	pvalue	p.adjust	qvalue	geneID	Count
hsa05152	Tuberculosis	50/874	180/8101	1.27450254786374E-10	4.06566312768532E-08	2.65633162607389E-08	ATP6V0A1/ATP6V0D2/BAD/BCL2/CALM3/CALML4/CAMK2A/CAMK2G/CASP3/CD14/CD209/CIITA/CLEC7A/CORO1A/CR1/CR1L/CYCS/CYP27B1/FCGR1A/FCGR2A/FCG	50

							R2B/FCGR2C/FCGR3A/FCGR3B/HLA-DMA/HLA-DMB/HLA-DOB/HSPD1/IL1A/IRAK2/ITGAM/ITGAX/ITGB2/KSR1/LSP1/MAPK13/MAPK3/MRC1/NFKB1/PPP3CA/PPP3CC/RIPK2/SPHK1/SRC/SYK/TGFB1/TLR4/TLR6/TNF/VDR	
hsa05134	Legionellosis	22/874	57/8101	3.56413068141313E-08	5.68478843685394E-06	3.71419934168315E-06	BNIP3/CASP1/CASP3/CASP7/CD14/CR1/CR1L/CYCS/HSPA2/HSPA6/HSPD1/ITGAM/ITGB2/NAIP/NFKB1/NFKB2/NFKBIA/NLRC4/PYCARD/TLR4/TLR5/TNF	22
hsa05140	Leishmaniasis	25/874	77/8101	2.25315665639932E-07	0.0000235829621763274	0.000015408105959125	CR1/CR1L/FCGR1A/FCGR2A/FCGR2C/FCGR3A/FCGR3B/FOS/HLA-DMA/HLA-DMB/HLA-DOB/IL1A/ITGAM/ITGB2/MAPK13/MAPK3/MARCKSL1/NCF2/NFKB1/NFKBIA/PRKCB/PTPN6/TGFB1/TLR4/TNF	25
hsa04062	Chemokine signaling pathway	45/874	192/8101	2.95711124468055E-07	0.0000235829621763274	0.000015408105959125	ARRB2/BAD/CCL13/CCL18/CCL19/CCL20/CCL22/CCL23/CCL28/CCL3/CCL4/CCL4L1/CCL4L2/CCL7/CCL8/CCR7/CXCL10/CXCL11/CXCL13/CXCL16/CXCL5/CXCL6/CXCL9/CXCR4/CXCR5/GNGT2/GRK6/MAPK3/NFKB1/NFKBIA/PIK3CD/PIK3CG/PIK3R2/PIK3R5/PIK3R6/PLCB2/PLCB4/PPBP/PRKACB/PRKCB/PTK2/RAC2/SRC/VAV1/VAV2	45
hsa04380	Osteoclast differentiation	34/874	128/8101	3.92335436749014E-07	0.0000250310008645871	0.0000163541929423799	CSF1R/CTSK/FCGR1A/FCGR2A/FCGR2B/FCGR2C/FCGR3A/FCGR3B/FOS/FOSL1/IL1A/IL1R1/ITGB3/LILRA1/LILRA5/LILRA6/LILRB3/LILRB5/MAP2K6/MAPK13/MAPK3/NCF2/NFKB1/NFKB2/NFKBIA/PIK3CD/PIK3R2/PPP3CA/PPP3CC/SIRPB1/SYK/TGFB1/TGFBR2/TNF	34
hsa05146	Amoebiasis	29/874	102/8101	6.0512328231	0.0000321723878433069	0.0000210200719121424	ACTN1/CASP3/CD14/COL1A2/COL4A2/FN1/GNA11/IL1R1/IL1R2/ITGAM/ITGB2/LAMA3/LAMB1/LAMB3/LAMC1/NFKB1/PIK	29

				9251E-07			3CD/PIK3R2/PLCB2/PLCB4/PRDX1/PRKACB/PRKCA/PRKCB/PTK2/SERPINB9/TGFB1/TLR4/TNF	
hsa04218	Cellular senescence	38/874	156/8101	8.9991157918693E-07	0.0000410102562515187	0.0000267943598013552	CALM3/CALML4/CCNA1/CCNA2/CCNB1/CCNB2/CCND3/CDK1/CDK6/CDKN1A/CDKN2B/E2F2/EIF4EBP1/FOXM1/GADD45B/GADD45G/HIPK2/IL1A/ITPR2/LIN9/MAP2K6/MAPK13/MAPK3/MYC/NBN/NFKB1/PIK3CD/PIK3R2/PPP1CB/PPP3CA/PPP3CC/RRAS/SERPINE1/TGFB1/TGFBR2/TRPV4/ZFP36L1/ZFP36L2	38
hsa00100	Steroid biosynthesis	11/874	20/8101	1.45325893403508E-06	0.0000553009060628397	0.000036131263489332 7	CYP27B1/CYP51A1/DHCR24/DHCR7/EBP/FDFT1/HSD17B7/MSMO1/SC5D/SOAT1/SQLE	11
hsa05145	Toxoplasmosis	30/874	112/8101	1.56021365067573E-06	0.0000553009060628397	0.0000361312634893327	ALOX5/BAD/BCL2/BIRC3/CASP3/CD40/CIITA/CYCS/HLA-DMA/HLA-DMB/HLA-DOB/HSPA2/HSPA6/LAMA3/LAMB1/LAMB3/LAMC1/LDLR/LY96/MAP2K6/MAPK13/MAPK3/NFKB1/NFKBIA/PIK3CG/PIK3R5/PIK3R6/TGFB1/TLR4/TNF	30
hsa05222	Small cell lung cancer	26/874	92/8101	2.60672478524982E-06	0.0000831545206494691	0.0000543296323662593	BCL2/BIRC3/CASP3/CDK6/CDKN1A/CDKN2B/COL4A2/CYCS/E2F2/FN1/GADD45B/GADD45G/LAMA3/LAMB1/LAMB3/LAMC1/MYC/NFKB1/NFKBIA/PIK3CD/PIK3R2/PTK2/RXRA/TRAF1/TRAF4/TRAF5	26
hsa04928	Parathyroid hormone synthesis; secretion and action	28/874	106/8101	4.65688177391345E-06	0.00013504957144349	0.0000882356546636233	AKAP13/ARRB2/BCL2/CDKN1A/CREB3L4/CREB5/CYP27B1/EGR1/FOS/GNA11/HBEGF/ITPR2/KL/MAPK3/MEF2D/MMP25/NR4A2/PDE4A/PDE4B/PLCB2/PLCB4/PRKACB/PRKCA/PRKCB/RUNX2/RXRA/SLC9A3R1/VDR	28

hsa04145	Phagosome	35/874	152/8101	9.37028958832133E-06	0.000239176542748909	0.000156267795625422	ATP6V0A1/ATP6V0D2/ATP6V0E2/CD14/CD209/CD36/CLEC7A/COLEC12/CORO1A/DYNC2H1/FCAR/FCGR1A/FCGR2A/FCGR2B/FCGR2C/FCGR3A/FCGR3B/HLA-DMA/HLA-DMB/HLA-DOB/ITGAM/ITGB2/ITGB3/ITGB5/MARCO/MRC1/NCF2/OLR1/SCARB1/TLR4/TLR6/TUBA1B/TUBA1C/TUBB/TUBB4B	35
hsa04620	Toll-like receptor signaling pathway	27/874	104/8101	9.74700644431291E-06	0.000239176542748909	0.000156267795625422	CCL3/CCL4/CCL4L1/CCL4L2/CD14/CD40/CD80/CD86/CTSK/CXCL10/CXCL11/CXCL9/FOS/LY96/MAP2K6/MAPK13/MAPK3/NFKB1/NFKBIA/PIK3CD/PIK3R2/SPP1/TLR4/TLR5/TLR6/TLR7/TNF	27
hsa04061	Viral protein interaction with cytokine and cytokine receptor	26/874	100/8101	0.0000137950358636769	0.000314329745750923	0.000205369706842708	CCL13/CCL18/CCL19/CCL20/CCL22/CCL23/CCL28/CCL3/CCL4/CCL4L1/CCL4L2/CCL7/CCL8/CCR7/CSF1R/CXCL10/CXCL11/CXCL13/CXCL5/CXCL6/CXCL9/CXCR4/CXCR5/PPBP/TNF/TNFRSF10C	26
hsa05202	Transcriptional misregulation in cancer	40/874	192/8101	0.0000280800982063132	0.000584060364493512	0.000381600238144581	BIRC3/CCNA1/CCNA2/CD14/CD40/CD86/CDKN1A/CDKN2C/CSF1R/DOT1L/DUSP6/ETV5/FCGR1A/FLI1/FLT1/GADD45B/GADD45G/GZMB/HHEX/ID2/IL1R2/ITGAM/ITGB7/LMO2/LYL1/MAF/MET/MMP9/MYC/NFKB1/NGFR/NR4A3/PBX3/PTK2/RARA/RUNX2/RXRA/SUPT3H/TGFBR2/TRAF1	40
hsa04060	Cytokine-cytokine receptor interaction	55/874	295/8101	0.0000292945637363517	0.000584060364493512	0.000381600238144581	BMP2/CCL13/CCL18/CCL19/CCL20/CCL22/CCL23/CCL28/CCL3/CCL4/CCL4L1/CCL4L2/CCL7/CCL8/CCR7/CD4/CD40/CD70/CSF1R/CXCL10/CXCL11/CXCL13/CXCL16/CXCL5/CXCL6/CXCL9/CXCR4/CXCR5/EBI3/EPOR/IL15RA/IL16/IL17RA/IL1A/IL1R1/IL1R2/I	55

							L21R/IL27/IL7/LEPR/LIFR/NGFR/PPBP/PRLR/TGFB1/TGFBR2/TNF/TNFRSF10C/TNFRSF6B/TNFRSF8/TNFRSF9/TNFSF12/TNFSF13/TNFSF15/TNFSF8	
hsa05133	Pertussis	21/874	76/8101	0.0000343861841323375	0.000622971534233679	0.000407023143963928	C1QA/CALM3/CALML4/CASP1/CASP3/CASP7/CD14/CXCL5/CXCL6/FOS/IL1A/IRF1/ITGAM/ITGB2/LY96/MAPK13/MAPK3/NFKB1/PYCARD/TLR4/TNF	21
hsa04210	Apoptosis	31/874	136/8101	0.0000370734117597273	0.000622971534233679	0.000407023143963928	BAD/BCL2/BCL2L11/BIRC3/BIRC5/CASP3/CASP7/CTSC/CTSK/CYCS/ENDOG/FOS/GADD45B/GADD45G/GZMB/ITPR2/LMNB1/LMNB2/MAP3K5/MAPK3/NFKB1/NFKBIA/PIK3CD/PIK3R2/PMAIP1/SEPT4/SPTAN1/TNF/TRAF1/TUBA1B/TUBA1C	31
hsa04662	B cell receptor signaling pathway	22/874	82/8101	0.0000371048876189338	0.000622971534233679	0.000407023143963928	CD72/FCGR2B/FOS/LILRA1/LILRA5/LILRA6/LILRB3/LILRB5/MAPK3/NFKB1/NFKBIA/NFKBIE/PIK3CD/PIK3R2/PPP3CA/PPP3CC/PRKCB/PTPN6/RAC2/SYK/VAV1/VAV2	22
hsa05221	Acute myeloid leukemia	19/874	67/8101	0.0000545473117498539	0.0008700296224101 7	0.000568440406656372	BAD/CCNA1/CCNA2/CD14/CSF1R/DUSP6/EIF4EBP1/FCGR1A/ITGAM/MAPK3/MYC/NFKB1/PIK3CD/PIK3R2/PIM1/PPARD/RARA/TCF7L1/TCF7L2	19
hsa05163	Human cytomegalovirus infection	44/874	225/8101	0.0000575393599603653	0.000874050277493169	0.000571067331937461	AKAP13/CALM3/CALML4/CASP3/CCL3/CCL4/CCL4L1/CCL4L2/CDK6/CDKN1A/CREB3L4/CREB5/CXCR4/CYCS/E2F2/EIF4EBP1/GNA11/GNGT2/IL1R1/ITGB3/ITPR2/MAP2K6/MAPK13/MAPK3/MB21D1/MYC/NFKB1/NFKBIA/PIK3CD/PIK3R2/PLCB2/PLCB4/PPP3CA/PPP3CC/PRKACB/PRKCA/PRKCB/PTGER4/PTK2/RAC2/SRC/TMEM173/TNF/TRAF5	44
hsa04151	PI3K-Akt signaling pathway	62/874	354/8101	0.0000656688	0.000952198827137576	0.000622126275443788	BAD/BCL2/BCL2L11/BRCA1/CCND3/CDK6/CDKN1A/COL1A2/COL4A2/COL6A1/COL6A2/COL9A2/CREB3L4/CREB5/CSF1R/DDI	62

				846301776			T4/EIF4EBP1/EPOR/FLT1/FN1/GNGT2/HSP90AA1/HSP90AB1/IL7/INSR/ITGA1/ITGB3/ITGB5/ITGB7/ITGB8/LAMA3/LAMB1/LAMB3/LAMC1/MAPK3/MET/MYC/NFKB1/NGFR/PCK2/PDGFC/PHLPP1/PIK3CD/PIK3CG/PIK3R2/PIK3R5/PIK3R6/PKN3/PPP2R1B/PPP2R3B/PRKCA/PRLR/PTK2/RXRA/SGK1/SGK3/SPP1/SYK/TGFA/TLR4/TNC/VTN	
hsa05210	Colorectal cancer	22/874	86/8101	0.000081431733777284	0.00105958818260174	0.000692289919667195	BAD/BCL2/BCL2L11/BIRC5/CASP3/CDKN1A/CYCS/FOS/GADD45B/GADD45G/MAPK3/MYC/PIK3CD/PIK3R2/PMAIP1/RAC2/RALA/TCF7L1/TCF7L2/TGFA/TGFB1/TGFBR2	22
hsa04625	C-type lectin receptor signaling pathway	25/874	104/8101	0.0000828330244710055	0.00105958818260174	0.000692289919667195	CALM3/CALML4/CASP1/CCL22/CD209/CLEC6A/CLEC7A/IRF1/ITPR2/KSR1/LSP1/MAPK13/MAPK3/NFKB1/NFKB2/NFKBIA/PIK3CD/PIK3R2/PPP3CA/PPP3CC/PYCARD/RRAS/SRC/SYK/TNF	25
hsa04510	Focal adhesion	40/874	201/8101	0.0000830398262227065	0.00105958818260174	0.000692289919667195	CTN1/BAD/BCL2/BIRC3/CCND3/COL1A2/COL4A2/COL6A1/COL6A2/COL9A2/FLT1/FN1/ITGA1/ITGB3/ITGB5/ITGB7/ITGB8/LAMA3/LAMB1/LAMB3/LAMC1/MAPK3/MET/MYL9/MYLK/PDGFC/PIK3CD/PIK3R2/PPP1CB/PRKCA/PRKCB/PTK2/RAC2/SPP1/SRC/TNC/VASP/VAV1/VAV2/VTN	40
hsa04668	TNF signaling pathway	26/874	112/8101	0.000112092645432516	0.00134791587696539	0.00088067099039481	BIRC3/CASP3/CASP7/CCL20/CREB3L4/CREB5/CXCL10/CXCL5/CXCL6/EDN1/FOS/IRF1/JAG1/MAP2K6/MAP3K5/MAPK13/MAPK3/MMP9/NFKB1/NFKBIA/PIK3CD/PIK3R2/TNF/TNFAIP3/TRAF1/TRAF5	26
hsa04933	AGE-RAGE signaling pathway in diabetic	24/874	100/8101	0.000117369938982229	0.00134791587696539	0.00088067099039481	BCL2/CASP3/COL1A2/COL4A2/EDN1/EGR1/FN1/IL1A/MAPK13/MAPK3/MMP2/NFKB1/PIK3CD/PIK3R2/PIM1/PLCB2/PLCB4/PRKCA/PRKCB/SERPINE1/TGFB1/TGFBR2/THBD/TNF	24

	complications							
hsa04657	IL-17 signaling pathway	23/874	94/8101	0.000118312365376272	0.00134791587696539	0.00088067099039481	CASP3/CCL20/CCL7/CXCL10/CXCL5/CXCL6/FOS/FOSL1/HSP90AA1/HSP90AB1/IL17RA/MAPK13/MAPK3/MAPK6/MMP9/NFKB1/NFKBIA/S100A8/S100A9/TNF/TNFAIP3/TRAF4/TRAF5	
hsa05166	Human T-cell leukemia virus 1 infection	42/874	219/8101	0.000133332072776019	0.0014666528005362	0.000958248653707865	BUB1B/CCNA1/CCNA2/CCNB2/CCND3/CD4/CD40/CDKN1A/CDKN2B/CDKN2C/CREB3L4/CREB5/E2F2/EGR1/FDPS/FOS/FOSL1/HLA-DMA/HLA-DMB/HLA-DOB/IL15RA/IL1R1/IL1R2/ITGAL/ITGB2/MAP3K3/MAPK3/MYC/NFKB1/NFKB2/NFKBIA/NRP1/PIK3CD/PIK3R2/PPP3CA/PPP3CC/PRKACB/SLC2A1/TGFB1/TGFBR2/TNF/ZFP36	42
hsa05418	Fluid shear stress and atherosclerosis	30/874	139/8101	0.000142517409001666	0.00151543511571772	0.00099012094674842	BCL2/CALM3/CALML4/EDN1/FOS/GSTM4/HSP90AA1/HSP90AB1/IL1A/IL1R1/IL1R2/ITGB3/MAP2K6/MAP3K5/MAPK13/MGST1/MMP2/MMP9/NCF2/NFKB1/PIK3CD/PIK3R2/PTK2/RAC2/SDC4/SRC/THBD/TNF/TRPV4/TXN	30
hsa05132	Salmonella infection	46/874	249/8101	0.000163425241268005	0.00168169845046754	0.00109875034876282	AHNAK/AHNAK2/BCL2/BIRC3/CASP1/CASP3/CASP7/CD14/CYCS/CYFIP2/CYTH3/DYNC2H1/DYNLT3/FOS/GSDMD/HSP90AA1/HSP90AB1/LY96/MAP2K6/MAPK13/MAPK3/MYC/MYL9/NAIP/NFKB1/NFKBIA/NLRC4/PIK3CD/PIK3CG/PYCARD/RALA/RIPK2/RRAS/S100A10/STX10/TCF7L1/TCF7L2/TLR4/TLR5/TLR6/TNF/TUBA1B/TUBA1C/TUBB/TUBB4B/TXN	46
hsa04064	NF-kappa B signaling pathway	24/874	104/8101	0.0002239705	0.00223270637981239	0.00145875552946	BCL2/BIRC3/CCL13/CCL19/CCL4/CCL4L1/CCL4L2/CD14/CD40/EDARADD/GADD45B/GADD45G/IL1R1/LY96/NFKB1/NFKB2/NF	24

				45937293			KBIA/PRKCB/SYK/TLR4/TNF/TNFAIP3/TRAF1/TRAF5	
hsa04613	Neutrophil extracellular trap formation	37/874	190/8101	0.000240041460473262	0.00232040078457486	0.00151605132930481	C5AR1/CASP1/CLEC7A/CR1/CR1L/FCGR1A/FCGR2A/FCGR3A/FCGR3B/FPR1/FPR2/GSDMD/H2AFY/H2AFY2/HDAC5/HIST1H2BK/ITGAL/ITGAM/ITGB2/ITGB3/MAPK13/MAPK3/NCF2/NFKB1/PIK3CD/PIK3R2/PLCB2/PLCB4/PRKCA/PRKCB/RAC2/SELPLG/SIGLEC9/SRC/SYK/TLR4/TLR7	37
hsa00900	Terpenoid backbone biosynthesis	9/874	22/8101	0.000257086964085773	0.00241208063362828	0.00157595104919452	ACAT2/FDPS/FNTB/HMGCR/HMGCS1/IDI1/MVD/MVK/RCE1	9
hsa05323	Rheumatoid arthritis	22/874	93/8101	0.000278197661030804	0.00253557296768076	0.00165663569576238	ATP6V0A1/ATP6V0D2/ATP6V0E2/CCL20/CCL3/CD80/CD86/CTSK/CXCL5/CXCL6/FLT1/FOS/HLA-DMA/HLA-DMB/HLA-DOB/IL1A/ITGAL/ITGB2/TGFB1/TLR4/TNF/TNFSF13	22
hsa04520	Adherens junction	18/874	71/8101	0.000399388090604266	0.00353902224729892	0.00231224684034049	ACTN1/INSR/MAPK3/MET/MLLT4/PTPN6/PTPRM/PVRL1/PVRL3/PVRL4/RAC2/SRC/SSX2IP/TCF7L1/TCF7L2/TGFBR2/TJP1/YES1	18
hsa00534	Glycosaminoglycan biosynthesis - heparan sulfate / heparin	9/874	24/8101	0.000553715679547193	0.00477392707501499	0.00311908121053611	EXT1/EXTL2/GLCE/HS2ST1/HS3ST1/HS3ST3A1/HS3ST3B1/NDST1/XYLT2	9
hsa01524	Platinum drug resistance	18/874	73/8101	0.000574166233766086	0.00481997443608898	0.0031491666007115	BAD/BCL2/BIRC3/BIRC5/BRCA1/CASP3/CDKN1A/CYCS/GSTM4/MAP3K5/MAPK3/MGST1/PIK3CD/PIK3R2/PMAIP1/REV3L/SLC31A1/TOP2A	18
hsa04750	Inflammatory	22/874	98/8101	0.0006056263	0.0049537129194777	0.00323654564611973	ASIC1/CALM3/CALML4/CAMK2A/CAMK2G/HTR2B/IL1R1/ITPR2/MAP2K6/MAPK13	22

	mediator regulation of TRP channels			443875 56			/P2RY2/PIK3CD/PIK3R2/PLCB2/PLCB4/PP P1CB/PRKACB/PRKCA/PRKCB/PTGER4/SR C/TRPV4	
hsa04640	Hematopoi etic cell lineage	22/874	99/8101	0.0007 012870 291476 26	0.0055927 640574523 1	0.00365407 452029552	CD14/CD33/CD36/CD37/CD4/CD9/CR1/C R1L/CSF1R/EPOR/FCGR1A/HLA-DMA/HLA-DMB/HLA-DOB/IL1A/IL1R1/IL1R2/IL7/ITGA1/ITGAM /ITGB3/TNF	22
hsa04659	Th17 cell differentiat ion	23/874	107/8101	0.0008 749133 334682 76	0.0068046 292697514 2	0.00444585 57842296	CD4/FOS/HLA-DMA/HLA-DMB/HLA-DOB/HSP90AA1/HSP90AB1/IL1R1/IL21R/I RF4/MAPK13/MAPK3/NFKB1/NFKBIA/NF KBIE/PPP3CA/PPP3CC/RARA/RORA/RXRA /TBX21/TGFB1/TGFBR2	23
hsa04512	ECM-receptor interaction	20/874	88/8101	0.0008 959073 019735 41	0.0068046 292697514 2	0.00444585 57842296	CD36/COL1A2/COL4A2/COL6A1/COL6A2/ COL9A2/FN1/ITGA1/ITGB3/ITGB5/ITGB7/ ITGB8/LAMA3/LAMB1/LAMB3/LAMC1/S DC4/SPP1/TNC/VTN	20
hsa05235	PD-L1 expression and PD-1 checkpoint pathway in cancer	20/874	89/8101	0.0010 412706 113122 8	0.0077247 750002003 8	0.00504703 992753564	ALK/BATF3/CD274/CD4/EML4/FOS/MAP 2K6/MAP3K3/MAPK13/MAPK3/NFKB1/N FKBIA/NFKBIE/PIK3CD/PIK3R2/PPP3CA/P PP3CC/PTPN6/RASGRP1/TLR4	20
hsa05205	Proteoglyc ans in cancer	37/874	205/8101	0.0010 889662 851189 3	0.0078181 492570406 5	0.00510804 670151476	CAMK2A/CAMK2G/CASP3/CDKN1A/COL1 A2/FN1/HBEGF/ITGB3/ITGB5/ITPR2/LUM /MAPK13/MAPK3/MET/MMP2/MMP9/M YC/PDCD4/PIK3CD/PIK3R2/PPP1CB/PRKA CB/PRKCA/PRKCB/PTK2/PTPN6/RRAS/SD C4/SRC/TGFB1/TIMP3/TLR4/TNF/VAV1/V AV2/VTN/WNT5A	37
hsa04514	Cell adhesion molecules	29/874	149/8101	0.0011 028737	0.0078181 492570406 5	0.00510804 670151476	C10orf54/CADM1/CD274/CD276/CD4/CD 40/CD80/CD86/CLDN23/CLDN7/HLA-DMA/HLA-DMB/HLA-	29

				1964523			DOB/ICOSLG/ITGAL/ITGAM/ITGB2/ITGB7/ITGB8/NRCAM/PDCD1LG2/PTPRM/PVR/PVRL1/PVRL3/SDC3/SDC4/SELPLG/VCAN	
hsa05215	Prostate cancer	21/874	97/8101	0.00131080948468927	0.00909017881773649	0.0059391367956173	BAD/BCL2/CDKN1A/CREB3L4/CREB5/E2F2/ETV5/HSP90AA1/HSP90AB1/IL1R2/MAPK3/MMP9/NFKB1/NFKBIA/NKX3-1/PDGFC/PIK3CD/PIK3R2/TCF7L1/TCF7L2/TGFA	21
hsa04611	Platelet activation	25/874	124/8101	0.00141576931781224	0.00945638385651374	0.00617839961587105	APBB1IP/COL1A2/FCGR2A/GUCY1A3/ITGB3/ITPR2/MAPK13/MAPK3/MYLK/P2RX1/PIK3CD/PIK3CG/PIK3R2/PIK3R5/PIK3R6/PLCB2/PLCB4/PPP1CB/PRKACB/PTGS1/RASGRP1/SRC/SYK/TBXAS1/VASP	25
hsa04215	Apoptosis - multiple species	10/874	32/8101	0.00142290415395818	0.00945638385651374	0.00617839961587105	BCL2/BCL2L11/BIRC3/BIRC5/CASP3/CASP7/CYCS/NGFR/PMAIP1/SEPT4	10
hsa04068	FoxO signaling pathway	26/874	131/8101	0.00145680406375087	0.00948409176196994	0.00619650278459016	BCL2L11/BNIP3/CCNB1/CCNB2/CDKN1A/CDKN2B/CDKN2D/FBXO32/FOXO4/GADD45B/GADD45G/HOMER3/INSR/IRS2/MAPK13/MAPK3/PCK2/PIK3CD/PIK3R2/PLK1/PRMT1/S1PR4/SGK1/SGK3/TGFB1/TGFBR2	26
hsa04672	Intestinal immune network for IgA production	13/874	49/8101	0.00159295272235342	0.0101630383686148	0.00664009766370477	CCL28/CD40/CD80/CD86/CXCR4/HLA-DMA/HLA-DMB/HLA-DOB/ICOSLG/IL15RA/ITGB7/TGFB1/TNFSF13	13
hsa04015	Rap1 signaling pathway	37/874	210/8101	0.00170668982044931	0.010675177504379	0.00697470762536561	ADORA2A/APBB1IP/CALM3/CALML4/CSF1R/EVL/FLT1/FPR1/FYB/INSR/ITGAL/ITGAM/ITGB2/ITGB3/MAP2K6/MAPK13/MAPK3/MET/MLLT4/NGFR/PDGFC/PIK3CD/PIK3R2/PLCB2/PLCB4/PRKCA/PRKCB/RAC2/	37

							RALA/RAPGEF2/RAPGEF5/RGS14/RRAS/SRC/VASP/VAV1/VAV2	
hsa04725	Cholinergic synapse	23/874	113/8101	0.0018950505292956	0.0116254061316403	0.00759554665588116	ACHE/BCL2/CAMK2A/CAMK2G/CREB3L4/CREB5/FOS/GNA11/GNGT2/ITPR2/KCNJ2/KCNQ1/MAPK3/PIK3CD/PIK3CG/PIK3R2/PIK3R5/PIK3R6/PLCB2/PLCB4/PRKACB/PRKCA/PRKCB	23
hsa04152	AMPK signaling pathway	24/874	120/8101	0.0019584267710393	0.011787512074746	0.00770145979475236	ACACA/ACACB/ADIPOR1/AKT1S1/CCNA1/CCNA2/CD36/CREB3L4/CREB5/EIF4EBP1/FASN/HMGCR/INSR/IRS2/LEPR/PCK2/PFKFB2/PFKFB4/PIK3CD/PIK3R2/PPP2R1B/PPP2R3B/SCD/ULK1	24
hsa04670	Leukocyte transendothelial migration	23/874	114/8101	0.00213845891548459	0.0126327480377701	0.00825370107730894	ACTN1/CLDN23/CLDN7/CXCR4/ITGAL/ITGAM/ITGB2/MAPK13/MLLT4/MMP2/MMP9/MYL9/NCF2/PIK3CD/PIK3R2/PRKCA/PRKCB/PTK2/RAC2/TXK/VASP/VAV1/VAV2	23
hsa05031	Amphetamine addiction	16/874	69/8101	0.00227854152392751	0.0132155408387796	0.00863447314330426	CALM3/CALML4/CAMK2A/CAMK2G/CREB3L4/CREB5/FOS/GRIN3A/MAOA/PPP1CB/PPP3CA/PPP3CC/PRKACB/PRKCA/PRKCB/STX1A	16
hsa04926	Relaxin signaling pathway	25/874	129/8101	0.00250876530197384	0.0142275543330533	0.00929567978203119	ACTA2/ARRB2/COL1A2/COL4A2/CREB3L4/CREB5/EDN1/EDNRB/FOS/GNGT2/MAPK13/MAPK3/MMP2/MMP9/NFKB1/NFKBIA/PIK3CD/PIK3R2/PLCB2/PLCB4/PRKACB/PRKCA/SRC/TGFB1/TGFBR2	25
hsa04971	Gastric acid secretion	17/874	76/8101	0.00254222757675247	0.0142275543330533	0.00929567978203119	CALM3/CALML4/CAMK2A/CAMK2G/HRH2/ITPR2/KCNJ10/KCNJ15/KCNJ2/KCNQ1/MYLK/PLCB2/PLCB4/PRKACB/PRKCA/PRKCB/SSTR2	17
hsa05150	Staphylococcus aureus infection	20/874	96/8101	0.00274167458228307	0.0150792102025569	0.00985211555883933	C1QA/C5AR1/CFB/CFH/FCAR/FCGR1A/FCGR2A/FCGR2B/FCGR2C/FCGR3A/FCGR3B/FPR1/FPR2/HLA-DMA/HLA-DMB/HLA-DOB/ITGAL/ITGAM/ITGB2/SELPLG	20

hsa04110	Cell cycle	24/874	124/8101	0.0030835581652242 6	0.0165525264258154	0.0108147178099701	BUB1/BUB1B/CCNA1/CCNA2/CCNB1/CCNB2/CCND3/CDC14A/CDC25B/CDK1/CDK6/CDKN1A/CDKN1C/CDKN2B/CDKN2C/CDKN2D/E2F2/GADD45B/GADD45G/MYC/PLK1/TFDP1/TFDP2/TGFB1	24
hsa04666	Fc gamma R-mediated phagocytosis	20/874	97/8101	0.00311332785438534	0.0165525264258154	0.0108147178099701	ASAP2/FCGR1A/FCGR2A/FCGR2B/FCGR3A/FCGR3B/MAPK3/MARCKSL1/MYO10/PIK3CD/PIK3R2/PRKCA/PRKCB/RAC2/SCIN/SPHK1/SYK/VASP/VAV1/VAV2	20
hsa04621	NOD-like receptor signaling pathway	32/874	181/8101	0.00321648331740755	0.0168206258730001	0.0109898826030491	BCL2/BIRC3/CASP1/GBP1/GBP2/GBP4/GBP7/GSDMD/HSP90AA1/HSP90AB1/ITPR2/MAPK13/MAPK3/MEFV/NAIP/NFKB1/NFKBIA/NLRC4/NLRP1/P2RX7/PLCB2/PLCB4/PSTPIP1/PYCARD/RIPK2/TLR4/TMEM173/TNF/TNFAIP3/TRAF5/TXN/TXNIP	32
hsa04022	cGMP-PKG signaling pathway	30/874	167/8101	0.00333686121595741	0.0171686891595228	0.011217292372828	ADORA3/ATP2A3/ATP2B1/ATP2B4/BAD/CALM3/CALML4/CREB3L4/CREB5/EDNRB/GNA11/GUCY1A3/INSR/IRS2/ITPR2/MAPK3/MEF2D/MYL9/MYLK/PIK3CG/PIK3R5/PIK3R6/PLCB2/PLCB4/PPP1CB/PPP3CA/PPP3CC/RGS2/SLC8A3/VASP	30
hsa04925	Aldosterone synthesis and secretion	20/874	98/8101	0.00352619854542851	0.0175829099182728	0.0114879266253688	ATP2B1/ATP2B4/CALM3/CALML4/CAMK2A/CAMK2G/CREB3L4/CREB5/DAGLA/GNA11/ITPR2/KCNJ5/LDLR/NR4A2/PLCB2/PLCB4/PRKACB/PRKCA/PRKCB/SCARB1	20
hsa05223	Non-small cell lung cancer	16/874	72/8101	0.00360253552489768	0.0175829099182728	0.0114879266253688	ALK/BAD/CDK6/CDKN1A/E2F2/EML4/GADD45B/GADD45G/MAPK3/MET/PIK3CD/PIK3R2/PRKCA/PRKCB/RXRA/TGFA	16
hsa04610	Complement and coagulation cascades	18/874	85/8101	0.00363635703042258	0.0175829099182728	0.0114879266253688	C1QA/C5AR1/CFB/CFH/CR1/CR1L/F13A1/F8/ITGAM/ITGAX/ITGB2/PROS1/SERPINB2/SERPINE1/TFPI/THBD/VSIG4/VTN	18

hsa05130	Pathogenic Escherichia coli infection	34/874	197/8101	0.0036378434313667 8	0.0175829099182728	0.0114879266253688	CASP1/CASP3/CASP7/CLDN23/CLDN7/CYCS/CYFIP2/CYTH3/FCGR2A/FOS/IL1R1/MAPK13/MAPK3/MYH11/MYO10/MYO1A/MYO1E/MYO1F/MYO1G/NAIP/NFKB1/NFKBIA/PTPN6/PYCARD/SLC9A3R1/SRC/TJP1/TLR4/TLR5/TNF/TUBA1B/TUBA1C/TUBB/TUBB4B	34
hsa04921	Oxytocin signaling pathway	28/874	154/8101	0.00376704463140598	0.0177873788681941	0.011621517953811	CACNA2D3/CACNA2D4/CALM3/CALML4/CAMK2A/CAMK2G/CDKN1A/FOS/GUCY1A3/ITPR2/KCNJ2/KCNJ5/MAPK3/MYL9/MYLK/PIK3CG/PIK3R5/PIK3R6/PLCB2/PLCB4/PPP1CB/PPP3CA/PPP3CC/PRKACB/PRKCA/PRKCB/RGS2/SRC	28
hsa04071	Sphingolipid signaling pathway	23/874	119/8101	0.00379166696876865	0.0177873788681941	0.011621517953811	ADORA3/BCL2/CERS4/CERS6/MAP3K5/MAPK13/MAPK3/NFKB1/PIK3CD/PIK3R2/PLCB2/PLCB4/PPP2R1B/PPP2R3B/PRKCA/PRKCB/RAC2/S1PR4/SGMS2/SGPL1/SGPP2/SPHK1/TNF	23
hsa04115	p53 signaling pathway	16/874	73/8101	0.00416408721001113	0.0192513597100515	0.0125780208631915	ADGRB1/BCL2/CASP3/CCNB1/CCNB2/CCND3/CDK1/CDK6/CDKN1A/CYCS/GADD45B/GADD45G/PMAIP1/SERPINE1/SESN3/STEAP3	16
hsa04912	GnRH signaling pathway	19/874	93/8101	0.00434203310447796	0.0197872651475496	0.0129281587170923	CALM3/CALML4/CAMK2A/CAMK2G/EGR1/GNA11/HBEGF/ITPR2/MAP2K6/MAP3K3/MAPK13/MAPK3/MMP2/PLCB2/PLCB4/PRKACB/PRKCA/PRKCB/SRC	19
hsa04931	Insulin resistance	21/874	108/8101	0.00514227777609139	0.0231040367686359	0.0150951964368583	ACACB/CD36/CREB3L4/CREB5/INSR/IRS2/NFKB1/NFKBIA/PCK2/PIK3CD/PIK3R2/PPP1CB/PPP1R3B/PPP1R3E/PRKCB/PYGL/SLC27A1/SLC27A3/SLC2A1/TNF/TRIB3	21
hsa04540	Gap junction	18/874	88/8101	0.00535036901411817	0.0237051071597736	0.0154879103040263	CDK1/GNA11/GUCY1A3/HTR2B/ITPR2/MAPK3/PDGFC/PLCB2/PLCB4/PRKACB/PRKCA/PRKCB/SRC/TJP1/TUBA1B/TUBA1C/TUBB/TUBB4B	18

hsa05142	Chagas disease	20/874	102/8101	0.0056608412816303 5	0.0246804389797982	0.016125150694605	ACE/C1QA/CCL3/FOS/GNA11/MAPK13/MAPK3/NFKB1/NFKBIA/PIK3CD/PIK3R2/PLCB2/PLCB4/PPP2R1B/SERPINE1/TGFB1/TGFBR2/TLR4/TLR6/TNF	20
hsa04066	HIF-1 signaling pathway	21/874	109/8101	0.0057418319174621	0.0246804389797982	0.016125150694605	BCL2/CAMK2A/CAMK2G/CDKN1A/EDN1/EGLN1/EIF4EBP1/ENO2/FLT1/HK3/INSR/MAPK3/NFKB1/PDK1/PIK3CD/PIK3R2/PRKCA/PRKCB/SERPINE1/SLC2A1/TLR4	21
hsa04979	Cholesterol metabolism	12/874	50/8101	0.0058026110454071 1	0.0246804389797982	0.016125150694605	ABCA1/APOC2/CD36/CYP27A1/LDLR/LDLRAP1/LPL/LRP1/PLTP/SCARB1/SOAT1/SORT1	12
hsa05161	Hepatitis B	28/874	162/8101	0.0077128473011062 9	0.0323736616980646	0.0211515757010948	BAD/BCL2/BIRC5/CASP3/CCNA1/CCNA2/CDKN1A/CREB3L4/CREB5/CYCS/E2F2/FOS/MAP2K6/MAPK13/MAPK3/MMP9/MYC/NFKB1/NFKBIA/PIK3CD/PIK3R2/PRKCA/PRKCB/SRC/TGFB1/TGFBR2/TLR4/TNF	28
hsa05165	Human papillomavirus infection	50/874	331/8101	0.0082320667030202	0.0341042763410837	0.0222822858126863	ATP6V0A1/ATP6V0D2/ATP6V0E2/BAD/CASP3/CCNA1/CCNA2/CCND3/CDK6/CDKN1A/COL1A2/COL4A2/COL6A1/COL6A2/COL9A2/CREB3L4/CREB5/EIF4EBP1/FN1/HES4/IRF1/ITGA1/ITGB3/ITGB5/ITGB7/ITGB8/JAG1/LAMA3/LAMB1/LAMB3/LAMC1/MAML3/MAPK3/NFKB1/PIK3CD/PIK3R2/PPP2R1B/PPP2R3B/PRKACB/PTGER4/PTK2/SLC9A3R1/SPP1/TCF7L1/TCF7L2/TNC/TNF/TUBG1/VTN/WNT5A	50
hsa04658	Th1 and Th2 cell differentiation	18/874	92/8101	0.0086096768070364 9	0.0352113705313415	0.0230056141402594	CD4/DLL1/FOS/HLA-DMA/HLA-DMB/HLA-DOB/JAG1/MAF/MAML3/MAPK13/MAPK3/NFKB1/NFKBIA/NFKBIE/PPP3CA/PPP3CC/RUNX3/TBX21	18
hsa04370	VEGF signaling pathway	13/874	59/8101	0.0089172146	0.0360074871727394	0.0235257629440766	BAD/MAPK13/MAPK3/PIK3CD/PIK3R2/PPP3CA/PPP3CC/PRKCA/PRKCB/PTK2/RAC2/SPHK1/SRC	13

				9168155				
hsa04010	MAPK signaling pathway	45/874	294/8101	0.00938486204299807	0.0374221373964548	0.0244500353225476	ARRB2/CACNA2D3/CACNA2D4/CASP3/CD14/CDC25B/CSF1R/DUSP2/DUSP5/DUSP6/FLT1/FOS/GADD45B/GADD45G/HSPA2/HSPA6/IL1A/IL1R1/INSR/MAP2K6/MAP3K13/MAP3K3/MAP3K5/MAPK13/MAPK3/MET/MYC/NFKB1/NFKB2/NGFR/PDGFC/PPP3CA/PPP3CC/PRKACB/PRKCA/PRKCB/RAC2/RAPGEF2/RASGRP1/RASGRP4/RRAS/TGFA/TGFB1/TGFBR2/TNF	45
hsa04261	Adrenergic signaling in cardiomyocytes	26/874	150/8101	0.00964892438907001	0.0380000849396708	0.0248276417028702	ATP2A3/ATP2B1/ATP2B4/BCL2/CACNA2D3/CACNA2D4/CALM3/CALML4/CAMK2A/CAMK2G/CREB3L4/CREB5/KCNQ1/MAPK13/MAPK3/PIK3CG/PIK3R5/PIK3R6/PLCB2/PLCB4/PPP1CB/PPP2R1B/PPP2R3B/PRKACB/PRKCA/SLC8A3	26
hsa05416	Viral myocarditis	13/874	60/8101	0.0102926997558101	0.0400411124646761	0.0261611624088628	CASP3/CD40/CD80/CD86/CYCS/EIF4G3/HLA-DMA/HLA-DMB/HLA-DOB/ITGAL/ITGB2/RAC2/SGCG	13
hsa04720	Long-term potentiation	14/874	67/8101	0.0109384325879069	0.0420404818740035	0.0274674654712182	CALM3/CALML4/CAMK2A/CAMK2G/ITPR2/MAPK3/PLCB2/PLCB4/PPP1CB/PPP3CA/PPP3CC/PRKACB/PRKCA/PRKCB	14
hsa05170	Human immunodeficiency virus 1 infection	34/874	212/8101	0.0113825266161579	0.0432264998875519	0.0282423592731736	AP1S3/BAD/BCL2/CALM3/CALML4/CASP3/CCNB1/CCNB2/CD4/CDK1/CXCR4/CYCS/FOS/GNA11/GNGT2/ITPR2/MAP2K6/MAPK13/MAPK3/MB21D1/NFKB1/NFKBIA/PIK3CD/PIK3R2/PPP3CA/PPP3CC/PRKCA/PRKCB/PTK2/RAC2/TLR4/TMEM173/TNF/TRAF5	34
hsa04360	Axon guidance	30/874	182/8101	0.0116183663014529	0.0436030452960408	0.0284883780518597	ABLIM3/CAMK2A/CAMK2G/CXCR4/EPHB6/FES/MAPK3/MET/MYL9/NRP1/NTN1/PDK1/PIK3CD/PIK3R2/PLXNB2/PPP3CA/PPP3CC/PRKCA/PTK2/RAC2/RGS3/RRAS/SE	30

							MA4B/SEMA4D/SEMA6B/SLIT2/SRC/SRGAP1/SSH2/WNT5A	
hsa04915	Estrogen signaling pathway	24/874	138/8101	0.012080222337017	0.0448091968082377	0.0292764262267977	BCL2/CALM3/CALML4/CREB3L4/CREB5/FOS/HBEGF/HSP90AA1/HSP90AB1/HSPA2/HSPA6/ITPR2/KCNJ5/MAPK3/MMP2/MMP9/PIK3CD/PIK3R2/PLCB2/PLCB4/PRKACB/RARA/SRC/TGFA	24
hsa05214	Glioma	15/874	75/8101	0.0128780123625032	0.0472193786625118	0.0308511366942001	CALM3/CALML4/CAMK2A/CAMK2G/CDK6/CDKN1A/E2F2/GADD45B/GADD45G/MAPK3/PIK3CD/PIK3R2/PRKCA/PRKCB/TGFA	15
hsa04728	Dopaminergic synapse	23/874	132/8101	0.0135213502044846	0.0490148944912565	0.0320242504843055	ARRB2/CALM3/CALML4/CAMK2A/CAMK2G/CREB3L4/CREB5/FOS/GNGT2/ITPR2/KCNJ5/MAOA/MAPK13/PLCB2/PLCB4/PPP1CB/PPP2R1B/PPP2R3B/PPP3CA/PPP3CC/PRKACB/PR	23
hsa03320	PPAR signaling pathway	15/874	76/8101	0.014497568244312	0.0513858252215058	0.0335733159341962	CD36/CYP27A1/FABP5/FADS2/HMGCS1/LPL/ME1/OLR1/PCK2/PLIN2/PLTP/PPARD/RXRA/SCD/SLC27A1	15
hsa05212	Pancreatic cancer	15/874	76/8101	0.014497568244312	0.0513858252215058	0.0335733159341962	BAD/CDK6/CDKN1A/E2F2/GADD45B/GADD45G/MAPK3/NFKB1/PIK3CD/PIK3R2/RAC2/RALA/TGFA/TGFB1/TGFBR2	15
hsa05167	Kaposi sarcoma-associated herpesvirus infection	31/874	193/8101	0.0148902914338542	0.0521978348065879	0.0341038485124712	CALM3/CALML4/CASP3/CD200R1/CD86/CDK6/CDKN1A/CLEC2B/CYCS/E2F2/FOS/GNGT2/ITPR2/MAP2K6/MAPK13/MAPK3/MYC/NFKB1/NFKBIA/PIK3CD/PIK3CG/PIK3R2/PIK3R5/	31
hsa04722	Neurotrophin signaling pathway	21/874	119/8101	0.0155056475874941	0.053764147613159	0.0351272107817373	ARHGDIB/BAD/BCL2/CALM3/CALML4/CAMK2A/CAMK2G/IRAK2/MAP3K3/MAP3K5/MAPK13/MAPK3/NFKB1/NFKBIA/NFKBIE/NGFR/NGFRAP1/PIK3CD/PIK3R2/RIPK2/SORT1	21

hsa05144	Malaria	11/874	50/8101	0.0156915677173429	0.0538237645358322	0.0351661619471862	CD36/CD40/CR1/CR1L/ITGAL/ITGB2/LRP1/MET/TGFB1/TLR4/TNF	11
hsa01522	Endocrine resistance	18/874	98/8101	0.0163017551166837	0.0548537368714716	0.0358391021301811	BAD/BCL2/CDKN1A/CDKN2C/DLL1/E2F2/FOS/HBEGF/JAG1/MAPK13/MAPK3/MMP2/MMP9/PIK3CD/PIK3R2/PRKACB/PTK2/SRC	18
hsa05169	Epstein-Barr virus infection	32/874	202/8101	0.0163357523598426	0.0548537368714716	0.0358391021301811	BCL2/BCL2L11/CASP3/CCNA1/CCNA2/CCND3/CD40/CDK6/CDKN1A/CXCL10/CYCS/E2F2/GADD45B/GADD45G/HLA-DMA/HLA-DMB/HLA-DOB/ITGAL/MAP2K6/MAPK13/MYC/NFKB1/NFKB2/NFKBIA/NFKBIE/PIK3CD/PIK3R2/RUNX3/SYK/TNF/TNFAIP3/TRAF5	32
hsa00072	Synthesis and degradation of ketone bodies	4/874	10/1/8101	0.0165823480955648	0.0551017608592205	0.0360011504706341	ACAT2/BDH1/HMGCS1/OXCT1	4
hsa05321	Inflammatory bowel disease	13/874	65/8101	0.0197982787529766	0.0651098033216448	0.0425399803916372	HLA-DMA/HLA-DMB/HLA-DOB/IL1A/IL21R/MAF/NFKB1/RORA/TBX21/TGFB1/TLR4/TLR5/TNF	13
hsa05135	Yersinia infection	23/874	137/8101	0.0205059659387554	0.0667490115761529	0.043610969450844	CASP1/CD4/FCGR2A/FN1/FOS/FYB/MAP2K6/MAPK13/MAPK3/MEFV/NFKB1/NFKBIA/NLRC4/PIK3CD/PIK3R2/PTK2/PYCARD/RAC2/SRC/TLR4/TNF/VAV1/VAV2	23
hsa05131	Shigellosis	37/874	246/8101	0.0223824471306092	0.072121218531963	0.0471209413275983	ACTN1/AKT1S1/BCL2/BNIP3/CASP1/CD14/CYCS/CYTH3/FOXO4/HK3/IL1R1/ITPR2/MAPK13/MAPK3/MB21D1/MYL9/NAIP/NFKB1/NFKBIA/NLRC4/PIK3CD/PIK3R2/PLCB2/PLCB4/PTK2/PYCARD/RIPK2/SEPT11/SEPT3/SEPT9/SRC/TECPR1/TLR4/TLR5/TMEM173/TNF/TRAF5	37

hsa04020	Calcium signaling pathway	36/874	240/8101	0.024948472470559	0.0795856271810833	0.0519978689386388	ADORA2A/ATP2A3/ATP2B1/ATP2B4/CALM3/CALML4/CAMK2A/CAMK2G/CXCR4/CYSLTR1/CYSLTR2/EDNRB/FLT1/GNA11/HRH2/HTR2B/HTR7/ITPR2/MCOLN2/MET/MST1/MYLK/ORAI3/P2RX1/P2RX7/PDGFC/PLCB2/PLCB4/PPP3CA/PPP3CC/PRKACB/PRKCA/PRKCB/SLC8A3/SPHK1/TPCN1	36
hsa00650	Butanoate metabolism	7/874	28/8101	0.0259202041278939	0.0818667833346352	0.0534882794926836	AACS/ACAT2/BDH1/HADH/HMGCS1/L2HGDH/OXCT1	7
hsa04664	Fc epsilon RI signaling pathway	13/874	68/8101	0.0280192201028761	0.0876287373805635	0.0572528955662484	ALOX5/ALOX5AP/MAP2K6/MAPK13/MAPK3/PIK3CD/PIK3R2/PRKCA/RAC2/SYK/TNF/VAV1/VAV2	13
hsa05220	Chronic myeloid leukemia	14/874	76/8101	0.0310398796870576	0.095841455303723	0.0626187366775686	BAD/CDK6/CDKN1A/E2F2/GADD45B/GADD45G/MAPK3/MYC/NFKB1/NFKBIA/PIK3CD/PIK3R2/TGFB1/TGFBR2	14
hsa04924	Renin secretion	13/874	69/8101	0.031246117089615	0.095841455303723	0.0626187366775686	ACE/CALM3/CALML4/EDN1/GUCY1A3/ITPR2/KCNJ2/PLCB2/PLCB4/PPP3CA/PPP3CC/PRKACB/PTGER4	13
hsa04310	Wnt signaling pathway	26/874	166/8101	0.0322306455731794	0.0979197708366117	0.0639766197843561	CAMK2A/CAMK2G/CCND3/CTNNBIP1/FOSL1/FRAT1/FRAT2/GPC4/LGR4/MYC/PLCB2/PLCB4/PORCN/PPARD/PPP3CA/PPP3CC/PRKACB/PRKCA/PRKCB/RAC2/SERPINF1/TCF7L1/TCF7L2/TLE3/VANGL1/WNT5A	26
hsa04371	Apelin signaling pathway	22/874	137/8101	0.0362577551510194	0.106485927978663	0.0695733830713585	ACTA2/CALM3/CALML4/EGR1/GNGT2/HDAC5/ITPR2/JAG1/MAPK3/MEF2D/MYLK/PIK3CG/PIK3R5/PIK3R6/PLCB2/PLCB4/PRKACB/RRAS/SERPINE1/SLC8A3/SPHK1/SPP1	22
hsa04910	Insulin signaling pathway	22/874	137/8101	0.0362577551510194	0.106485927978663	0.0695733830713585	ACACA/ACACB/BAD/CALM3/CALML4/EIF4EBP1/FASN/HK3/INSR/IRS2/MAPK3/PCK2/PIK3CD/PIK3R2/PPP1CB/PPP1R3B/PPP1R3E/PRKACB/PRKAR2B/PYGL/SOCS2/TRIP10	22

hsa05225	Hepatocellular carcinoma	26/874	168/8101	0.0367165315275466	0.106485927978663	0.0695733830713585	BAD/CDK6/CDKN1A/E2F2/FRAT1/FRAT2/GADD45B/GADD45G/GSTM4/MAPK3/MET/MGST1/MYC/PHF10/PIK3CD/PIK3R2/PRKCA/PRKCB/SMARCA2/SMARCD3/TCF7L1/TCF7L2/TGFA/TGFB1/TGFBR2/WNT5A	26
hsa04922	Glucagon signaling pathway	18/874	107/8101	0.0367291958774964	0.106485927978663	0.0695733830713585	ACACA/ACACB/CALM3/CALML4/CAMK2A/CAMK2G/CREB3L4/CREB5/ITPR2/PCK2/PLCB2/PLCB4/PPP3CA/PPP3CC/PRKACB/PRMT1/PYGL/SLC2A1	18
hsa04012	ErbB signaling pathway	15/874	85/8101	0.037007440125216	0.106485927978663	0.0695733830713585	BAD/CAMK2A/CAMK2G/CDKN1A/EIF4EBP1/HBEGF/MAPK3/MYC/PIK3CD/PIK3R2/PRKCA/PRKCB/PTK2/SRC/TGFA	15
hsa04940	Type I diabetes mellitus	9/874	43/8101	0.0370530971963371	0.106485927978663	0.0695733830713585	CD80/CD86/GZMB/HLA-DMA/HLA-DMB/HLA-DOB/HSPD1/IL1A/TNF	9
hsa04914	Progesterone-mediated oocyte maturation	17/874	100/8101	0.0380583128442443	0.10839823033316	0.0708228002176727	AURKA/BUB1/CCNA1/CCNA2/CCNB1/CCNB2/CDC25B/CDK1/CPEB2/HSP90AA1/HSP90AB1/MAPK13/MAPK3/PIK3CD/PIK3R2/PLK1/PRKACB	17
hsa04024	cAMP signaling pathway	32/874	216/8101	0.0385102468736241	0.108714767722886	0.0710296123053337	ABCC4/ADORA2A/ATP2A3/ATP2B1/ATP2B4/BAD/CALM3/CALML4/CAMK2A/CAMK2G/CREB3L4/CREB5/EDN1/FOS/GRIN3A/MAPK3/MLLT4/MYL9/NFKB1/NFKBIA/PDE4A/PDE4B/PIK3CD/PIK3R2/PPP1CB/PRKACB/RAC2/RRAS/SSTR2/SUCNR1/VAV1/VAV2	32
hsa04929	GnRH secretion	12/874	64/8101	0.0388612280819841	0.108743261036429	0.0710482286263421	ARRB2/GNA11/ITPR2/KCNJ5/MAPK3/PIK3CD/PIK3R2/PLCB2/PLCB4/PRKCA/PRKCB/SPP1	12
hsa04960	Aldosterone-regulated sodium	8/874	37/8101	0.0403355384466718	0.110922730728348	0.0724722015648006	INSR/MAPK3/NR3C2/PIK3CD/PIK3R2/PRKCA/PRKCB/SGK1	8

	reabsorption							
hsa05216	Thyroid cancer	8/874	37/8101	0.0403355384466718	0.110922730728348	0.0724722015648006	CDKN1A/GADD45B/GADD45G/MAPK3/MYC/RXRA/TCF7L1/TCF7L2	8
hsa04916	Melanogenesis	17/874	101/8101	0.0413568471263236	0.112527998644354	0.0735210154482167	CALM3/CALML4/CAMK2A/CAMK2G/CREB3L4/EDN1/EDNRB/MAPK3/MC1R/PLCB2/PLCB4/PRKACB/PRKCA/PRKCB/TCF7L1/TCF7L2/WNT5A	17
hsa01521	EGFR tyrosine kinase inhibitor resistance	14/874	79/8101	0.0416247769280055	0.112527998644354	0.0735210154482167	BAD/BCL2/BCL2L11/EIF4EBP1/GAS6/MAPK3/MET/PDGFC/PIK3CD/PIK3R2/PRKCA/PRKCB/SRC/TGFA	14
hsa01523	Antifolate resistance	7/874	31/8101	0.0434015351475087	0.116345291697944	0.0760150726157163	ABCC4/GART/NFKB1/SHMT1/SLC46A1/TNF/TYMS	7
hsa05330	Allograft rejection	8/874	38/8101	0.0464537982863838	0.123489680444637	0.0806829128131929	CD40/CD80/CD86/GZMB/HLA-DMA/HLA-DMB/HLA-DOB/TNF	8
hsa04978	Mineral absorption	11/874	59/8101	0.0484408481268866	0.127707690516337	0.0834387814625798	ATP2B1/ATP2B4/CYBRD1/MT1G/MT1H/MT1M/MT1X/SLC31A1/SLC46A1/SLC8A3/VDR	11

IFNL3 VS NT M2-MDM Reactome enrichment

PATH_ID	Description	GeneRatio	BgRatio	pvalue	p.adjust	qvalue	geneID	Count
5620924	Intraflagellar transport	17/801	37/6750	2.45749658955555E-07	0.000148190178516302	0.000140569247598573	IFT57/IFT52/IFT172/IFT140/DYNC2H1/WDR19/IFT20/TTC30B/IFT81/WDR35/TRAF3IP1/IFT22/IFT46/TTC26/KIF3C/DYNC2LI1/CLUAP1	17

191273	Cholesterol biosynthesis	13/801	23/6750	3.0840827995068E-07	0.000148190178516302	0.000140569247598573	HMGCS1/FDFT1/HMGCR/MVD/IDI1/FDPS/MSMO1/SQLE/CYP51A1/HSD17B7/EBP/MVK/ARV1	13
5617833	Assembly of the primary cilium	39/801	163/6750	0.0000104189144322104	0.003337525589784 73	0.003165887683612	UNC119B/LZTFL1/BBS1/BBS9/IFT57/HSP90AA1/TUBB/IFT52/IFT172/CEP131/TTC8/IFT140/DYNC2H1/WDR19/IFT20/TTC30B/IFT81/ARL3/KIF24/TUBB4B/CEP83/WDR35/CDK5RAP2/RPGRIP1L/TUBG1/TRAF3IP1/IFT22/CENPJ/BBIP1/IFT46/TMEM67/TTC26/BBS5/TCTN2/KIF3C/DYNC2LI1/NPHP4/CLUAP1/B9D2	39
2426168	Activation of gene expression by SREBF (SREBP)	12/801	26/6750	0.0000142112549058389	0.003414253991127 8	0.00323867019696224	HMGCS1/FDFT1/HMGCR/MVD/IDI1/FDPS/SQLE/CYP51A1/FASN/ACACA/ACACB/MVK	12
3000170	Syndecan interactions	11/801	25/6750	0.0000565867131877839	0.010875966274692 1	0.0103166512885518	ITGB5/VTN/ITGB3/SDC4/SDC2/TGFB1/FN1/TNC/COL5A2/SDC3/PRKCA	11
380108	Chemokine receptors bind chemokines	17/801	56/6750	0.000169190782080069	0.027098723596491	0.02570512583883150.025705125838 83150.025705125838831 5	PPBP/CXCL5/CCL19/CCL7/CXCL16/CXCL9/CCR7/CXCR4/CCRL2/CCL22/CXCL13/CXCL10/CXCL11/CCL4/CXCL1/CCL3/CXCL6	17
622312	Inflammasomes	8/801	17/6750	0.000345061541359242	0.042621768602574 1	0.0404298719643235	NLRC4/NLRP1/PSTPIP1/HSP90AB1/TXNIP/CASP1/P2RX7/TXN	8
1655829	Regulation of cholesterol biosynthesis by SREBP (SREBF)	13/801	39/6750	0.000354811809386673	0.042621768602574 1	0.0404298719643235	HMGCS1/FDFT1/HMGCR/MVD/IDI1/FDPS/SQLE/CYP51A1/FASN/INSIG1/ACACA/ACACB/MVK	13

204998	Cell death signalling via NRAGE; NRIF and NADE	17/801	61/6750	0.000522365618259331	0.0557770399052464	0.0529086111593662	FGD4/VAV2/AKAP13/PSENEN/NET1/TRAF6/TIAM2/CASP3/ARHGAP4/UBB/ITSN1/VAV1/BAD/BCL2L11/ARHGEF3/KALRN/NGFRAP1	17
2022928	HS-GAG biosynthesis	10/801	27/6750	0.000663113125572875	0.0637251713675533	0.0604479964995905	NDST1/GPC4/EXT1/SDC4/SDC2/GLCE/HS3ST3B1/HS2ST1/HS3ST1/SDC3	10
5362517	Signaling by Retinoic Acid	13/801	42/6750	0.000794434595180593	0.06940469508805	0.0658354410934348	DHRS3/RXRA/RARA/PDK2/PDK4/PPARD/PDK3/FABP5/ALDH1A2/PDK1/AKR1C3/CRABP1/DHRS9	13
168898	Toll-Like Receptors Cascades	25/801	111/6750	0.00103054005567532	0.0807545098270639	0.0766015723864805	FOS/BIRC3/CD14/TLR4/DUSP6/TLR6/CNPY3/ITGAM/NFKB2/MAPK3/CD36/TRAF6/TLR7/MAP3K1/TLR5/UBB/ITGB2/LY96/NFKBIA/PELI2/NOD1/CTSK/PELI1/LBP/RIPK2	25
168643	Nucleotide-binding domain; leucine rich repeat containing receptor (NLR) signaling pathways	13/801	44/6750	0.00129013615177374	0.0807545098270639	0.0766015723864805	NLRC4/NLRP1/PSTPIP1/HSP90AB1/TXNIP/TRAF6/CASP1/UBB/P2RX7/TNFAIP3/NOD1/TXN/RIPK2	13
1474244	Extracellular matrix organization	46/801	249/6750	0.00129756415269724	0.0807545098270639	0.0766015723864805	MMP10/ITGB5/FURIN/VTN/ITGB3/TIMP2/SPP1/ITGAM/SPARC/ITGAX/SDC4/ITGAL/ADAMTS4/MMP9/SDC2/PECAM1/COLGALT2/BMP2/VCAN/ITGB7/ADAM8/CASP3/MMP8/TGFB1/ITGB2/FN1/ITGA1/ITGA9/COL6A2/PLOD1/PLEC/LTBP3/TNC/LAMB3/COL6A1/CTSK/COL5A2/SDC3/LAMB1/LUM/PR	46

							KCA/SERPINE1/MMP2/LAMA3/LAMC1/COL23A1	
5620922	BBSome-mediated cargo-targeting to cilium	6/801	12/1/6750	0.00134450796798442	0.08075450982706 39	0.0766015723864805	LZTFL1/BBS1/BBS9/TTC8/BBIP1/BBS5	6
844456	The NLRP3 inflammasome	6/801	12/1/6750	0.00134450796798442	0.08075450982706 39	0.0766015723864805	PSTPIP1/HSP90AB1/TXNIP/CASP1/P2RX7/TXN	6
3000178	ECM proteoglycans	17/801	67/6750	0.00165219260646627	0.09339747616553 42	0.0885943527677886	ITGB5/VTN/ITGB3/SPARC/ITGAX/VCAN/TGFB1/FN1/ITGA9/COL6A2/TNC/COL6A1/COL5A2/LAMB1/SERPINE1/LAMA3/LAMC1	17
1630316	Glycosaminoglycan metabolism	25/801	116/6750	0.00199087206079228	0.106290447245632	0.100824280973457	B3GNT7/ST3GAL6/NDST1/GPC4/B4GAT1/EXT1/SDC4/HGSNAT/SDC2/GLCE/B4GALT5/SGSH/VCAN/HS3ST3B1/HS2ST1/CHST15/PAPSS2/B3GNT2/ST3GAL4/CHST2/HS3ST1/SDC3/LUM/CHST14/ST3GAL3	25
379724	tRNA Aminoacylation	12/801	42/6750	0.00268929218622262	0.126791650791439	0.120271175404334	AIMP2/HARS/KARS/FARSB/PPA1/VARS/EARS2/WARS/AARS2/MARS2/YARS2/WARS2	12
194840	Rho GTPase cycle	26/801	125/6750	0.00276685785138477	0.126791650791439	0.120271175404334	TRIP10/FGD4/ARHGAP6/FAM13A/VAV2/ARHGAP9/AKAP13/ARHGDIB/ARHGAP26/NET1/RAC2/TIAM2/SRGAP1/PIK3R2/ARHGAP4/ITSN1/VAV1/ARHGAP20/ARHGEF3/KALRN/RHOBTB2/ARHGAP18/ARHGAP11A/ARHGAP24/ARHGAP22/RHOF	26
3000171	Non-integrin membrane-ECM interactions	14/801	53/6750	0.00277068123477651	0.126791650791439	0.120271175404334	ITGB5/VTN/ITGB3/SDC4/SDC2/TGFB1/FN1/TNC/COL5A2/SDC3/LAMB1/PRKCA/LAMA3/LAMC1	14

216083	Integrin cell surface interactions	19/801	83/6750	0.00327686549121145	0.132863284857309	0.126030565404929	ITGB5/VTN/ITGB3/SPP1/ITGAM/ITGAX/ITGAL/PECAM1/ITGB7/ITGB2/FN1/ITGA1/ITGA9/COL6A2/TNC/COL6A1/COL5A2/LUM/COL23A1	19
166016	Toll Like Receptor 4 (TLR4) Cascade	21/801	96/6750	0.00366494521186792	0.132863284857309	0.126030565404929	FOS/BIRC3/CD14/TLR4/DUSP6/TLR6/ITGAM/NFKB2/MAPK3/CD36/TRAF6/MAP3K1/UBB/ITGB2/LY96/NFKBIA/PELI2/NOD1/PELI1/LBP/RIPK2	21
1852241	Organelle biogenesis and maintenance	49/801	283/6750	0.00371503866347329	0.132863284857309	0.126030565404929	PPRC1/UNC119B/MRPS28/LZTFL1/BBS1/BBS9/MRPL32/IFT57/HSP90AA1/GFM1/CYCS/ALAS1/TUBB/IFT52/MRPS27/IFT172/CEP131/TTC8/IFT140/DYNC2H1/WDR19/MRPL3/IFT20/TTC30B/IFT81/ARL3/KIF24/TUBB4B/CEP83/WDR35/CDK5RAP2/RPGRIP1L/TUBG1/TRAF3IP1/IFT22/CENPJ/BBIP1/IFT46/TMEM67/TTC26/BBS5/GADD45GIP1/TCTN2/FLJ10038/KIF3C/DYNC2LI1/NPHP4/CLUAP1/B9D2	49
1638091	Heparan sulfate/heparin (HS-GAG) metabolism	13/801	49/6750	0.00372198292871393	0.132863284857309	0.126030565404929	NDST1/GPC4/EXT1/SDC4/HGSNAT/SDC2/GLCE/SGSH/VCAN/HS3ST3B1/HS2ST1/HS3ST1/SDC3	13
2022854	Keratan sulfate biosynthesis	9/801	28/6750	0.00379528298335269	0.132863284857309	0.126030565404929	B3GNT7/ST3GAL6/B4GAT1/B4GALT5/B3GNT2/ST3GAL4/CHST2/LUM/ST3GAL3	9
76002	Platelet activation; signaling and aggregation	35/801	189/6750	0.00453511474368197	0.132863284857309	0.126030565404929	PPBP/ARRB2/ITGB3/VAV2/PTK2/APBB1IP/CD9/CALM3/F13A1/SPARC/PLA2G4A/PIK3R6/ARRB1/MAPK3/PIK3CG/CD36/PECAM1/DGKE/RAC2/GNA11/SYK/RASGRP1/P	35

							ROS1/TGFB1/FN1/GNAQ/PRKCB/F8/VAV1/ABCC4/FLNA/DAGLA/PRKCA/F5/SERPINE1	
535734	Fatty acid; triacylglycerol; and ketone body metabolism	25/801	123/6750	0.00454867187105979	0.132863284857309	0.126030565404929	HMGCS1/FDFT1/RXRA/HMGCR/AGPAT2/SLC27A1/FASN/ALAS1/ELOVL5/GLIPR1/CD36/MMAA/TIAM2/SMARCD3/GPD2/LPCAT1/PLIN2/ACACA/ELOVL7/ME1/ACACB/TECR/HADH/OXCT1/TRIB3	25
5260271	Diseases of Immune System	8/801	24/6750	0.00489021999202617	0.132863284857309	0.126030565404929	CD14/TLR4/TLR6/NFKB2/CD36/TLR5/LY96/NFKBIA	8
5602358	Diseases associated with the TLR signaling cascade	8/801	24/6750	0.00489021999202617	0.132863284857309	0.126030565404929	CD14/TLR4/TLR6/NFKB2/CD36/TLR5/LY96/NFKBIA	8
193704	p75 NTR receptor-mediated signalling	19/801	86/6750	0.00496839042751695	0.132863284857309	0.126030565404929	FGD4/VAV2/AKAP13/PSENEN/NET1/TRAF6/TIAM2/CASP3/ARHGAP4/UBB/NFKBIA/ITSN1/VAV1/BAD/BCL2L11/ARHGEF3/KALRN/NGFRAP1/RIPK2	19
193648	NRAGE signals death through JNK	12/801	45/6750	0.00500670578657254	0.132863284857309	0.126030565404929	FGD4/VAV2/AKAP13/NET1/TIAM2/ARHGAP4/ITSN1/VAV1/BAD/BCL2L11/ARHGEF3/KALRN	12
166058	MyD88:Mal cascade initiated on plasma membrane	17/801	74/6750	0.00506887581992261	0.132863284857309	0.126030565404929	FOS/CD14/TLR4/DUSP6/TLR6/NFKB2/MAPK3/CD36/TRAF6/MAP3K1/UBB/LY96/NFKBIA/PELI2/NOD1/PELI1/RIPK2	17
168179	Toll Like Receptor	17/801	74/6750	0.00506887581992261	0.132863284857309	0.126030565404929	FOS/CD14/TLR4/DUSP6/TLR6/NFKB2/MAPK3/CD36/TRAF6/MAP3	17

	TLR1:TLR2 Cascade						K1/UBB/LY96/NFKBIA/PELI2/NOD1/PELI1/RIPK2	
168188	Toll Like Receptor TLR6:TLR2 Cascade	17/801	74/6750	0.00506887581992261	0.132863284857309	0.126030565404929	FOS/CD14/TLR4/DUSP6/TLR6/NFKB2/MAPK3/CD36/TRAF6/MAP3K1/UBB/LY96/NFKBIA/PELI2/NOD1/PELI1/RIPK2	17
181438	Toll Like Receptor 2 (TLR2) Cascade	17/801	74/6750	0.00506887581992261	0.132863284857309	0.126030565404929	FOS/CD14/TLR4/DUSP6/TLR6/NFKB2/MAPK3/CD36/TRAF6/MAP3K1/UBB/LY96/NFKBIA/PELI2/NOD1/PELI1/RIPK2	17
202733	Cell surface interactions at the vascular wall	21/801	99/6750	0.00535555871851939	0.132863284857309	0.126030565404929	DOK2/SELPLG/ITGB3/PTPN6/OLR1/ITGAM/AMICA1/ITGAX/MERTK/ITGAL/PECAM1/TREM1/SLC7A5/PROS1/PIK3R2/ITGB2/FN1/SLC7A6/THBD/PLCG1/SLC7A8	21
975634	Retinoid metabolism and transport	11/801	40/6750	0.00551206420558644	0.132863284857309	0.126030565404929	GPC4/LDLR/LRP1/LRP8/SDC4/SDC2/LPL/APOC2/PLB1/SDC3/AKR1C3	11
71387	Metabolism of carbohydrates	43/801	247/6750	0.00575571111816387	0.132863284857309	0.126030565404929	B3GNT7/ST3GAL6/PYGL/SLC2A3/NDST1/SLC2A1/HK3/GPC4/SEH1L/PFKFB4/B4GAT1/CALM3/EXT1/SDC4/HGSNAT/SDC2/PFKP/GLCE/B4GALT5/TKT/PCK2/SGSH/VCAN/HS3ST3B1/ENO2/UBB/RPIA/HS2ST1/CHST15/EPM2A/PAPSS2/NUP62/PFKFB2/B3GNT2/FBP1/ST3GAL4/CHST2/HS3ST1/SDC3/LUM/CHST14/PRKACB/ST3GAL3	43
196780	Biotin transport and metabolism	5/801	11/1/6750	0.00580672004579291	0.132863284857309	0.126030565404929	MCCC2/BTD/SLC5A6/ACACA/ACACB	5
5602498	MyD88 deficiency (TLR2/4)	5/801	11/1/6750	0.00580672004579291	0.132863284857309	0.126030565404929	CD14/TLR4/TLR6/CD36/LY96	5

5603041	IRAK4 deficiency (TLR2/4)	5/801	11/1/6750	0.00580672004579291	0.132863284857309	0.126030565404929	CD14/TLR4/TLR6/CD36/LY96	5
2029482	Regulation of actin dynamics for phagocytic cup formation	13/801	52/6750	0.00643484072776167	0.143811207892534	0.136415472955731	VAV2/PTK2/HSP90AB1/FCGR3A/HSP90AA1/MAPK3/WIPF1/SYK/WAS/NCKIPSD/VAV1/MYO10/CYFIP2	13
166520	Signalling by NGF	46/801	273/6750	0.00809201946769717	0.174793531058534	0.165804477678613	RALA/MAPK13/FURIN/PIK3CD/FGD4/DUSP6/VAV2/KL/FOXO4/IRS2/AKT1S1/AKAP13/CALM3/MAPK3/PCSK5/PSENEN/NET1/CDKN1A/TRAF6/TIAM2/CASP3/PIK3R2/ARHGAP4/UBB/NFKBIA/CASP9/CD86/ITSN1/CD80/VAV1/BAD/BCL2L11/ARHGEF3/DNAL4/HBEGF/PHLPP1/KALRN/PHLPP2/PLCG1/ADORA2A/PRKCA/PCSK6/TRIB3/PRKACB/NGFRAP1/RIPK2	46
1989781	PPARA activates gene expression	11/801	42/6750	0.00818492080919252	0.174793531058534	0.165804477678613	HMGCS1/FDFT1/HMGCR/SLC27A1/ALAS1/GLIPR1/CD36/TIAM2/PLIN2/ME1/TRIB3	11
204174	Regulation of pyruvate dehydrogenase (PDH) complex	5/801	12/1/6750	0.0089940143870847	0.187825970190381	0.178166701555255	PDK2/PDK4/PDK3/PDK1/PDP2	5
109582	Hemostasis	70/801	450/6750	0.00918607762637661	0.187825970190381	0.178166701555255	PPBP/DOK2/SELPLG/GUCY1A3/ARRB2/ITGB3/SLC8A3/PTPN6/VAV2/PTK2/APBB1IP/OLR1/ATP2A3/CD9/ITGAM/CALM3/F13A1/LRP8/SPARC/PLA2G4A/PIK3R6/AMICA1/ITGAX/ARRB1/MAPK3/MERTK/ITGAL/PIK3CG/CD36/PECAM1/DGKE/TREM1/RAC2/P2RX1/PHF21A/GNA11/SYK/RASGRP1/SLC7A5/P	70

							ROS1/TGFB1/PIK3R2/ITGB2/FN1/ITGA1/GNAQ/IRF1/PRKCB/F8/KIF11/VAV1/PDE9A/ABCC4/SLC7A6/FLNA/THBD/KIF23/ATP2B1/PLCG1/H3F3A/DAGLA/TFPI/SLC7A8/PLAUR/PRKCA/F5/SERPINE1/KIF3C/CENPE/PRKACB	
15869	Metabolism of nucleotides	17/801	79/6750	0.0100162142236912	0.200532955603485	0.190220208721856	AMPD2/PAICS/CTPS1/TK2/CDA/AK2/GART/UCK2/APRT/NME1/ADA/TXN/NUDT1/CAD/CAT/NT5C/TK1	17
211976	Endogenous sterols	6/801	17/6750	0.0106868400274824	0.205401065328211	0.194837967658942	CYP51A1/CYP7B1/CYP27A1/CYP19A1/FDXR/FDX1L	6
418038	Nucleotide-like (purinergic) receptors	6/801	17/6750	0.0106868400274824	0.205401065328211	0.194837967658942	P2RY11/ADORA3/TMIGD3/LPAR6/ADORA2A/P2RY2	6
76005	Response to elevated platelet cytosolic Ca2+	18/801	86/6750	0.0109472121915373	0.206279821883674	0.195671532670203	PPBP/ITGB3/CD9/CALM3/F13A1/SPARC/CD36/PECAM1/PROS1/TGFB1/FN1/PRKCB/F8/ABCC4/FLNA/PRKCA/F5/SERPINE1	18
1638074	Keratan sulfate/keratin metabolism	9/801	33/6750	0.0123177458241013	0.227641418018488	0.215934572543962	B3GNT7/ST3GAL6/B4GAT1/B4GALT5/B3GNT2/ST3GAL4/CHST2/LUM/ST3GAL3	9
982772	Growth hormone receptor signaling	7/801	23/6750	0.0142994218286678	0.257238588520919	0.244009658425014	PRLR/PTPN6/IRS2/MAPK3/SOCS3/SOCS2/SOCS1	7
1368108	BMAL1:CLOCK; NPAS2 activates circadian gene expression	6/801	18/6750	0.0144546137150152	0.257238588520919	0.244009658425014	NOCT/DBP/BHLHE40/BHLHE41/CRY2/SERPINE1	6
2454202	Fc epsilon receptor	30/801	169/6750	0.0147687593054928	0.258050503501428	0.244779819302521	FOS/PIK3CD/VAV2/KL/FOXO4/IRS2/AKT1S1/CALM3/MAPK3/NFATC	30

	(FCERI) signaling						1/LAT2/CDKN1A/TRAF6/SYK/RASGRP1/MAP3K1/PIK3R2/UBB/NFKBIA/PPP3CA/CASP9/CD86/CD80/VAV1/BAD/HBEGF/PHLPP1/PHLPP2/PLCG1/TRIB3	
445355	Smooth Muscle Contraction	8/801	29/6750	0.0167068374579474	0.286701264233704	0.271957166138768	ITGB5/ACTA2/CALM3/MYH11/MYL9/ITGA1/MYLK/MYL6B	8
379716	Cytosolic tRNA aminoacylation	7/801	24/6750	0.0181737990555202	0.301155487927072	0.285668056897798	AIMP2/HARS/KARS/FARSB/PPA1/VARS/WARS	7
5668914	Diseases of metabolism	9/801	35/6750	0.018175877523174	0.301155487927072	0.285668056897798	MCCC2/MMAA/UBB/EPM2A/ACACA/ACACB/MMACHC/FDXR/FDX1L	9
2029480	Fcgamma receptor (FCGR) dependent phagocytosis	15/801	72/6750	0.0199922218553213	0.325636020389216	0.308889636515758	VAV2/PTK2/HSP90AB1/FCGR3A/HSP90AA1/MAPK3/WIPF1/SYK/WAS/PIK3R2/NCKIPSD/VAV1/MYO10/PLCG1/CYFIP2	15
400206	Regulation of lipid metabolism by Peroxisome proliferator-activated receptor alpha (PPARalpha)	13/801	60/6750	0.0215138183179023	0.342867124668103	0.325234602073035	HMGCS1/FDFT1/RXRA/HMGCR/SLC27A1/ALAS1/GLIPR1/CD36/TIAM2/SMARCD3/PLIN2/ME1/TRIB3	13
109606	Intrinsic Pathway for Apoptosis	9/801	36/6750	0.0217636780486517	0.342867124668103	0.325234602073035	TFDP1/CYCS/CASP3/CASP9/BAD/BCL2L11/CASP7/PPP3CC/PMAIP1	9
166054	Activated TLR4 signalling	18/801	93/6750	0.0236732310952991	0.366935081977136	0.348064823913905	FOS/BIRC3/CD14/TLR4/DUSP6/TLR6/NFKB2/MAPK3/CD36/TRAF6/MAP3K1/UBB/LY96/NFKBIA/PELI2/NOD1/PELI1/RIPK2	18

352230	Amino acid transport across the plasma membrane	8/801	31/6750	0.0248257230599347	0.378554448520611	0.359086644853332	SLC16A10/SLC7A1/SLC7A5/SLC7A6/SLC36A1/SLC7A8/SLC38A2/SLC1A4	8
937072	TRAF6 mediated induction of TAK1 complex	5/801	15/6750	0.0252107020867005	0.378554448520611	0.359086644853332	CD14/TLR4/TRAF6/UBB/LY96	5
114608	Platelet degranulation	16/801	81/6750	0.0266772339410489	0.394412643343816	0.374129305148962	PPBP/ITGB3/CD9/CALM3/F13A1/SPARC/CD36/PECAM1/PROS1/TGFB1/FN1/F8/ABCC4/FLNA/F5/SERPINE1	16
1474228	Degradation of the extracellular matrix	20/801	109/6750	0.0302300329631838	0.420088131670247	0.398484388002009	MMP10/FURIN/TIMP2/SPP1/ADAMTS4/MMP9/ADAM8/CASP3/MMP8/FN1/COL6A2/LAMB3/COL6A1/CTSK/COL5A2/LAMB1/MMP2/LAMA3/LAMC1/COL23A1	20
111465	Apoptotic cleavage of cellular proteins	9/801	38/6750	0.0304111146168227	0.420088131670247	0.398484388002009	STK26/PTK2/SPTAN1/TJP1/CASP3/LMNB1/CLSPN/CASP7/PLEC	9
2024096	HS-GAG degradation	6/801	21/6750	0.0310366881879163	0.420088131670247	0.398484388002009	GPC4/SDC4/HGSNAT/SDC2/SGSH/SDC3	6
3371571	HSF1-dependent transactivation	6/801	21/6750	0.0310366881879163	0.420088131670247	0.398484388002009	HSP90AB1/AKT1S1/CAMK2A/HSP90AA1/CAMK2G/HSPA8	6
379726	Mitochondrial tRNA aminoacylation	6/801	21/6750	0.0310366881879163	0.420088131670247	0.398484388002009	KARS/EARS2/AARS2/MARS2/YARS2/WARS2	6
389960	Formation of tubulin folding intermediates by CCT/TriC	6/801	21/6750	0.0310366881879163	0.420088131670247	0.398484388002009	CCT2/TCP1/CCT3/TUBA1C/TUBA1B/TUBB4B	6

198323	AKT phosphorylates targets in the cytosol	4/801	11/1/6750	0.0327567533782463	0.43093346600602	0.408771982650981	AKT1S1/CDKN1A/CASP9/BAD	4
194315	Signaling by Rho GTPases	53/801	349/6750	0.0328204861053092	0.43093346600602	0.408771982650981	SEH1L/TRIP10/FGD4/ARHGAP6/FAM13A/VAV2/ARHGAP9/PTK2/PPP1CB/AKAP13/CALM3/NCF2/MAPK3/ARHGDIB/WIPF1/MYH11/ARHGAP26/NET1/DSN1/RAC2/TIAM2/SRGAP1/MYL9/WAS/PIK3R2/ARHGAP4/ITSN1/NCKIPSD/PRC1/VAV1/ARHGAP20/EVL/NOXA1/ARHGEF3/MAD1L1/CENPF/KALRN/FLNA/SGOL2/MYLK/RHOBTB2/H3F3A/DLG4/ARHGAP18/ARHGAP11A/ARHGAP24/APITD1/ARHGAP22/ZWINT/CENPE/CYFIP2/RHOF/B9D2	53
2173791	TGF-beta receptor signaling in EMT (epithelial to mesenchymal transition)	5/801	16/6750	0.0331832221482263	0.43093346600602	0.408771982650981	TGFBR2/TGFB1/UBB/SMURF1/PARD3	5
1592389	Activation of Matrix Metalloproteinases	7/801	27/6750	0.0340782439341868	0.436655898943381	0.414200129782538	MMP10/FURIN/TIMP2/MMP9/MMP8/CTSK/MMP2	7
446652	Interleukin-1 signaling	10/801	45/6750	0.03498434054283	0.44236777975868	0.419618267452781	IL1R1/IL1R2/MAP3K3/TRAF6/UBB/PELI2/NOD1/IL1A/PELI1/RIPK2	10
422475	Axon guidance	45/801	292/6750	0.037632447155692	0.4656425206244	0.441696065349396	CD72/FES/VASP/ITGB3/NRCAM/RRAS/VAV2/PTK2/HSP90AB1/HSP90AA1/NRP1/MAPK3/PSENEN/SPT	45

							AN1/MYH11/NTN1/MET/MMP9/SDC2/RAC2/SRGAP1/MYL9/PIP5K1C/PRNP/ITGA1/ITGA9/EPHB6/ITSN1/COL6A2/SEMA4D/SLIT2/MYO10/EVL/KALRN/COL6A1/PLCG1/DLG4/NRP2/COL5A2/LAMB1/HSPA8/MMP2/LAMC1/EPHA7/ABLIM3	
373076	Class A/1 (Rhodopsin-like receptors)	47/801	307/6750	0.0377940859611896	0.4656425206244	0.441696065349396	PPBP/CXCL5/CCL19/CCL7/EDN1/CXCL16/P2RY11/CXCL9/C5AR1/CCR7/CXCR4/GPR132/CCRL2/LTB4R/CCL22/SSTR2/GPR65/CXCL13/ADORA3/CXCL10/CCL23/GPR68/FPR3/GPBAR1/C5AR2/CXCL11/OPRL1/FPR2/FPR1/SUCNR1/CCL4/CXCL1/CYSLTR1/LPAR2/TMIGD3/HTR7/S1PR4/LPAR6/OXER1/ADORA2A/GAL/CYSLTR2/P2RY2/CCL3/CXCL6/MC1R/HTR2B	47
445989	TAK1 activates NFkB by phosphorylation and activation of IKKs complex	6/801	22/6750	0.0385307538103806	0.468709549516149	0.444605367085805	NFKB2/TRAF6/UBB/NFKBIA/NOD1/RIPK2	6
373755	Semaphorin interactions	13/801	65/6750	0.0393900212755787	0.47317263057289	0.448838926640147	CD72/FES/RRAS/HSP90AB1/HSP90AA1/NRP1/MYH11/MET/RAC2/MYL9/PIP5K1C/ITGA1/SEMA4D	13
456926	Thrombin signalling through proteinase activated	5/801	17/6750	0.0425567146985363	0.49307167910055	0.467714632894547	ARRB2/ARRB1/MAPK3/GNA11/GNAQ	5

	receptors (PARs)							
1474151	Tetrahydrobiopterin (BH4) synthesis; recycling; salvage and regulation	4/801	12/1/6750	0.0446381228738271	0.49307167910055	0.467714632894547	CALM3/HSP90AA1/SPR/GCHFR	4
210990	PECAM1 interactions	4/801	12/1/6750	0.0446381228738271	0.49307167910055	0.467714632894547	ITGB3/PTPN6/PECAM1/PLCG1	4
264870	Caspase-mediated cleavage of cytoskeletal proteins	4/801	12/1/6750	0.0446381228738271	0.49307167910055	0.467714632894547	SPTAN1/CASP3/CASP7/PLEC	4
3656237	Defective EXT2 causes exostoses 2	4/801	12/1/6750	0.0446381228738271	0.49307167910055	0.467714632894547	GPC4/SDC4/SDC2/SDC3	4
3656253	Defective EXT1 causes exostoses 1; TRPS2 and CHDS	4/801	12/1/6750	0.0446381228738271	0.49307167910055	0.467714632894547	GPC4/SDC4/SDC2/SDC3	4
428790	Facilitative Na+-independent glucose transporters	4/801	12/1/6750	0.0446381228738271	0.49307167910055	0.467714632894547	SLC2A3/SLC2A1/SLC2A9/SLC2A6	4
194138	Signaling by VEGF	18/801	100/6750	0.0453705923042668	0.494085018125619	0.468675859243974	FLT1/MAPK13/ITGB3/VAV2/PTK2/CALM3/HSP90AA1/NCF2/NRP1/MAPK3/SPHK1/PIK3R2/PRKCB/VAV1/PLCG1/NRP2/PRKCA/CYFIP2	18
211945	Phase 1 - Functionalizati	14/801	73/6750	0.0457581338326535	0.494085018125619	0.468675859243974	PTGS1/MAOA/CYP51A1/TBXAS1/SMOX/CYP27B1/CYP7B1/ALDH2/	14

	on of compounds						CYP2S1/CYP27A1/CYP19A1/CYP3A7/FDXR/FDX1L	
180024	DARPP-32 events	6/801	23/6750	0.0470829886274558	0.494289835852822	0.468870143872659	PDE4A/CALM3/PPP3CA/PPP3CC/PDE4B/PRKACB	6
5627123	RHO GTPases activate PAKs	6/801	23/6750	0.0470829886274558	0.494289835852822	0.468870143872659	PPP1CB/CALM3/MYH11/MYL9/FLNA/MYLK	6
390466	Chaperonin-mediated protein folding	7/801	29/6750	0.048665243259684	0.494289835852822	0.468870143872659	CCT2/TCP1/CCT3/SPHK1/TUBA1C/TUBA1B/TUBB4B	7
168142	Toll Like Receptor 10 (TLR10) Cascade	13/801	67/6750	0.0488631991737961	0.494289835852822	0.468870143872659	FOS/DUSP6/NFKB2/MAPK3/TRAF6/MAP3K1/TLR5/UBB/NFKBIA/PELI2/NOD1/PELI1/RIPK2	13
168176	Toll Like Receptor 5 (TLR5) Cascade	13/801	67/6750	0.0488631991737961	0.494289835852822	0.468870143872659	FOS/DUSP6/NFKB2/MAPK3/TRAF6/MAP3K1/TLR5/UBB/NFKBIA/PELI2/NOD1/PELI1/RIPK2	13
975871	MyD88 cascade initiated on plasma membrane	13/801	67/6750	0.0488631991737961	0.494289835852822	0.468870143872659	FOS/DUSP6/NFKB2/MAPK3/TRAF6/MAP3K1/TLR5/UBB/NFKBIA/PELI2/NOD1/PELI1/RIPK2	13

IFN-L4 vs NT M2-MDMs Reactome enrichment

PATH_ID	Description	GeneRatio	BgRatio	pvalue	p.adjust	qvalue	geneID	Count
449147	Signaling by Interleukins	311/2018	107/6750	0.00265741546172367	0.26520264839251	0.240444967424635	IL1R2/NOD1/SOCS3	3
446652	Interleukin-1 signaling	211/2018	45/6750	0.00621335059865272	0.26520264839251	0.240444967424635	IL1R2/NOD1	2
1059683	Interleukin-6 signaling	111/2018	1011/6750	0.0263663034391871	0.26520264839251	0.240444967424635	SOCS3	1

166016	Toll Like Receptor 4 (TLR4) Cascade	211/2018	96/6750	0.0264140833719553	0.26520264839251	0.240444967424635	LBP/NOD1	2
157052	NICD traffics to nucleus	111/2018	1111/6750	0.0289665121243647	0.26520264839251	0.240444967424635	NOTCH3	1
1980148	Signaling by NOTCH3	111/2018	1111/6750	0.0289665121243647	0.26520264839251	0.240444967424635	NOTCH3	1
350054	Notch-HLH transcription pathway	111/2018	1111/6750	0.0289665121243647	0.26520264839251	0.240444967424635	NOTCH3	1
381042	PERK regulates gene expression	111/2018	1111/6750	0.0289665121243647	0.26520264839251	0.240444967424635	CXCL8	1
5668599	RHO GTPases Activate NADPH Oxidases	111/2018	1111/6750	0.0289665121243647	0.26520264839251	0.240444967424635	NCF2	1
3656237	Defective EXT2 causes exostoses 2	111/2018	1211/6750	0.0315601614464841	0.26520264839251	0.240444967424635	SDC2	1
3656253	Defective EXT1 causes exostoses 1; TRPS2 and CHDS	111/2018	1211/6750	0.0315601614464841	0.26520264839251	0.240444967424635	SDC2	1
156988	Receptor-ligand binding initiates the second proteolytic cleavage of Notch receptor	111/2018	13/6750	0.0341472669813553	0.26520264839251	0.240444967424635	NOTCH3	1

168898	Toll-Like Receptors Cascades	211/2018	111/6750	0.0345420992396934	0.26520264839251	0.240444967424635	LBP/NOD1	2
877312	Regulation of IFNG signaling	111/2018	14/6750	0.0367278442701089	0.26520264839251	0.240444967424635	SOCS3	1
1280215	Cytokine Signaling in Immune system	311/2018	284/6750	0.0376431942165174	0.26520264839251	0.240444967424635	IL1R2/NOD1/SOCS3	3
194840	Rho GTPase cycle	211/2018	125/6750	0.0428994695174932	0.26520264839251	0.240444967424635	ARAP3/NET1	2
1912408	Pre-NOTCH Transcription and Translation	111/2018	19/6750	0.0495333485431758	0.26520264839251	0.240444967424635	NOTCH3	1
3560783	Defective B4GALT7 causes EDS; progeroid type	111/2018	19/6750	0.0495333485431758	0.26520264839251	0.240444967424635	SDC2	1
3560801	Defective B3GAT3 causes JDSSDHD	111/2018	19/6750	0.0495333485431758	0.26520264839251	0.240444967424635	SDC2	1

IFN-L4 vs IFN-L3 M2-MDMs Reactome enrichment

PATH_ID	Description	GeneRatio	BgRatio	pvalue	p.adjust	qvalue	geneID	Count
191273	Cholesterol biosynthesis	15/779	23/6750	1.51942025549532E-09	1.41609967812164E-06	1.32589409663749E-06	CYP51A1/DHCR24/DHCR7/EBP/FDFT1/FDPS/HMGCR/HMGCS1/HSD17B7/IDI1/MSMO1/MVD/MVK/SC5D/SQLE	15

2426168	Activation of gene expression by SREBF (SREBP)	14/779	26/6750	1.69220947253809E-07	0.0000788569614202752	0.0000738337711965305	ACACA/ACACB/CYP51A1/DHCR7/FASN/FDFT1/FDPS/HMGCR/HMGCS1/IDI1/MVD/MVK/SC5D/SQLE	14
622312	Inflammasomes	11/779	17/6750	2.90342727174821E-07	0.000090199807242311	0.000084454077483483	BCL2/CASP1/HSP90AB1/MEFV/NLRC4/NLRP1/P2RX7/PSTPIP1/PYCARD/TXN/TXNIP	11
3000170	Syndecan interactions	12/779	25/6750	6.39324924668549E-06	0.00148962707447772	0.00139473779618481	ACTN1/COL1A2/COL5A2/FN1/ITGB3/ITGB5/PRKCA/SDC3/SDC4/TGFB1/TNC/VTN	12
380108	Chemokine receptors bind chemokines	19/779	56/6750	8.11400720972856E-06	0.0015124509438934	0.00141610778460315	CCL19/CCL20/CCL22/CCL28/CCL3/CCL4/CCL7/CCR7/CCRL2/CXCL10/CXCL11/CXCL13/CXCL16/CXCL5/CXCL6/CXCL9/CXCR4/CXCR5/PPBP	19
844456	The NLRP3 inflammasome	8/779	12/1/6750	9.83511563039533E-06	0.00152772129458807	0.00143040541361364	CASP1/HSP90AB1/MEFV/P2RX7/PSTPIP1/PYCARD/TXN/TXNIP	8
1655829	Regulation of cholesterol biosynthesis by SREBP (SREBF)	15/779	39/6750	0.0000130364856291703	0.00173571494376953	0.00162514986264393	ACACA/ACACB/CYP51A1/DHCR7/FASN/FDFT1/FDPS/HMGCR/HMGCS1/IDI1/INSIG1/MVD/MVK/SC5D/SQLE	15
1474244	Extracellular matrix organization	51/779	249/6750	0.0000239369587897201	0.0027886556990024	0.00261101826798395	ACTN1/ADAM8/ADAM9/ADAMTS2/ADAMTS4/BMP2/CASP3/COL1A2/COL23A1/COL4A2/COL5A2/COL6A1/COL6A2/COL9A2/COLGALT2/CTSK/FN1/FURIN/ITGA1/ITGAL/ITGAM/ITGAX/ITGB2/ITGB3/ITGB5/ITGB7/ITGB8/LAMA3/LAMB1/LAMB3/LAMC1/LTBP3/LUM/MMP10/MMP12/M	51

							MP2/MMP8/MMP9/PLOD1/PLOD2/PRKCA/SDC3/SDC4/SERPINE1/SPARC/SPP1/TGFB1/TIMP2/TNC/VCAN/VTN	
1989781	PPARA activates gene expression	15/779	42/6750	0.0000366500873868923	0.00379532016050929	0.00355355818055365	ABCA1/ABCB4/ALAS1/CD36/FDFT1/G0S2/GLIPR1/HMGCR/HMGCS1/ME1/PEX11A/PLIN2/SLC27A1/TIAM2/TRIB3	15
168643	Nucleotide-binding domain; leucine rich repeat containing receptor (NLR) signaling pathways	15/779	44/6750	0.0000683869657548844	0.00637366520835523	0.00596766259061044	BCL2/CASP1/HSP90AB1/IRAK2/MAP2K6/MEFV/NLRC4/NLRP1/P2RX7/PSTPIP1/PYCARD/RIPK2/TNFAIP3/TXN/TXNIP	15
5620924	Intraflagellar transport	13/779	37/6750	0.000146791426668144	0.0124372372413373	0.0116449849481236	CLUAP1/DYNC2H1/IFT140/IFT172/IFT20/IFT22/IFT57/IFT81/TRAF3IP1/TTC26/TTC30B/WDR19/WDR35	13
3000171	Non-integrin membrane-ECM interactions	16/779	53/6750	0.000203023422389776	0.0157681524722726	0.0147637208036074	ACTN1/COL1A2/COL4A2/COL5A2/FN1/ITGB3/ITGB5/LAMA3/LAMB1/LAMC1/PRKCA/SDC3/SDC4/TGFB1/TNC/VTN	16
2029481	FCGR activation	7/779	13/6750	0.0002431783777199	0.0174340190796113	0.0163234716704289	FCGR1A/FCGR2A/FCGR2C/FCGR3A/SRC/SYK/YES1	7
400206	Regulation of lipid metabolism by	17/779	60/6750	0.000303023032951569	0.020172676193633	0.0188876762644249	ABCA1/ABCB4/ALAS1/CD36/FDFT1/G0S2/GLIPR1/HMGCR/HMGCS1/ME1/PEX11A/PLIN2/RXR	17

	Peroxisome proliferator-activated receptor alpha (PPARalpha)						A/SLC27A1/SMARCD3/TIAM2/TRIB3	
216083	Integrin cell surface interactions	21/779	83/6750	0.000351604267829362	0.0218463451744643	0.0204547324933713	COL1A2/COL23A1/COL4A2/COL5A2/COL6A1/COL6A2/COL9A2/FN1/ITGA1/ITGAL/ITGAM/ITGAX/ITGB2/ITGB3/ITGB5/ITGB7/ITGB8/LUM/SPP1/TNC/VTN	21
5617833	Assembly of the primary cilium	34/779	163/6750	0.000380177135084042	0.0221453181186454	0.0207346608542547	ARL3/BBS1/BBS9/CDK1/CENPJ/CEP131/CEP135/CEP83/CLUAP1/DYNC2H1/HSP90AA1/IFT140/IFT172/IFT20/IFT22/IFT57/IFT81/KIF24/LZTFL1/PLK1/PRKAR2B/RAB3IP/TCTN2/TMEM67/TRAF3IP1/TTC26/TTC30B/TTC8/TUBB/TUBB4B/TUBG1/UNC119B/WDR19/WDR35	34
3000178	ECM proteoglycans	18/779	67/6750	0.000418159382647119	0.0229249732133597	0.0214646519018242	COL1A2/COL4A2/COL5A2/COL6A1/COL6A2/FN1/ITGAX/ITGB3/ITGB5/LAMA3/LAMB1/LAMC1/SERPINE1/SPARC/TGFB1/TNC/VCAN/VTN	18
1474228	Degradation of the extracellular matrix	25/779	109/6750	0.000513281538402347	0.0259795219032311	0.0243246257711527	ADAM8/ADAM9/ADAMTS4/CASP3/COL1A2/COL23A1/COL4A2/COL5A2/COL6A1/COL6A2/COL9A2/CTSK/FN1/FURIN/LAMA3/LAMB1/LAMB3/LAMC1/MMP10/MMP12/MMP2/MMP8/MMP9/SPP1/TIMP2	25

2022928	HS-GAG biosynthesis	10/779	27/6750	0.000529625446525098	0.0259795219032311	0.0243246257711527	EXT1/GLCE/GPC4/HS2ST1/HS3ST1/HS3ST3A1/HS3ST3B1/NDST1/SDC3/SDC4	10
535734	Fatty acid; triacylglycerol; and ketone body metabolism	27/779	123/6750	0.00065088264452859	0.0283990374902211	0.0283990374902211	ABCA1/ABCB4/ACACA/ACACB/AGPAT2/ALAS1/BDH1/CD36/ELOVL3/ELOVL7/FASN/FDFT1/G0S2/GLIPR1/HADH/HMGCR/HMGCS1/LCLAT1/ME1/OXCT1/PEX11A/PLIN2/RXRA/SLC27A1/SMARCD3/TIAM2/TRIB3	27
5260271	Diseases of Immune System	9/779	24/6750	0.000907539458681811	0.0384466716132476	0.0359976177630249	CD14/CD36/LY96/NFKB1/NFKB2/NFKBIA/TLR4/TLR5/TLR6	9
69273	Cyclin A/B1 associated events during G2/M transition	7/779	16/6750	0.00118597297851592	0.048057687651167	0.0449964118622289	CCNA1/CCNA2/CCNB1/CCNB2/CDC25B/CDK1/FOXM1	7
109582	Hemostasis	73/779	450/6750	0.00129169501489857	0.0501608230785613	0.0469655775153911	ABCC4/ACTN1/AMICA1/APBB1IP/ARRB2/ATP2A3/ATP2B1/ATP2B4/CALM3/CD36/CD9/CENPE/COL1A2/DAGLA/DGKE/DGKH/DOK2/F13A1/F8/FN1/GAS6/GNA11/GUCY1A3/IRF1/ITGA1/ITGAL/ITGAM/ITGAX/ITGB2/ITGB3/ITPR2/KIF11/KIF18A/KIF23/KIF2C/KIF4A/LRP8/MAFF/MAPK3/MERTK/OLR1/P2RX1/PDE9A/PHF21A/PIK3CG/PIK3R2/PIK3R5/PIK3R6/PPBP/PRKACB/PRKAR2B/PRKCA/PRKCB/PROS1/PTK2/PTPN6/RAC2/RASGRP1/SELPLG/SERPINB2/SERPINE1/SLC7A5/S	73

							LC8A3/SPARC/SRC/SYK/TFPI/TGFB1/THBD/TREM1/VAV1/VAV2/YES1	
76002	Platelet activation; signaling and aggregation	36/779	189/6750	0.00151756252696161	0.0565747310051288	0.0529709193621547	ABCC4/ACTN1/APBB1IP/ARRB2/CALM3/CD36/CD9/COL1A2/DAGLA/DGKE/DGKH/F13A1/F8/FN1/GAS6/GNA11/ITGB3/ITPR2/MAPK3/PIK3CG/PIK3R5/PIK3R6/PPBP/PRKCA/PRKCB/PROS1/PTK2/RAC2/RASGRP1/SERPINE1/SPARC/SRC/SYK/TGFB1/VAV1/VAV2	36
168898	Toll-Like Receptors Cascades	24/779	111/6750	0.00158814184352797	0.056928776852618	0.0533024124811614	BIRC3/CD14/CD36/CDK1/CNPY3/CTSK/DUSP6/FOS/IRAK2/ITGAM/ITGB2/LY96/MAP2K6/MAPK3/NFKB1/NFKB2/NFKBIA/PELI1/PELI2/RIPK2/TLR4/TLR5/TLR6/TLR7	24
109606	Intrinsic Pathway for Apoptosis	11/779	36/6750	0.00177860068791331	0.0613946607827854	0.0574838195040988	BAD/BCL2/BCL2L11/BMF/CASP3/CASP7/CYCS/GZMB/PMAIP1/PPP3CC/TFDP1	11
5362517	Signaling by Retinoic Acid	12/779	42/6750	0.00211964898843251	0.0705540306149678	0.0660597372710733	CRABP1/DHRS3/DHRS9/FABP5/PDK1/PDK2/PDK3/PDK4/PPARD/RARA/RDH10/RXRA	12
2029480	Fcgamma receptor (FCGR) dependent phagocytosis	17/779	72/6750	0.00280382865313848	0.0901092518870712	0.0843692905064175	CYFIP2/FCGR1A/FCGR2A/FCGR2C/FCGR3A/HSP90AA1/HSP90AB1/ITPR2/MAPK3/MYO10/PIK3R2/PTK2/SRC/SYK/VAV1/VAV2/YES1	17
2022854	Keratan sulfate	9/779	28/6750	0.0031346159874734	0.0973820700108404	0.0911788299514194	B3GNT2/B3GNT7/B4GALT5/B4GALT6/B4GAT1/CHST2/LUM/ST3GAL4/ST3GAL6	9

	biosynthesis							
1442490	Collagen degradation	15/779	62/6750	0.00379737032669612	0.0999906483134758	0.0936212417572526	ADAM9/COL1A2/COL23A1/COL4A2/COL5A2/COL6A1/COL6A2/COL9A2/CTSK/FURIN/MMP10/MMP12/MMP2/MMP8/MMP9	15
166058	MyD88:Mal cascade initiated on plasma membrane	17/779	74/6750	0.00380471236437311	0.0999906483134758	0.0936212417572526	CD14/CD36/CDK1/DUSP6/FOS/IRAK2/LY96/MAP2K6/MAPK3/NFKB1/NFKB2/NFKBIA/PELI1/PELI2/RIPK2/TLR4/TLR6	17
168179	Toll Like Receptor TLR1:TLR2 Cascade	17/779	74/6750	0.00380471236437311	0.0999906483134758	0.0936212417572526	CD14/CD36/CDK1/DUSP6/FOS/IRAK2/LY96/MAP2K6/MAPK3/NFKB1/NFKB2/NFKBIA/PELI1/PELI2/RIPK2/TLR4/TLR6	17
168188	Toll Like Receptor TLR6:TLR2 Cascade	17/779	74/6750	0.00380471236437311	0.0999906483134758	0.0936212417572526	CD14/CD36/CDK1/DUSP6/FOS/IRAK2/LY96/MAP2K6/MAPK3/NFKB1/NFKB2/NFKBIA/PELI1/PELI2/RIPK2/TLR4/TLR6	17
181438	Toll Like Receptor 2 (TLR2) Cascade	17/779	74/6750	0.00380471236437311	0.0999906483134758	0.0936212417572526	CD14/CD36/CDK1/DUSP6/FOS/IRAK2/LY96/MAP2K6/MAPK3/NFKB1/NFKB2/NFKBIA/PELI1/PELI2/RIPK2/TLR4/TLR6	17
202733	Cell surface interactions at the vascular wall	21/779	99/6750	0.00386229972026301	0.0999906483134758	0.0936212417572526	AMICA1/COL1A2/DOK2/FN1/GAS6/ITGAL/ITGAM/ITGAX/ITGB2/ITGB3/MERTK/OLR1/PIK3R2/PROS1/PTPN6/SELPLG/SLC7A5/SRC/THBD/TREM1/YES1	21
193648	NRAGE signals death through JNK	12/779	45/6750	0.00398368922907873	0.100345901662199	0.0939538654596376	AKAP13/ARHGAP4/ARHGEF3/BAD/BCL2L11/FGD4/KALRN/NET1/NGFR/TIAM2/VAV1/VAV2	12

2173782	Binding and Uptake of Ligands by Scavenger Receptors	11/779	40/6750	0.00444937226079241	0.109126709133119	0.102175335296313	CD163/CD36/COL1A2/COL4A2/COLEC12/HSP90AA1/LRP1/MARCO/SCARB1/SCARF1/SPARC	11
2029482	Regulation of actin dynamics for phagocytic cup formation	13/779	52/6750	0.00507908499687005	0.116853334453895	0.109409774409621	CYFIP2/FCGR1A/FCGR2A/FCGR2C/FCGR3A/HSP90AA1/HSP90AB1/MAPK3/MYO10/PTK2/SYK/VAV1/VAV2	13
5602498	MyD88 deficiency (TLR2/4)	5/779	11/1/6750	0.0051405436830576	0.116853334453895	0.109409774409621	CD14/CD36/LY96/TLR4/TLR6	5
5603041	IRAK4 deficiency (TLR2/4)	5/779	11/1/6750	0.0051405436830576	0.116853334453895	0.109409774409621	CD14/CD36/LY96/TLR4/TLR6	5
166016	Toll Like Receptor 4 (TLR4) Cascade	20/779	96/6750	0.00586663455216367	0.130183414348013	0.121890727913376	BIRC3/CD14/CD36/CDK1/DUSP6/FOS/IRAK2/ITGAM/ITGB2/LY96/MAP2K6/MAPK3/NFKB1/NFKB2/NFKBIA/PELI1/PELI2/RIPK2/TLR4/TLR6	20
1630316	Glycosaminoglycan metabolism	23/779	116/6750	0.00619005480661522	0.134165839064311	0.125619472085288	B3GNT2/B3GNT7/B4GALT5/B4GALT6/B4GAT1/CHST13/CHST15/CHST2/CHST3/EXT1/GLCE/GPC4/HS2ST1/HS3ST1/HS3ST3A1/HS3ST3B1/LUM/NDST1/SDC3/SDC4/ST3GAL4/ST3GAL6/VCAN	23
156711	Polo-like kinase mediated events	6/779	16/6750	0.0066868104165244	0.141638802459108	0.132616407543032	CCNB1/CCNB2/CENPF/FOXM1/LIN9/PLK1	6

2029485	Role of phospholipids in phagocytosis	7/779	21/6750	0.00719083765571174	0.148930237669407	0.139443378165732	FCGR1A/FCGR2A/FCGR2C/FCGR3A/ITPR2/PIK3R2/SYK	7
166520	Signalling by NGF	45/779	273/6750	0.00795046844447271	0.155044788799061	0.145168432250306	ADORA2A/AKAP13/AKT1S1/ARHGAP4/ARHGEF3/BAD/BCL2L11/CALM3/CASP3/CD80/CD86/CDK1/CDKN1A/DNAL4/DUSP6/FGD4/FOXO4/FURIN/HBEGF/IRS2/ITPR2/KALRN/KL/MAPK13/MAPK3/NET1/NFKB1/NFKBIA/NGFR/NGFRAP1/PCSK5/PCSK6/PHLPP1/PIK3CD/PIK3R2/PRKACB/PRKAR2B/PRKCA/RALA/RIPK2/SRC/TIAM2/TRIB3/VAV1/VAV2	45
428790	Facilitative Na+-independent glucose transporters	5/779	12/1/6750	0.00798513933729068	0.155044788799061	0.145168432250306	SLC2A1/SLC2A3/SLC2A5/SLC2A6/SLC2A9	5
5620922	BBSome-mediated cargo-targeting to cilium	5/779	12/1/6750	0.00798513933729068	0.155044788799061	0.145168432250306	BBS1/BBS9/LZTFL1/RAB3IP/TTC8	5
204998	Cell death signalling via NRAGE; NRIF and NADE	14/779	61/6750	0.008327660201585	0.158395496079127	0.148305699400945	AKAP13/ARHGAP4/ARHGEF3/BAD/BCL2L11/CASP3/FGD4/KALRN/NET1/NGFR/NGFRAP1/TIAM2/VAV1/VAV2	14
5218921	VEGFR2 mediated	8/779	27/6750	0.00907701415429655	0.167452492370556	0.156785764824024	CALM3/CDK1/ITPR2/MAPK3/PRKCA/PRKCB/SPHK1/SRC	8

	cell proliferation							
194138	Signaling by VEGF	20/779	100/6750	0.00933571526798399	0.167452492370556	0.156785764824024	CALM3/CDK1/CYFIP2/FLT1/HSP90AA1/ITGB3/ITPR2/MAPK13/MAPK3/NCF2/NRP1/NRP2/PIK3R2/PRKCA/PRKCB/PTK2/SPHK1/SRC/VAV1/VAV2	20
418038	Nucleotide-like (purinergic) receptors	6/779	17/6750	0.00934284292196235	0.167452492370556	0.156785764824024	ADORA2A/ADORA3/P2RY11/P2RY13/P2RY2/TMIGD3	6
1638074	Keratan sulfate/keratin metabolism	9/779	33/6750	0.0103300219634185	0.18165246169634	0.170081195782997	B3GNT2/B3GNT7/B4GALT5/B4GALT6/B4GAT1/CHST2/LUM/ST3GAL4/ST3GAL6	9
373076	Class A/1 (Rhodopsin-like receptors)	49/779	307/6750	0.0106052178982213	0.183038205206338	0.171378667400106	ADORA2A/ADORA3/C5AR1/C5AR2/CCL19/CCL20/CCL22/CCL23/CCL28/CCL3/CCL4/CCL7/CCR7/CCRL2/CXCL10/CXCL11/CXCL13/CXCL16/CXCL5/CXCL6/CXCL9/CXCR4/CXCR5/CYSLTR1/CYSLTR2/EDN1/EDNRB/FPR1/FPR2/GPBAR1/GPR132/GPR65/GPR68/HRH2/HTR2B/HTR7/LTB4R/MC1R/OPRL1/OXER1/P2RY11/P2RY13/P2RY2/PPBP/PTGER4/S1PR4/SSTR2/SUCNR1/TMIGD3	49
446652	Interleukin-1 signaling	11/779	45/6750	0.0114394087748676	0.188364179062357	0.17636537660119	IL1A/IL1R1/IL1R2/IRAK2/MAP2K6/MAP3K3/NFKB1/PELI1/PELI2/PELI3/RIPK2	11
114604	GPVI-mediated	8/779	28/6750	0.0114539734210258	0.188364179062357	0.17636537660119	COL1A2/PIK3CG/PIK3R5/PIK3R6/RAC2/SYK/VAV1/VAV2	8

	activation cascade							
383280	Nuclear Receptor transcription pathway	12/779	51/6750	0.0115201268310669	0.188364179062357	0.17636537660119	NR2F6/NR3C2/NR4A2/NR4A3/NR6A1/PPARD/RARA/RORA/RXRA/THRA/THRB/VDR	12
114452	Activation of BH3-only proteins	7/779	23/6750	0.0123502704175739	0.195092407274218	0.182665016523974	BAD/BCL2/BCL2L11/BMF/PMAIP1/PPP3CC/TFDP1	7
180024	DARPP-32 events	7/779	23/6750	0.0123502704175739	0.195092407274218	0.182665016523974	CALM3/PDE4A/PDE4B/PPP3CA/PPP3CC/PRKACB/PRKAR2B	7
1368108	BMAL1:CLOCK;NPAS2 activates circadian gene expression	6/779	18/6750	0.0126735673817249	0.196862746662793	0.184322585253508	BHLHE41/CRY2/DBP/NOCT/RORA/SERPINE1	6
194840	Rho GTPase cycle	23/779	125/6750	0.0151687209721793	0.231758163050346	0.216995162828933	AKAP13/ARHGAP11A/ARHGAP18/ARHGAP20/ARHGAP24/ARHGAP26/ARHGAP4/ARHGAP6/ARHGAP9/ARHGDIB/ARHGEF3/FAM13A/FGD4/KALRN/NET1/PIK3R2/RAC2/RHOBTB2/SRGAP1/TIAM2/TRIP10/VAV1/VAV2	23
418360	Platelet calcium homeostasis	6/779	19/6750	0.0167557942545601	0.249302627851118	0.233422044825589	ATP2A3/ATP2B1/ATP2B4/ITPR2/P2RX1/SLC8A3	6
69231	Cyclin D associated events in G1	8/779	30/6750	0.0175339809175171	0.249302627851118	0.233422044825589	CDK6/CDKN1A/CDKN2B/CDKN2C/CDKN2D/E2F2/TFDP1/TFDP2	8

193704	p75 NTR receptor-mediated signalling	17/779	86/6750	0.0176544779379547	0.249302627851118	0.233422044825589	AKAP13/ARHGAP4/ARHGEF3/BAD/BCL2L11/CASP3/FGD4/KALRN/NET1/NFKB1/NFKBIA/NGFR/NGFRAP1/RIPK2/TIAM2/VAV1/VAV2	17
76005	Response to elevated platelet cytosolic Ca2+	17/779	86/6750	0.0176544779379547	0.249302627851118	0.233422044825589	ABCC4/ACTN1/CALM3/CD36/CD9/F13A1/F8/FN1/GAS6/ITGB3/PPBP/PRKCA/PRKCB/PROS1/SERPINE1/SPARC/TGFB1	17
166054	Activated TLR4 signalling	18/779	93/6750	0.0183149963702146	0.254769800254328	0.238540958223219	BIRC3/CD14/CD36/CDK1/DUSP6/FOS/IRAK2/LY96/MAP2K6/MAPK3/NFKB1/NFKB2/NFKBIA/PELI1/PELI2/RIPK2/TLR4/TLR6	18
379724	tRNA Aminoacylation	10/779	42/6750	0.0186719696665109	0.255915819546884	0.239613976060952	AARS2/AIMP2/EARS2/FARSB/HARS/MARS2/PPA1/VARS/WARS/WARS2	10
186797	Signaling by PDGF	30/779	177/6750	0.0189757378449314	0.255965678759421	0.239660659240524	AKT1S1/BAD/CALM3/CD80/CD86/CDK1/CDKN1A/COL4A2/COL5A2/COL6A1/COL6A2/COL9A2/FOXO4/FURIN/HBEGF/IRS2/ITPR2/KL/MAPK3/PDGFC/PHLPP1/PIK3CD/PIK3R2/PRKACB/PRKAR2B/PRKCA/SPP1/SRC/TRIB3/VAV1	30
449147	Signaling by Interleukins	20/779	107/6750	0.0192248900355788	0.255965678759421	0.239660659240524	CASP1/CDK1/HAVCR2/IL1A/IL1R1/IL1R2/IL7/IRAK2/MAP2K6/MAP3K3/MAPK3/NFKB1/PELI1/PELI2/PELI3/PIK3CD/PTPN6/RIPK2/SYK/VAV1	20
418359	Reduction of cytosolic Ca++ levels	4/779	10/1/6750	0.0208810845390267	0.274100997047506	0.256640757344005	ATP2A3/ATP2B1/ATP2B4/SLC8A3	4

1638091	Heparan sulfate/heparin (HS-GAG) metabolism	11/779	49/6750	0.0214548293978819	0.277720847205916	0.260030022965557	EXT1/GLCE/GPC4/HS2ST1/HS3ST1/HS3ST3A1/HS3ST3B1/NDST1/SDC3/SDC4/VCAN	11
354194	GRB2:SOS provides linkage to MAPK signaling for Integrins	5/779	15/6750	0.022573676680075	0.280515555544399	0.26264670831975	APBB1IP/FN1/ITGB3/PTK2/SRC	5
372708	p130Cas linkage to MAPK signaling for integrins	5/779	15/6750	0.022573676680075	0.280515555544399	0.26264670831975	APBB1IP/FN1/ITGB3/PTK2/SRC	5
391903	Eicosanoid ligand-binding receptors	5/779	15/6750	0.022573676680075	0.280515555544399	0.26264670831975	CYSLTR1/CYSLTR2/LTB4R/OXER1/PTGER4	5
69275	G2/M Transition	20/779	109/6750	0.0231828315123216	0.28429472328268	0.266185142987737	AURKA/CCNA1/CCNA2/CCNB1/CCNB2/CDC25B/CDK1/CENPF/CENPJ/CEP131/CEP135/FOXM1/HSP90AA1/LIN9/PLK1/PPP1CB/PRKAR2B/TUBB/TUBB4B/TUBG1	20
453274	Mitotic G2-G2/M phases	20/779	111/6750	0.0277368957097702	0.3354714833798	0.314101942310655	AURKA/CCNA1/CCNA2/CCNB1/CCNB2/CDC25B/CDK1/CENPF/CENPJ/CEP131/CEP135/FOXM1/HSP90AA1/LIN9/PLK1/PPP1CB/	20

							PRKAR2B/TUBB/TUBB4B/TUBG1	
1592389	Activation of Matrix Metalloproteinases	7/779	27/6750	0.0297768809291938	0.3354714833798	0.314101942310655	CTSK/FURIN/MMP10/MMP2/MMP8/MMP9/TIMP2	7
354192	Integrin alphaIIb beta3 signaling	7/779	27/6750	0.0297768809291938	0.3354714833798	0.314101942310655	APBB1IP/FN1/ITGB3/PTK2/RASGRP1/SRC/SYK	7
162658	Golgi Cisternae Pericentriolar Stack Reorganization	4/779	11/1/6750	0.0298756793138663	0.3354714833798	0.314101942310655	CCNB1/CCNB2/CDK1/PLK1	4
2514853	Condensation of Prometaphase Chromosomes	4/779	11/1/6750	0.0298756793138663	0.3354714833798	0.314101942310655	CCNB1/CCNB2/CDK1/NCAPG	4
389513	CTLA4 inhibitory signaling	4/779	11/1/6750	0.0298756793138663	0.3354714833798	0.314101942310655	CD80/CD86/SRC/YES1	4
397795	G-protein beta:gamma signalling	4/779	11/1/6750	0.0298756793138663	0.3354714833798	0.314101942310655	PIK3CG/PIK3R5/PIK3R6/PLCB2	4
1566948	Elastic fibre formation	9/779	39/6750	0.0303293153248879	0.336510974795185	0.3150752180994	BMP2/FN1/FURIN/ITGB3/ITGB5/ITGB8/LTBP3/TGFB1/VTN	9
4420097	VEGFA-VEGFR2 Pathway	17/779	92/6750	0.0324901511883428	0.356244951853359	0.333552140373203	CALM3/CDK1/CYFIP2/HSP90AA1/ITGB3/ITPR2/MAPK13/MAPK3/NCF2/PIK3R2/PRKCA/PRKCB/PTK2/SPHK1/SRC/VAV1/VAV2	17

5339562	Uptake and actions of bacterial toxins	6/779	22/6750	0.0341708142202586	0.366059756934264	0.342741742148752	CD9/FURIN/HBEGF/MAP2K6/STX1A/STX1B	6
983189	Kinesins	6/779	22/6750	0.0341708142202586	0.366059756934264	0.342741742148752	CENPE/KIF11/KIF18A/KIF23/KIF2C/KIF4A	6
1280218	Adaptive Immune System	62/779	430/6750	0.0347945091828183	0.36850548361803	0.345031675987517	AKT1S1/AMICA1/AP1S3/BAD/CALM3/CD200R1/CD274/CD36/CD4/CD40/CD80/CD86/CDKN1A/CENPE/DYNC2H1/FCGR1A/FCGR2B/FCGR3A/FOXO4/FYB/HBEGF/HCST/HLA-DMA/HLA-DMB/HLA-DOB/ICOSLG/IRS2/ITGAL/ITGB2/ITGB5/ITGB7/ITPR2/KIF11/KIF18A/KIF23/KIF2C/KIF4A/KL/LILRA1/NCF2/NFKB1/NFKBIA/NFKBIE/PDCD1LG2/PHLPP1/PIK3CD/PIK3R2/PRKACB/PRKCB/PSMA2/PSMA6/PSME2/PTPN6/PVR/RASGRP1/RIPK2/SRC/SYK/TRIB3/VASP/VAV1/YES1	62
975634	Retinoid metabolism and transport	9/779	40/6750	0.035277535340887	0.369423179075356	0.345890914223481	APOC2/GPC4/LDLR/LPL/LRP1/LRP8/PLB1/SDC3/SDC4	9
71387	Metabolism of carbohydrates	38/779	247/6750	0.0378059702520161	0.391501825276434	0.366563150162823	B3GNT2/B3GNT7/B4GALT5/B4GALT6/B4GAT1/CALM3/CHST13/CHST15/CHST2/CHST3/ENO2/EPM2A/EXT1/GLCE/GPC4/HK3/HS2ST1/HS3ST1/HS3ST3A1/HS3ST3B1/LUM/NDST1/PCK2/PFKFB2/PFKFB4/PRKACB/PYGL/SDC3/SDC4/SEH1L/SLC25A13/SLC	38

							2A1/SLC2A3/SLC2A5/ST3GAL4/ST3GAL6/TKT/VCAN	
211976	Endogenous sterols	5/779	17/6750	0.0383167031126357	0.39243040990084	0.367432583925679	CYP19A1/CYP27A1/CYP51A1/CYP7B1/FDX1L	5
168142	Toll Like Receptor 10 (TLR10) Cascade	13/779	67/6750	0.0403769178504327	0.396303382808412	0.371058848371554	CDK1/DUSP6/FOS/IRAK2/MAP2K6/MAPK3/NFKB1/NFKB2/NFKBIA/PELI1/PELI2/RIPK2/TLR5	13
168176	Toll Like Receptor 5 (TLR5) Cascade	13/779	67/6750	0.0403769178504327	0.396303382808412	0.371058848371554	CDK1/DUSP6/FOS/IRAK2/MAP2K6/MAPK3/NFKB1/NFKB2/NFKBIA/PELI1/PELI2/RIPK2/TLR5	13
975871	MyD88 cascade initiated on plasma membrane	13/779	67/6750	0.0403769178504327	0.396303382808412	0.371058848371554	CDK1/DUSP6/FOS/IRAK2/MAP2K6/MAPK3/NFKB1/NFKB2/NFKBIA/PELI1/PELI2/RIPK2/TLR5	13
204174	Regulation of pyruvate dehydrogenase (PDH) complex	4/779	12/1/6750	0.040820949302154	0.396303382808412	0.371058848371554	PDK1/PDK2/PDK3/PDK4	4
210990	PECAM1 interactions	4/779	12/1/6750	0.040820949302154	0.396303382808412	0.371058848371554	ITGB3/PTPN6/SRC/YES1	4
174824	Lipoprotein metabolism	6/779	23/6750	0.041872182621206	0.397040889261895	0.37174937564729	ABCA1/ABCG1/APOC2/LDLR/LPL/SCARB1	6
114608	Platelet degranulation	15/779	81/6750	0.0421334254812747	0.397040889261895	0.37174937564729	ABCC4/ACTN1/CALM3/CD36/CD9/F13A1/F8/FN1/GAS6/ITGB3/PPBP/PROS1/SERPINE1/SPARC/TGFB1	15
2129379	Molecules associated	8/779	35/6750	0.0421749442456305	0.397040889261895	0.37174937564729	BMP2/FN1/ITGB3/ITGB5/ITGB8/LTBP3/TGFB1/VTN	8

	with elastic fibres							
445355	Smooth Muscle Contraction	7/779	29/6750	0.0427651267723292	0.398570981518109	0.37318200099222	ACTA2/CALM3/ITGA1/ITGB5/MYH11/MYL9/MYLK	7
975138	TRAF6 mediated induction of NFkB and MAP kinases upon TLR7/8 or 9 activation	13/779	68/6750	0.0448731451395755	0.414076943268162	0.387700232628537	CDK1/DUSP6/FOS/IRAK2/MAP2K6/MAPK3/NFKB1/NFKB2/NFKBIA/PELI1/PELI2/RIPK2/TLR7	13
168181	Toll Like Receptor 7/8 (TLR7/8) Cascade	13/779	69/6750	0.0497069691756176	0.444287283436806	0.415986173446027	CDK1/DUSP6/FOS/IRAK2/MAP2K6/MAPK3/NFKB1/NFKB2/NFKBIA/PELI1/PELI2/RIPK2/TLR7	13
975155	MyD88 dependent cascade initiated on endosome	13/779	69/6750	0.0497069691756176	0.444287283436806	0.415986173446027	CDK1/DUSP6/FOS/IRAK2/MAP2K6/MAPK3/NFKB1/NFKB2/NFKBIA/PELI1/PELI2/RIPK2/TLR7	13
